# Molecular Editing of Pyrroles via a Skeletal Recasting Strategy

**DOI:** 10.1021/acscentsci.3c00812

**Published:** 2023-08-15

**Authors:** Xueting Zhou, Qingqin Huang, Jiami Guo, Lei Dai, Yixin Lu

**Affiliations:** † Joint School of National University of Singapore and Tianjin University, International Campus of Tianjin University, Binhai New City, Fuzhou, Fujian 350207, China; ‡ Department of Chemistry, National University of Singapore, 3 Science Drive 3, Singapore 117543, Singapore

## Abstract

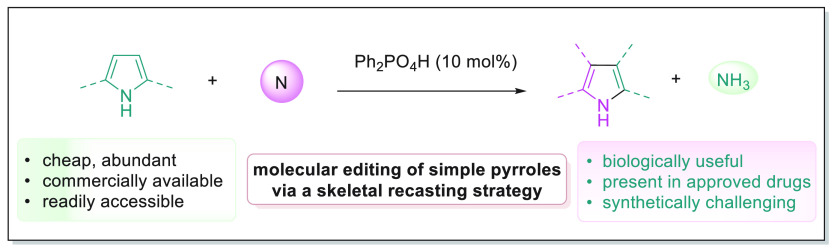

Heterocyclic scaffolds are commonly found in numerous biologically active molecules, therapeutic agents, and agrochemicals. To probe chemical space around heterocycles, many powerful molecular editing strategies have been devised. Versatile C–H functionalization strategies allow for peripheral modifications of heterocyclic motifs, often being specific and taking place at multiple sites. The past few years have seen the quick emergence of exciting “single-atom skeletal editing” strategies, through one-atom deletion or addition, enabling ring contraction/expansion and structural diversification, as well as scaffold hopping. The construction of heterocycles via deconstruction of simple heterocycles is unknown. Herein, we disclose a new molecular editing method which we name the skeletal recasting strategy. Specifically, by tapping on the 1,3-dipolar property of azoalkenes, we recast simple pyrroles to fully substituted pyrroles, through a simple phosphoric acid-promoted one-pot reaction consisting of dearomative deconstruction and rearomative reconstruction steps. The reaction allows for easy access to synthetically challenging tetra-substituted pyrroles which are otherwise difficult to synthesize. Furthermore, we construct N–N axial chirality on our pyrrole products, as well as accomplish a facile synthesis of the anticancer drug, Sutent. The potential application of this method to other heterocycles has also been demonstrated.

## Abstract 2

We present a “skeletal recasting” strategy for synthesis of fully substituted pyrroles from simple pyrroles via sequential dearomative deconstruction and rearomative reconstruction steps.

## Introduction

**Figure 1:**
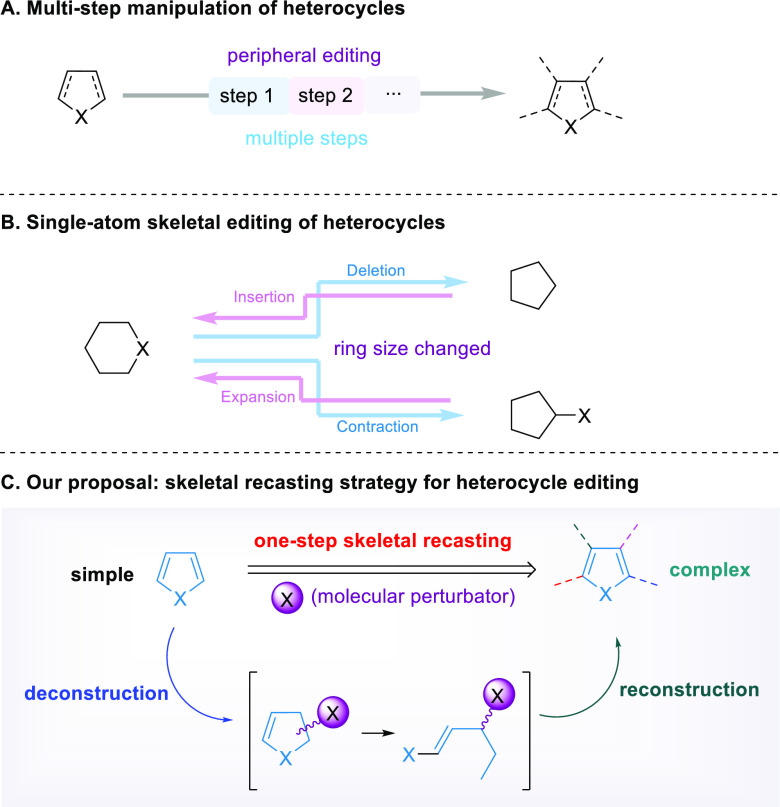
Background of heterocycle editing. (A) Multistep manipulation of heterocycles. (B) Single-atom skeletal editing of heterocycles. (C) Our proposal: skeletal recasting strategy for heterocycle editing.

Heterocyclic compounds are among the most significant structural scaffolds in medicinal chemistry and drug discovery,^1−4^ consequently, their selective functionalization is of crucial importance. The past two decades have witnessed remarkable progress in evolving C–H activation as a powerful strategy for functionalizing heterocycles, as well as their late-stage diversification.^5−11^ For structural editing of heterocyclic compounds at different peripheral sites, multistep synthetic manipulations are usually required (Figure 1A). In addition to peripheral editing, heterocycle editing through “single-atom skeletal editing” has recently emerged, which turned out to be a promising and fast-growing subfield in molecular editing (Figure 1B).^12−26^ In this tactic, the structures of heterocycles are synthetically “edited” through one-atom deletion or addition of the core molecular framework, to achieve desired transformations, thus offering powerful tools in drug discovery. In medicinal chemistry, maintaining the molecular core/skeleton of a lead compound would be ideal in the lead optimization process, which enables structural interrogation/modification in an efficient and productive manner. When the heterocyclic structures are concerned, the ability of maintaining the ring size while allowing synthetic manipulations to take place at various ring sites represents an ideal approach in molecular editing. Toward this end, we wondered if we could disrupt a simple heterocycle with a carefully chosen molecular perturbator, resulting in ring deconstruction to form an advanced intermediate, which will then incorporate the perturbator moieties and recast back to form the same type of heterocyclic ring with more structural complexity. We term this the skeletal recasting strategy (Figure 1C).

**Figure 2:**
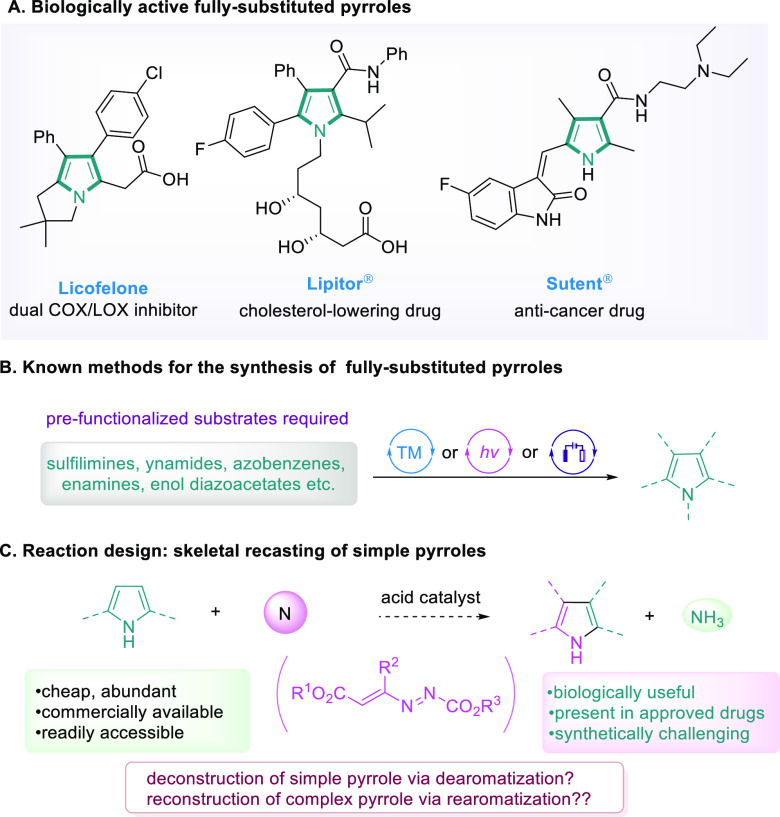
Reaction design. (A) Biologically active fully substituted pyrroles. (B) Known methods for the synthesis of fully substituted pyrroles. (C) Reaction design: skeletal recasting of simple pyrroles.

In our proof-of-concept study, we decided to focus on pyrrole and its derivatives. Pyrrole is one of the most prominent classes of five-membered nitrogen-containing heterocycles, the structure of which is widely present in natural products, drug molecules, catalysts, and advanced materials.^27−33^ In fact, pyrrole synthesis dates back to the 19th century, and the most well-known methods include the Knorr,^34−36^ Paal–Knorr,^37,38^ and Hantzsch^39^ reactions, which are still being commonly practiced nowadays. Our attention was drawn to fully substituted pyrrole derivatives, which are privileged scaffolds in numerous biologically active molecules, metabolites, and natural products, including a few best-selling pharmaceuticals (Figure 2A).^40−43^ On the other hand, synthesis of such structurally encumbered scaffolds represents a major synthetic challenge, presenting a bottleneck in the development of pyrrole-containing agrochemicals and pharmaceuticals. There are some reports on the synthesis of multisubstituted pyrroles;^44−53^ nevertheless, the reported methods often require specific prefunctionalized substrates, being somewhat less general (Figure 2B). Therefore, we became interested in devising an efficient approach to access fully substituted pyrroles, aiming to use our proposed skeletal recasting strategy. When the synthesis of complex pyrroles is concerned, simple pyrroles are arguably ideal starting materials, as they are cheap and readily accessible. The presence of a nitrogen atom makes multipositions of pyrrole nucleophilic, which in combination with the installation of different substituents on the pyrrole core will make synthetic manipulations more versatile. We reckon the key in our proposed skeletal recasting strategy is to introduce a suitable molecular perturbator, which will first trigger the pyrrole deconstruction via dearomatization and then reconstruct the pyrrole ring through rearomatization at a later stage. Consequently, we set the following criteria: (1) a dipole molecule that enables deconstruction/reconstruction sequence and (2) contains (at least) a nitrogen atom to facilitate reforming pyrrole ring. We reasoned that azoalkenes^54−59^ may serve as a suitable molecular perturbator. In a recent study, we showed that azoalkenes could serve as a valuable CCN 1,3-dipole due to a facile hydrazine-enamine tautomerization.^58^ In a projected reaction, we hypothesize that an acid-promoted nucleophilic attack of pyrrole on the azoalkene leads to dearomatization of the pyrrole ring. Subsequently, with the participation of azoalkene nitrogen, and an acid-promoted C–N bond cleavage, a rearomatization reaction may take place. Lastly, hydrolysis and elimination would yield a fully substituted pyrrole (Figure 2C). Herein, we introduce a new molecular editing strategy, termed skeletal recasting, for one-step facile synthesis of fully substituted pyrroles from simple pyrroles.

## Results and Discussion

To start our investigation, we chose 2,5-dimethyl-1H-pyrrole **1a** and azoalkene **2a** as model substrates and examined the potential skeletal recasting reaction (Table 1). To our delight, the projected reaction proceeded smoothly to yield recast pyrrole in the presence of acid catalysts (entries 1–5). Among all the acid catalysts examined, **Cat. 5** gave the best results. Catalyst is essential for the reaction, without which no reaction was observed (entry 6). Varying equivalence of azoalkene **2a** had no influence on the reaction (entries 7 and 8). A quick solvent screening revealed that chloroform was the solvent of choice (entries 9–13). When the catalyst loading was further lowered to 10 mol %, comparable results were obtained. Under the optimized reaction conditions, the recast tetra-substituted **3a** was obtained in 90% yield (entry 14).

**Table 1:**
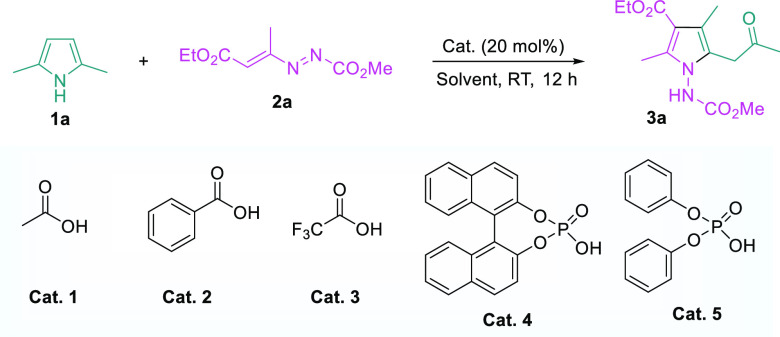
Optimization of Reaction Conditions^a^

entry	eq of **2a**	solvent	catalyst	yield (%)^b^
1	1.2	CH_2_Cl_2_	**Cat. 1**	22
2	1.2	CH_2_Cl_2_	**Cat. 2**	40
3	1.2	CH_2_Cl_2_	**Cat. 3**	20
4	1.2	CH_2_Cl_2_	**Cat. 4**	23
5	1.2	CH_2_Cl_2_	**Cat. 5**	49
6	1.2	CHCl_3_	–	–c
7	1.5	CH_2_Cl_2_	**Cat. 5**	44
8	2.0	CH_2_Cl_2_	**Cat. 5**	45
9	1.2	THF	**Cat. 5**	28
10	1.2	PhMe	**Cat. 5**	43
11	1.2	Et_2_O	**Cat. 5**	32
12	1.2	CH_3_CN	**Cat. 5**	45
13	1.2	CHCl_3_	**Cat. 5**	94
14^d^	1.2	CHCl_3_	**Cat. 5**	90
15^5^	1.2	CHCl_3_	**Cat. 5**	82

a Reaction conditions: **1a** (0.2 mmol), **2a** (0.24–0.4 mmol), catalyst (0.04 mmol), and solvent (2 mL).

b Isolated yield.

c No reaction was observed.

d 10 mol % of catalyst was used.

5 5 mol % of catalyst was used.

**Figure 3:**
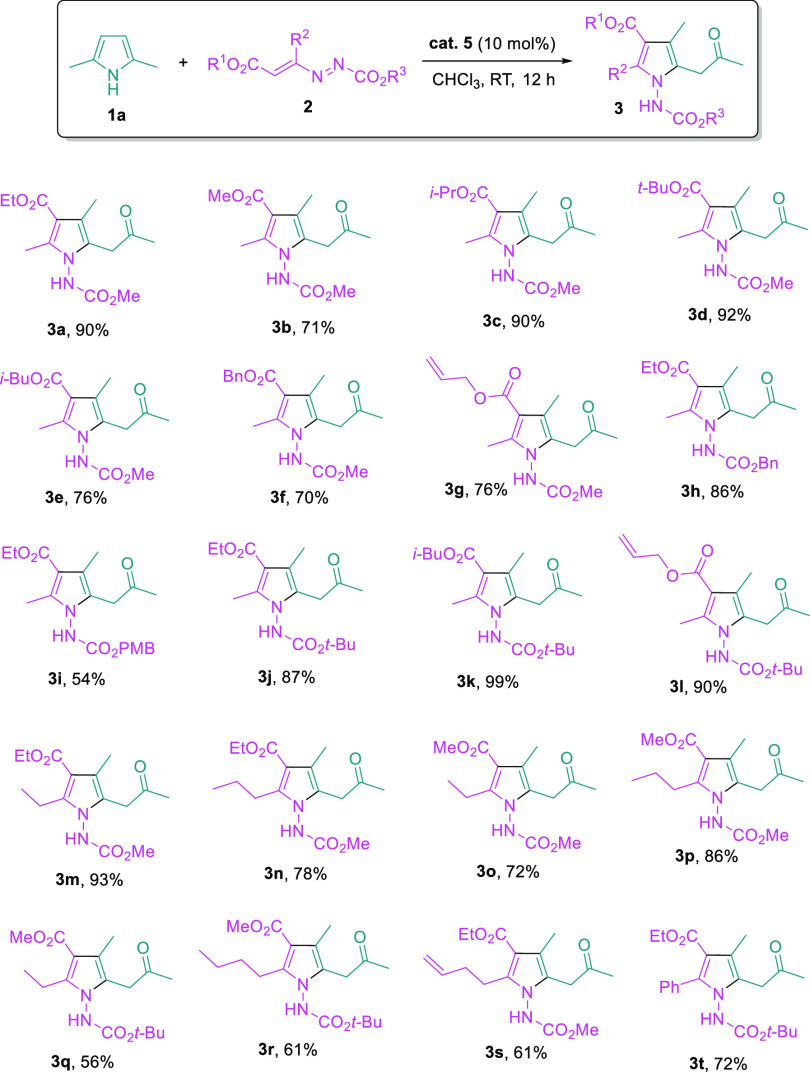
Reaction scope of azoalkene. Reaction conditions: **1a** (0.2 mmol), **2** (0.24 mmol), **Cat. 5** (10 mol %), and CHCl_3_ (2 mL).

With the optimized reaction conditions in hand, we explored the scope of azoalkene substrates (Figure 3). The tolerance of the reaction to the ester moieties appended to the C=C double bond of azoalkenes was first evaluated. A broad range of esters, such as methyl (**3b**), ethyl (**3a**), *i*-propyl (**3c**), *t*-butyl (**3d**), *i*-butyl (**3e**), benzyl (**3f**), and allyl (**3g**) esters, were all found to be suitable, and the tetra-substituted pyrroles were obtained in good yields. Subsequently, the ester groups at the azoalkene *N*-terminal were varied, and benzyl ester (**3h**), *p*-methoxybenzyl ester (**3i**), and *t*-butyl ester (**3j**) all worked well. Both ester moieties in the azoalkene structure can be changed at the same time, and the results remained excellent (**3k** and **3l**). In the reaction, the R^2^ group in azoalkene substrates ends up at the C5-position of pyrrole products, and modification of R^2^ offers great flexibility in accessing diverse 5-substituted pyrrole scaffolds. Indeed, the alkyl chain lengths could be varied from methyl, ethyl, *n*-propyl, to *n*-butyl, meanwhile, different esters could be installed at the two ester sites of the azoalkene structures, and decent yields were constantly obtained (**3m**–**3r**). Interestingly, when azoalkenes with an alkyl substituent bearing a terminal C=C bond or a phenyl substituent were employed, and the corresponding pyrroles (**3s** and **3t**) were obtained, such modifications not only add in great structural diversity to the pyrrole products but also make synthetic manipulations of the products more feasible.

**Figure 4:**
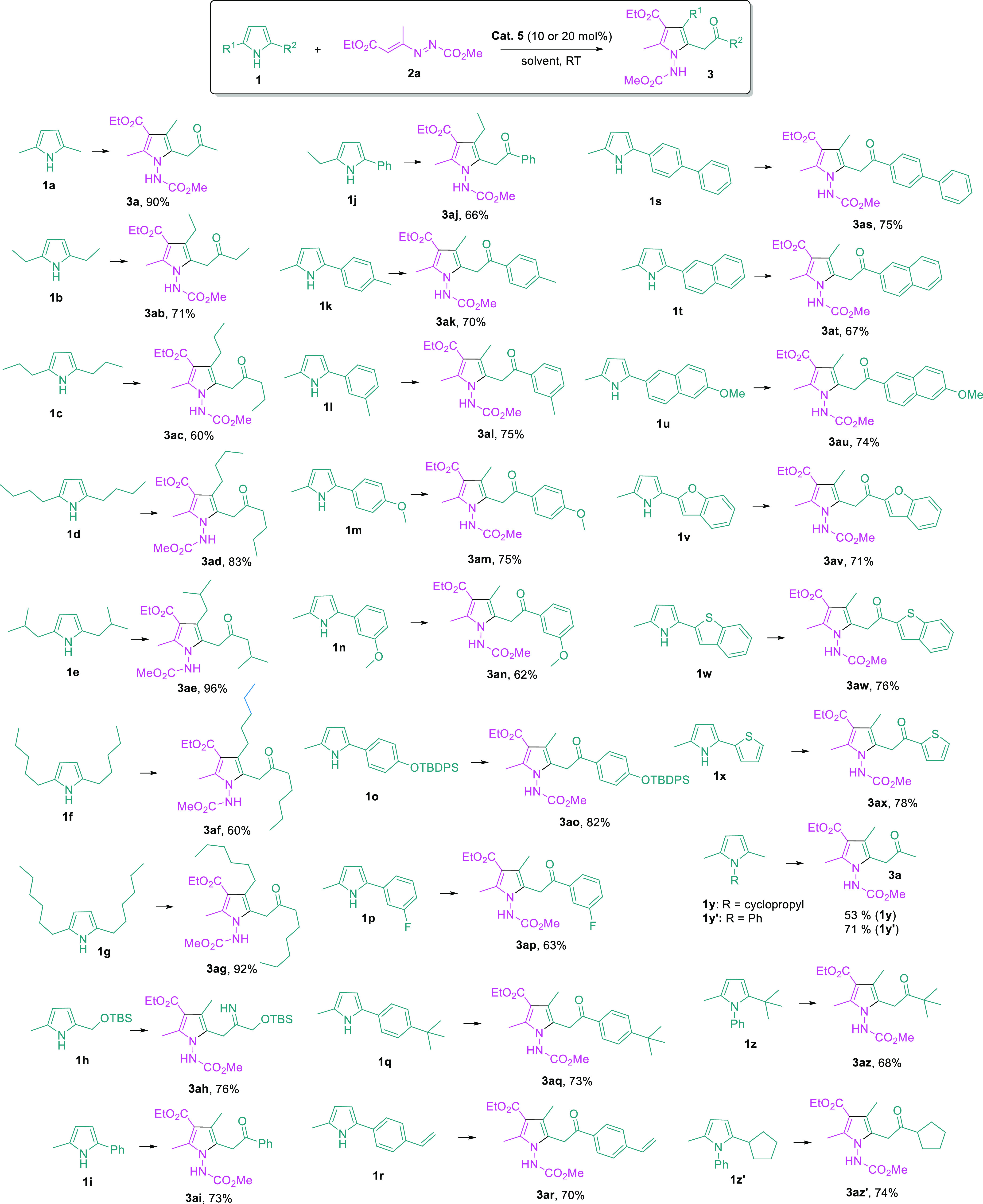
Reaction scope of pyrrole. Reaction conditions: **1** (0.2 mmol), **2a** (0.4 mmol), cat **5** (20 mol %), CH_2_Cl_2_ (2 mL), and 1 h; for substrates **1a**–**1h** and **1y**–**1z′**, the following conditions were used: **1** (0.2 mmol), **2a** (0.24 mmol), cat **5** (10 mol %), CHCl_3_ (2 mL), and 12 h.

Next, the applicability of this method to different pyrrole starting materials was evaluated (Figure 4). Our strategy starts with simple pyrrole substrates, which is highly practical, as these pyrroles are either commercially available or synthetically readily accessible through classic and robust reactions, e.g., Paal–Knorr synthesis. Symmetric pyrroles bearing various alkyl substituents at C2- and C5-positions are suitable substrates, and the corresponding 2,3-substituted pyrroles were obtained in moderate to excellent yields (**3a**–**3ag**). Moreover, unsymmetric pyrroles bearing different C2- and C5-substituents could also be employed. The utilization of pyrrole **1h** containing a siloxy group led to the formation of product containing an imine bond and untouched siloxy moiety (**3ah**), and it seems that unusual stability of imine is due to the formation of the intramolecular hydrogen-bonding network. When unsymmetric pyrroles bearing an alkyl and an aryl group were reacted with azoalkene **2a**, the recasting reaction took place smoothly to form desired products. Notably, the more sterically hindered aryl moieties in pyrrole substrates ended up at the 2-position of pyrrole products. The employment of aryl groups in pyrrole substrates is versatile, from simple phenyl to various substituted phenyls, regardless of the substitution pattern and electronic nature (**3ai**–**3aq**). In addition, styrene and biphenyl-containing pyrrole substrates were also found to be suitable (**3ar** and **3as**). Moreover, the reaction was applicable to pyrrole starting materials containing a (substituted)-naphthyl, benzofuran, benzothiophene, or thiophene, and the tetra-substituted pyrroles were constantly obtained in good yields (**3at**–**3ax**). Interestingly, replacement of the hydrogen atom of pyrrole NH moiety with a cyclopropyl group (**1y**) or phenyl (**1y′**) group had little influence, and the same pyrrole product **3a** was obtained. At last, when unsymmetric *N*-phenyl pyrroles bearing two different C2- and C5-alkyl substituents were utilized, the corresponding pyrrole products were obtained in good yields; it is notable that highly sterically hindered *t*-butyl could be employed (**3az**).

**Figure 5:**
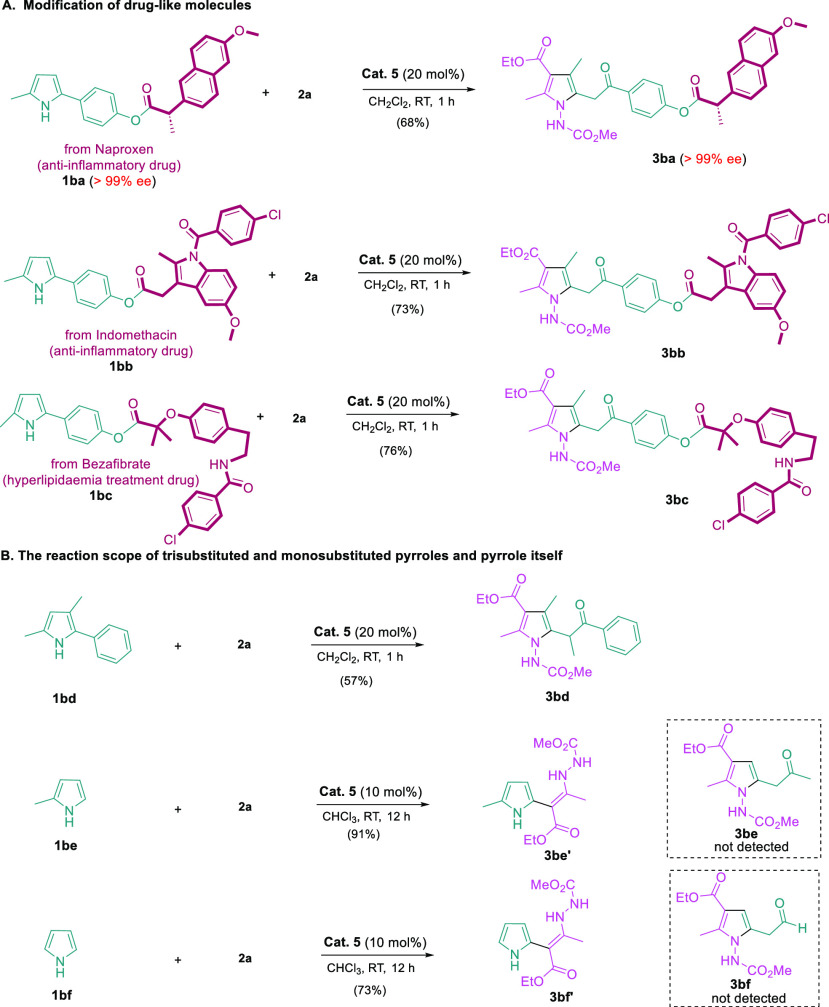
Further reaction scope.

To further investigate the reaction scope, we turned our attention to the application of this methodology to biologically active molecules to demonstrate the potential of our method for the modification of drug-like molecules (Figure 5A). 2,5-Disubstituted pyrroles derived from naproxen (anti-inflammatory), indomethacin (anti-inflammatory), and bezafibrate (hyperlipidaemia treatment) were subjected to the standard reaction conditions, skeletal recasting took place smoothly without touching ester and amide groups, and the corresponding tetra-substituted pyrrole derivatives (**3ba**–**3bc**) were formed in good yields. It is noteworthy that enantiomeric excess of **3ba** remained >99% after the skeletal recasting process, indicating no enantiomeric erosion under our reaction conditions. Furthermore, the reaction scope with regard to trisubstituted and monosubstituted pyrroles, as well as pyrrole itself, was also examined. The utilization of trisubstituted pyrrole formed the desired recast product in good yield (**3bd**), while the reaction with monosubstituted pyrrole or pyrrole only led to the formation of a 1,4-addition product (**3be′** and **3bf′**), not the recast products (Figure 5B).

**Figure 6:**
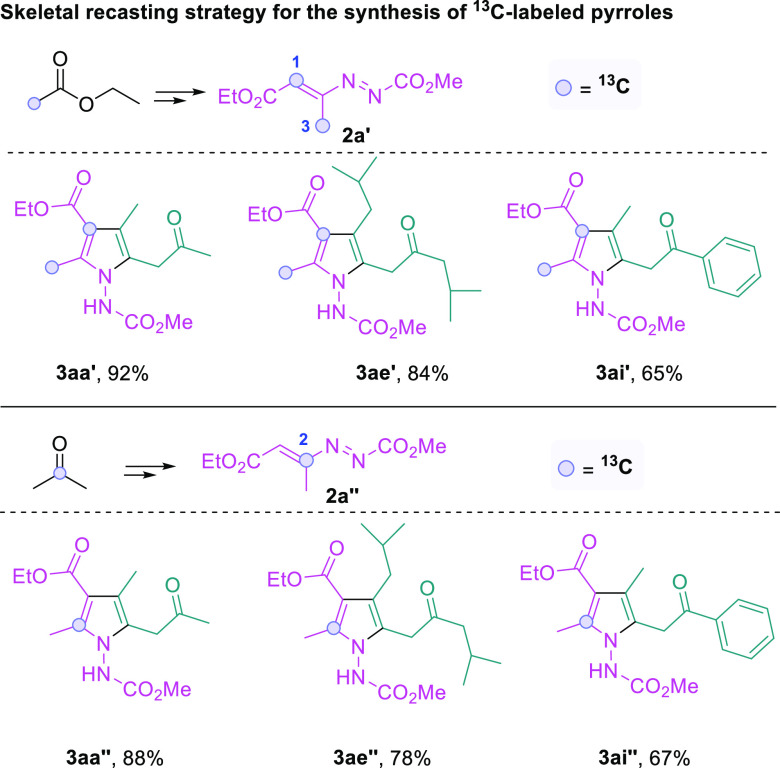
Skeletal recasting strategy for the synthesis of ^13^C-labeled pyrroles.

Compounds with ^13^C labeling have broad applications in organic chemistry, medicinal chemistry, and life sciences; consequently, catalytic methods enabling site-selective ^13^C labeling are of urgent need.^60−63^ Notably, ^13^C-labeled fully substituted pyrroles could be readily synthesized from ^13^C-labeled azoalkenes through skeletal recasting (Figure 6). Specifically, the 1,3-^13^C-labeled azoalkene **2a′** was easily prepared from 2-^13^C-labeled ethyl acetate in excellent yield, which was then subjected to the reaction with different pyrrole starting materials. The projected recasting reaction proceeded smoothly, resulting in the formation of ^13^C-labeled fully substituted pyrroles in good to excellent yields (**3aa′**, **3ae′**, and **3ai′**). Similarly, the reaction of 2-^13^C-labeled azoalkene **2a″** with simple pyrroles formed site-specific ^13^C-labeled fully substituted pyrroles in good to excellent yields (**3aa″**, **3ae″**, and **3ai″**).

**Figure 7:**
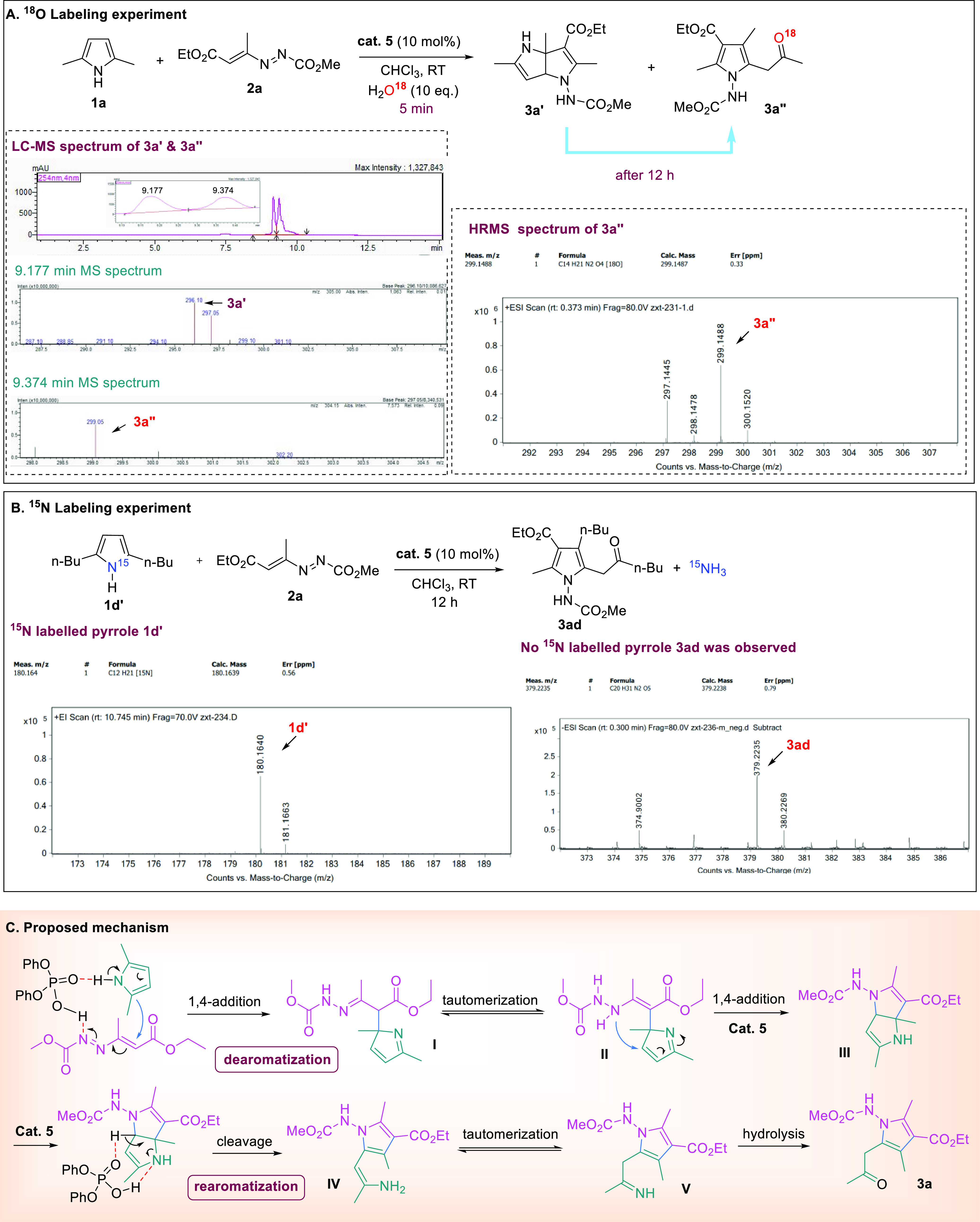
Mechanistic studies. (A) ^18^O-labeling experiment. (B) ^15^N-labeling experiment. (C) Proposed mechanism.

A series of experiments were conducted to shed light on the mechanism of this skeletal recasting reaction of simple pyrroles with azoalkenes (Figure 7). In our hypothesis, the deconstruction of a simple pyrrole substrate entails a dearomatization process to form an advanced intermediate and the detection of which would provide strong evidence to our mechanistic proposal. Accordingly, we mixed pyrrole **1a** and azoalkene **2a** in anhydrous chloroform with the introduction of 10 molar equivalence of O^18^-labeled H_2_O and monitored the reaction progress with liquid chromatography–mass spectrometry (LC–MS). Within 5 min, two peaks with retention time of 9.177 and 9.374 min were observed, corresponding to advanced intermediate **3a′** (MS = 296.10 observed) and the final pyrrole product **3a″** (MS = 299.05 observed). After the overnight reaction, intermediate **3a′** disappeared and only **3a″** was observed, and high-resolution mass spectrometry (HRMS) of the latter was taken, confirming its presence ambiguously (Figure 7A). Another key point to be clarified in the mechanism is the fate of the pyrrole nitrogen atom. The fact that the newly recast pyrrole contains a hydrazine moiety clearly suggests the incorporation of azoalkene into the product and the departure of the nitrogen atom from the pyrrole starting material. Consequently, we performed the reaction using ^15^N-labeled pyrrole (**1d′**) as the starting material. Indeed, the recast pyrrole product (**3ad**) did not contain a radio-labeled nitrogen (Figure 7B). With the above mechanistic studies, a plausible mechanism for the reaction is proposed (Figure 7C). Phosphoric acid promotes a Friedel–Crafts-type 1,4-addition of pyrrole to azoalkene, forming a dearomatized hydrazine intermediate (**I**). Subsequently, a tautomerization to enamine takes place, yielding intermediate **II**. Under the catalysis of phosphoric acid catalyst, another 1,4-addition occurs to provide the bicyclic intermediate **III**, which undergoes a crucial rearomatization, through the cleavage of the C–N bond at the original pyrrole nitrogen site, restoring aromaticity and furnishing a new pyrrole ring (**V**). Finally, an enamine-imine tautomerization, followed by a hydrolysis, leads to the formation of final product **3a**.

The tetra-substituted pyrroles prepared using the skeletal recasting strategy are useful and interesting. The presence of the N–N bond in the pyrrole structure offers an opportunity to create N–N axial chirality.^64−69^ As an illustration, we carried out the asymmetric *N*-alkylation reaction of pyrrole **3t**, using the Morita–Baylis–Hillman (MBH) carbonate **5a** as the alkylating agent (Table 2). Cinchona alkaloids turned out to be good catalysts, promoting the reaction in an enantioselective manner (entries 1–3). Among all the alkaloids examined, quinidine was found to be the best, forming the desired alkylation product **6** in 70% yield with 70% ee. Subsequently, a quick solvent screening showed that dichloromethane was the most suitable solvent (entries 4–6). Lowering the reaction temperature further enhanced the enantioselectivity of the reaction. When the reaction was performed in dichloromethane at −20 °C, the desired product **6** bearing an N–N axial chirality was obtained in 80% yield and with 91% ee (entry 9).

**Table 2:**
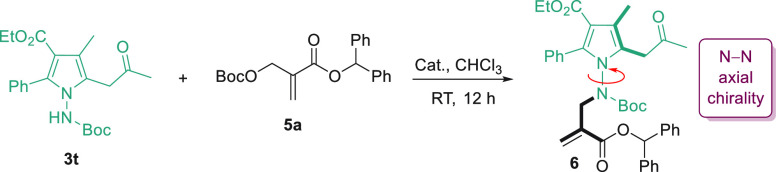
Asymmetric Organocatalytic Synthesis of N–N Axially Chiral Molecules^a^

entry	cat.	solvent	temp (°C)	yield (%)^b^	ee (%)^c^
1	quinine	CHCl_3_	rt	30	–49
2	cinchonidine	CHCl_3_	rt	80	–55
3	quinidine	CHCl_3_	rt	70	70
4	quinidine	toluene	rt	85	54
5	quinidine	DCE	rt	87	75
6	quinidine	CH_2_Cl_2_	rt	90	78
7	quinidine	CH_2_Cl_2_	0	90	81
8	quinidine	CH_2_Cl_2_	–10	90	85
9	quinidine	CH_2_Cl_2_	–20	80	91

a Reaction conditions: **3o** (0.1 mmol), **2a** (0.16 mmol), catalyst (10 mol %), and solvent (2 mL).

b Isolated yield.

c Determined by HPLC analysis on a chiral-stationary-phase. ee, enantiomeric excess; DCE, 1,2-dichloroethane.

**Figure 8:**
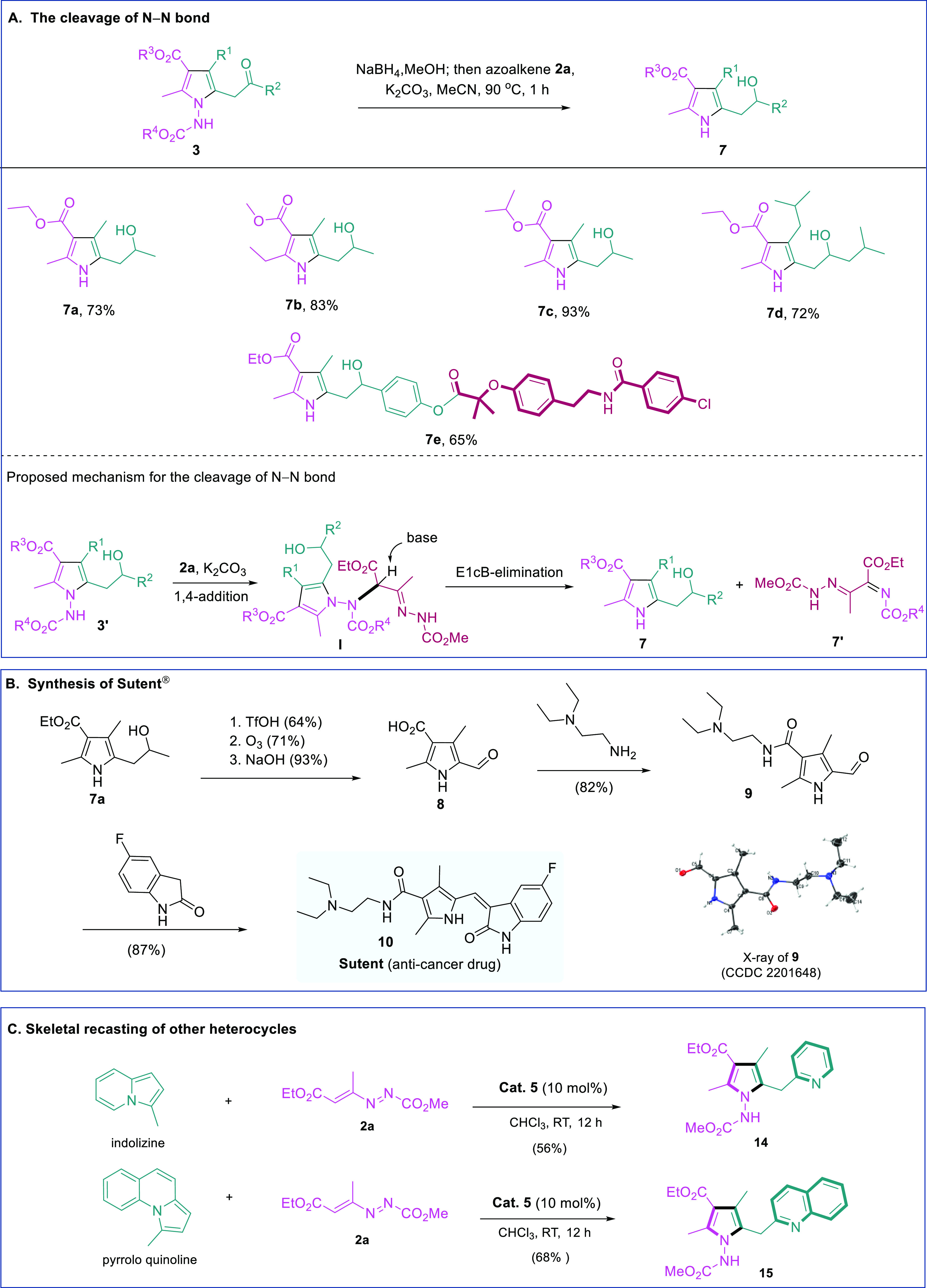
Synthetic applications. (A) Cleavage of N–N bond. (B) Synthesis of Sutent. (C) Skeletal recasting of other heterocycles.

We next proceeded to perform the N–N bond cleavage and form the *N*-unprotected pyrrole products.^70^ When *N*-protected pyrrole products **3** were treated with azoalkene **2a**, the N–N bond was cleaved, and the corresponding *N*-unprotected pyrroles **7** were obtained in high yields (**7a**–**7d**). It is noteworthy that the modified drug-like **3bc** well tolerated the deprotection conditions, and the complex *N*-unprotected pyrrole **7e** was obtained in good yield. The proposed mechanism of N–N bond cleavage is also illustrated. The reaction commences with a nucleophilic attack by the exocyclic nitrogen of pyrroles **3′** on the electrophilic carbon of azoalkene **2a**, leading to the formation of intermediate **I**, which undergoes a N–N bond cleavage through an E1cB process to afford *N*-unprotected pyrrole **7** (Figure 8A).

To highlight the synthetic value of our tetra-substituted pyrrole products, we conducted a concise synthesis of Sutent, one of the best-selling anticancer drugs.^40^ As depicted in Figure 8B, the *N*-unprotected pyrrole **7a** was subjected to a few trivial reactions, yielding aldehyde **8**. At last, a simple amination, followed by a condensation, completed the synthesis of Sutent (**10**).

From a conceptual viewpoint, the skeletal recasting strategy we introduced herein for heterocycle editing should be generally applicable to other heterocyclic structures, provided these heterocycles may: (1) chemically interact with a judiciously selected/designed molecular perturbator (azoalkene in current study) and (2) be capable of incorporating extra structural moieties from perturbator to form a more complex heterocyclic structure. Indeed, when indolizine was treated with azoalkene **2a** in the presence of phosphoric acid, a novel heterocycle **14** containing both pyrrole and pyridine moieties was formed. Similarly, when pyrrolo quinoline was subjected to our standard reaction conditions, deconstruction and reconstruction processes happened, and the recast product **15** bearing both pyrrole and quinoline substructures was obtained in good yield (Figure 8C).

## Conclusions

In summary, we have developed an efficient synthesis of fully substituted pyrroles from simple pyrroles, enabled by the skeletal recasting strategy. By introducing an azoalkene as a molecular perturbator, a dearomatization of pyrrole starting materials takes place, which is followed by a rearomatization process incorporating structural moieties of the perturbator to form more complex pyrrole motifs. A broad range of tetra-substituted pyrroles are conveniently prepared, and we also introduce N–N axial chirality to the products, as well as to complete a facile synthesis of the anticancer drug, Sutent. Conceptually, the skeletal recasting strategy has broad applicability to other heterocycles, thus may offer a powerful tool for molecular editing of heterocyclic structures. Currently, we are working toward extending this concept to heterocycle editing in a broader context, targeting the synthesis of complex heterocycles. Such strategies should find wide applications in medicinal chemistry, argochemistry, and materials sciences, and we anticipate that more exciting discoveries in the science of molecular editing are forthcoming.

## Supporting Information Available

Relevant data include reaction optimization, reaction procedure, product characterization, and NMR spectra (PDF)
X-ray structure of compound **9** (CIF)

## Supplementary Material

oc3c00812_si_001.pdf

oc3c00812_si_002.cif
