# Supplementary material for: Molecular Editing of Pyrroles via a Skeletal Recasting Strategy
Source: ACS Cent Sci. 2023 Aug 15;9(9):1758–67. doi: 10.1021/acscentsci.3c00812 (PMC10540293; doi:10.1021/acscentsci.3c00812)
Supplement: Supplementary file 1 — oc3c00812_si_001.pdf [file oc3c00812_si_001.pdf]

Supporting Information

Molecular Editing of Pyrroles via a Skeletal Recasting Strategy

Xueting Zhou[†,‡], Qingqin Huang[†,‡], Jiami Guo[†,‡], Lei Dai*[,‡], and Yixin Lu*[,†,‡]

[†]Joint School of National University of Singapore and Tianjin University, International Campus of Tianjin University, Binhai New City, Fuzhou, Fujian, 350207, China

[‡]Department of Chemistry, National University of Singapore, 3 Science Drive 3, Singapore, 117543, Singapore

*Corresponding authors: L. Dai, Email: chmdail@nus.edu.sg ;Y. Lu, Email: chmlyx@nus.edu.sg

Contents

1. Materials and methods S3

2. General procedures for the synthesis of fully substituted pyrroles S3

3. Characterization data of the products S4

4. Synthesis of ^{13}C-labeled fully-substituted pyrroles S27

5. Mechanistic studies S32

6. Synthetic applications. S35

7. Single Crystal Structure X-ray Analysis of **9** S44

8. NMR Spectra S76

9. References S148

1. Materials and methods

All starting materials were obtained from commercial suppliers (Sigma Aldrich, TCI and BLD Pharm) and directly used without further purification unless otherwise stated. All reactions were carried out under argon atmosphere with magnetic stirring unless otherwise stated. Azoalkenes[1-4] and pyrroles [5-9] were synthesized according to literatures. Diphenyl phosphate (CAS: 838-85-7) was purchased from BLD Pharmatech Ltd.

Analytical thin layer chromatography was carried out with silica gel pre-coated glass plates (TLC-Silica gel GF254, coating thickness: 0.25 mm) purchased from Merck. Visualization was accomplished with short wave UV light (254nm) and/or 10% phosphomolybdic acid in ethanol or $KMnO_4$ staining solutions followed by heating. Column chromatography was performed on silica gel 200~300 mesh. ^1H NMR and ^{13}C NMR spectra were recorded on a Bruker AV-III400 (400 MHZ) or AMX500 (500 MHz) spectrometer. Chemical shifts were calibrated using residual solvent as an internal reference ($CDCl_3$: 7.26 ppm ^1H NMR, 77.00 ppm ^{13}C NMR). ^1H NMR spectroscopy splitting patterns were designated as singlet (s), doublet (d), triplet (t), quartet (q). Splitting patterns that could not be interpreted or easily visualized were designated as multiplet (m). All high-resolution mass spectra (HRMS) were obtained on a Finnigan/MAT 95XL-T spectrometer, the calculated values are based on the most abundant isotope. Chiral HPLC analyses were performed on an Agilent 1100 Series using a Daicel Chiralpak column (AD-H) with hexanes/*i*-PrOH as the eluent.

2. General procedures for the synthesis of fully substituted pyrroles

1） R^1, R^2 = alkyl group (GP-1)

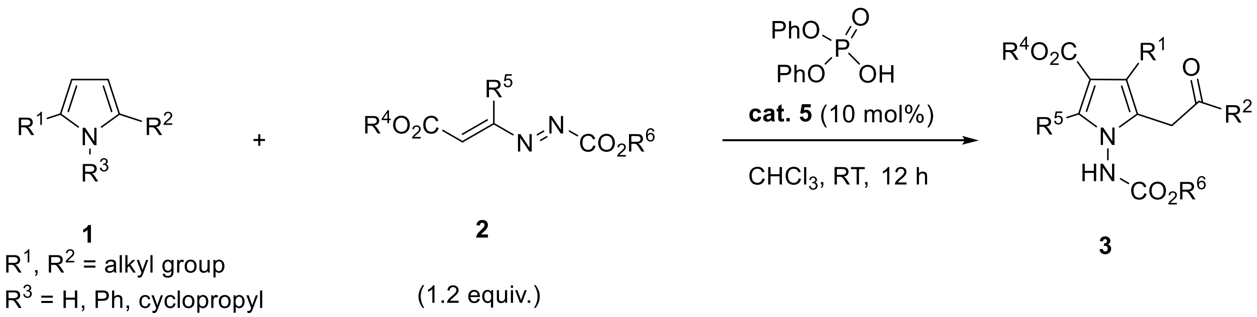

To an 8 mL screw-cap vial equipped with a magnetic stir bar was charged with simple pyrrole **1** (0.2 mmol), azoalkene **2** (0.24 mmol) and cat. **5** (10 mol%) in $CHCl_3$ (2 mL) at room temperature.

The reaction mixture was concentrated under reduced pressure after 12 h and the residue was purified by column chromatography on silica gel to furnish the product.

2）R^1 = alkyl group, R^2 = aryl group (GP-2)

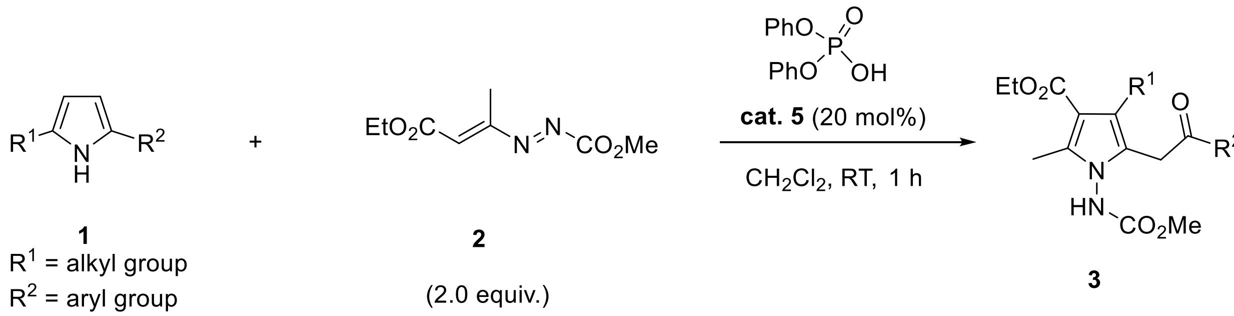

To an 8 mL screw-cap vial equipped with a magnetic stir bar was charged with simple pyrrole **1** (0.2 mmol), azoalkene **2** (0.4 mmol) and cat. **5** (20 mol%) in CH_2Cl_2 (2 mL) at room temperature. The reaction mixture was concentrated under reduced pressure after 1 h and the residue was purified by column chromatography on silica gel to furnish the product.

3. Characterization data of the products

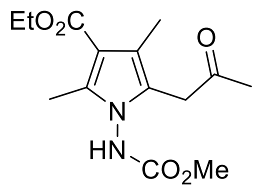

3a

Ethyl 1-((methoxycarbonyl)amino)-2,4-dimethyl-5-(2-oxopropyl)-1H-pyrrole-3-carboxylate (3a)

(GP-1) A light-yellow oil; isolated yield = 90%. ^{1}H NMR (500 MHz, Acetonitrile-*d*3) δ 8.34 (s, 1H), 4.24 (q, *J* = 7.1 Hz, 2H), 3.75 (s, 3H), 3.64 (d, *J* = 17.3 Hz, 1H), 3.51 (d, *J* = 17.3 Hz, 1H), 2.34 (s, 3H), 2.16 (s, 3H), 2.08 (s, 3H), 1.33 (t, *J* = 7.1 Hz, 3H). ^{13}C NMR (126 MHz, Acetonitrile-*d*3) δ 205.0, 165.3, 155.7, 136.3, 123.6, 116.0, 59.1, 52.9, 38.1, 28.2, 13.8, 10.5, 10.0. HRMS (ESI) m/z calcd for $C_{14}H_{21}N_2O_5$ $[M+H]^+$ = 297.1445, found = 297.1445.

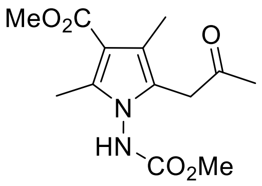

3b

Methyl 1-((methoxycarbonyl)amino)-2,4-dimethyl-5-(2-oxopropyl)-1H-pyrrole-3-carboxylate (3b)

(GP-1) A light-yellow oil; isolated yield = 71%. ^{1}H NMR (400 MHz, $CDCl_3$) δ 7.82 (s, 1H), 3.77 (s, 6H), 3.68 (d, *J* = 17.1 Hz, 1H), 3.40 (d, *J* = 17.1 Hz, 1H), 2.36 (s, 3H), 2.15 (d, *J* = 1.7 Hz, 6H). ^{13}C NMR (101 MHz, $CDCl_3$) δ 166.2, 155.6, 137.3, 122.0, 116.6, 109.6, 53.4, 50.5, 38.5, 11.3, 10.8. HRMS (ESI) m/z calcd for $C_{13}H_{19}N_2O_5$ $[M+H]^+$ = 283.1288, found = 283.1290.

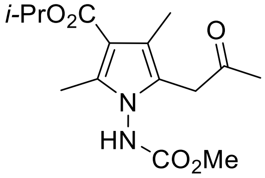

3c

Isopropyl 1-((methoxycarbonyl)amino)-2,4-dimethyl-5-(2-oxopropyl)-1H-pyrrole-3-carboxylate (3c)

(GP-1) A light-yellow oil; isolated yield = 90%. ^{1}H NMR (400 MHz, $CDCl_3$) δ 7.60 (s, 1H), 5.15 (p, *J* = 6.3 Hz, 1H), 3.78 (s, 3H), 3.72 (d, *J* = 17.1 Hz, 1H), 3.40 (d, *J* = 17.1 Hz, 1H), 2.39 (s, 3H), 2.18 (s, 3H), 2.18 (s, 3H), 1.31 (s, 3H), 1.30 (s, 3H). ^{13}C NMR (101 MHz, $CDCl_3$) δ 206.5, 165.2, 155.6, 137.03, 121.8, 116.7, 110.2, 66.5, 53.5, 38.5, 29.4, 22.2, 11.5, 10.9. HRMS (ESI) m/z calcd for $C_{15}H_{23}N_2O_5$ $[M+H]^+$ =311.1601, found = 311.1604.

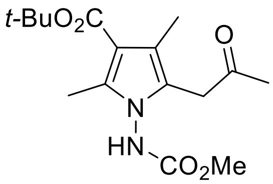

3d

***Tert*-butyl 1-((methoxycarbonyl)amino)-2,4-dimethyl-5-(2-oxopropyl)-1H-pyrrole-3-carboxylate (3d)**

(GP-1) A light-yellow oil; isolated yield = 92%. ^{1}H NMR (400 MHz, $CDCl_3$) δ 7.77 (s, 1H), 3.76 (s, 3H), 3.68 (d, *J* = 17.1 Hz, 1H), 3.40 (d, *J* = 17.1 Hz, 1H), 2.35 (s, 3H), 2.15 (s, 3H), 2.15 (s, 3H), 1.53 (s, 9H). ^{13}C NMR (101 MHz, $CDCl_3$) δ 206.6, 165.0, 155.7, 136.5, 121.7, 116.5, 111.1, 79.6, 53.4, 38.5, 29.3, 28.5, 11.5, 10.8. HRMS (ESI) m/z calcd for $C_{16}H_{24}N_2NaO_5$ $[M+Na]^+$ = 347.1577, found = 347.1570.

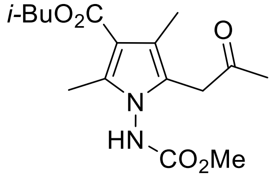

3e

Iso-butyl 1-((methoxycarbonyl)amino)-2,4-dimethyl-5-(2-oxopropyl)-1H-pyrrole-3-carboxylate (3e)

(GP-1) A light-yellow oil; isolated yield = 76%. ^{1}H NMR (400 MHz, $CDCl_3$) δ 7.62 (s, 1H), 3.99 (d, *J* = 6.4 Hz, 2H), 3.78 (s, 3H), 3.72 (d, *J* = 17.2 Hz, 1H), 3.41 (d, *J* = 17.1 Hz, 1H), 2.40 (s, 3H), 2.19 (s, 3H), 2.18 (s, 3H), 2.01 (dt, *J* = 13.3, 6.6 Hz, 1H), 1.00 (s, 3H), 0.98 (s, 3H). ^{13}C NMR (101 MHz, $CDCl_3$) δ 206.5, 165.8, 155.6, 137.3, 121.9, 116.7, 109.9, 69.9, 53.5, 38.5, 29.4, 27.8, 19.4, 11.6, 10.9. HRMS (ESI) m/z calcd for $C_{16}H_{24}N_2NaO_5$ $[M+Na]^+$ = 347.1577, found =347.1571.

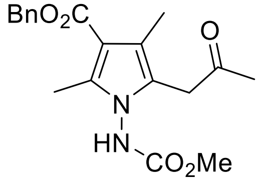

3f

Benzyl 1-((methoxycarbonyl)amino)-2,4-dimethyl-5-(2-oxopropyl)-1H-pyrrole-3-carboxylate (3f)

(GP-1) A light-yellow oil; isolated yield = 70%. ^{1}H NMR (400 MHz, $CDCl_3$) δ 7.69 (s, 1H), 7.43 – 7.27 (m, 5H), 5.26 (s, 2H), 3.77 (s, 3H), 3.71 (d, *J* = 17.2 Hz, 1H), 3.40 (d, *J* = 17.2 Hz, 1H), 2.39 (s, 3H), 2.18 (s, 3H), 2.17 (s, 3H). ^{13}C NMR (101 MHz, $CDCl_3$) δ 206.7, 165.4, 155.6, 136.7, 128.4, 128.0, 127.8, 122.0, 116.7, 109.4, 65.3, 53.5, 38.5, 29.4, 11.5, 11.0 HRMS (ESI) m/z calcd for $C_{19}H_{23}N_2O_5$ $[M+H]^+$ = 359.1601, found = 359.1600.

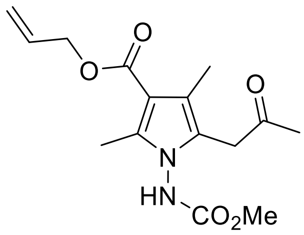

3g

Allyl 1-((methoxycarbonyl)amino)-2,4-dimethyl-5-(2-oxopropyl)-1H-pyrrole-3-carboxylate (3g)

(GP-1) A light-yellow oil; isolated yield = 76%. ^{1}H NMR (400 MHz, $CDCl_3$) δ 7.85 (s, 1H), 5.99 (ddt, *J* = 17.1, 10.4, 5.5 Hz, 1H), 5.42 – 5.16 (m, 2H), 4.70 (dt, *J* = 5.6, 1.5 Hz, 2H), 3.76 (s, 3H), 3.69 (d, *J* = 17.1 Hz, 1H), 3.41 (d, *J* = 17.1 Hz, 1H), 2.38 (s, 3H), 2.17 (s, 3H), 2.15 (s, 3H). ^{13}C NMR (101 MHz, $CDCl_3$) δ 206.5, 165.3, 155.6, 137.4, 132.9, 122.1, 117.4, 116.7, 109.5, 64.1, 53.4, 38.5, 29.3, 11.4, 10.9. HRMS (ESI) m/z calcd for $C_{15}H_{21}N_2O_5$ $[M+H]^+$ = 309.1445, found = 309.1442.

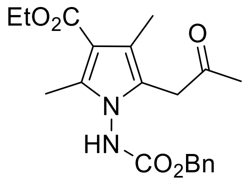

3h

Ethyl 1-(((benzyloxy)carbonyl)amino)-2,4-dimethyl-5-(2-oxopropyl)-1H-pyrrole-3-carboxylate (3h)

(GP-1) A light-yellow oil; isolated yield = 86%. ^{1}H NMR (400 MHz, $CDCl_3$) δ 7.63 (s, 1H), 7.36 (s, 5H), 5.19 (s, 2H), 4.25 (q, *J* = 7.1 Hz, 2H), 3.68 (d, *J* = 17.2 Hz, 1H), 3.31 (d, *J* = 17.3 Hz, 1H), 2.36 (s, 3H), 2.17 (s, 3H), 2.11 (s, 3H), 1.32 (t, *J* = 7.1 Hz, 4H). ^{13}C NMR (101 MHz, $CDCl_3$) δ 206.4, 165.7, 155.0, 137.2, 135.3, 128.6, 128.4, 121.8, 116.7, 109.8, 68.2, 59.3, 38.4, 29.4, 14.4, 11.4, 10.9. HRMS (ESI) m/z calcd for $C_{20}H_{25}N_2O_5$ $[M+H]^+$ = 373.1758, found = 373.1763

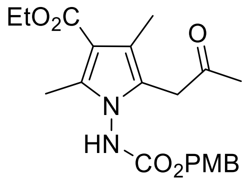

3i

Ethyl 1-((((4-methoxybenzyl)oxy)carbonyl)amino)-2,4-dimethyl-5-(2-oxopropyl)-1H-pyrrole-3-carboxylate (3i)

(GP-1) A light-yellow oil; isolated yield = 54%. ^{1}H NMR (400 MHz, $CDCl_3$) δ 7.79 (s, 1H), 7.28 (s, 2H), 6.87 (d, *J* = 8.5 Hz, 2H), 5.11 (s, 2H), 4.23 (q, *J* = 7.1 Hz, 2H), 3.80 (s, 3H), 3.63 (d, *J* = 17.7 Hz, 1H), 3.31 (d, *J* = 17.3 Hz, 1H), 2.33 (s, 3H), 2.15 (s, 3H), 2.08 (s, 3H), 1.31 (t, *J* = 7.1 Hz, 3H). ^{13}C NMR (101 MHz, $CDCl_3$) δ 206.5, 165.7, 159.8, 155.2, 137.1, 130.3, 127.3, 122.0, 116.6, 113.9, 109.7, 68.0, 59.2, 55.2, 38.4, 29.2, 14.4, 11.4, 10.8. HRMS (ESI) m/z calcd for $C_{21}H_{27}N_2O_5$ $[M+H]^+$= 403.1864, found = 403.1864.

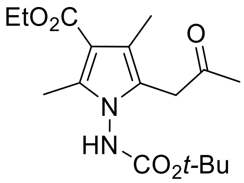

3j

Ethyl 1-((tert-butoxycarbonyl)amino)-2,4-dimethyl-5-(2-oxopropyl)-1H-pyrrole-3-carboxylate (3j)

(GP-1) A light-yellow oil; isolated yield = 87%. ^{1}H NMR (400 MHz, $CDCl_3$) δ 7.60 (s, 1H), 4.21 (q, *J* = 7.3 Hz, 2H), 3.63 (d, *J* = 17.0 Hz, 1H), 3.38 (d, *J* = 17.0 Hz, 1H), 2.35 (s, 3H), 2.15 (s, 3H), 2.12 (s, 3H), 1.45 (s, 9H), 1.30 (t, *J* = 6.6 Hz, 3H). ^{13}C NMR (101 MHz, $CDCl_3$) δ 206.4, 165.8, 154.1, 137.2, 122.2, 116.4, 109.5, 82.4, 59.2, 38.7, 29.1, 28.0, 14.4, 11.3, 10.8. HRMS (ESI) m/z calcd for $C_{17}H_{27}N_2O_5$ $[M+H]^+$ = 339.1914, found = 339.1904.

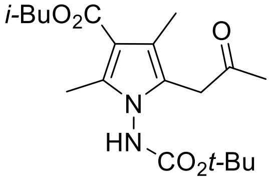

3k

***Iso*-butyl 1-((tert-butoxycarbonyl)amino)-2,4-dimethyl-5-(2-oxopropyl)-1H-pyrrole-3-carboxylate (3k)**

(GP-1) A light-yellow oil; isolated yield = 99%. ^{1}H NMR (400 MHz, $CDCl_3$) δ 7.41 (s, 1H), 3.97 (d, *J* = 6.4 Hz, 2H), 3.67 (d, *J* = 17.0 Hz, 1H), 3.39 (d, *J* = 17.0 Hz, 1H), 2.39 (s, 3H), 2.18 (s, 3H), 2.15 (s, 3H), 2.01 (dtd, *J* = 14.1, 7.0, 5.7 Hz, 1H), 1.46 (s, 9H), 0.98 (s, 3H), 0.97 (s, 3H). ^{13}C NMR

(101 MHz, $CDCl_3$) δ 206.4, 165.9, 154.1, 137.3, 122.1, 116.5, 109.6, 82.5, 69.8, 38.7, 29.2, 28.0, 27.8, 19.4, 11.6, 10.9. HRMS (ESI) m/z calcd for $C_{19}H_{31}N_2O_5$ $[M+H]^+$ = 367.2227, found = 367.2227.

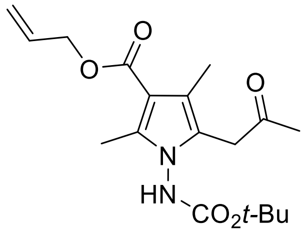

3l

Allyl 1-((tert-butoxycarbonyl)amino)-2,4-dimethyl-5-(2-oxopropyl)-1H-pyrrole-3-carboxylate (3l)

(GP-1) A light-yellow oil; isolated yield = 90%. ^{1}H NMR (400 MHz, $CDCl_3$) δ 7.47 (s, 1H), 6.08 – 5.87 (m, 1H), 5.34 (d, J = 17.1 Hz, 1H), 5.21 (d, J = 10.5 Hz, 1H), 4.70 (d, J = 4.0 Hz, 2H), 3.67 (d, J = 17.9 Hz, 1H), 3.39 (d, J = 17.0 Hz, 1H), 2.38 (s, 3H), 2.17 (s, 3H), 2.14 (s, 3H), 1.46 (s, 9H). ^{13}C NMR (101 MHz, $CDCl_3$) δ 206.3, 165.4, 154.0, 137.5, 133.0, 122.2, 117.3, 116.5, 109.3, 82.5, 64.1, 38.6, 29.2, 28.0, 11.5, 10.9. HRMS (ESI) m/z calcd for $C_{18}H_{27}N_2O_5$ $[M+H]^+$ = 351.1914, found = 351.1913.

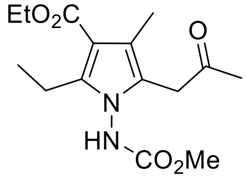

3m

Ethyl 2-ethyl-1-((methoxycarbonyl)amino)-4-methyl-5-(2-oxopropyl)-1H-pyrrole-3-carboxylate (3m)

(GP-1) A light-yellow oil; isolated yield = 93%. ^{1}H NMR (400 MHz, $CDCl_3$) δ 8.07 (s, 1H), 4.22 (q, J = 7.1 Hz, 2H), 3.74 (s, 3H), 3.64 (d, J = 17.1 Hz, 1H), 3.38 (d, J = 17.1 Hz, 1H), 2.97 – 2.64 (m, 2H), 2.15 (s, 3H), 2.12 (s, 3H), 1.30 (t, J = 7.1 Hz, 3H), 1.08 (t, J = 7.4 Hz, 3H). ^{13}C NMR (101 MHz, $CDCl_3$) δ 206.7, 165.4, 155.9, 142.7, 122.1, 116.7, 109.0, 59.2, 53.3, 38.5, 29.1, 18.4, 14.3, 13.6, 11.3. HRMS (ESI) m/z calcd for $C_{15}H_{23}N_2O_5$ $[M+H]^+$ = 311.1601, found = 311.1596.

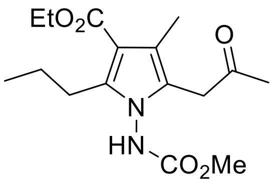

3n

Ethyl 1-((methoxycarbonyl)amino)-4-methyl-5-(2-oxopropyl)-2-propyl-1H-pyrrole-3-carboxylate (3n)

(GP-1) A light-yellow oil; isolated yield = 78%. ^{1}H NMR (400 MHz, $CDCl_3$) δ 7.80 (s, 1H), 4.23 (q, *J* = 7.1 Hz, 2H), 3.76 (s, 3H), 3.67 (d, *J* = 17.6 Hz, 1H), 3.37 (d, *J* = 17.1 Hz, 1H), 2.95 – 2.53 (m, 2H), 2.17 (s, 3H), 2.14 (s, 3H), 1.51 (qd, *J* = 8.2, 4.3 Hz, 2H), 1.31 (t, *J* = 7.1 Hz, 3H), 0.91 (t, *J* = 7.3 Hz, 3H). ^{13}C NMR (101 MHz, $CDCl_3$) δ 206.6, 165.5, 155.8, 141.4, 122.1, 116.8, 109.5, 59.2, 53.4, 38.5, 29.3, 27.0, 22.7, 14.32, 14.0, 11.4. HRMS (ESI) m/z calcd for $C_{16}H_{25}N_2O_5$ $[M+H]^+$ = 325.1758, found = 325.1758.

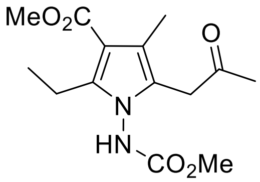

3o

Methyl 2-ethyl-1-((methoxycarbonyl)amino)-4-methyl-5-(2-oxopropyl)-1H-pyrrole-3-carboxylate (3o)

(GP-1) A light-yellow oil; isolated yield = 72%. ^{1}H NMR (400 MHz, $CDCl_3$) δ 7.84 (s, 1H), 3.77 (s, 6H), 3.68 (d, *J* = 17.2 Hz, 1H), 3.38 (d, *J* = 17.1 Hz, 1H), 2.95 – 2.71 (m, 2H), 2.16 (s, 3H), 2.15 (s, 3H), 1.10 (t, *J* = 7.4 Hz, 3H). ^{13}C NMR (101 MHz, $CDCl_3$) δ 206.7, 165.9, 155.8, 142.9, 122.1, 116.7, 108.9, 53.4, 50.6, 38.5, 29.3, 18.4, 13.7, 11.4. HRMS (ESI) m/z calcd for $C_{14}H_{21}N_2O_5$ $[M+H]^+$ = 297.1445, found = 297.1447.

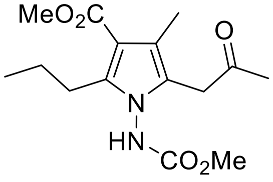

3p

Methyl 1-((methoxycarbonyl)amino)-4-methyl-5-(2-oxopropyl)-2-propyl-1H-pyrrole-3-carboxylate (3p)

(GP-1) A light-yellow oil; isolated yield = 86%. ^{1}H NMR (400 MHz, $CDCl_3$) δ 7.76 (s, 1H), 3.77 (s, 6H), 3.68 (d, J = 17.5 Hz, 1H), 3.37 (d, J = 17.1 Hz, 1H), 2.97 – 2.56 (m, 2H), 2.16 (s, 3H), 2.15 (s, 3H), 1.68 – 1.42 (m, 2H), 0.91 (t, J = 7.3 Hz, 3H). ^{13}C NMR (101 MHz, $CDCl_3$) δ 206.6, 165.9, 155.8, 141.6, 122.1, 116.7, 109.3, 53.4, 50.5, 38.5, 29.3, 26.9, 22.7, 14.0, 11.4. HRMS (ESI) m/z calcd for $C_{15}H_{23}N_2O_5$ [M+H]$^+$ = 311.1601, found = 311.1599.

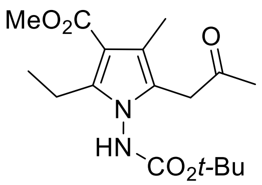

3q

Methyl 1-((tert-butoxycarbonyl)amino)-2-ethyl-4-methyl-5-(2-oxopropyl)-1H-pyrrole-3-carboxylate (3q)

(GP-1) A light-yellow oil; isolated yield = 56%. ^{1}H NMR (400 MHz, $CDCl_3$) δ 7.42 (s, 1H), 3.77 (s, 3H), 3.66 (d, J = 17.1 Hz, 1H), 3.36 (d, J = 17.0 Hz, 1H), 2.95 – 2.65 (m, 2H), 2.16 (s, 3H), 2.15 (s, 3H), 1.46 (s, 10H), 1.11 (t, J = 7.2 Hz, 3H). ^{13}C NMR (101 MHz, $CDCl_3$) δ 206.4, 166.0, 154.2, 143.0, 122.2, 116.6, 108.7, 82.5, 50.5, 38.7, 29.2, 28.0, 18.5, 13.7, 11.4. HRMS (ESI) m/z calcd for $C_{17}H_{27}N_2O_5$ [M+H]$^+$ = 339.1914, found = 339.1908.

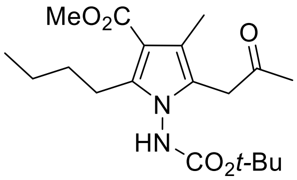

3r

Methyl 1-((tert-butoxycarbonyl)amino)-2-butyl-4-methyl-5-(2-oxopropyl)-1H-pyrrole-3-carboxylate (3r)

(GP-1) A light-yellow oil; isolated yield = 61%. ^{1}H NMR (400 MHz, $CDCl_3$) δ 7.33 (s, 1H), 3.76 (s, 3H), 3.67 (d, J = 17.0 Hz, 1H), 3.36 (d, J = 17.1 Hz, 1H), 2.96 – 2.58 (m, 2H), 2.16 (s, 3H), 2.15 (s, 3H), 1.67 – 1.27 (m, 13H), 0.89 (t, J = 7.3 Hz, 3H). ^{13}C NMR (101 MHz, $CDCl_3$) δ 206.4, 166.0, 154.2, 141.9, 122.3, 116.6, 109.0, 82.5, 50.4, 38.7, 31.5, 29.3, 28.0, 24.8, 22.7, 13.8, 11.5. HRMS (ESI) m/z calcd for $C_{19}H_{31}N_2O_5$ [M+H]$^+$ = 367.2227, found = 367.2222.

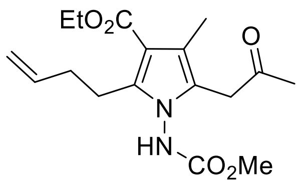

3s

Ethyl 2-(but-3-en-1-yl)-1-((methoxycarbonyl)amino)-4-methyl-5-(2-oxopropyl)-1H-pyrrole-3-carboxylate (3s)

(GP-1) A light-yellow oil; isolated yield = 61%. ^{1}H NMR (400 MHz, $CDCl_3$) δ 7.70 (s, 1H), 5.95 – 5.66 (m, 1H), 5.07 – 4.84 (m, 2H), 4.24 (q, *J* = 7.1 Hz, 2H), 3.76 (s, 3H), 3.68 (d, *J* = 17.1 Hz, 1H), 3.38 (d, *J* = 17.1 Hz, 1H), 3.08 – 2.67 (m, 2H), 2.32 – 2.22 (m, 2H), 2.18 (s, 3H), 2.15 (s, 3H), 1.32 (t, *J* = 7.1 Hz, 3H). ^{13}C NMR (101 MHz, $CDCl_3$) δ 206.4, 165.3, 155.7, 140.4, 137.7, 122.3, 116.9, 115.2, 109.6, 59.3, 53.5, 38.5, 33.5, 29.3, 24.7, 14.4, 11.4. HRMS (ESI) m/z calcd for $C_{17}H_{25}N_2O_5$ $[M+H]^+$ = 337.1758, found = 337.1757.

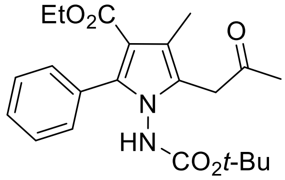

3t

Ethyl 1-((tert-butoxycarbonyl)amino)-4-methyl-5-(2-oxopropyl)-2-phenyl-1H-pyrrole-3-carboxylate (3t)

(GP-1) A light-yellow oil; isolated yield = 72%. ^{1}H NMR (400 MHz, $CDCl_3$) δ 7.39 – 7.28 (m, 5H), 6.99 (s, 1H), 4.04 (q, *J* = 7.1 Hz, 2H), 3.74 (d, *J* = 17.4 Hz, 1H), 3.51 (d, *J* = 17.2 Hz, 1H), 2.27 (s, 3H), 2.20 (s, 3H), 1.31 (s, 9H), 1.01 (t, *J* = 7.1 Hz, 3H). ^{13}C NMR (101 MHz, $CDCl_3$) δ 205.8, 165.0, 154.2, 138.9, 130.8, 130.5, 128.2, 127.6, 124.1, 117.1, 111.4, 82.3, 59.3, 38.9, 29.3, 27.8, 13.9, 11.2. HRMS (ESI) m/z calcd for $C_{22}H_{29}N_2O_5$ $[M+H]^+$ = 401.2071, found = 401.2070.

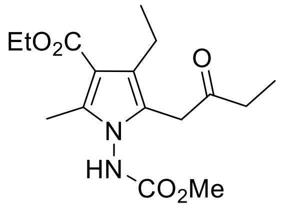

3ab

Ethyl 4-ethyl-1-((methoxycarbonyl)amino)-2-methyl-5-(2-oxobutyl)-1H-pyrrole-3-carboxylate (3ab)

(GP-1) A light-yellow oil; isolated yield = 71%. ^{1}H NMR (400 MHz, $CDCl_3$) δ 8.56 – 7.45 (m, 1H), 4.29 – 4.16 (m, 3H), 3.87 – 3.52 (m, 4H), 2.76 – 2.41 (m, 3H), 2.40 – 2.31 (m, 4H), 1.42 – 1.18 (m, 6H), 1.05 (dt, J = 8.7, 7.3 Hz, 3H). ^{13}C NMR (101 MHz, $CDCl_3$) δ 165.71, 155.65, 123.04, 122.38, 121.61, 108.73, 59.37, 53.49, 37.03, 35.48, 18.66, 14.27, 7.58. ^{13}C NMR (101 MHz, $CDCl_3$) δ 209.4, 165.7, 155.7, 123.0, 122.4, 121.6, 108.7, 59.4, 53.5, 37.0, 35.5, 18.7, 14.3, 7.6. HRMS (ESI) m/z calcd for $C_{16}H_{25}N_2O_5$ $[M+H]^+$ = 325.1758, found = 325.1759.

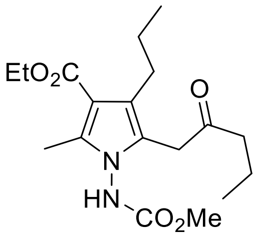

3ac

Ethyl 1-((methoxycarbonyl)amino)-2-methyl-5-(2-oxopentyl)-4-propyl-1H-pyrrole-3-carboxylate (3ac)

(GP-1) A light-yellow oil; isolated yield = 60%. ^{1}H NMR (400 MHz, $CDCl_3$) δ 8.54 – 7.77 (m, 1H), 4.41 – 4.15 (m, 3H), 3.88 – 3.32 (m, 4H), 3.05 – 2.36 (m, 4H), 2.36 – 2.31 (m, 3H), 1.74 – 0.55 (m, 13H). ^{13}C NMR (101 MHz, $CDCl_3$) δ 206.4, 165.8, 156.5, 125.9, 122.5, 121.0, 109.4, 59.4, 53.6, 44.3, 37.5, 27.5, 24.3, 16.0, 14.3, 14.1, 13.9, 10.8. HRMS (ESI) m/z calcd for $C_{18}H_{29}N_2O_5$ $[M+H]^+$ = 353.2071, found = 353.2060.

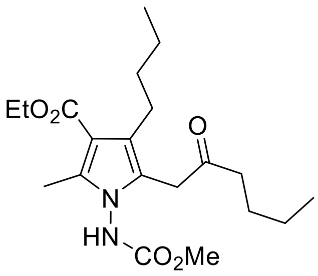

3ad

Ethyl 4-butyl-1-((methoxycarbonyl)amino)-2-methyl-5-(2-oxohexyl)-1H-pyrrole-3-carboxylate (3ad)

(GP-1) A light-yellow oil; isolated yield = 83%. ^{1}H NMR (400 MHz, $CDCl_3$) δ 8.46 – 7.58 (m, 1H), 4.31 – 4.16 (m, 3H), 3.88 – 3.41 (m, 4H), 2.90 – 2.38 (m, 4H), 2.34 (d, *J* = 7.2 Hz, 3H), 1.61 – 1.17 (m, 10H), 0.94 (t, *J* = 7.2 Hz, 3H), 0.85 (d, *J* = 6.7 Hz, 3H). ^{13}C NMR (101 MHz, $CDCl_3$) δ 166.3, 165.7, 156.3, 136.7, 135.0, 125.7, 122.9, 121.3, 121.0, 109.5, 59.4, 58.9, 53.6, 33.5, 27.4, 25.4, 24.8, 22.9, 19.4, 14.4, 14.4, 14.2, 14.0, 14.0, 10.8. HRMS (ESI) m/z calcd for $C_{20}H_{33}N_2O_5$ $[M+H]^+$ = 381.2384, found = 381.2380.

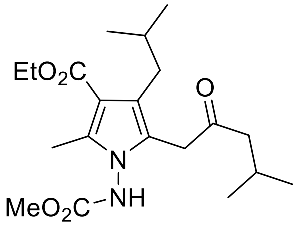

3ae

Ethyl 4-isobutyl-1-((methoxycarbonyl)amino)-2-methyl-5-(4-methyl-2-oxopentyl)-1H-pyrrole-3-carboxylate (3ae)

(GP-1) A light-yellow oil; isolated yield = 96%. ^{1}H NMR (400 MHz, $CDCl_3$) δ 7.65 (s, 1H), 4.23 (q, *J* = 7.1 Hz, 2H), 3.85 – 3.63 (m, 4H), 3.32 (d, *J* = 17.9 Hz, 1H), 2.56 (dd, *J* = 13.5, 6.4 Hz, 1H), 2.40 (s, 3H), 2.38 – 2.23 (m, 3H), 2.13 (dt, *J* = 13.4, 6.7 Hz, 1H), 1.78 (dt, *J* = 13.5, 6.7 Hz, 1H), 1.32 (t, *J* = 7.1 Hz, 3H), 0.91 (t, *J* = 6.3 Hz, 6H), 0.87 (d, *J* = 6.6 Hz, 3H), 0.76 (d, *J* = 6.6 Hz, 3H). ^{13}C NMR (101 MHz, $CDCl_3$) δ 208.5, 165.6, 155.6, 137.5, 122.4, 120.3, 109.1, 59.2, 53.4, 51.5, 38.3, 34.7, 29.6, 24.6, 22.6, 22.4, 22.4, 22.2, 14.4, 11.0. HRMS (ESI) m/z calcd for $C_{20}H_{33}N_2O_5$ $[M+H]^+$ = 381.2384, found = 381.2384.

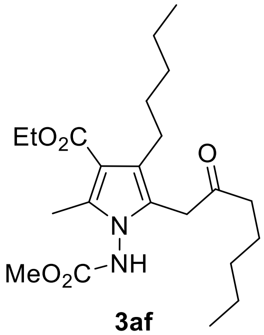

3af

Ethyl 1-((methoxycarbonyl)amino)-2-methyl-5-(2-oxoheptyl)-4-pentyl-1H-pyrrole-3-carboxylate (3af)

(GP-1) A light-yellow oil; isolated yield = 60%. ^{1}H NMR (400 MHz, $CDCl_3$) δ 7.69 (s, 1H), 4.24 (q, *J* = 7.1 Hz, 2H), 3.85 – 3.66 (m, 4H), 3.34 (d, *J* = 17.4 Hz, 1H), 2.72 – 2.42 (m, 4H), 2.39 (s, 3H), 1.61 – 1.37 (m, 4H), 1.35 – 1.22 (m, 11H), 0.87 (dt, *J* = 8.9, 7.0 Hz, 6H). ^{13}C NMR (101 MHz, $CDCl_3$) δ 209.1, 165.5, 155.6, 137.3, 121.8, 121.5, 109.0, 59.2, 53.4, 42.5, 37.4, 32.0, 31.2, 25.7, 23.4, 22.6, 22.4, 14.4, 14.1, 13.8, 10.9. HRMS (ESI) m/z calcd for $C_{22}H_{37}N_2O_5$ $[M+H]^+$ = 409.2697, found = 409.2694.

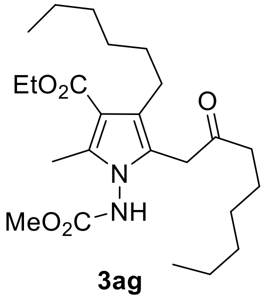

3ag

Ethyl 4-hexyl-1-((methoxycarbonyl)amino)-2-methyl-5-(2-oxooctyl)-1H-pyrrole-3-carboxylate (3ag)

(GP-1) A light-yellow oil; isolated yield = 92%. ^{1}H NMR (400 MHz, $CDCl_3$) δ 7.68 (s, 1H), 4.24 (q, *J* = 7.1 Hz, 2H), 3.74 (d, *J* = 17.7 Hz, 4H), 3.34 (d, *J* = 17.4 Hz, 1H), 2.76 – 2.43 (m, 4H), 2.39 (s, 3H), 1.69 – 1.36 (m, 4H), 1.35 – 1.21 (m, 15H), 0.87 (q, *J* = 6.3 Hz, 6H). ^{13}C NMR (101 MHz, $CDCl_3$) δ 209.0, 165.5, 155.6, 137.3, 121.8, 121.5, 109.0, 59.2, 53.4, 42.6, 37.4, 31.8, 31.6, 31.5, 29.5, 28.8, 25.8, 23.7, 22.7, 22.4, 14.4, 14.0, 14.0, 11.0. HRMS (ESI) m/z calcd for $C_{24}H_{41}N_2O_5$ $[M+H]^+$ = 437.3010, found = 437.3001.

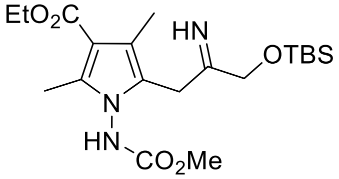

3ah

Ethyl 5-(3-((tert-butyldimethylsilyl)oxy)-2-iminopropyl)-1-((methoxycarbonyl)amino)-2,4-dimethyl-1H-pyrrole-3-carboxylate (3ah)

(GP-1) A light-yellow oil; isolated yield = 76%. ^{1}H NMR (400 MHz, $CDCl_3$) δ 7.00 (s, 1H), 4.59 – 3.49 (m, 9H), 2.09 (s, 3H), 2.04 (s, 3H), 1.26 (t, *J* = 7.2 Hz, 3H), 0.82 (s, 9H), -0.02 (d, *J* = 6.6 Hz, 6H). ^{13}C NMR (101 MHz, $CDCl_3$) δ 172.5, 166.1, 162.6, 156.0, 101.0, 89.3, 69.0, 66.9, 59.1,

52.8, 45.6, 25.8, 19.5, 18.1, 14.4, 12.0, -5.5, -5.7. HRMS (ESI) m/z calcd for $C_{20}H_{36}N_3O_5Si$ $[M+H]^+$ = 426.2419, found = 426.2418.

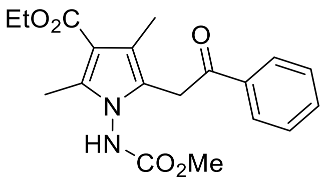

3ai

Ethyl 1-((methoxycarbonyl)amino)-2,4-dimethyl-5-(2-oxo-2-phenylethyl)-1H-pyrrole-3-carboxylate (3ai)

(GP-2) A light-yellow oil; isolated yield = 73%. ^{1}H NMR (400 MHz, $CDCl_3$) δ 8.05 – 7.98 (m, 2H), 7.65 – 7.60 (m, 1H), 7.55 – 7.46 (m, 2H), 4.50 (d, *J* = 17.7 Hz, 1H), 4.26 (q, *J* = 7.1 Hz, 2H), 3.83 (d, *J* = 17.7 Hz, 1H), 3.75 (s, 3H), 2.44 (s, 3H), 2.19 (s, 3H), 1.33 (t, *J* = 7.1 Hz, 4H). ^{13}C NMR (101 MHz, $CDCl_3$) δ 165.7, 155.6, 137.3, 136.1, 133.8, 128.9, 128.4, 121.6, 116.8, 109.8, 59.2, 53.5, 33.3, 14.5, 11.5, 11.0. HRMS (ESI) m/z calcd for $C_{19}H_{23}N_2O_5$ $[M+H]^+$ = 359.1601, found = 359.1594.

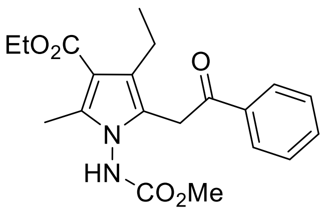

3aj

Ethyl 4-ethyl-1-((methoxycarbonyl)amino)-2-methyl-5-(2-oxo-2-phenylethyl)-1H-pyrrole-3-carboxylate (3aj)

(GP-2) A light-yellow oil; isolated yield = 66%. ^{1}H NMR (400 MHz, $CDCl_3$) δ 8.07 – 7.95 (m, 2H), 7.62 (d, *J* = 7.4 Hz, 1H), 7.56 – 7.48 (m, 2H), 4.50 (d, *J* = 17.8 Hz, 1H), 4.27 (q, *J* = 7.1 Hz, 2H), 3.83 (d, *J* = 17.8 Hz, 1H), 3.75 (s, 3H), 2.78 – 2.50 (m, 2H), 2.44 (s, 3H), 1.33 (t, *J* = 7.1 Hz, 3H), 1.03 (t, *J* = 7.4 Hz, 3H). ^{13}C NMR (101 MHz, $CDCl_3$) δ 197.5, 165.5, 155.6, 136.0, 133.8, 128.8, 128.4, 123.4, 121.3, 108.9, 59.2, 53.4, 33.1, 19.0, 16.1, 14.4, 11.0. HRMS (ESI) m/z calcd for $C_{20}H_{25}N_2O_5$ $[M+H]^+$ = 373.1758, found = 373.1749.

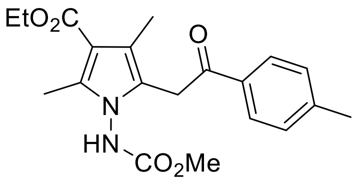

3ak

Ethyl 1-((methoxycarbonyl)amino)-2,4-dimethyl-5-(2-oxo-2-(p-tolyl)ethyl)-1H-pyrrole-3-carboxylate (3ak)

(GP-2) A light-yellow oil; isolated yield = 70%. ^{1}H NMR (400 MHz, $CDCl_3$) δ 7.90 (d, *J* = 8.3 Hz, 2H), 7.80 (s, 1H), 7.29 (d, *J* = 7.9 Hz, 2H), 4.49 (s, 1H), 4.25 (q, *J* = 7.1 Hz, 2H), 3.79 (d, *J* = 17.7 Hz, 1H), 3.74 (s, 3H), 2.42 (d, *J* = 4.2 Hz, 6H), 2.18 (s, 3H), 1.32 (t, *J* = 7.1 Hz, 3H). ^{13}C NMR (101 MHz, $CDCl_3$) δ 197.16, 165.75, 155.69, 144.81, 137.25, 133.57, 129.49, 128.54, 121.83, 116.65, 109.71, 59.19, 53.40, 33.10, 21.68, 14.46, 11.47, 10.94. HRMS (ESI) m/z calcd for $C_{20}H_{25}N_2O_5$ $[M+H]^+$ = 373.1758, found = 373.1747.

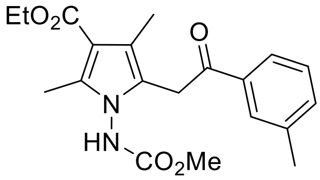

3al

Ethyl 1-((methoxycarbonyl)amino)-2,4-dimethyl-5-(2-oxo-2-(m-tolyl)ethyl)-1H-pyrrole-3-carboxylate (3al)

(GP-2) A light-yellow oil; isolated yield = 75%. ^{1}H NMR (400 MHz, $CDCl_3$) δ 7.81 (s, 2H), 7.75 – 7.48 (m, 1H), 7.47 – 7.33 (m, 2H), 4.49 (d, *J* = 17.7 Hz, 1H), 4.26 (q, *J* = 7.1 Hz, 2H), 3.91 – 3.65 (m, 4H), 2.43 (s, 6H), 2.19 (s, 3H), 1.33 (t, *J* = 7.1 Hz, 3H). ^{13}C NMR (101 MHz, $CDCl_3$) δ 197.7, 165.7, 155.6, 138.7, 136.1, 134.6, 128.9, 128.7, 125.6, 121.7, 116.7, 109.8, 59.2, 53.4, 33.3, 21.4, 14.5, 11.5, 11.0. HRMS (ESI) m/z calcd for $C_{20}H_{25}N_2O_5$ $[M+H]^+$ = 373.1758, found = 373.1759.

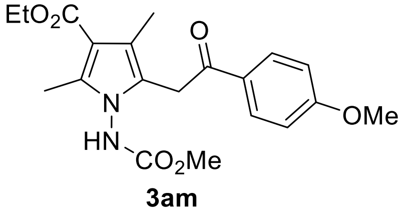

3am

Ethyl 1-((methoxycarbonyl)amino)-5-(2-(4-methoxyphenyl)-2-oxoethyl)-2,4-dimethyl-1H-pyrrole-3-carboxylate (3am)

(GP-2) A light-yellow oil; isolated yield = 75%. ^{1}H NMR (400 MHz, $CDCl_3$) δ 7.99 (d, *J* = 9.0 Hz, 2H), 7.80 (s, 1H), 6.96 (d, *J* = 8.9 Hz, 2H), 4.45 (d, *J* = 17.5 Hz, 1H), 4.25 (q, *J* = 7.1 Hz, 2H), 3.88 (s, 3H), 3.75 (d, *J* = 7.8 Hz, 4H), 2.42 (s, 3H), 2.18 (s, 3H), 1.32 (t, *J* = 7.1 Hz, 3H). ^{13}C NMR (101 MHz, $CDCl_3$) δ 196.1, 165.7, 164.1, 155.7, 137.2, 130.8, 129.0, 121.9, 116.6, 114.0, 109.7, 59.2, 55.5, 53.4, 32.8, 14.5, 11.5, 11.0. HRMS (ESI) m/z calcd for $C_{20}H_{25}N_2O_6$ $[M+H]^+$ = 389.1707, found = 389.1710.

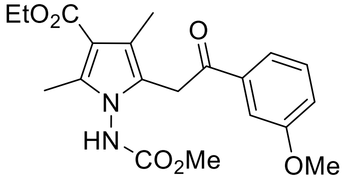

3an

Ethyl 1-((methoxycarbonyl)amino)-5-(2-(3-methoxyphenyl)-2-oxoethyl)-2,4-dimethyl-1H-pyrrole-3-carboxylate (3an)

(GP-2) A light-yellow oil; isolated yield = 62%. ^{1}H NMR (400 MHz, $CDCl_3$) δ 7.84 – 7.33 (m, 4H), 7.22 – 7.10 (m, 1H), 4.46 (d, *J* = 17.8 Hz, 1H), 4.25 (q, *J* = 7.1 Hz, 2H), 3.85 (s, 4H), 3.75 (s, 3H), 2.42 (s, 3H), 2.18 (s, 3H), 1.33 (t, *J* = 7.1 Hz, 3H). ^{13}C NMR (101 MHz, $CDCl_3$) δ 197.2, 165.7, 159.9, 155.6, 137.4, 129.8, 121.7, 121.0, 120.1, 116.8, 112.8, 109.8, 77.0, 59.2, 55.5, 53.5, 33.4, 14.5, 11.5, 11.0. HRMS (ESI) m/z calcd for $C_{20}H_{25}N_2O_6$ $[M+H]^+$ = 389.1707, found = 389.1713.

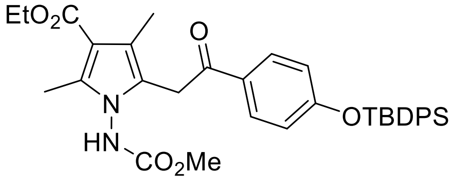

3ao

Ethyl 5-(2-(4-((*tert*-butyldiphenylsilyl)oxy)phenyl)-2-oxoethyl)-1-((methoxycarbonyl)amino)-2,4-dimethyl-1H-pyrrole-3-carboxylate (3ao)

(GP-2) A light-yellow oil; isolated yield = 82%. ^{1}H NMR (400 MHz, $CDCl_3$) δ 7.80 (d, *J* = 8.8 Hz, 2H), 7.71 (d, *J* = 6.6 Hz, 4H), 7.51 – 7.35 (m, 6H), 6.82 (d, *J* = 8.8 Hz, 2H), 4.39 (d, *J* = 17.6 Hz,

1H), 4.25 (q, J = 7.1 Hz, 2H), 3.77 – 3.62 (m, 4H), 2.41 (s, 3H), 2.14 (s, 3H), 1.32 (t, J = 7.2 Hz, 3H), 1.12 (s, 9H). ^{13}C NMR (101 MHz, $CDCl_3$) δ 196.0, 165.7, 160.7, 155.7, 135.4, 131.9, 130.5, 130.2, 129.4, 119.9, 116.5, 109.6, 77.0, 59.2, 53.4, 32.7, 26.4, 19.4, 14.5, 11.5, 11.0. HRMS (ESI) m/z calcd for $C_{35}H_{41}N_2O_6Si$ $[M+H]^+$ = 613.2728, found = 613.2733.

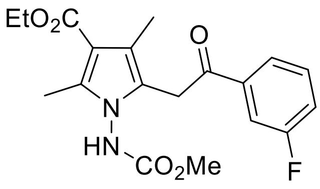

3ap

Ethyl 5-(2-(3-fluorophenyl)-2-oxoethyl)-1-((methoxycarbonyl)amino)-2,4-dimethyl-1H-pyrrole-3-carboxylate (3ap)

(GP-2) A light-yellow oil; isolated yield = 63%. ^{1}H NMR (400 MHz, $CDCl_3$) δ 7.78 – 7.71 (m, 1H), 7.60 (ddd, J = 9.4, 4.7, 2.3 Hz, 1H), 7.42 (ddd, J = 8.0, 5.3, 2.8 Hz, 1H), 7.25 (dq, J = 5.7, 3.0 Hz, 1H), 4.35 (d, J = 17.9 Hz, 1H), 4.27 – 4.14 (m, 2H), 3.78 (d, J = 17.8 Hz, 1H), 3.68 (s, 3H), 2.35 (s, 3H), 2.11 (s, 3H), 1.31 – 1.24 (m, 3H). ^{13}C NMR (101 MHz, $CDCl_3$) δ 198.2, 165.7, 164.1, 155.6, 138.1, 137.4, 130.6, 130.5, 124.2, 124.2, 121.4, 121.0, 120.8, 117.0, 115.3, 115.1, 109.9, 59.3, 53.5, 33.6, 14.5, 11.5, 11.0. HRMS (ESI) m/z calcd for $C_{19}H_{22}FN_2O_5$ $[M+H]^+$ = 377.1507, found = 377.1510.

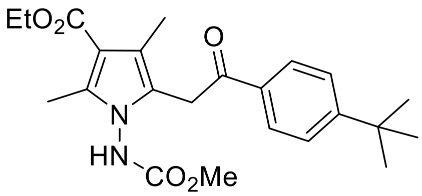

3aq

Ethyl 5-(2-(4-(tert-butyl)phenyl)-2-oxoethyl)-1-((methoxycarbonyl)amino)-2,4-dimethyl-1H-pyrrole-3-carboxylate (3aq)

(GP-2) A light-yellow oil; isolated yield = 73%. ^{1}H NMR (400 MHz, $CDCl_3$) δ 7.96 (d, J = 8.5 Hz, 2H), 7.84 – 7.59 (m, 1H), 7.52 (d, J = 8.5 Hz, 2H), 4.50 (d, J = 17.6 Hz, 1H), 4.26 (q, J = 7.1 Hz, 2H), 3.86 – 3.63 (m, 4H), 2.43 (s, 3H), 2.19 (s, 3H), 1.34 (d, J = 10.6 Hz, 12H). ^{13}C NMR (101 MHz, $CDCl_3$) δ 197.1, 165.7, 157.8, 155.7, 137.3, 133.5, 128.4, 125.8, 121.8, 116.7, 109.7, 77.0,

59.2, 53.4, 35.2, 33.1, 31.0, 14.5, 11.5, 11.0. HRMS (ESI) m/z calcd for $C_{23}H_{31}N_2O_5$ $[M+H]^+$ = 415.2227, found = 415.2230.

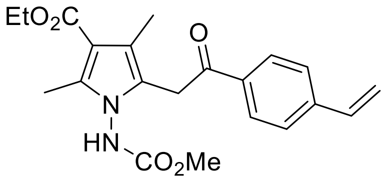

3ar

Ethyl 1-((methoxycarbonyl)amino)-2,4-dimethyl-5-(2-oxo-2-(4-vinylphenyl)ethyl)-1H-pyrrole-3-carboxylate (3ar)

(GP-2) A light-yellow oil; isolated yield = 70%. ^{1}H NMR (400 MHz, $CDCl_3$) δ 7.97 (d, *J* = 8.5 Hz, 2H), 7.72 (s, 1H), 7.51 (d, *J* = 8.3 Hz, 2H), 6.77 (dd, *J* = 17.6, 10.9 Hz, 1H), 5.90 (d, *J* = 17.6 Hz, 1H), 5.43 (d, *J* = 11.5 Hz, 1H), 4.47 (d, *J* = 17.6 Hz, 1H), 4.25 (q, *J* = 7.1 Hz, 2H), 3.81 (d, *J* = 17.7 Hz, 1H), 3.74 (s, 3H), 2.43 (s, 3H), 2.19 (s, 3H), 1.33 (t, *J* = 7.1 Hz, 3H). ^{13}C NMR (101 MHz, $CDCl_3$) δ 196.8, 165.7, 155.7, 142.8, 137.3, 135.7, 135.1, 128.8, 126.5, 121.7, 117.3, 116.7, 109.8, 59.2, 53.4, 33.2, 14.5, 11.5, 11.0. HRMS (ESI) m/z calcd for $C_{21}H_{25}N_2O_5$ $[M+H]^+$ = 385.1758, found = 385.1761.

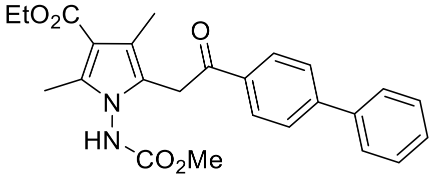

3as

Ethyl 5-(2-([1,1'-biphenyl]-4-yl)-2-oxoethyl)-1-((methoxycarbonyl)amino)-2,4-dimethyl-1H-pyrrole-3-carboxylate (3as)

(GP-2) A light-yellow oil; isolated yield = 75%. ^{1}H NMR (400 MHz, $CDCl_3$) δ 8.09 (d, *J* = 8.5 Hz, 2H), 7.83 (s, 1H), 7.72 (d, *J* = 8.4 Hz, 2H), 7.64 (d, *J* = 7.0 Hz, 2H), 7.52 – 7.46 (m, 2H), 7.45 – 7.38 (m, 1H), 4.53 (d, *J* = 17.7 Hz, 1H), 4.26 (qd, *J* = 7.1, 3.4 Hz, 3H), 3.87 (d, *J* = 13.4 Hz, 1H), 3.76 (s, 3H), 2.44 (s, 3H), 2.21 (s, 3H), 1.33 (t, *J* = 7.0 Hz, 3H). ^{13}C NMR (101 MHz, $CDCl_3$) δ 196.6, 165.7, 155.7, 146.6, 139.6, 137.3, 134.8, 129.02, 128.5, 127.3, 121.7, 116.8, 77.0, 59.2,

53.5, 33.3, 14.5, 11.5, 11.0. HRMS (ESI) m/z calcd for $C_{25}H_{27}N_2O_5$ $[M+H]^+$ = 435.1914, found = 435.1919.

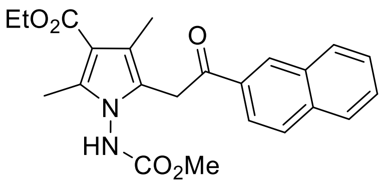

3at

Ethyl 1-((methoxycarbonyl)amino)-2,4-dimethyl-5-(2-(naphthalen-2-yl)-2-oxoethyl)-1H-pyrrole-3-carboxylate (3at)

(GP-2) A light-yellow oil; isolated yield = 67%. ^1H NMR (400 MHz, $CDCl_3$) δ 8.56 (s, 1H), 8.03 (dd, *J* = 8.6, 1.8 Hz, 1H), 8.02 – 7.97 (m, 1H), 7.94 – 7.87 (m, 2H), 7.61 (dddd, *J* = 20.7, 8.2, 6.9, 1.4 Hz, 2H), 4.64 (d, *J* = 17.6 Hz, 1H), 4.26 (q, *J* = 7.1 Hz, 2H), 3.97 (d, *J* = 17.6 Hz, 1H), 3.74 (s, 3H), 2.45 (s, 3H), 2.23 (s, 3H), 1.33 (t, *J* = 7.1 Hz, 3H). ^{13}C NMR (101 MHz, $CDCl_3$) δ 197.6, 165.8, 155.7, 137.4, 135.9, 133.4, 132.5, 130.4, 129.7, 129.0, 128.8, 127.9, 127.1, 123.8, 121.8, 116.8, 109.9, 59.3, 53.5, 33.4, 14.5, 11.6, 11.0. HRMS (ESI) m/z calcd for $C_{23}H_{25}N_2O_5$ $[M+H]^+$ = 409.1758, found = 409.1755.

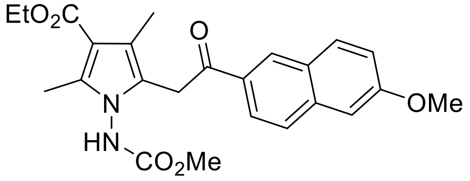

3au

Ethyl 1-((methoxycarbonyl)amino)-5-(2-(6-methoxynaphthalen-2-yl)-2-oxoethyl)-2,4-dimethyl-1H-pyrrole-3-carboxylate (3au)

(GP-2) A light-yellow oil; isolated yield = 74%. ^1H NMR (400 MHz, $CDCl_3$) δ 8.47 (s, 1H), 8.07 – 7.69 (m, 4H), 7.25 – 7.07 (m, 2H), 4.61 (d, *J* = 17.6 Hz, 1H), 4.26 (q, *J* = 7.1 Hz, 2H), 3.96 (s, 4H), 3.74 (s, 3H), 2.43 (s, 3H), 2.22 (s, 3H), 1.33 (t, *J* = 7.1 Hz, 3H). ^{13}C NMR (101 MHz, $CDCl_3$) δ 197.1, 165.8, 160.1, 155.7, 137.6, 131.4, 131.2, 130.2, 127.7, 127.4, 124.6, 121.9, 120.0, 116.7, 109.8, 105.8, 59.2, 55.4, 33.2, 14.5, 11.5, 11.0. HRMS (ESI) m/z calcd for $C_{24}H_{27}N_2O_6$ $[M+H]^+$ = 439.1864, found = 439.1860.

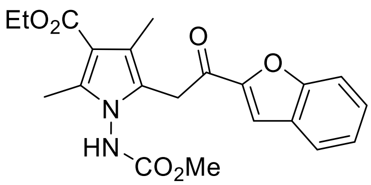

3av

Ethyl 5-(2-(benzofuran-2-yl)-2-oxoethyl)-1-((methoxycarbonyl)amino)-2,4-dimethyl-1H-pyrrole-3-carboxylate (3av)

(GP-2) A light-yellow oil; isolated yield = 71%. ^{1}H NMR (400 MHz, $CDCl_3$) δ 7.72 (d, *J* = 7.8 Hz, 2H), 7.63 – 7.56 (m, 2H), 7.51 (ddd, *J* = 8.5, 7.1, 1.3 Hz, 1H), 7.34 (ddd, *J* = 8.1, 7.1, 1.0 Hz, 1H), 4.49 (d, *J* = 17.3 Hz, 1H), 4.26 (q, *J* = 7.1 Hz, 2H), 3.81 (d, *J* = 34.4 Hz, 4H), 2.43 (s, 3H), 2.25 (s, 3H), 1.33 (t, *J* = 7.1 Hz, 3H). ^{13}C NMR (101 MHz, $CDCl_3$) δ 188.2, 165.7, 155.9, 155.7, 151.7, 137.6, 128.8, 128.8, 127.0, 124.2, 123.5, 120.9, 117.4, 112.5, 109.9, 59.3, 53.5, 33.6, 14.5, 11.5, 11.0. HRMS (ESI) m/z calcd for $C_{21}H_{23}N_2O_6$ $[M+H]^+$ = 399.1551, found = 399.1552.

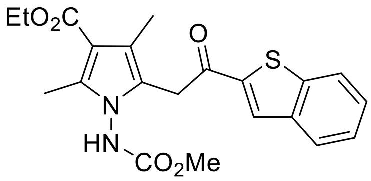

3aw

Ethyl 5-(2-(benzo[*b*]thiophen-2-yl)-2-oxoethyl)-1-((methoxycarbonyl)amino)-2,4-dimethyl-1H-pyrrole-3-carboxylate (3aw)

(GP-2) A light-yellow oil; isolated yield = 76%. ^{1}H NMR (400 MHz, $CDCl_3$) δ 8.09 (s, 1H), 7.89 (dd, *J* = 24.0, 7.9 Hz, 3H), 7.46 (dt, *J* = 23.9, 7.0 Hz, 2H), 4.49 (d, *J* = 17.2 Hz, 1H), 4.26 (q, *J* = 7.1 Hz, 2H), 3.88 (d, *J* = 17.2 Hz, 1H), 3.75 (s, 3H), 2.43 (s, 3H), 2.24 (s, 3H), 1.33 (t, *J* = 7.1 Hz, 3H). ^{13}C NMR (101 MHz, $CDCl_3$) δ 191.8, 165.7, 155.7, 142.9, 142.4, 139.0, 137.5, 130.2, 127.9, 127.9, 126.2, 125.2, 123.0, 121.3, 117.1, 109.8, 59.3, 53.5, 33.9, 14.5, 11.6, 11.0. HRMS (ESI) m/z calcd for $C_{21}H_{23}N_2O_5S$ $[M+H]^+$ = 415.1322, found = 415.1324.

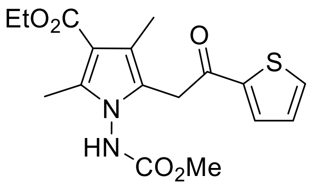

3ax

Ethyl 1-((methoxycarbonyl)amino)-2,4-dimethyl-5-(2-oxo-2-(thiophen-2-yl)ethyl)-1H-pyrrole-3-carboxylate (3ax)

(GP-2) A light-yellow oil; isolated yield = 78%. ^{1}H NMR (400 MHz, $CDCl_3$) δ 7.84 (d, *J* = 3.7 Hz, 1H), 7.71 (d, *J* = 4.9 Hz, 1H), 7.20 – 7.14 (m, 1H), 4.38 (d, *J* = 17.1 Hz, 1H), 4.25 (q, *J* = 7.1 Hz, 2H), 3.87 – 3.69 (m, 4H), 2.42 (s, 3H), 2.21 (s, 3H), 1.33 (t, *J* = 7.1 Hz, 3H). ^{13}C NMR (101 MHz, $CDCl_3$) δ 190.1, 165.7, 155.7, 143.1, 137.5, 135.1, 133.0, 128.5, 121.4, 117.0, 109.8, 59.3, 53.5, 34.0, 14.5, 11.5, 11.0. HRMS (ESI) m/z calcd for $C_{17}H_{21}N_2O_5S$ $[M+H]^+$ = 365.1166, found = 365.1170.

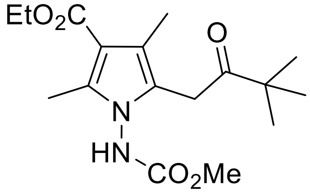

3az

Ethyl 5-(3,3-dimethyl-2-oxobutyl)-1-((methoxycarbonyl)amino)-2,4-dimethyl-1H-pyrrole-3-carboxylate (3az)

(GP-1) A light-yellow oil; isolated yield = 68%. ^{1}H NMR (400 MHz, $CDCl_3$) δ 7.74 (s, 1H), 4.25 (q, *J* = 7.1 Hz, 2H), 4.02 (d, *J* = 17.8 Hz, 1H), 3.78 (s, 3H), 3.29 (d, *J* = 17.8 Hz, 1H), 2.41 (s, 3H), 2.15 (s, 3H), 1.32 (t, *J* = 7.1 Hz, 3H), 1.21 (s, 9H). ^{13}C NMR (101 MHz, $CDCl_3$) δ 165.7, 155.7, 123.4, 122.0, 116.1, 109.5, 59.2, 53.4, 44.5, 31.3, 26.2, 14.5, 11.4, 10.9. HRMS (ESI) m/z calcd for $C_{17}H_{27}N_2O_5$ $[M+H]^+$ = 339.1914, found = 339.1917.

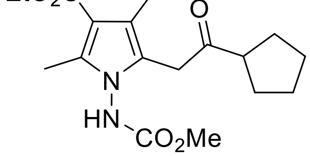

3az'

Ethyl 5-(2-cyclopentyl-2-oxoethyl)-1-((methoxycarbonyl)amino)-2,4-dimethyl-1H-pyrrole-3-carboxylate (3az')

(GP-1) A light-yellow oil; isolated yield = 74%. ^{1}H NMR (400 MHz, $CDCl_3$) δ 7.82 (s, 1H), 4.24 (q, J = 7.1 Hz, 2H), 3.88 – 3.61 (m, 4H), 3.38 (d, J = 17.1 Hz, 1H), 3.03 – 2.90 (m, 1H), 2.38 (s, 3H), 2.16 (s, 3H), 1.96 – 1.50 (m, 9H), 1.32 (t, J = 7.1 Hz, 3H). ^{13}C NMR (101 MHz, $CDCl_3$) δ 210.9, 165.7, 155.7, 137.1, 121.9, 116.3, 109.6, 77.0, 59.2, 53.4, 50.9, 36.5, 28.7, 26.0, 14.4, 11.4, 10.9. HRMS (ESI) m/z calcd for $C_{18}H_{27}N_2O_5$ $[M+H]^+$ = 351.1914, found = 351.1910.

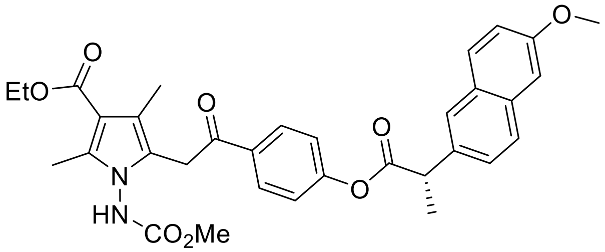

3ba

Ethyl (*S*)-1-((methoxycarbonyl)amino)-5-(2-(4-((2-(6-methoxynaphthalen-2-yl)propanoyl)oxy)phenyl)-2-oxoethyl)-2,4-dimethyl-1H-pyrrole-3-carboxylate (3ba)

(GP-2) A light-yellow oil; isolated yield = 68%.^{1}H NMR (400 MHz, $CDCl_3$) δ 7.92 (d, J = 8.8 Hz, 2H), 7.73 – 7.64 (m, 3H), 7.42 (dd, J = 8.6, 1.8 Hz, 1H), 7.14 – 7.02 (m, 4H), 4.35 (d, J = 17.7 Hz, 1H), 4.18 (q, J = 7.1 Hz, 2H), 4.05 (q, J = 7.1 Hz, 1H), 3.86 (s, 3H), 3.78 – 3.66 (m, 4H), 2.35 (s, 3H), 2.09 (s, 3H), 1.64 (d, J = 7.1 Hz, 3H), 1.26 (d, J = 7.1 Hz, 3H). ^{13}C NMR (101 MHz, $CDCl_3$) δ 196.1, 172.6, 165.7, 157.9, 155.6, 155.1, 137.1, 134.6, 133.9, 133.5, 130.0, 129.3, 129.0, 127.5, 126.2, 125.9, 121.9, 119.3, 116.8, 109.8, 105.6, 59.2, 55.3, 45.6, 33.3, 29.7, 18.4, 14.5, 11.5, 11.0. HRMS (ESI) m/z calcd for $C_{33}H_{35}N_2O_8$ $[M+H]^+$ = 587.2388, found = 587.2389; the ee value was > 99%, t (minor) = 15.2 min, t (major) = 21.7 min (Chiralpak IC, λ = 220 nm, 10% *i*-PrOH/Hexane, flow rate = 1 mL/min).

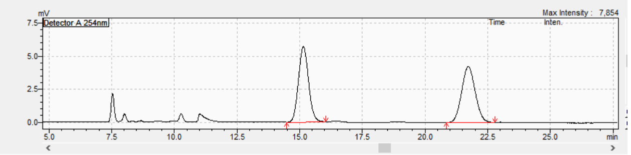

Results View - Peak Table

Peak#	Ret. Time	Area	Height	Conc.	Unit	ID#	Name	Area%
1	15.149	159473	5703	49.899				49.899
2	21.727	160120	4243	50.101				50.101
Total		319594	9946	100.000				100.000

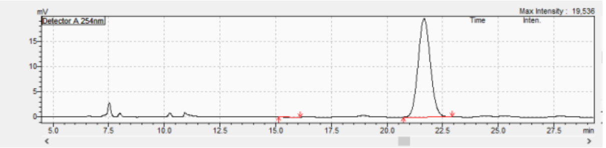

Results View - Peak Table

Peak#	Ret. Time	Area	Height	Conc.	Unit	ID#	Name	Area%
1	15.175	203	3	0.027				0.027
2	21.662	742078	19523	99.973				99.973
Total		742281	19525	100.000				100.000

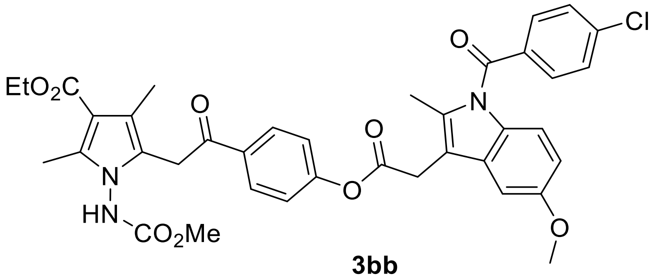

3bb

Ethyl 5-(2-(4-(2-(1-(4-chlorobenzoyl)-5-methoxy-2-methyl-1*H*-indol-3-yl)acetoxy)phenyl)-2-oxoethyl)-1-((methoxycarbonyl)amino)-2,4-dimethyl-1*H*-pyrrole-3-carboxylate (3bb)

(GP-2) A light-yellow oil; isolated yield = 73%.^{1}H NMR (400 MHz, $CDCl_3$) δ 8.03 (td, *J* = 5.0, 2.1 Hz, 2H), 7.68 (ddd, *J* = 7.7, 4.9, 2.4 Hz, 2H), 7.49 (td, *J* = 4.9, 2.2 Hz, 2H), 7.27 (d, *J* = 1.9 Hz, 1H), 7.25 – 7.14 (m, 2H), 7.05 (q, *J* = 2.5 Hz, 1H), 6.89 (dd, *J* = 9.1, 4.3 Hz, 1H), 6.78 – 6.67 (m, 1H), 4.43 (d, *J* = 17.9 Hz, 1H), 4.32 – 4.21 (m, 2H), 3.94 (d, *J* = 4.2 Hz, 2H), 3.85 (s, 4H), 3.74 (s, 3H), 2.44 (d, *J* = 19.6 Hz, 6H), 2.17 (s, 3H), 1.36 – 1.31 (m, 3H). ^{13}C NMR (101 MHz, $CDCl_3$) δ 196.0, 168.7, 168.3, 156.2, 155.6, 139.5, 136.4, 133.7, 133.7, 131.2, 130.9, 130.1, 129.2, 121.9, 121.5, 116.8, 115.1, 111.7, 111.4, 101.3, 59.2, 55.8, 53.5, 33.4, 30.6, 14.5, 13.4, 11.5, 10.9. HRMS (ESI) m/z calcd for $C_{38}H_{37}ClN_3O_9$ $[M+H]^+$ = 714.2213, found = 714.2210.

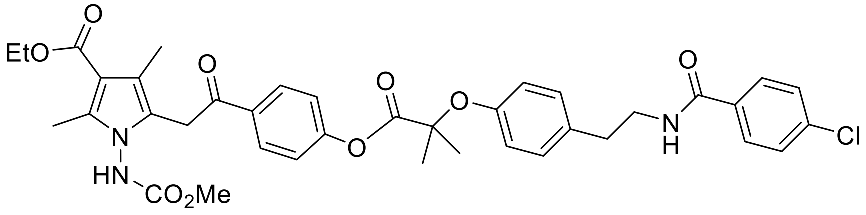

3bc

Ethyl 5-(2-(4-((2-(4-(2-(4-chlorobenzamido)ethyl)phenoxy)-2-methylpropanoyl)oxy) phenyl)-2-oxoethyl)-1-((methoxycarbonyl)amino)-2,4-dimethyl-1*H*-pyrrole-3-carboxylate (3bc)

(GP-2) A light-yellow oil; isolated yield = 76%.^{1}H NMR (400 MHz, $CDCl_3$) δ 7.94 (d, *J* = 8.7 Hz, 2H), 7.53 (d, *J* = 8.5 Hz, 2H), 7.27 (d, *J* = 8.5 Hz, 2H), 7.07 (dd, *J* = 8.7, 7.0 Hz, 4H), 6.85 (d, *J* = 8.6 Hz, 2H), 6.12 (s, 1H), 4.34 (d, *J* = 17.7 Hz, 1H), 4.18 (q, *J* = 7.1 Hz, 2H), 3.77 (d, *J* = 17.5 Hz, 1H), 3.66 (s, 3H), 3.59 (q, *J* = 6.7 Hz, 2H), 2.81 (t, *J* = 7.0 Hz, 2H), 2.34 (s, 3H), 2.10 (s, 3H), 1.68

(s, 6H), 1.25 (t, *J* = 7.1 Hz, 3H). ^{13}C NMR (126 MHz, $CDCl_3$) δ 195.9, 172.5, 154.7, 154.0, 133.9, 133.0, 130.2, 129.7, 128.8, 128.3, 121.8, 121.6, 119.5, 79.3, 59.3, 535, 41.3, 34.8, 33.5, 25.4, 14.5, 11.6, 11.0. HRMS (ESI) m/z calcd for $C_{38}H_{41}ClN_3O_9$ $[M+H]^+$ = 718.2526, found = 718.2527.

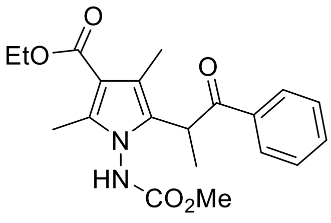

3bd

Ethyl 1-((methoxycarbonyl)amino)-2,4-dimethyl-5-(1-oxo-1-phenylpropan-2-yl)-1H-pyrrole-3-carboxylate (3bd)

(GP-2) A light-yellow oil; isolated yield = 57%. ^{1}H NMR (400 MHz, $CDCl_3$) δ 8.00 – 7.76 (m, 3H), 7.56 (t, *J* = 7.4 Hz, 1H), 7.45 (t, *J* = 7.7 Hz, 1H), 7.36 (t, *J* = 7.6 Hz, 1H), 4.84 – 4.58 (m, 1H), 4.24 (q, *J* = 7.1 Hz, 2H), 3.87 – 3.74 (m, 3H), 2.39 – 2.31 (m, 3H), 2.22 (d, *J* = 7.0 Hz, 3H), 1.47 (d, *J* = 7.4 Hz, 3H), 1.32 (s, 3H). ^{13}C NMR (101 MHz, $CDCl_3$) δ 199.2, 165.7, 155.5, 133.5, 132.8, 128.8, 128.6, 128.4, 128.2, 126.5, 109.8, 59.4, 53.5, 38.1, 29.7, 14.5, 11.2, 10.8. HRMS (ESI) m/z calcd for $C_{20}H_{25}N_2O_5$ $[M+H]^+$ = 373.1758, found = 373.1761.

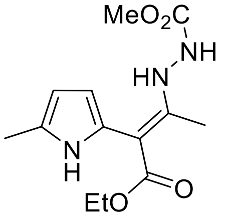

3be'

Methyl (E)-2-(4-ethoxy-3-(5-methyl-1H-pyrrol-2-yl)-4-oxobut-2-en-2-yl)hydrazine-1-carboxylate (3be')

(GP-1) A light-yellow oil; isolated yield = 91%. ^{1}H NMR (400 MHz, $CDCl_3$) δ 8.77 (s, 1H), 7.90 (s, 1H), 5.93 (t, *J* = 3.0 Hz, 1H), 5.79 – 5.74 (m, 1H), 4.69 (s, 1H), 4.20 (dq, *J* = 7.0, 3.9 Hz, 2H), 3.81 (s, 3H), 2.22 (s, 3H), 1.84 (s, 3H), 1.26 (t, *J* = 7.1 Hz, 3H). ^{13}C NMR (101 MHz, $CDCl_3$) δ 170.2, 168.5, 128.4, 122.4, 109.1, 108.0, 106.0, 61.5, 53.0, 14.0, 13.3, 12.9. HRMS (ESI) m/z calcd for $C_{13}H_{20}N_3O_4$ $[M+H]^+$ = 282.1448, found = 282.1449.

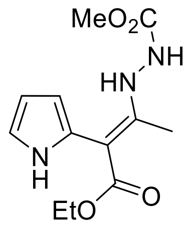

3bf'

Methyl (E)-2-(4-ethoxy-4-oxo-3-(1H-pyrrol-2-yl)but-2-en-2-yl)hydrazine-1-carboxylate (3bf')

(GP-1) A light-yellow oil; isolated yield = 73%. ^{1}H NMR (400 MHz, $CDCl_3$) δ 8.39 (s, 1H), 7.69 (s, 1H), 6.74 (dd, *J* = 10.3, 2.3 Hz, 2H), 6.14 (q, *J* = 1.2 Hz, 1H), 4.75 (s, 1H), 4.18 (dtt, *J* = 10.1, 7.2, 3.6 Hz, 2H), 3.82 (s, 3H), 1.84 (s, 3H), 1.25 (t, *J* = 7.1 Hz, 4H). ^{13}C NMR (101 MHz, $CDCl_3$) δ 171.5, 169.7, 151.4, 118.2, 117.1, 116.8, 108.9, 108.4, 61.0, 52.7, 14.1, 12.9. HRMS (ESI) m/z calcd for $C_{12}H_{18}N_3O_4$ $[M+H]^+$ = 268.1292, found = 268.1290.

4. Synthesis of ^{13}C-labeled fully-substituted pyrroles

A. Synthesis of ^{13}C-labeled azoalkenes

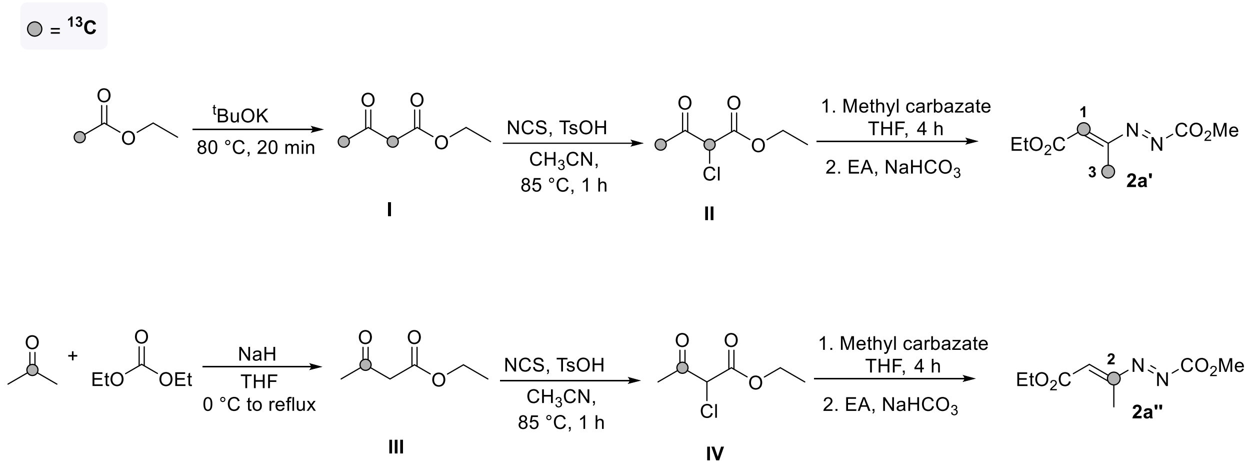

For the synthesis of 1,3-^{13}C-labeled azoalkene (**2a'**):

After a mixture of ethyl acetate-2-^{13}C (2.0 eq) and powdered ^{t}BuOK (1.4 eq) was kept at 80 °C for 20 min, the reaction mixture was neutralized by addition of dil. HCl and extracted with ether. The oil left after evaporation of the solvent from the dried ether solution was distilled in vacuo by Kugel-Rohr apparatus to give I. The resulting product I and 4-methylbenzenesulfonic acid (1.5 eq)

was dissolved in acetonitrile, and then *N*-Chlorosuccinimide (1.0 eq) was added in the mixture slowly. The reaction was allowed to proceed at 80 °C for 1 hour. After the reaction, the mixture is subjected to flash chromatography, and the desired compound II was obtained. The product II was dissolved in THF, and methyl carbazate (1.2 eq) was added into the solution. The compound II was dissolved in THF, and then methyl carbazate (1.2 eq) was added to the solution. The reaction mixture was allowed to react for 4 h at room temperature. The resulting product was dissolved in ethyl acetate after the THF is evaporated. The dissolved product was then subjected to at least three washes with sodium bicarbonate. During the washing process, the extracted part changes color to red, and the extracts was purified by column chromatography on silica gel to furnish the product **2a’**.

For the synthesis of 2-^{13}C-labeled azoalkene (**2a’’**):

NaH (ca. 200 mol %, ca. 60% in mineral oil) was washed with twice with a small amount of hexanes in a two-neck round-bottom flask equipped with a reflux condenser and a magnetic stirring bar. The washed NaH thus obtained was suspended in dry THF (0.6 M) and cooled to 0 °C. Diethyl carbonate (130 mol %) and the corresponding ketone-2-^{13}C (100 mol %) were added successively via syringe. After 10 minutes the reaction mixture was allowed to warm to ambient temperature. Once the reaction mixture had reached ambient temperature it was slowly heated to reflux in an oil bath. After full conversion of the ketone the reaction mixture was cooled to 0 °C in an ice bath. The reaction was quenched with 1M aq. $NaHSO_4$ and diluted with Et_2O. The layers were separated, and the aqueous layer was extracted with Et_2O three times. The combined organic layers were washed successively with sat. aq. $NaHCO_3$ and brine, dried over Na_2SO_4, filtered and concentrated, and the compound III was obtained. The resulting product III and 4-methylbenzenesulfonic acid (1.5 eq) was dissolved in acetonitrile, and then N-Chlorosuccinimide (1eq) was added in the mixture slowly. The reaction was allowed to proceed at 80 °C for 1 hour. After the reaction, the mixture is subjected to flash chromatography, and the desired compound IV was obtained. The product IV was dissolved in THF, and methyl carbazate (1.2 eq) was added into the solution. The compound IV was dissolved in THF, and then methyl carbazate (1.2 eq) was added to the solution. The reaction mixture was allowed to react for 4 h at room temperature. The resulting product was dissolved in ethyl acetate after the THF is evaporated. The dissolved product was then subjected to at least three washes with sodium bicarbonate. During the washing process,

the extracted part changes color to red, and the extracts was purified by column chromatography on silica gel to furnish the product **2a''**.

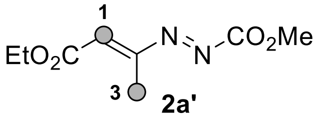

2a'

Methyl (*E*)-2-((*E*)-4-ethoxy-4-oxobut-2-en-2-yl)diazene-1-carboxylate (2a')

A red liquid; totally isolated yield = 52%. ^{1}H NMR (400 MHz, $CDCl_3$) δ 6.97 (dd, *J* = 165.7, 6.0 Hz, 1H), 4.30 (q, *J* = 7.1 Hz, 2H), 4.05 (s, 3H), 2.25 (dd, *J* = 130.5, 4.0 Hz, 3H), 1.35 (t, *J* = 7.1 Hz, 3H). ^{13}C NMR (101 MHz, $CDCl_3$) δ 166.6, 162.8, 142.3, 133.4, 59.8, 55.8, 23.9, 10.5. HRMS (ESI) m/z calcd for $C_6[^{13}C]_2H_{12}N_2NaO_4$ $[M+Na]^+$ = 225.0756, found = 225.0759.

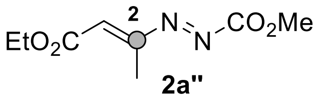

2a''

methyl (E)-2-((E)-4-ethoxy-4-oxobut-2-en-2-yl)diazene-1-carboxylate (2a'')

A red liquid; totally isolated yield = 39%. ^{1}H NMR (400 MHz, $CDCl_3$) δ 6.96 (d, *J* = 5.2 Hz, 1H), 4.29 (q, *J* = 7.2 Hz, 2H), 4.04 (s, 3H), 2.24 (d, *J* = 6.8 Hz, 3H), 1.34 (t, *J* = 7.2 Hz, 3H). ^{13}C NMR (101 MHz, $CDCl_3$) δ 163.2, 162.9, 147.6, 133.8, 61.1, 55.4, 23.7, 11.5. HRMS (ESI) m/z calcd for $C_7[^{13}C]H_{13}N_2O_4$ $[M+H]^+$ = 202.0903, found = 202.0904.

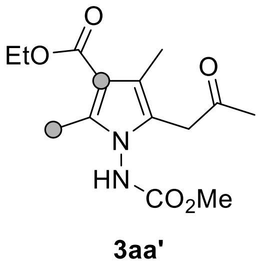

3aa'

Ethyl 1-((methoxycarbonyl)amino)-2,4-dimethyl-5-(2-oxopropyl)-1H-pyrrole-3-carboxylate (3aa')

(GP-1) A light-yellow oil; isolated yield = 92%. ^{1}H NMR (400 MHz, $CDCl_3$) δ 7.82 (s, 1H), 4.24 (q, *J* = 7.1 Hz, 2H), 3.76 (s, 3H), 3.68 (d, *J* = 17.1 Hz, 1H), 3.40 (d, *J* = 17.1 Hz, 1H), 2.53 (d, *J* = 3.0 Hz, 1H), 2.25 – 2.19 (m, 2H), 2.18 – 2.09 (m, 6H), 1.32 (t, *J* = 7.1 Hz, 3H). ^{13}C NMR (101

MHz, $CDCl_3$) δ 206.6, 166.1, 165.3, 155.7, 122.0, 116.3, 114.1, 109.7, 59.3, 53.4, 38.5, 29.3, 14.4, 11.4, 10.8. HRMS (ESI) m/z calcd for $C_{12}[^{13}C]_2H_{21}N_2O_5$ $[M+H]^+$ = 299.1512, found = 299.1515.

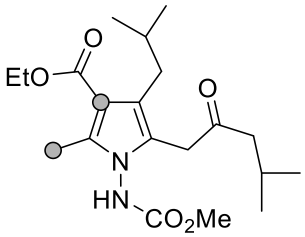

3ae'

Ethyl 4-isobutyl-1-((methoxycarbonyl)amino)-2-methyl-5-(4-methyl-2-oxopentyl)-1H-pyrrole-3-carboxylate (3ae')

(GP-1) A light-yellow oil; isolated yield = 84%. ^1H NMR (400 MHz, $CDCl_3$) δ 7.80 (s, 1H), 4.22 (q, J = 7.1 Hz, 2H), 3.73 (d, J = 12.1 Hz, 4H), 3.33 (d, J = 18.0 Hz, 1H), 2.61 – 2.05 (m, 8H), 1.77 (hept, J = 6.7 Hz, 1H), 1.31 (t, J = 7.1 Hz, 3H), 0.90 (t, J = 6.1 Hz, 6H), 0.85 (d, J = 6.7 Hz, 3H), 0.75 (d, J = 6.6 Hz, 3H). ^{13}C NMR (101 MHz, $CDCl_3$) δ 208.6, 166.0, 165.1, 155.6, 137.5, 122.5, 122.0, 109.0, 59.2, 53.3, 51.3, 38.3, 34.7, 29.6, 24.5, 22.5, 22.4, 22.3, 22.2, 14.3, 10.9. HRMS (ESI) m/z calcd for $C_{18}[^{13}C]_2H_{33}N_2O_5$ $[M+H]^+$ = 383.2451, found = 383.2452.

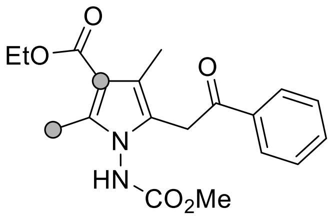

3ai'

Ethyl 1-((methoxycarbonyl)amino)-2,4-dimethyl-5-(2-oxo-2-phenylethyl)-1H-pyrrole-3-carboxylate (3ai')

(GP-2) A light-yellow oil; isolated yield = 65%. ^1H NMR (400 MHz, $CDCl_3$) δ 8.11 – 7.97 (m, 2H), 7.73 (s, 1H), 7.62 (t, J = 7.4 Hz, 1H), 7.50 (t, J = 7.8 Hz, 2H), 4.48 (d, J = 17.8 Hz, 1H), 4.25 (q, J = 7.1 Hz, 2H), 3.84 (d, J = 17.8 Hz, 1H), 3.74 (s, 3H), 2.58 (d, J = 3.1 Hz, 1H), 2.26 (d, J = 3.1 Hz, 1H), 2.18 (d, J = 3.4 Hz, 3H), 1.33 (t, J = 7.1 Hz, 3H). ^{13}C NMR (101 MHz, $CDCl_3$) δ 197.5, 166.2, 165.3, 155.7, 136.1, 133.8, 128.8, 128.4, 121.7, 116.5, 100.8, 77.3, 77.0, 76.7, 59.2, 53.4, 33.3, 14.5, 11.2, 10.9. HRMS (ESI) m/z calcd for $C_{17}[^{13}C]_2H_{23}N_2O_5$ $[M+H]^+$ = 361.1669, found = 361.1670.

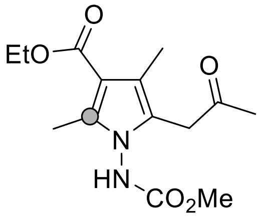

3aa''

Ethyl 1-((methoxycarbonyl)amino)-2,4-dimethyl-5-(2-oxopropyl)-1H-pyrrole-3-carboxylate (3aa'')

(GP-1) A light-yellow oil; isolated yield = 88%. ^{1}H NMR (400 MHz, $CDCl_3$) δ 7.52 (s, 1H), 4.26 (q, *J* = 7.1 Hz, 2H), 3.78 (s, 3H), 3.73 (d, *J* = 17.1 Hz, 1H), 3.40 (d, *J* = 17.1 Hz, 1H), 2.40 (d, *J* = 7.0 Hz, 3H), 2.19 (d, *J* = 2.3 Hz, 6H), 1.33 (t, *J* = 7.1 Hz, 3H). ^{13}C NMR (101 MHz, $CDCl_3$) δ 198.8, 165.6, 156.2, 127.2, 121.9, 116.7, 109.5, 59.3, 53.5, 38.5, 29.5, 14.5,11.4, 10.6. HRMS (ESI) m/z calcd for $C_{13}[^{13}C]H_{21}N_2O_5$ $[M+H]^+$ = 298.1479, found = 298.1480.

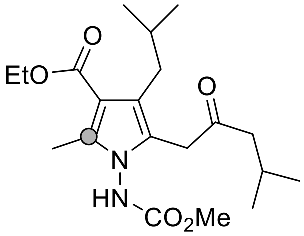

3ae''

Ethyl 4-isobutyl-1-((methoxycarbonyl)amino)-2-methyl-5-(4-methyl-2-oxopentyl)-1H-pyrrole-3-carboxylate (3ae'')

(GP-1) A light-yellow oil; isolated yield = 78%. ^{1}H NMR (400 MHz, $CDCl_3$) δ 7.58 (s, 1H), 4.24 (q, *J* = 7.1 Hz, 2H), 3.85 – 3.69 (m, 4H), 3.32 (d, *J* = 18.0 Hz, 1H), 2.56 (dd, *J* = 13.5, 6.4 Hz, 1H), 2.49 – 2.35 (m, 5H), 2.30 (dt, *J* = 13.7, 6.4 Hz, 1H), 2.14 (dt, *J* = 13.4, 6.7 Hz, 1H), 1.81 – 1.75 (m, 1H), 1.33 (t, *J* = 7.1 Hz, 3H), 0.92 (t, *J* = 6.4 Hz, 6H), 0.87 (d, *J* = 6.6 Hz, 3H), 0.77 (d, *J* = 6.6 Hz, 3H). ^{13}C NMR (101 MHz, $CDCl_3$) δ 199.2, 165.6, 156.8, 138.1, 122.0, 121.0, 109.3, 59.2, 53.4, 51.9, 38.3, 34.8, 29.7, 24.6, 22.6, 22.4, 22.4, 22.3, 14.4, 11.3. HRMS (ESI) m/z calcd for $C_{19}[^{13}C]H_{33}N_2O_5$ $[M+H]^+$ = 382.2418, found = 382.2420.

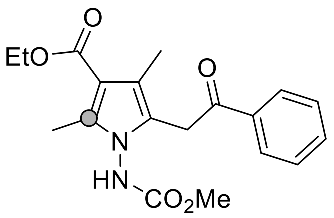

3ai"

Ethyl 1-((methoxycarbonyl)amino)-2,4-dimethyl-5-(2-oxo-2-phenylethyl)-1H-pyrrole-3-carboxylate (3ai'')

(GP-2) A light-yellow oil; isolated yield = 67%. ^{1}H NMR (400 MHz, $CDCl_3$) δ 8.02 (d, J = 7.1 Hz, 2H), 7.63 (t, J = 7.5 Hz, 1H), 7.51 (t, J = 7.7 Hz, 2H), 4.51 (d, J = 17.7 Hz, 1H), 4.26 (q, J = 7.1 Hz, 2H), 3.83 (d, J = 17.8 Hz, 1H), 3.75 (s, 3H), 2.44 (d, J = 6.9 Hz, 3H), 2.19 (s, 3H), 1.33 (t, J = 7.1 Hz, 3H). ^{13}C NMR (101 MHz, $CDCl_3$) δ 199.7, 158.3, 153.6, 139.3, 138.5, 137.3, 134.5, 133.4, 129.5, 128.9, 128.4, 66.5, 60.8, 33.4 14.1, 12.3, 10.8. HRMS (ESI) m/z calcd for $C_{18}[^{13}C]H_{23}N_2O_5$ $[M+H]^+$ = 360.1635, found = 360.1636.

5. Mechanistic studies

(1) ^{18}O labelling experiment for the reaction of 1a and 2a.

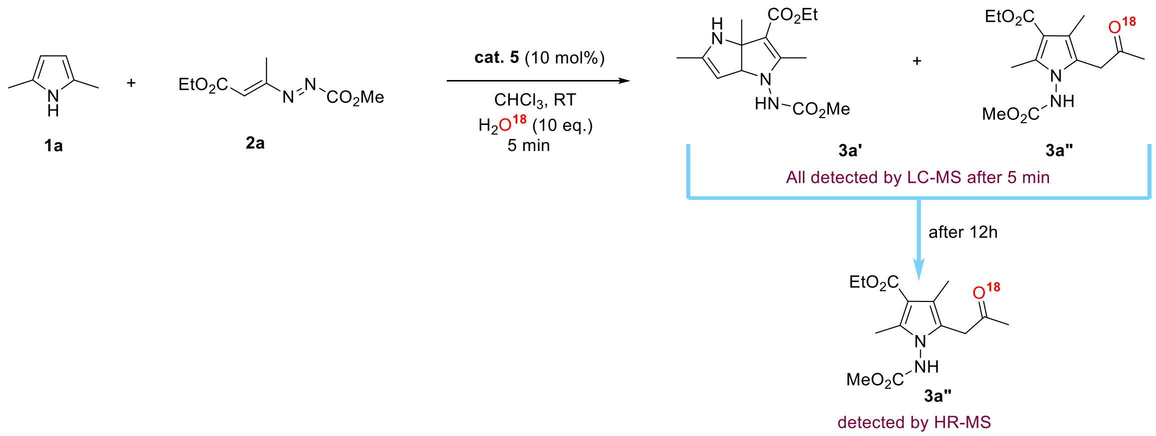

To an 8 mL screw-cap vial equipped with a magnetic stir bar was charged with simple pyrrole **1a** (0.2 mmol, 20.3 μL), azoalkene **2a** (0.24 mmol, 48.0 mg), H_2O^{18} (2 mmol) and cat. **5** (10 mol%) in dry $CHCl_3$ (2 mL) at room temperature. The reaction mixture was detected by LC-MS and HRMS in 5min and 12 h respectively.

5 min LC-MS spectrum:

LC spectrum

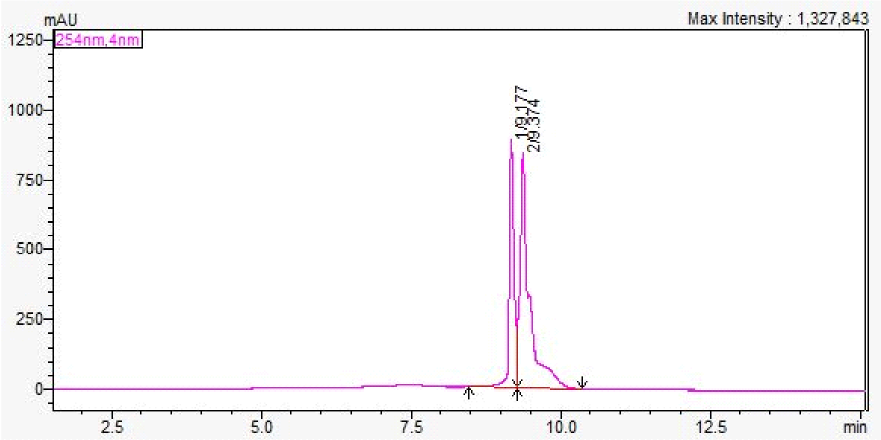

9.177 min MS spectrum

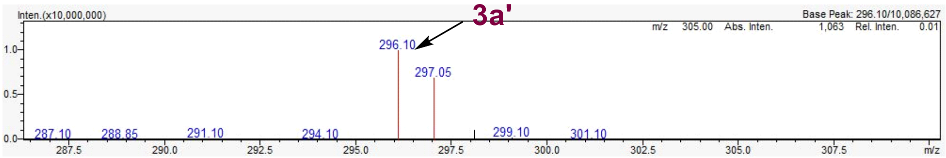

9.374 min MS spectrum

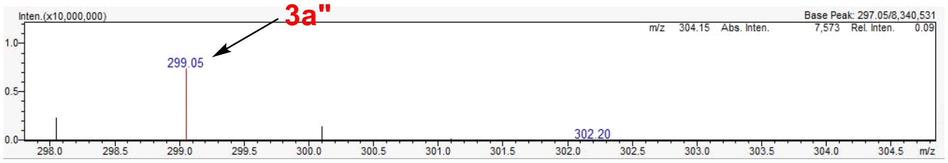

The intermediate of **3a'** (9.177 min MS spectrum) and desired product **3a''** (9.374 min MS spectrum) were all detected by LC-MS.

12 h HR-MS spectrum:

Meas. m/z	#	Formula	Calc. Mass	Err [ppm]
299.1488	1	C14 H21 N2 O4 [18O]	299.1487	0.33

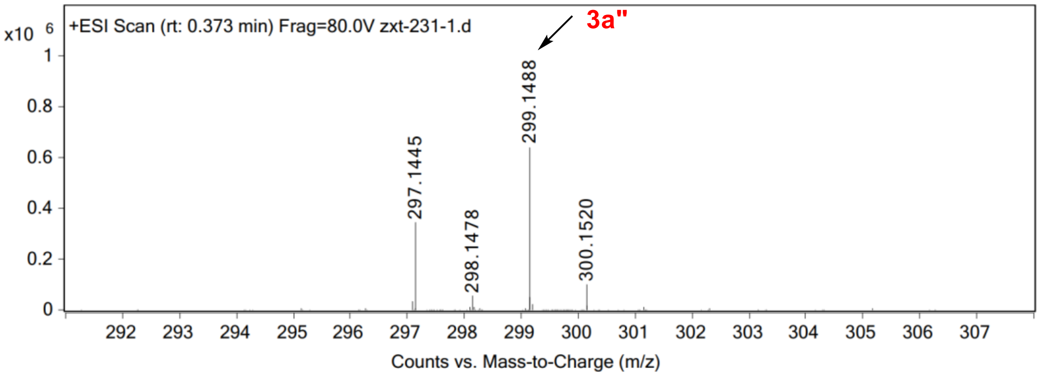

(2) ^{15}N labelling experiment.

a. synthesis of ^{15}N labelled pyrrole **1d'**

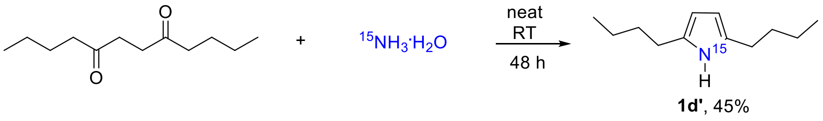

To a 25 mL round-bottom flask equipped with a magnetic stir bar was charged with 1,4-diketone (2 mmol) and $^{15}NH_3 \cdot H_2O$ (20 mmol) at room temperature. The reaction mixture was concentrated under reduced pressure after 48 h and the residue was purified by column chromatography on silica gel to furnish the product.

A light-yellow liquid; isolated yield = 45%. ^{1}H NMR (400 MHz, $CDCl_3$) δ 7.78 – 7.40 (m, 1H), 5.78 (dd, J = 4.9, 2.7 Hz, 2H), 2.56 (td, J = 7.8, 2.4 Hz, 4H), 1.65 – 1.54 (m, 4H), 1.45 – 1.32 (m, 4H), 0.93 (t, J = 7.3 Hz, 6H). ^{13}C NMR (101 MHz, $CDCl_3$) δ 131.2 (*^{15}N-C*, d, J = 12.8 Hz), 104.6 (*^{15}N-C*, d, J = 5.1 Hz), 31.8, 27.5 (*^{15}N-C*, d, J = 2.0 Hz), 22.5, 13.9.

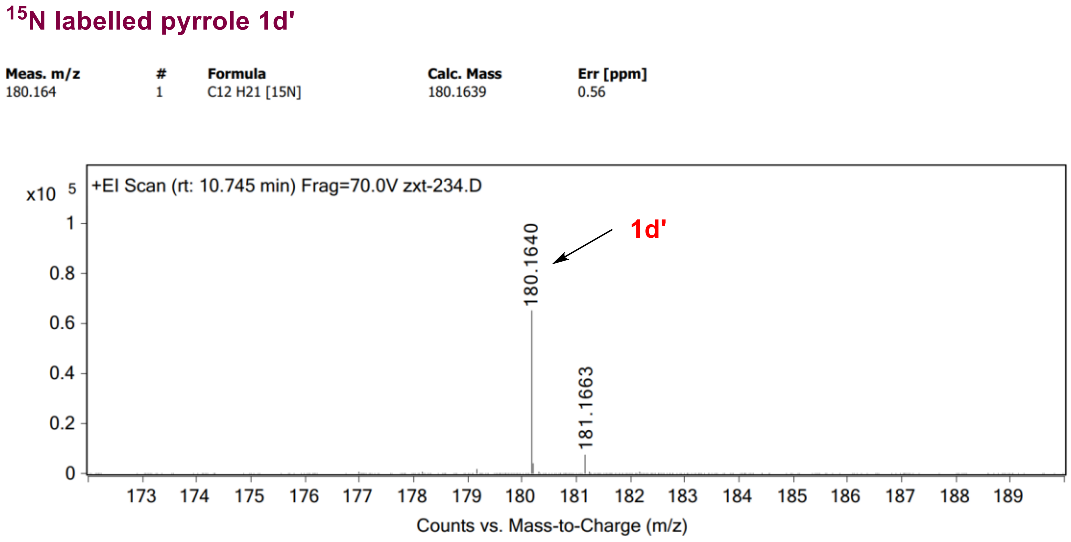

b. ^{15}N labelling experiment of the reaction **1d'** with **2a**.

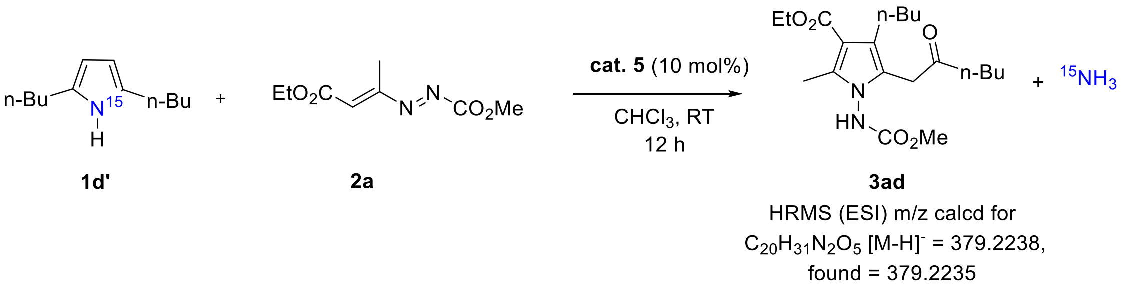

To an 8 mL screw-cap vial equipped with a magnetic stir bar was charged with simple pyrrole **1d'** (0.2 mmol, 36.0 mg), azoalkene **2a** (0.24 mmol, 48.0 mg) and cat. **5** (10 mol%) in $CHCl_3$ (2 mL)

at room temperature. After 12 h, the corresponding ^{15}N labelled pyrrole **3ad** was not detected by HRMS.

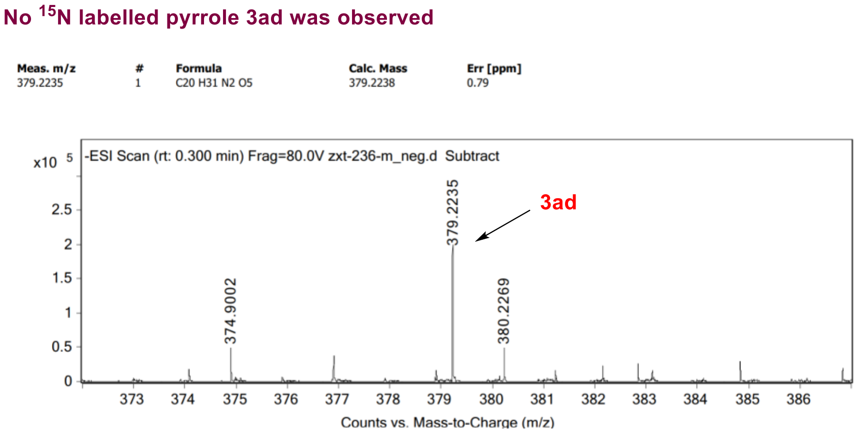

6. Synthetic applications.

(1) Asymmetric organocatalytic synthesis of N-N axially chiral compounds

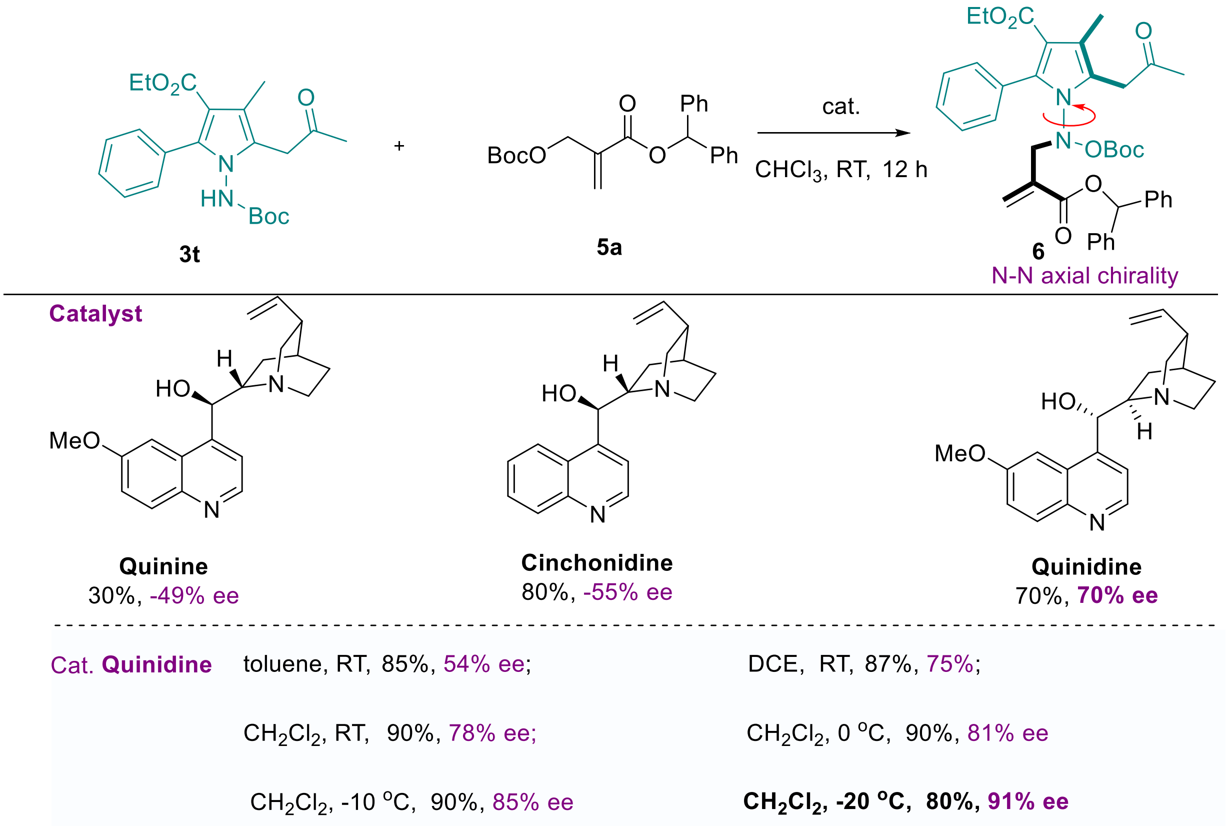

To a stirring anhydrous DCM solution (2 ml) of substrate **3o** (0.1 mmol) and MBH-adduct **5a** (0.16 mmol) was added quinidine (10 mol%) at -20 °C. The reaction mixture was stirred until completion

of reaction (12 h, as monitored by TLC). The solvent was then removed under reduced pressure and the residue was purified by flash column chromatography on silica gel to afford desired products **6**.

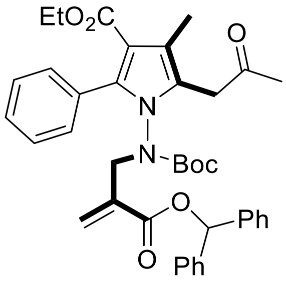

6

Ethyl 1-((2-((benzhydryloxy)carbonyl)allyl)(tert-butoxycarbonyl)amino)-4-methyl-5-(2-oxopropyl)-2-phenyl-1H-pyrrole-3-carboxylate (6)

A light-yellow oil; isolated yield = 80%. ^{1}H NMR (400 MHz, $CDCl_3$) δ 7.32 (tt, *J* = 10.4, 6.2 Hz, 15H), 6.85 (s, 1H), 6.27 (d, *J* = 5.0 Hz, 1H), 5.55 – 5.40 (m, 1H), 4.52 – 4.16 (m, 1H), 4.16 – 3.96 (m, 2H), 3.76 (q, *J* = 14.9 Hz, 1H), 3.42 – 3.11 (m, 2H), 2.23 – 1.94 (m, 6H), 1.44 (d, *J* = 7.8 Hz, 9H), 1.06 – 0.96 (m, 3H). ^{13}C NMR (101 MHz, $CDCl_3$) δ 205.2, 203.3, 164.9, 164.3, 164.3, 154.5, 154.1, 140.0, 140.0, 137.6, 134.2, 133.7, 128.6, 128.5, 128.4, 128.2, 128.0, 127.8, 127.7, 127.1, 127.0, 127.0, 124.4, 124.1, 117.7, 117.4, 111.7, 83.2, 83.0, 59.2, 51.9, 50.7, 39.3, 38.7, 29.0, 28.4, 28.0, 27.9, 13.8, 11.4, 11.4. HRMS (ESI) m/z calcd for $C_{39}H_{42}N_2NaO_7$ $[M+Na]^+$ = 673.2884, found = 673.2889; the ee value was 91%, t_R (major) = 13.0 min, t_R (minor) = 16.0 min (Chiralpak AD-H, λ = 254 nm, 10% *i*-PrOH/Hexane, flow rate = 0.7 mL/min).

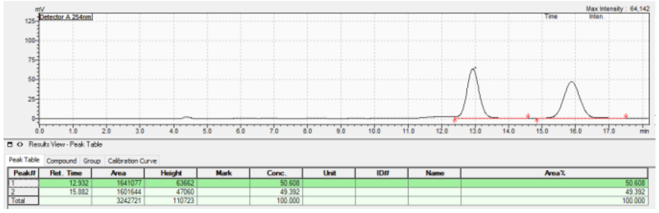

Peak#	Ret. Time	Area	Height	Mark	Conc.	Unit	ID#	Name	Area%
1	12.932	1641077	63662		50.608				50.608
2	15.882	1601644	47060		49.392				49.392
Total		3242721	110723		100.000				100.000

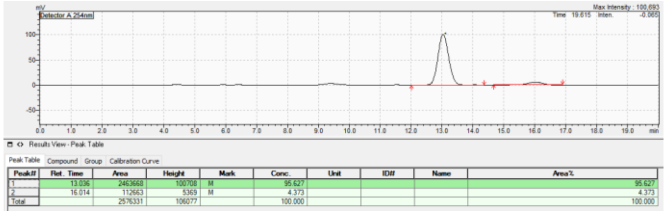

Peak#	Ret. Time	Area	Height	Mark	Conc.	Unit	ID#	Name	Area%
1	13.036	2463668	100708	M	95.627				95.627
2	16.014	112663	5369	M	4.373				4.373
Total		2576331	106077		100.000				100.000

(2) The cleavage of N-N bond

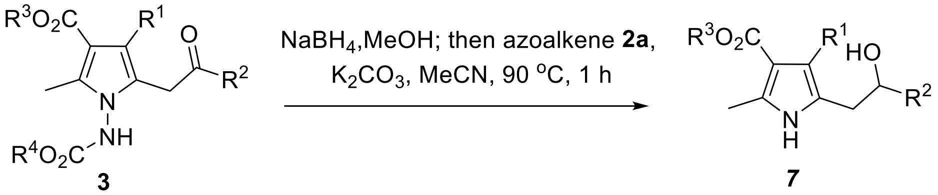

To a 25 mL round-bottom flask equipped with a magnetic stir bar was charged with **3** (2.0 mmol) in MeOH (10 mL) at 0 °C. Sodium borohydride was dropwise added into the reaction mixture. The reaction mixture was concentrated under reduced pressure after 1 h and the residue was purified by column chromatography on silica gel to furnish the product. The resulting product (1.0 mmol) and **2a** (1.5 mmol) were added to 10 mL CH_3CN in a 25 mL round-bottom flask, and the mixture was refluxed for 8 h. The reaction mixture was concentrated under reduced pressure and the residue was purified by column chromatography on silica gel to furnish the product **7** [10,11].

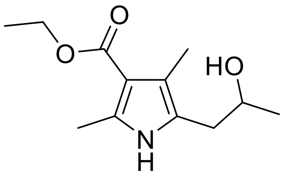

7a

Ethyl 5-(2-hydroxypropyl)-2,4-dimethyl-1H-pyrrole-3-carboxylate (7a)

A light-yellow oi; isolated yield = 73%. ^{1}H NMR (400 MHz, $CDCl_3$) δ 8.63 (s, 1H), 4.24 (q, *J* = 7.1 Hz, 2H), 3.99 (dtd, *J* = 12.1, 6.1, 3.6 Hz, 1H), 2.69 (dd, *J* = 15.2, 3.6 Hz, 1H), 2.52 (dd, *J* = 15.2, 7.7 Hz, 1H), 2.44 (s, 3H), 2.15 (s, 3H), 2.01 (s, 1H), 1.33 (t, *J* = 7.1 Hz, 3H), 1.21 (d, *J* = 6.2 Hz, 3H). ^{13}C NMR (101 MHz, $CDCl_3$) δ 166.6, 134.3, 123.8, 117.1, 110.4, 68.2, 59.0, 33.9, 23.1, 14.5, 13.9, 11.0. HRMS (ESI) m/z calcd for $C_{12}H_{20}NO_3$ $[M+H]^+$ = 226.1438, found = 226.1439.

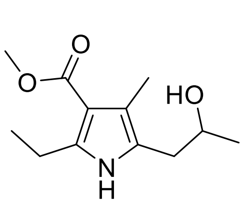

7b

Methyl 2-ethyl-5-(2-hydroxypropyl)-4-methyl-1H-pyrrole-3-carboxylate (7b)

A light-yellow oi; isolated yield = 83%. ^{1}H NMR (400 MHz, $CDCl_3$) δ 8.56 (s, 1H), 4.09 – 3.98 (m, 1H), 3.78 (s, 3H), 2.90 (q, *J* = 7.5 Hz, 2H), 2.80 – 2.46 (m, 2H), 2.15 (s, 3H), 1.25 – 1.21 (m, 3H), 1.19 (d, *J* = 7.5 Hz, 3H). ^{13}C NMR (101 MHz, $CDCl_3$) δ 166.7, 140.2, 123.8, 117.1, 109.4, 68.3, 50.3, 33.8, 23.2, 21.1, 13.5, 11.0. HRMS (ESI) m/z calcd for $C_{12}H_{18}NO_3$ [M-H]$^-$ = 224.1292, found = 224.1291.

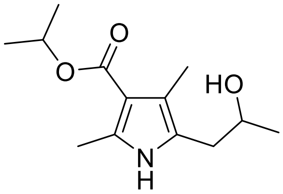

7c

Isopropyl 5-(2-hydroxypropyl)-2,4-dimethyl-1H-pyrrole-3-carboxylate (7c)

A light-yellow oi; isolated yield = 93%. ^{1}H NMR (400 MHz, $CDCl_3$) δ 8.39 (s, 1H), 5.17 (p, *J* = 6.3 Hz, 1H), 4.02 (ddd, *J* = 7.7, 6.0, 3.3 Hz, 1H), 2.71 (dd, *J* = 15.2, 3.5 Hz, 1H), 2.53 (dd, *J* = 15.3, 7.8 Hz, 1H), 2.46 (s, 3H), 2.16 (s, 3H), 1.31 (d, *J* = 6.2 Hz, 6H), 1.24 (d, *J* = 6.2 Hz, 3H). ^{13}C NMR (101 MHz, $CDCl_3$) δ 166.3, 134.1, 122.8, 118.5, 111.4, 67.8, 66.0, 33.8, 29.7, 23.3, 22.3, 14.0, 11.0. HRMS (ESI) m/z calcd for $C_{13}H_{20}NO_3$ [M-H]$^-$ = 238.1449, found = 238.1448.

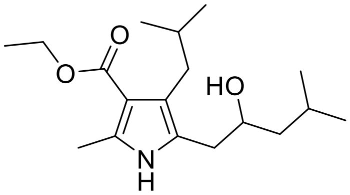

7d

Ethyl 5-(2-hydroxy-4-methylpentyl)-4-isobutyl-2-methyl-1H-pyrrole-3-carboxylate (7d)

A light-yellow oi; isolated yield = 72%. ^{1}H NMR (400 MHz, $CDCl_3$) δ 8.66 (s, 1H), 4.24 (q, *J* = 7.1 Hz, 2H), 3.90 (tdd, *J* = 8.2, 4.7, 2.7 Hz, 1H), 2.72 (dd, *J* = 15.4, 2.7 Hz, 1H), 2.53 – 2.47 (m, 1H), 2.46 (s, 3H), 2.45 – 2.35 (m, 2H), 1.79 – 1.73 (m, 1H), 1.45 (ddd, *J* = 14.1, 8.4, 5.8 Hz, 1H), 1.36 – 1.26 (m, 5H), 0.93 (dd, *J* = 7.7, 6.6 Hz, 6H), 0.86 (dd, *J* = 10.2, 6.7 Hz, 6H). ^{13}C NMR (101 MHz, $CDCl_3$) δ 166.4, 134.6, 124.9, 120.9, 109.9, 70.6, 58.9, 46.6, 34.4, 32.6, 30.1, 24.7, 23.2,

22.6, 22.4, 22.1, 14.5, 14.2. HRMS (ESI) m/z calcd for $C_{18}H_{30}NO_3$ $[M-H]^-$ = 308.2231, found = 308.2229.

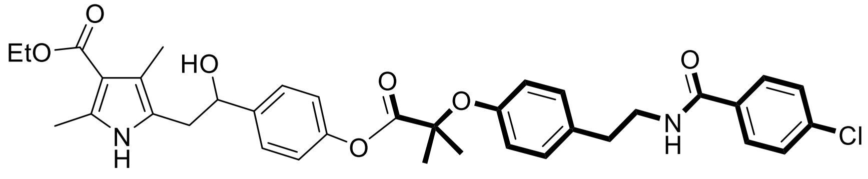

7e

Ethyl 5-(2-(4-((2-(4-(2-(4-chlorobenzamido)ethyl)phenoxy)-2-methylpropanoyl)oxy)phenyl)-2-hydroxyethyl)-2,4-dimethyl-1H-pyrrole-3-carboxylate

A light-yellow oi; isolated yield = 65%. ^{1}H NMR (400 MHz, $CDCl_3$) δ 8.36 (s, 1H), 7.60 (d, *J* = 8.4 Hz, 2H), 7.36 (d, *J* = 8.4 Hz, 2H), 7.29 (d, *J* = 8.4 Hz, 2H), 7.23 (s, 1H), 7.14 (d, *J* = 8.4 Hz, 2H), 7.01 – 6.87 (m, 3H), 6.08 (s, 1H), 4.85 (dd, *J* = 8.1, 3.7 Hz, 1H), 4.25 (q, *J* = 7.1 Hz, 2H), 3.88 – 3.61 (m, 4H), 2.96 – 2.86 (m, 2H), 2.45 (s, 3H), 2.06 (d, *J* = 13.0 Hz, 3H), 1.75 (s, 6H), 1.33 (t, *J* = 7.1 Hz, 3H). ^{13}C NMR (101 MHz, $CDCl_3$) δ 176.6, 173.7, 169.3, 166.6, 152.8, 151.2, 145.9, 144.5, 142.8, 141.7, 140.7, 139.5, 139.0, 138.1, 135.9, 135.6, 131.9, 131.3, 128.8, 128.2, 126.8, 124.1, 123.9, 121.3, 119.4, 117.1, 115.6, 112.7, 80.8, 74.0, 60.1. $C_{36}H_{39}ClN_2O_7$ $[M-H]^-$ = 645.2373, found = 645.2372.

(3) Synthesis of Sutent®

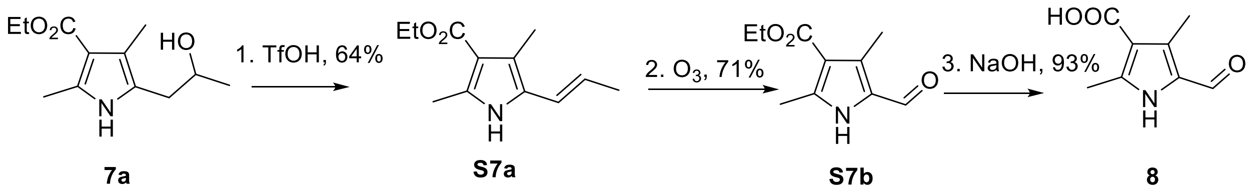

To a 25 mL round-bottom flask equipped with a magnetic stir bar was charged with **7a** (1.0 mmol, 225 mg) in DCM (5 mL). The TfOH (2.0 mmol, 300 mg) was added dropwise into the reaction mixture. The reaction mixture was concentrated under reduced pressure after 12 h and the residue was purified by column chromatography on silica gel to furnish the product **S7a** (132 mg, 64%). The resulting product **S7a** (0.5 mmol, 104 mg) was added in a 25 mL round-bottom flask in 5 mL DCM at -78 ºC, and the reaction system is fed with ozone through the ozone generator. After 30

min, 0.3 mL Me_2S was added into the mixture. The reaction mixture was concentrated under reduced pressure and the residue was purified by column chromatography on silica gel to furnish the product (69 mg, 71%). Compound **S7b** (195 mg, 1.0 mmol) was added into a flask equipped with a stirrer, a thermometer, and a condenser; absolute ethanol (3.0 mL) was added into the flask until partially dissolving. Aqueous solution (6.0 mL) of sodium hydroxide (80 mg, 2.0 mmol) was added under stirring, being kept at 60 ^oC for 12 h. The reaction was then cooled down to RT. The reaction mixture was poured into ice water (20 mL) to form an aqueous solution. A small amount of insoluble substance was filtered under vacuum and was washed with water (20 mL). The combined aqueous layers were adjusted with hydrochloride (2 mol/L) to pH 4 to afford some white precipitates, which were collected via filtering and washing with ice-water to provide **8** (155 mg, 93%).

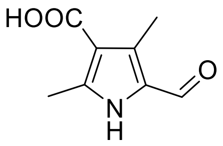

8

5-Formyl-2,4-dimethyl-1H-pyrrole-3-carboxylic acid (8)

A light-yellow solid. ^1H NMR (400 MHz, DMSO) δ 12.09 (s, 2H), 9.60 (s, 1H), 2.45 (s, 3H), 2.41 (s, 3H). ^{13}C NMR (101 MHz, DMSO) δ 177.8, 166.0, 142.8, 134.1, 128.2, 113.3, 13.6, 10.4. HRMS (ESI) m/z calcd for $C_8H_{10}NO_3$ $[M+H]^+$ = 168.0655, found = 168.0654.

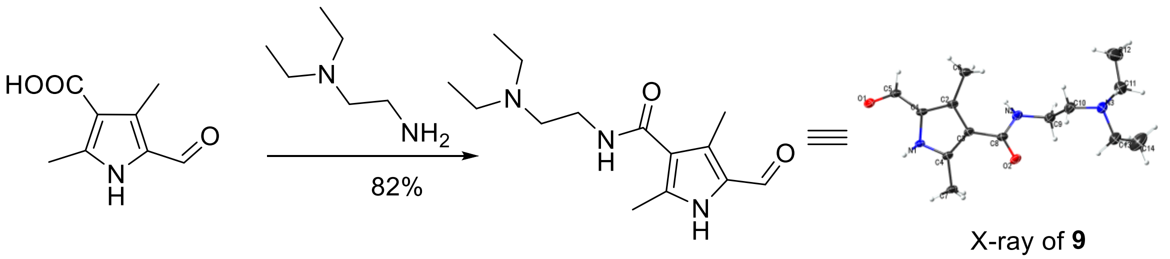

8 **9** X-ray of **9** CCDC 2201648

To a 25 mL round-bottom flask, **8** (55 mg, 0.33 mmol) and anhydrous DMF (2 mL) were added while being cooled down to 0 ^oC with an ice bath. To the above mixture was added dropwise a solution of dicyclohexylcarbodiimide (DCC, 101 mg, 0.49 mmol) in dichloromethane (1 mL) under stirring while keeping the reaction temperature at 0 ^oC, to which were then added at r.t. 4-dimethylaminopyridine (DMAP, 165 mg) and *N* ,*N*-diethylethane-1,2-diamine (50 μL, 0.4 mmol).

The reaction mixture was concentrated under reduced pressure after 48 h and the residue was purified by column chromatography on silica gel to furnish the product **9** (71.7 mg, 82%).

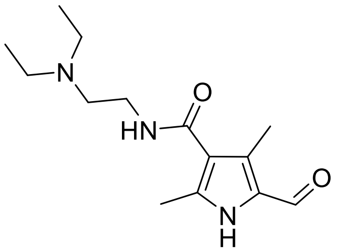

9

A light-yellow solid. ^{1}H NMR (400 MHz, $CDCl_3$) δ 10.62 (s, 1H), 9.53 (s, 1H), 6.46 (s, 1H), 3.44 (q, *J* = 5.6 Hz, 2H), 2.68 – 2.43 (m, 12H), 0.99 (t, *J* = 7.2 Hz, 6H). ^{13}C NMR (101 MHz, $CDCl_3$) δ 177.0, 165.0, 140.3, 132.2, 128.0, 118.9, 51.2, 46.3, 36.6, 13.5, 11.7, 10.2. HRMS (ESI) m/z calcd for $C_{14}H_{24}N_3O_2$ $[M+H]^+$ = 266.1863, found = 266.1856.

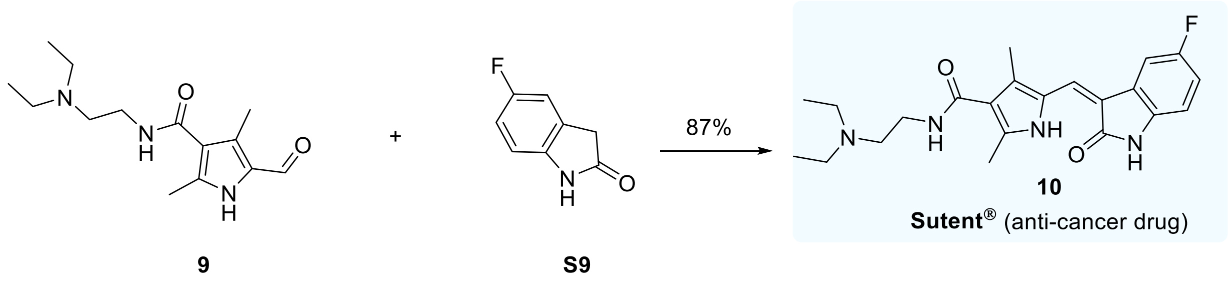

To a flask equipped with a condenser were added **9** (26.5 mg, 0.1 mmol), **S9** (18 mg, 0.12 mmol), and toluene (2.0 mL). The mixture was stirred to be fully dissolved before addition of a drop of piperidine with stirring. The mixture was heated to reflux for 3 h before cooling down with an ice-bath. The precipitates were collected by filtration. The filter cake was washed with hexane and dried under vacuum to give the free base of the desired target molecule **10** as orange–yellow solid (34.7 mg, 87%)

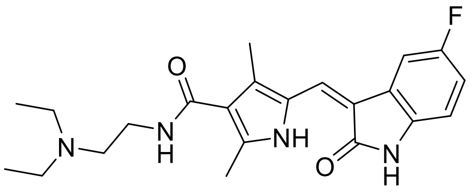

10

An orange-yellow solid. ^{1}H NMR (400 MHz, $CDCl_3$) δ 13.30 (s, 1H), 8.96 (s, 1H), 7.21 (s, 1H), 7.13 (dd, J = 8.7, 2.4 Hz, 1H), 6.91 – 6.64 (m, 3H), 3.54 (q, J = 5.0 Hz, 2H), 2.78 – 2.59 (m, 6H), 2.54 (s, 3H), 2.31 (s, 3H), 1.06 (t, J = 7.1 Hz, 6H). ^{13}C NMR (101 MHz, $CDCl_3$) δ 170.1, 165.7, 160.2, 157.9, 138.2, 133.4, 130.4, 127.5, 127.4, 126.2, 123.9, 119.9, 114.0, 114.0, 112.7, 112.5, 109.9, 109.8, 105.0, 104.8, 51.4, 46.2, 36.6, 14.1, 11.2, 11.0. ^{19}F NMR (377 MHz, $CDCl_3$) δ -121.6. HRMS (ESI) m/z calcd for $C_{22}H_{28}FN_4O_2$ $[M+H]^+$ = 399.2191, found = 399.2195.

(4) Skeletal recasting of other heterocycles.

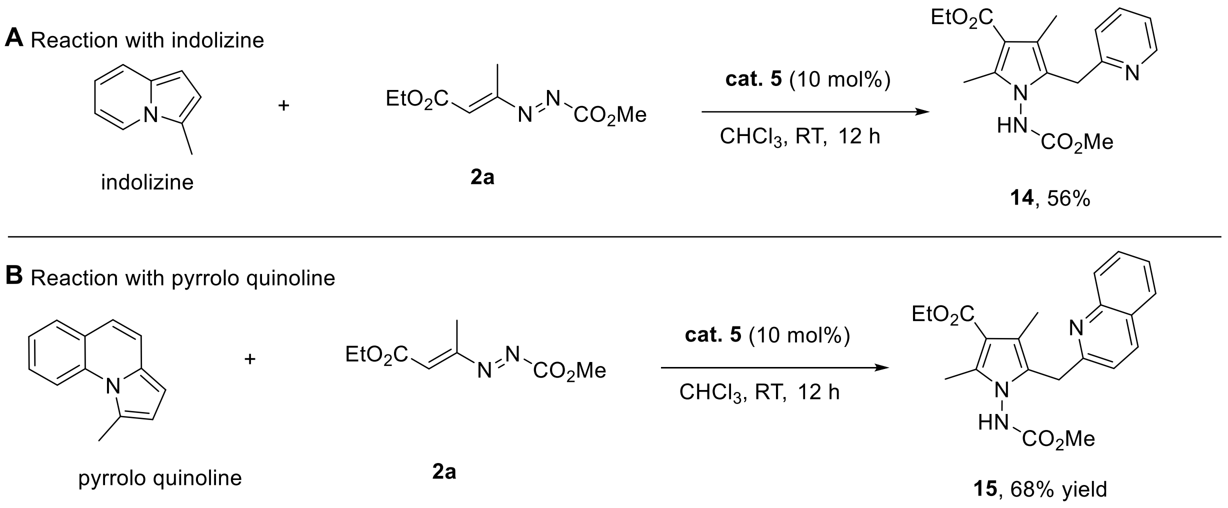

To an 8 mL screw-cap vial equipped with a magnetic stir bar was charged with simple pyrrole **1o** or **1p** (0.2 mmol), azoalkene **2a** (0.24 mmol, 48.0 mg) and cat. **5** (10 mol%) in $CHCl_3$ (2 mL) at room temperature. The reaction mixture was concentrated under reduced pressure after 12 h and the residue was purified by column chromatography on silica gel to furnish the product.

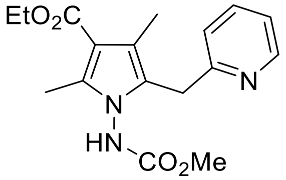

14

Ethyl 1-((methoxycarbonyl)amino)-2,4-dimethyl-5-(pyridin-2-ylmethyl)-1H-pyrrole-3-carboxylate (14)

A light-yellow oil; isolated yield = 56%. ^{1}H NMR (400 MHz, $CDCl_3$) δ 10.09 (s, 1H), 8.46 (t, *J* = 2.8 Hz, 1H), 7.63 (td, *J* = 7.7, 1.8 Hz, 1H), 7.23 – 7.03 (m, 2H), 4.23 (q, *J* = 7.1 Hz, 2H), 4.01 – 3.65 (m, 5H), 2.43 (s, 3H), 2.22 (s, 3H), 1.31 (t, *J* = 7.1 Hz, 3H). ^{13}C NMR (101 MHz, $CDCl_3$) δ 165.9, 159.3, 156.7, 149.1, 137.6, 136.8, 125.1, 122.7, 121.8, 115.4, 109.0, 59.1, 53.3, 31.7, 14.5, 11.3, 11.0. HRMS (ESI) m/z calcd for $C_{17}H_{22}N_3O_4$ $[M+H]^+$ = 332.1605, found = 332.1606.

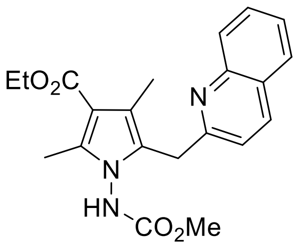

15

Ethyl 1-((methoxycarbonyl)amino)-2,4-dimethyl-5-(quinolin-2-ylmethyl)-1H-pyrrole-3-carboxylate (15)

A light-yellow oil; isolated yield = 68%. ^{1}H NMR (400 MHz, $CDCl_3$) δ 10.28 (s, 1H), 8.01 (d, *J* = 8.4 Hz, 1H), 7.90 (dt, *J* = 8.5, 1.0 Hz, 1H), 7.72 (d, *J* = 8.2 Hz, 1H), 7.66 (ddd, *J* = 8.5, 7.0, 1.5 Hz, 1H), 7.47 (ddd, *J* = 8.1, 6.9, 1.2 Hz, 1H), 7.22 (d, *J* = 8.4 Hz, 1H), 4.24 (q, *J* = 7.1 Hz, 2H), 4.19 – 4.02 (m, 2H), 3.63 (s, 3H), 2.45 (s, 3H), 2.29 (s, 3H), 1.32 (t, *J* = 7.1 Hz, 3H). ^{13}C NMR (101 MHz, $CDCl_3$) δ 165.9, 159.7, 156.4, 147.1, 137.4, 137.0, 129.8, 128.1, 127.5, 126.8, 126.2, 121.0, 115.7, 109.1, 59.1, 53.1, 32.4, 14.4, 11.4, 11.0. HRMS (ESI) m/z calcd for $C_{21}H_{24}N_3O_4$ $[M+H]^+$ = 382.1761, found = 382.1762.

7. Single Crystal Structure X-ray Analysis of 9

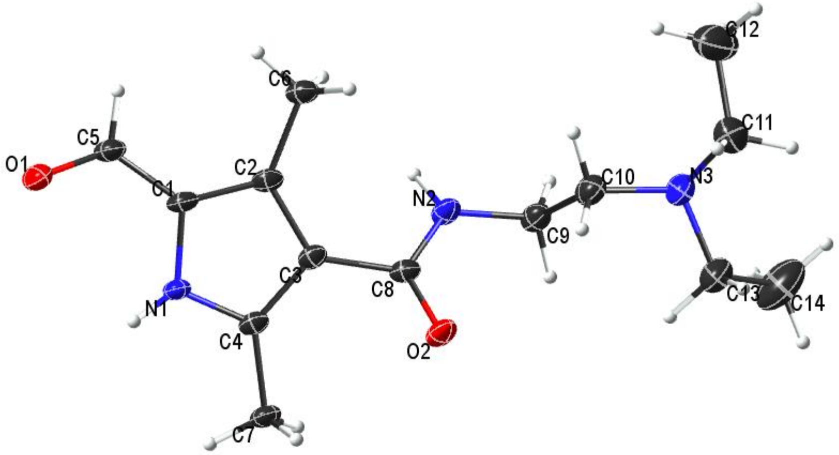

Single Crystal Structure X-ray Analysis

Sample Code:	M257
Sample ID:	XT-666
Student/Researcher:	Zhou Xueting
Supervisor:	Lu Yixin
**CCDC:**	**2201648**

Date: 16-08-2022

Note: The crystal is Triclinic, space group P-1. The asymmetric unit contains four molecules of the compound $C_{14}H_{23}N_3O2$. One ethyl group of a molecule and one $CH_2N(CH_2CH_3)_2$ group of another molecule were disordered into two positions with occupancy ratios=80:20 and 68:32 respectively. Restraints in bond lengths and thermal parameters were applied to the disordered atoms.

Final R values are R^1=0.0652 and wR^2=0.1772 for 2- theta up to 133º.

Table S1. Crystal data and structure refinement for M257.

Identification code	M257	
Empirical formula	$C_{14}H_{23}N_3O_2$	
Formula weight	265.35	
Temperature	100(2) K	
Wavelength	1.54178 Å	
Crystal system	Triclinic	
Space group	P-1	
Unit cell dimensions	a = 10.0203(3) Å	α= 71.995(2)°.
	b = 14.2291(5) Å	β= 86.107(2)°.
	c = 21.7415(8) Å	γ = 87.540(2)°.
Volume	2940.51(18) $Å^3$	
Z	8	
Density (calculated)	1.199 Mg/m^3	
Absorption coefficient	0.653 mm^{-1}	
F(000)	1152	
Crystal size	0.402 x 0.236 x 0.086 mm^3	
Theta range for data collection	3.267 to 66.594°.	
Index ranges	-11<=h<=11, -16<=k<=16, -25<=l<=25	
Reflections collected	36055	
Independent reflections	10196 [R(int) = 0.0375]	
Completeness to theta = 66.594°	98.3 %	
Absorption correction	Semi-empirical from equivalents	
Max. and min. transmission	0.7533 and 0.6110	
Refinement method	Full-matrix least-squares on F^2	
Data / restraints / parameters	10196 / 252 / 802	
Goodness-of-fit on F^2	1.048	
Final R indices [I>2sigma(I)]	R1 = 0.0652, wR2 = 0.1720	
R indices (all data)	R1 = 0.0742, wR2 = 0.1772	
Extinction coefficient	n/a	
Largest diff. peak and hole	0.463 and -0.388 e.$Å^{-3}$	

Table S2. Atomic coordinates (x 10^4) and equivalent isotropic displacement parameters (Å^{2}x 10^3) for M257. U(eq) is defined as one third of the trace of the orthogonalized U^{ij} tensor.

	x	y	z	U(eq)
O(1)	3954(2)	4566(1)	4500(1)	28(1)
O(2)	1622(1)	9237(1)	2937(1)	25(1)
O(3)	1031(2)	5504(1)	5409(1)	26(1)
O(4)	3435(1)	842(1)	6928(1)	23(1)
O(5)	6263(2)	10649(1)	5410(1)	32(1)
O(6)	4962(1)	6030(1)	7128(1)	28(1)
O(7)	1276(2)	10561(1)	5459(1)	33(1)
O(8)	-47(1)	5924(1)	7151(1)	26(1)
N(1)	2592(2)	6447(1)	4255(1)	19(1)
N(2)	3863(2)	9317(1)	2712(1)	22(1)
N(3)	3483(2)	11462(1)	1205(1)	31(1)
N(4)	2432(2)	3634(1)	5659(1)	19(1)
N(5)	1244(2)	764(1)	7246(1)	22(1)
N(7)	7153(2)	8622(1)	5719(1)	20(1)
N(8)	7188(2)	5725(1)	7094(1)	22(1)
N(9)	7088(2)	3600(2)	8638(1)	41(1)
N(10)	2127(2)	8525(1)	5741(1)	20(1)
N(11)	2185(2)	5627(2)	7123(1)	29(1)
C(1)	3644(2)	6225(2)	3867(1)	20(1)
C(2)	3891(2)	7076(2)	3349(1)	19(1)
C(3)	2963(2)	7814(2)	3434(1)	19(1)
C(4)	2177(2)	7391(2)	4004(1)	19(1)
C(5)	4274(2)	5271(2)	4026(1)	23(1)
C(6)	4941(2)	7169(2)	2809(1)	24(1)
C(7)	1096(2)	7837(2)	4339(1)	23(1)
C(8)	2753(2)	8846(2)	3011(1)	20(1)
C(9)	3823(2)	10337(2)	2295(1)	25(1)
C(10)	3385(2)	10436(2)	1621(1)	29(1)
C(11)	3564(3)	11538(2)	512(1)	38(1)
C(12)	4896(3)	11187(3)	296(2)	55(1)
C(13)	2383(3)	12095(2)	1348(1)	38(1)
C(14)	2819(4)	13118(2)	1270(2)	62(1)

C(15)	1390(2)	3855(2)	6049(1)	20(1)
C(16)	1151(2)	3004(2)	6566(1)	19(1)
C(17)	2072(2)	2266(2)	6474(1)	19(1)
C(18)	2856(2)	2692(2)	5905(1)	18(1)
C(19)	742(2)	4802(2)	5889(1)	22(1)
C(20)	126(2)	2914(2)	7115(1)	23(1)
C(21)	3950(2)	2260(2)	5569(1)	22(1)
C(22)	2298(2)	1242(2)	6895(1)	20(1)
C(23)	1412(2)	-216(2)	7709(1)	25(1)
C(24)	2088(2)	-183(2)	8310(1)	27(1)
N(6)	1221(2)	262(2)	8721(1)	28(1)
C(25)	1967(3)	724(2)	9101(1)	36(1)
C(26)	2519(4)	1714(3)	8697(2)	59(1)
C(27)	237(3)	-437(2)	9124(1)	35(1)
C(28)	784(3)	-1327(2)	9651(1)	48(1)
C(29)	6102(2)	8977(2)	6042(1)	21(1)
C(30)	5571(2)	8180(2)	6532(1)	19(1)
C(31)	6339(2)	7336(2)	6503(1)	18(1)
C(32)	7313(2)	7640(2)	5987(1)	18(1)
C(33)	5724(2)	9999(2)	5857(1)	26(1)
C(34)	4412(2)	8216(2)	7000(1)	22(1)
C(35)	8343(2)	7074(2)	5714(1)	21(1)
C(36)	6105(2)	6319(2)	6931(1)	19(1)
C(37)	7063(2)	4710(2)	7514(1)	24(1)
C(38)	7233(3)	4617(2)	8220(1)	31(1)
C(39)	6913(3)	3566(3)	9336(2)	38(1)
C(40)	5533(4)	3932(3)	9495(2)	45(1)
C(39A)	5970(14)	3344(11)	9133(6)	40(1)
C(40A)	6473(16)	3802(11)	9614(7)	41(1)
C(41)	8257(3)	2979(2)	8582(1)	42(1)
C(42)	7933(4)	1940(2)	8625(2)	50(1)
C(43)	1126(2)	8882(2)	6086(1)	21(1)
C(44)	606(2)	8085(2)	6582(1)	19(1)
C(45)	1338(2)	7233(2)	6530(1)	19(1)
C(46)	2277(2)	7540(2)	6001(1)	18(1)
C(47)	760(2)	9907(2)	5914(1)	26(1)
C(48)	-488(2)	8116(2)	7080(1)	24(1)

C(49)	3293(2)	6976(2)	5719(1)	22(1)
C(50)	1103(2)	6216(2)	6959(1)	21(1)
C(51)	2108(2)	4668(2)	7620(1)	30(1)
C(52)	2814(6)	4639(6)	8213(3)	37(1)
N(12)	2218(4)	5386(3)	8471(2)	44(1)
C(53)	1041(6)	5082(4)	8896(3)	49(1)
C(54)	1030(7)	4153(5)	9558(3)	66(1)
C(55)	3157(5)	5965(3)	8658(2)	48(1)
C(56)	2560(7)	6935(4)	8724(3)	63(1)
C(52A)	2621(15)	4764(14)	8255(6)	40(1)
N(12A)	1781(11)	5392(8)	8591(5)	45(1)
C(53A)	623(11)	4805(9)	8976(5)	48(1)
C(54A)	1450(12)	4420(9)	9581(5)	49(2)
C(55A)	2408(10)	5865(7)	9014(5)	49(1)
C(56A)	3185(12)	6758(8)	8623(6)	56(2)

Table S3. Bond lengths [Å] and angles [°] for M257.

O(1)-C(5)	1.230(3)
O(2)-C(8)	1.239(3)
O(3)-C(19)	1.230(3)
O(4)-C(22)	1.248(3)
O(5)-C(33)	1.228(3)
O(6)-C(36)	1.237(3)
O(7)-C(47)	1.231(3)
O(8)-C(50)	1.243(3)
N(1)-C(4)	1.344(3)
N(1)-C(1)	1.392(3)
N(1)-H(1N)	0.85(3)
N(2)-C(8)	1.344(3)
N(2)-C(9)	1.455(3)
N(2)-H(2N)	0.93(3)
N(3)-C(10)	1.463(3)
N(3)-C(13)	1.471(3)
N(3)-C(11)	1.474(3)
N(4)-C(18)	1.343(3)
N(4)-C(15)	1.387(3)
N(4)-H(4N)	0.83(3)
N(5)-C(22)	1.339(3)
N(5)-C(23)	1.456(3)
N(5)-H(5N)	0.90(3)
N(7)-C(32)	1.344(3)
N(7)-C(29)	1.388(3)
N(7)-H(7N)	0.78(3)
N(8)-C(36)	1.343(3)
N(8)-C(37)	1.456(3)
N(8)-H(8N)	0.94(3)
N(9)-C(41)	1.458(3)
N(9)-C(38)	1.459(3)
N(9)-C(39A)	1.475(13)
N(9)-C(39)	1.504(4)
N(10)-C(46)	1.345(3)
N(10)-C(43)	1.384(3)

N(10)-H(10N)	0.83(3)
N(11)-C(50)	1.340(3)
N(11)-C(51)	1.456(3)
N(11)-H(11N)	0.94(4)
C(1)-C(2)	1.393(3)
C(1)-C(5)	1.424(3)
C(2)-C(3)	1.419(3)
C(2)-C(6)	1.501(3)
C(3)-C(4)	1.401(3)
C(3)-C(8)	1.488(3)
C(4)-C(7)	1.492(3)
C(5)-H(5)	0.9500
C(6)-H(6A)	0.9800
C(6)-H(6B)	0.9800
C(6)-H(6C)	0.9800
C(7)-H(7A)	0.9800
C(7)-H(7B)	0.9800
C(7)-H(7C)	0.9800
C(9)-C(10)	1.521(3)
C(9)-H(9A)	0.9900
C(9)-H(9B)	0.9900
C(10)-H(10A)	0.9900
C(10)-H(10B)	0.9900
C(11)-C(12)	1.502(4)
C(11)-H(11A)	0.9900
C(11)-H(11B)	0.9900
C(12)-H(12A)	0.9800
C(12)-H(12B)	0.9800
C(12)-H(12C)	0.9800
C(13)-C(14)	1.493(4)
C(13)-H(13A)	0.9900
C(13)-H(13B)	0.9900
C(14)-H(14A)	0.9800
C(14)-H(14B)	0.9800
C(14)-H(14C)	0.9800
C(15)-C(16)	1.390(3)
C(15)-C(19)	1.423(3)

C(16)-C(17)	1.421(3)
C(16)-C(20)	1.499(3)
C(17)-C(18)	1.400(3)
C(17)-C(22)	1.479(3)
C(18)-C(21)	1.491(3)
C(19)-H(19)	0.9500
C(20)-H(20A)	0.9800
C(20)-H(20B)	0.9800
C(20)-H(20C)	0.9800
C(21)-H(21A)	0.9800
C(21)-H(21B)	0.9800
C(21)-H(21C)	0.9800
C(23)-C(24)	1.527(3)
C(23)-H(23A)	0.9900
C(23)-H(23B)	0.9900
C(24)-N(6)	1.467(3)
C(24)-H(24A)	0.9900
C(24)-H(24B)	0.9900
N(6)-C(25)	1.459(3)
N(6)-C(27)	1.469(3)
C(25)-C(26)	1.516(4)
C(25)-H(25A)	0.9900
C(25)-H(25B)	0.9900
C(26)-H(26A)	0.9800
C(26)-H(26B)	0.9800
C(26)-H(26C)	0.9800
C(27)-C(28)	1.532(4)
C(27)-H(27A)	0.9900
C(27)-H(27B)	0.9900
C(28)-H(28A)	0.9800
C(28)-H(28B)	0.9800
C(28)-H(28C)	0.9800
C(29)-C(30)	1.390(3)
C(29)-C(33)	1.425(3)
C(30)-C(31)	1.414(3)
C(30)-C(34)	1.501(3)
C(31)-C(32)	1.409(3)

C(31)-C(36)	1.478(3)
C(32)-C(35)	1.486(3)
C(33)-H(33)	0.9500
C(34)-H(34A)	0.9800
C(34)-H(34B)	0.9800
C(34)-H(34C)	0.9800
C(35)-H(35A)	0.9800
C(35)-H(35B)	0.9800
C(35)-H(35C)	0.9800
C(37)-C(38)	1.519(3)
C(37)-H(37A)	0.9900
C(37)-H(37B)	0.9900
C(38)-H(38A)	0.9900
C(38)-H(38B)	0.9900
C(39)-C(40)	1.513(5)
C(39)-H(39A)	0.9900
C(39)-H(39B)	0.9900
C(40)-H(40A)	0.9800
C(40)-H(40B)	0.9800
C(40)-H(40C)	0.9800
C(39A)-C(40A)	1.514(15)
C(39A)-H(39C)	0.9900
C(39A)-H(39D)	0.9900
C(40A)-H(40D)	0.9800
C(40A)-H(40E)	0.9800
C(40A)-H(40F)	0.9800
C(41)-C(42)	1.500(4)
C(41)-H(41A)	0.9900
C(41)-H(41B)	0.9900
C(42)-H(42A)	0.9800
C(42)-H(42B)	0.9800
C(42)-H(42C)	0.9800
C(43)-C(44)	1.392(3)
C(43)-C(47)	1.427(3)
C(44)-C(45)	1.419(3)
C(44)-C(48)	1.499(3)
C(45)-C(46)	1.406(3)

C(45)-C(50)	1.479(3)
C(46)-C(49)	1.485(3)
C(47)-H(47)	0.9500
C(48)-H(48A)	0.9800
C(48)-H(48B)	0.9800
C(48)-H(48C)	0.9800
C(49)-H(49A)	0.9800
C(49)-H(49B)	0.9800
C(49)-H(49C)	0.9800
C(51)-C(52)	1.500(5)
C(51)-C(52A)	1.551(8)
C(51)-H(51A)	0.9900
C(51)-H(51B)	0.9900
C(51)-H(51C)	0.9900
C(51)-H(51D)	0.9900
C(52)-N(12)	1.440(6)
C(52)-H(52A)	0.9900
C(52)-H(52B)	0.9900
N(12)-C(55)	1.433(6)
N(12)-C(53)	1.443(6)
C(53)-C(54)	1.624(7)
C(53)-H(53A)	0.9900
C(53)-H(53B)	0.9900
C(54)-H(54A)	0.9800
C(54)-H(54B)	0.9800
C(54)-H(54C)	0.9800
C(55)-C(56)	1.525(6)
C(55)-H(55A)	0.9900
C(55)-H(55B)	0.9900
C(56)-H(56A)	0.9800
C(56)-H(56B)	0.9800
C(56)-H(56C)	0.9800
C(52A)-N(12A)	1.515(11)
C(52A)-H(52C)	0.9900
C(52A)-H(52D)	0.9900
N(12A)-C(55A)	1.482(11)
N(12A)-C(53A)	1.505(11)

C(53A)-C(54A)	1.541(12)
C(53A)-H(53C)	0.9900
C(53A)-H(53D)	0.9900
C(54A)-H(54D)	0.9800
C(54A)-H(54E)	0.9800
C(54A)-H(54F)	0.9800
C(55A)-C(56A)	1.507(12)
C(55A)-H(55C)	0.9900
C(55A)-H(55D)	0.9900
C(56A)-H(56D)	0.9800
C(56A)-H(56E)	0.9800
C(56A)-H(56F)	0.9800
C(4)-N(1)-C(1)	110.29(18)
C(4)-N(1)-H(1N)	120.5(18)
C(1)-N(1)-H(1N)	129.1(19)
C(8)-N(2)-C(9)	122.09(18)
C(8)-N(2)-H(2N)	118.3(17)
C(9)-N(2)-H(2N)	117.5(18)
C(10)-N(3)-C(13)	112.35(19)
C(10)-N(3)-C(11)	112.07(19)
C(13)-N(3)-C(11)	110.4(2)
C(18)-N(4)-C(15)	110.65(18)
C(18)-N(4)-H(4N)	126.3(19)
C(15)-N(4)-H(4N)	123.0(19)
C(22)-N(5)-C(23)	120.59(18)
C(22)-N(5)-H(5N)	121.7(18)
C(23)-N(5)-H(5N)	116.8(18)
C(32)-N(7)-C(29)	110.23(18)
C(32)-N(7)-H(7N)	126(2)
C(29)-N(7)-H(7N)	124(2)
C(36)-N(8)-C(37)	121.19(18)
C(36)-N(8)-H(8N)	117.8(17)
C(37)-N(8)-H(8N)	119.6(17)
C(41)-N(9)-C(38)	112.4(2)
C(41)-N(9)-C(39A)	126.6(6)
C(38)-N(9)-C(39A)	120.0(6)

C(41)-N(9)-C(39)	106.8(2)
C(38)-N(9)-C(39)	110.4(2)
C(46)-N(10)-C(43)	110.19(18)
C(46)-N(10)-H(10N)	123.1(19)
C(43)-N(10)-H(10N)	126.7(19)
C(50)-N(11)-C(51)	121.92(19)
C(50)-N(11)-H(11N)	117.0(19)
C(51)-N(11)-H(11N)	117.4(18)
N(1)-C(1)-C(2)	107.34(18)
N(1)-C(1)-C(5)	121.7(2)
C(2)-C(1)-C(5)	130.97(19)
C(1)-C(2)-C(3)	106.90(18)
C(1)-C(2)-C(6)	125.43(19)
C(3)-C(2)-C(6)	127.7(2)
C(4)-C(3)-C(2)	107.49(18)
C(4)-C(3)-C(8)	123.06(18)
C(2)-C(3)-C(8)	129.41(19)
N(1)-C(4)-C(3)	107.97(18)
N(1)-C(4)-C(7)	121.47(19)
C(3)-C(4)-C(7)	130.50(19)
O(1)-C(5)-C(1)	125.0(2)
O(1)-C(5)-H(5)	117.5
C(1)-C(5)-H(5)	117.5
C(2)-C(6)-H(6A)	109.5
C(2)-C(6)-H(6B)	109.5
H(6A)-C(6)-H(6B)	109.5
C(2)-C(6)-H(6C)	109.5
H(6A)-C(6)-H(6C)	109.5
H(6B)-C(6)-H(6C)	109.5
C(4)-C(7)-H(7A)	109.5
C(4)-C(7)-H(7B)	109.5
H(7A)-C(7)-H(7B)	109.5
C(4)-C(7)-H(7C)	109.5
H(7A)-C(7)-H(7C)	109.5
H(7B)-C(7)-H(7C)	109.5
O(2)-C(8)-N(2)	122.6(2)
O(2)-C(8)-C(3)	121.73(19)

N(2)-C(8)-C(3)	115.66(18)
N(2)-C(9)-C(10)	112.78(18)
N(2)-C(9)-H(9A)	109.0
C(10)-C(9)-H(9A)	109.0
N(2)-C(9)-H(9B)	109.0
C(10)-C(9)-H(9B)	109.0
H(9A)-C(9)-H(9B)	107.8
N(3)-C(10)-C(9)	110.48(19)
N(3)-C(10)-H(10A)	109.6
C(9)-C(10)-H(10A)	109.6
N(3)-C(10)-H(10B)	109.6
C(9)-C(10)-H(10B)	109.6
H(10A)-C(10)-H(10B)	108.1
N(3)-C(11)-C(12)	112.4(2)
N(3)-C(11)-H(11A)	109.1
C(12)-C(11)-H(11A)	109.1
N(3)-C(11)-H(11B)	109.1
C(12)-C(11)-H(11B)	109.1
H(11A)-C(11)-H(11B)	107.9
C(11)-C(12)-H(12A)	109.5
C(11)-C(12)-H(12B)	109.5
H(12A)-C(12)-H(12B)	109.5
C(11)-C(12)-H(12C)	109.5
H(12A)-C(12)-H(12C)	109.5
H(12B)-C(12)-H(12C)	109.5
N(3)-C(13)-C(14)	112.5(3)
N(3)-C(13)-H(13A)	109.1
C(14)-C(13)-H(13A)	109.1
N(3)-C(13)-H(13B)	109.1
C(14)-C(13)-H(13B)	109.1
H(13A)-C(13)-H(13B)	107.8
C(13)-C(14)-H(14A)	109.5
C(13)-C(14)-H(14B)	109.5
H(14A)-C(14)-H(14B)	109.5
C(13)-C(14)-H(14C)	109.5
H(14A)-C(14)-H(14C)	109.5
H(14B)-C(14)-H(14C)	109.5

N(4)-C(15)-C(16)	107.31(18)
N(4)-C(15)-C(19)	121.91(19)
C(16)-C(15)-C(19)	130.76(19)
C(15)-C(16)-C(17)	106.81(18)
C(15)-C(16)-C(20)	125.50(19)
C(17)-C(16)-C(20)	127.7(2)
C(18)-C(17)-C(16)	107.57(18)
C(18)-C(17)-C(22)	122.81(18)
C(16)-C(17)-C(22)	129.51(19)
N(4)-C(18)-C(17)	107.65(18)
N(4)-C(18)-C(21)	121.44(19)
C(17)-C(18)-C(21)	130.89(19)
O(3)-C(19)-C(15)	125.2(2)
O(3)-C(19)-H(19)	117.4
C(15)-C(19)-H(19)	117.4
C(16)-C(20)-H(20A)	109.5
C(16)-C(20)-H(20B)	109.5
H(20A)-C(20)-H(20B)	109.5
C(16)-C(20)-H(20C)	109.5
H(20A)-C(20)-H(20C)	109.5
H(20B)-C(20)-H(20C)	109.5
C(18)-C(21)-H(21A)	109.5
C(18)-C(21)-H(21B)	109.5
H(21A)-C(21)-H(21B)	109.5
C(18)-C(21)-H(21C)	109.5
H(21A)-C(21)-H(21C)	109.5
H(21B)-C(21)-H(21C)	109.5
O(4)-C(22)-N(5)	121.2(2)
O(4)-C(22)-C(17)	121.18(19)
N(5)-C(22)-C(17)	117.66(18)
N(5)-C(23)-C(24)	112.09(18)
N(5)-C(23)-H(23A)	109.2
C(24)-C(23)-H(23A)	109.2
N(5)-C(23)-H(23B)	109.2
C(24)-C(23)-H(23B)	109.2
H(23A)-C(23)-H(23B)	107.9
N(6)-C(24)-C(23)	112.59(18)

N(6)-C(24)-H(24A)	109.1
C(23)-C(24)-H(24A)	109.1
N(6)-C(24)-H(24B)	109.1
C(23)-C(24)-H(24B)	109.1
H(24A)-C(24)-H(24B)	107.8
C(25)-N(6)-C(24)	113.08(19)
C(25)-N(6)-C(27)	112.33(19)
C(24)-N(6)-C(27)	111.89(19)
N(6)-C(25)-C(26)	112.4(2)
N(6)-C(25)-H(25A)	109.1
C(26)-C(25)-H(25A)	109.1
N(6)-C(25)-H(25B)	109.1
C(26)-C(25)-H(25B)	109.1
H(25A)-C(25)-H(25B)	107.9
C(25)-C(26)-H(26A)	109.5
C(25)-C(26)-H(26B)	109.5
H(26A)-C(26)-H(26B)	109.5
C(25)-C(26)-H(26C)	109.5
H(26A)-C(26)-H(26C)	109.5
H(26B)-C(26)-H(26C)	109.5
N(6)-C(27)-C(28)	116.8(2)
N(6)-C(27)-H(27A)	108.1
C(28)-C(27)-H(27A)	108.1
N(6)-C(27)-H(27B)	108.1
C(28)-C(27)-H(27B)	108.1
H(27A)-C(27)-H(27B)	107.3
C(27)-C(28)-H(28A)	109.5
C(27)-C(28)-H(28B)	109.5
H(28A)-C(28)-H(28B)	109.5
C(27)-C(28)-H(28C)	109.5
H(28A)-C(28)-H(28C)	109.5
H(28B)-C(28)-H(28C)	109.5
N(7)-C(29)-C(30)	107.91(18)
N(7)-C(29)-C(33)	121.73(19)
C(30)-C(29)-C(33)	130.4(2)
C(29)-C(30)-C(31)	106.55(18)
C(29)-C(30)-C(34)	126.42(19)

C(31)-C(30)-C(34)	127.04(19)
C(32)-C(31)-C(30)	107.86(18)
C(32)-C(31)-C(36)	127.18(18)
C(30)-C(31)-C(36)	124.92(18)
N(7)-C(32)-C(31)	107.44(18)
N(7)-C(32)-C(35)	120.82(19)
C(31)-C(32)-C(35)	131.69(19)
O(5)-C(33)-C(29)	125.2(2)
O(5)-C(33)-H(33)	117.4
C(29)-C(33)-H(33)	117.4
C(30)-C(34)-H(34A)	109.5
C(30)-C(34)-H(34B)	109.5
H(34A)-C(34)-H(34B)	109.5
C(30)-C(34)-H(34C)	109.5
H(34A)-C(34)-H(34C)	109.5
H(34B)-C(34)-H(34C)	109.5
C(32)-C(35)-H(35A)	109.5
C(32)-C(35)-H(35B)	109.5
H(35A)-C(35)-H(35B)	109.5
C(32)-C(35)-H(35C)	109.5
H(35A)-C(35)-H(35C)	109.5
H(35B)-C(35)-H(35C)	109.5
O(6)-C(36)-N(8)	121.7(2)
O(6)-C(36)-C(31)	121.25(18)
N(8)-C(36)-C(31)	117.00(18)
N(8)-C(37)-C(38)	112.28(18)
N(8)-C(37)-H(37A)	109.1
C(38)-C(37)-H(37A)	109.1
N(8)-C(37)-H(37B)	109.1
C(38)-C(37)-H(37B)	109.1
H(37A)-C(37)-H(37B)	107.9
N(9)-C(38)-C(37)	111.8(2)
N(9)-C(38)-H(38A)	109.3
C(37)-C(38)-H(38A)	109.3
N(9)-C(38)-H(38B)	109.3
C(37)-C(38)-H(38B)	109.3
H(38A)-C(38)-H(38B)	107.9

N(9)-C(39)-C(40)	111.5(3)
N(9)-C(39)-H(39A)	109.3
C(40)-C(39)-H(39A)	109.3
N(9)-C(39)-H(39B)	109.3
C(40)-C(39)-H(39B)	109.3
H(39A)-C(39)-H(39B)	108.0
C(39)-C(40)-H(40A)	109.5
C(39)-C(40)-H(40B)	109.5
H(40A)-C(40)-H(40B)	109.5
C(39)-C(40)-H(40C)	109.5
H(40A)-C(40)-H(40C)	109.5
H(40B)-C(40)-H(40C)	109.5
N(9)-C(39A)-C(40A)	98.8(11)
N(9)-C(39A)-H(39C)	112.0
C(40A)-C(39A)-H(39C)	112.0
N(9)-C(39A)-H(39D)	112.0
C(40A)-C(39A)-H(39D)	112.0
H(39C)-C(39A)-H(39D)	109.7
C(39A)-C(40A)-H(40D)	109.5
C(39A)-C(40A)-H(40E)	109.5
H(40D)-C(40A)-H(40E)	109.5
C(39A)-C(40A)-H(40F)	109.5
H(40D)-C(40A)-H(40F)	109.5
H(40E)-C(40A)-H(40F)	109.5
N(9)-C(41)-C(42)	114.1(3)
N(9)-C(41)-H(41A)	108.7
C(42)-C(41)-H(41A)	108.7
N(9)-C(41)-H(41B)	108.7
C(42)-C(41)-H(41B)	108.7
H(41A)-C(41)-H(41B)	107.6
C(41)-C(42)-H(42A)	109.5
C(41)-C(42)-H(42B)	109.5
H(42A)-C(42)-H(42B)	109.5
C(41)-C(42)-H(42C)	109.5
H(42A)-C(42)-H(42C)	109.5
H(42B)-C(42)-H(42C)	109.5
N(10)-C(43)-C(44)	108.08(18)

N(10)-C(43)-C(47)	121.9(2)
C(44)-C(43)-C(47)	130.02(19)
C(43)-C(44)-C(45)	106.33(18)
C(43)-C(44)-C(48)	127.06(19)
C(45)-C(44)-C(48)	126.6(2)
C(46)-C(45)-C(44)	107.70(18)
C(46)-C(45)-C(50)	127.66(19)
C(44)-C(45)-C(50)	124.63(19)
N(10)-C(46)-C(45)	107.70(18)
N(10)-C(46)-C(49)	120.81(19)
C(45)-C(46)-C(49)	131.49(19)
O(7)-C(47)-C(43)	125.3(2)
O(7)-C(47)-H(47)	117.4
C(43)-C(47)-H(47)	117.4
C(44)-C(48)-H(48A)	109.5
C(44)-C(48)-H(48B)	109.5
H(48A)-C(48)-H(48B)	109.5
C(44)-C(48)-H(48C)	109.5
H(48A)-C(48)-H(48C)	109.5
H(48B)-C(48)-H(48C)	109.5
C(46)-C(49)-H(49A)	109.5
C(46)-C(49)-H(49B)	109.5
H(49A)-C(49)-H(49B)	109.5
C(46)-C(49)-H(49C)	109.5
H(49A)-C(49)-H(49C)	109.5
H(49B)-C(49)-H(49C)	109.5
O(8)-C(50)-N(11)	122.0(2)
O(8)-C(50)-C(45)	121.20(18)
N(11)-C(50)-C(45)	116.81(19)
N(11)-C(51)-C(52)	112.6(4)
N(11)-C(51)-C(52A)	108.8(8)
N(11)-C(51)-H(51A)	109.1
C(52)-C(51)-H(51A)	109.1
N(11)-C(51)-H(51B)	109.1
C(52)-C(51)-H(51B)	109.1
H(51A)-C(51)-H(51B)	107.8
N(11)-C(51)-H(51C)	109.9

C(52A)-C(51)-H(51C)	109.9
N(11)-C(51)-H(51D)	109.9
C(52A)-C(51)-H(51D)	109.9
H(51C)-C(51)-H(51D)	108.3
N(12)-C(52)-C(51)	108.4(4)
N(12)-C(52)-H(52A)	110.0
C(51)-C(52)-H(52A)	110.0
N(12)-C(52)-H(52B)	110.0
C(51)-C(52)-H(52B)	110.0
H(52A)-C(52)-H(52B)	108.4
C(55)-N(12)-C(52)	114.6(4)
C(55)-N(12)-C(53)	117.1(4)
C(52)-N(12)-C(53)	114.7(5)
N(12)-C(53)-C(54)	123.5(5)
N(12)-C(53)-H(53A)	106.4
C(54)-C(53)-H(53A)	106.4
N(12)-C(53)-H(53B)	106.4
C(54)-C(53)-H(53B)	106.4
H(53A)-C(53)-H(53B)	106.5
C(53)-C(54)-H(54A)	109.5
C(53)-C(54)-H(54B)	109.5
H(54A)-C(54)-H(54B)	109.5
C(53)-C(54)-H(54C)	109.5
H(54A)-C(54)-H(54C)	109.5
H(54B)-C(54)-H(54C)	109.5
N(12)-C(55)-C(56)	113.5(4)
N(12)-C(55)-H(55A)	108.9
C(56)-C(55)-H(55A)	108.9
N(12)-C(55)-H(55B)	108.9
C(56)-C(55)-H(55B)	108.9
H(55A)-C(55)-H(55B)	107.7
C(55)-C(56)-H(56A)	109.5
C(55)-C(56)-H(56B)	109.5
H(56A)-C(56)-H(56B)	109.5
C(55)-C(56)-H(56C)	109.5
H(56A)-C(56)-H(56C)	109.5
H(56B)-C(56)-H(56C)	109.5

N(12A)-C(52A)-C(51)	117.1(8)
N(12A)-C(52A)-H(52C)	108.0
C(51)-C(52A)-H(52C)	108.0
N(12A)-C(52A)-H(52D)	108.0
C(51)-C(52A)-H(52D)	108.0
H(52C)-C(52A)-H(52D)	107.3
C(55A)-N(12A)-C(53A)	107.6(8)
C(55A)-N(12A)-C(52A)	120.2(8)
C(53A)-N(12A)-C(52A)	109.7(10)
N(12A)-C(53A)-C(54A)	91.4(8)
N(12A)-C(53A)-H(53C)	113.4
C(54A)-C(53A)-H(53C)	113.4
N(12A)-C(53A)-H(53D)	113.4
C(54A)-C(53A)-H(53D)	113.4
H(53C)-C(53A)-H(53D)	110.7
C(53A)-C(54A)-H(54D)	109.5
C(53A)-C(54A)-H(54E)	109.5
H(54D)-C(54A)-H(54E)	109.5
C(53A)-C(54A)-H(54F)	109.5
H(54D)-C(54A)-H(54F)	109.5
H(54E)-C(54A)-H(54F)	109.5
N(12A)-C(55A)-C(56A)	111.3(9)
N(12A)-C(55A)-H(55C)	109.4
C(56A)-C(55A)-H(55C)	109.4
N(12A)-C(55A)-H(55D)	109.4
C(56A)-C(55A)-H(55D)	109.4
H(55C)-C(55A)-H(55D)	108.0
C(55A)-C(56A)-H(56D)	109.5
C(55A)-C(56A)-H(56E)	109.5
H(56D)-C(56A)-H(56E)	109.5
C(55A)-C(56A)-H(56F)	109.5
H(56D)-C(56A)-H(56F)	109.5
H(56E)-C(56A)-H(56F)	109.5

Symmetry transformations used to generate equivalent atoms:

Table S4. Anisotropic displacement parameters ($Å^2 x 10^3$) for M257. The anisotropic displacement factor exponent takes the form: $-2\pi^2[h^2 a^{*2}U^{11} + ... + 2 h k a^* b^* U^{12}]$

	U^{11}	U^{22}	U^{33}	U^{23}	U^{13}	U^{12}
O(1)	27(1)	20(1)	35(1)	-7(1)	7(1)	4(1)
O(2)	14(1)	22(1)	37(1)	-7(1)	-2(1)	3(1)
O(3)	24(1)	19(1)	34(1)	-8(1)	4(1)	3(1)
O(4)	12(1)	22(1)	34(1)	-10(1)	-1(1)	3(1)
O(5)	34(1)	20(1)	37(1)	-7(1)	14(1)	4(1)
O(6)	11(1)	26(1)	42(1)	-3(1)	2(1)	-2(1)
O(7)	36(1)	22(1)	36(1)	-7(1)	14(1)	4(1)
O(8)	10(1)	28(1)	38(1)	-6(1)	2(1)	-1(1)
N(1)	15(1)	20(1)	23(1)	-8(1)	3(1)	3(1)
N(2)	12(1)	21(1)	32(1)	-6(1)	-2(1)	1(1)
N(3)	36(1)	21(1)	33(1)	-6(1)	2(1)	4(1)
N(4)	16(1)	18(1)	23(1)	-7(1)	2(1)	3(1)
N(5)	13(1)	23(1)	29(1)	-9(1)	-1(1)	2(1)
N(7)	18(1)	17(1)	23(1)	-6(1)	4(1)	1(1)
N(8)	14(1)	19(1)	30(1)	-6(1)	1(1)	1(1)
N(9)	51(1)	26(1)	37(1)	1(1)	15(1)	9(1)
N(10)	17(1)	19(1)	23(1)	-8(1)	3(1)	1(1)
N(11)	13(1)	23(1)	44(1)	-1(1)	2(1)	-1(1)
C(1)	14(1)	22(1)	28(1)	-14(1)	2(1)	2(1)
C(2)	12(1)	22(1)	27(1)	-13(1)	0(1)	-1(1)
C(3)	13(1)	19(1)	26(1)	-9(1)	-2(1)	1(1)
C(4)	14(1)	20(1)	26(1)	-11(1)	-2(1)	1(1)
C(5)	16(1)	23(1)	31(1)	-12(1)	4(1)	1(1)
C(6)	17(1)	23(1)	33(1)	-13(1)	4(1)	-1(1)
C(7)	16(1)	24(1)	28(1)	-10(1)	2(1)	5(1)
C(8)	15(1)	21(1)	27(1)	-12(1)	1(1)	0(1)
C(9)	19(1)	20(1)	35(1)	-9(1)	0(1)	-1(1)
C(10)	30(1)	21(1)	35(1)	-9(1)	1(1)	2(1)
C(11)	39(2)	38(2)	34(1)	-7(1)	0(1)	1(1)
C(12)	48(2)	71(2)	42(2)	-16(2)	6(1)	9(2)
C(13)	43(2)	28(1)	40(1)	-8(1)	1(1)	9(1)
C(14)	65(2)	38(2)	92(3)	-34(2)	-22(2)	14(2)

C(15)	14(1)	21(1)	27(1)	-12(1)	1(1)	3(1)
C(16)	12(1)	22(1)	27(1)	-13(1)	0(1)	0(1)
C(17)	14(1)	21(1)	24(1)	-10(1)	-2(1)	3(1)
C(18)	13(1)	19(1)	26(1)	-11(1)	-2(1)	3(1)
C(19)	16(1)	23(1)	31(1)	-12(1)	2(1)	1(1)
C(20)	16(1)	26(1)	30(1)	-13(1)	4(1)	-1(1)
C(21)	16(1)	24(1)	27(1)	-10(1)	2(1)	6(1)
C(22)	14(1)	23(1)	25(1)	-12(1)	-1(1)	1(1)
C(23)	22(1)	21(1)	32(1)	-9(1)	3(1)	-2(1)
C(24)	19(1)	26(1)	33(1)	-6(1)	1(1)	4(1)
N(6)	22(1)	32(1)	29(1)	-10(1)	2(1)	2(1)
C(25)	35(1)	38(1)	37(1)	-15(1)	-5(1)	3(1)
C(26)	78(2)	51(2)	50(2)	-14(2)	-11(2)	-18(2)
C(27)	29(1)	38(1)	34(1)	-6(1)	5(1)	-2(1)
C(28)	47(2)	43(2)	43(2)	0(1)	7(1)	2(1)
C(29)	17(1)	22(1)	25(1)	-11(1)	2(1)	1(1)
C(30)	12(1)	24(1)	24(1)	-11(1)	-1(1)	1(1)
C(31)	11(1)	18(1)	26(1)	-8(1)	-1(1)	-1(1)
C(32)	15(1)	17(1)	23(1)	-7(1)	-3(1)	1(1)
C(33)	24(1)	23(1)	30(1)	-11(1)	7(1)	1(1)
C(34)	15(1)	24(1)	30(1)	-12(1)	3(1)	2(1)
C(35)	15(1)	21(1)	27(1)	-9(1)	1(1)	2(1)
C(36)	12(1)	20(1)	26(1)	-9(1)	-1(1)	0(1)
C(37)	18(1)	17(1)	34(1)	-7(1)	-1(1)	1(1)
C(38)	38(1)	22(1)	32(1)	-6(1)	4(1)	6(1)
C(39)	35(2)	42(2)	31(2)	-6(1)	4(1)	8(1)
C(40)	41(2)	49(2)	37(2)	-4(1)	8(1)	11(1)
C(39A)	36(2)	44(2)	33(2)	-5(2)	5(2)	8(2)
C(40A)	37(2)	45(2)	34(2)	-6(2)	5(2)	8(2)
C(41)	58(2)	33(1)	31(1)	-4(1)	1(1)	16(1)
C(42)	64(2)	37(2)	49(2)	-16(1)	-13(2)	13(1)
C(43)	18(1)	24(1)	23(1)	-12(1)	3(1)	2(1)
C(44)	12(1)	22(1)	25(1)	-12(1)	0(1)	0(1)
C(45)	11(1)	20(1)	26(1)	-9(1)	-1(1)	-1(1)
C(46)	13(1)	20(1)	24(1)	-11(1)	-2(1)	0(1)
C(47)	24(1)	24(1)	29(1)	-10(1)	6(1)	1(1)
C(48)	17(1)	28(1)	29(1)	-13(1)	5(1)	-1(1)

C(49)	14(1)	22(1)	29(1)	-10(1)	1(1)	2(1)
C(50)	13(1)	22(1)	30(1)	-11(1)	1(1)	1(1)
C(51)	19(1)	20(1)	43(1)	-3(1)	3(1)	1(1)
C(52)	21(2)	29(2)	57(2)	-7(1)	-6(2)	2(2)
N(12)	35(2)	42(1)	62(2)	-25(1)	-3(1)	-2(1)
C(53)	43(2)	51(2)	63(2)	-31(2)	-5(2)	-2(2)
C(54)	61(3)	75(3)	67(2)	-30(2)	-6(2)	1(2)
C(55)	44(2)	40(2)	66(2)	-24(2)	-2(2)	-5(2)
C(56)	64(3)	48(2)	83(3)	-32(2)	8(2)	-3(2)
C(52A)	26(3)	32(2)	61(2)	-9(2)	-9(2)	-1(2)
N(12A)	36(2)	39(2)	63(2)	-21(2)	-8(2)	-3(2)
C(53A)	40(3)	49(2)	63(2)	-26(2)	-6(2)	-4(2)
C(54A)	44(4)	58(4)	58(3)	-35(3)	-8(3)	-14(3)
C(55A)	42(2)	43(2)	67(2)	-24(2)	-4(2)	-4(2)
C(56A)	48(4)	43(3)	79(3)	-22(3)	1(3)	-7(3)

Table S5. Hydrogen coordinates (x 10^4) and isotropic displacement parameters (Å^{2}x 10^3) for M257.

	x	y	z	U(eq)
H(1N)	2200(30)	6070(20)	4596(14)	29
H(2N)	4690(30)	9050(20)	2861(13)	34
H(4N)	2710(30)	4040(20)	5316(14)	29
H(5N)	400(30)	980(20)	7158(13)	33
H(7N)	7590(30)	8960(20)	5435(14)	29
H(8N)	8030(30)	6020(20)	7010(13)	33
H(10N)	2590(30)	8850(20)	5423(14)	30
H(11N)	3020(30)	5930(20)	7029(14)	44
H(5)	4997	5171	3745	27
H(6A)	4531	7438	2392	35
H(6B)	5342	6516	2842	35
H(6C)	5636	7613	2841	35
H(7A)	1405	8454	4386	34
H(7B)	870	7375	4769	34
H(7C)	302	7975	4083	34
H(9A)	4723	10618	2257	30
H(9B)	3197	10727	2498	30
H(10A)	3959	10004	1429	34
H(10B)	2450	10221	1652	34
H(11A)	3404	12235	253	46
H(11B)	2850	11140	429	46
H(12A)	4947	11333	-176	82
H(12B)	4998	10472	502	82
H(12C)	5612	11526	422	82
H(13A)	2021	11794	1799	46
H(13B)	1656	12128	1056	46
H(14A)	2051	13513	1363	92
H(14B)	3175	13421	824	92
H(14C)	3514	13092	1571	92
H(19)	30	4899	6176	27
H(20A)	514	2539	7524	34

H(20B)	-651	2568	7050	34
H(20C)	-153	3574	7132	34
H(21A)	3661	1634	5528	34
H(21B)	4749	2141	5821	34
H(21C)	4159	2721	5137	34
H(23A)	1959	-638	7497	30
H(23B)	525	-519	7840	30
H(24A)	2345	-865	8566	32
H(24B)	2915	201	8174	32
H(25A)	2717	276	9292	43
H(25B)	1372	817	9461	43
H(26A)	2981	2006	8975	89
H(26B)	1782	2157	8501	89
H(26C)	3149	1621	8355	89
H(27A)	-271	-688	8835	42
H(27B)	-406	-68	9335	42
H(28A)	44	-1751	9877	72
H(28B)	1236	-1095	9962	72
H(28C)	1422	-1704	9452	72
H(33)	4997	10192	6099	31
H(34A)	4680	7908	7444	33
H(34B)	3662	7859	6919	33
H(34C)	4137	8906	6942	33
H(35A)	7992	6430	5739	31
H(35B)	9146	6977	5962	31
H(35C)	8572	7443	5260	31
H(37A)	7748	4288	7371	28
H(37B)	6172	4468	7473	28
H(38A)	8129	4849	8263	38
H(38B)	6556	5045	8362	38
H(39A)	7595	3980	9429	45
H(39B)	7055	2878	9616	45
H(40A)	5408	3818	9963	68
H(40B)	4856	3576	9356	68
H(40C)	5440	4642	9268	68
H(39C)	5873	2620	9320	48
H(39D)	5112	3651	8961	48

H(40D)	6070	3472	10047	62
H(40E)	6224	4507	9486	62
H(40F)	7449	3724	9619	62
H(41A)	8856	2958	8930	51
H(41B)	8751	3286	8162	51
H(42A)	8765	1553	8621	74
H(42B)	7423	1947	8255	74
H(42C)	7399	1640	9028	74
H(47)	63	10099	6171	31
H(48A)	-209	7731	7511	36
H(48B)	-1299	7836	6987	36
H(48C)	-673	8803	7069	36
H(49A)	2964	6314	5776	32
H(49B)	4128	6919	5940	32
H(49C)	3458	7322	5256	32
H(51A)	2510	4156	7439	35
H(51B)	1156	4509	7745	35
H(51C)	2663	4173	7477	35
H(51D)	1171	4446	7695	35
H(52A)	2727	3979	8540	44
H(52B)	3777	4766	8099	44
H(53A)	354	4951	8628	58
H(53B)	714	5666	9023	58
H(54A)	103	3979	9710	99
H(54B)	1478	4335	9890	99
H(54C)	1502	3586	9471	99
H(55A)	3920	6111	8332	57
H(55B)	3507	5570	9078	57
H(56A)	1890	6797	9089	94
H(56B)	2137	7304	8325	94
H(56C)	3271	7330	8802	94
H(52C)	2709	4091	8565	48
H(52D)	3529	5039	8156	48
H(53C)	354	4276	8806	58
H(53D)	-161	5221	9038	58
H(54D)	847	4174	9967	73
H(54E)	1975	4959	9624	73

H(54F)	2055	3883	9535	73
H(55C)	3016	5381	9299	59
H(55D)	1704	6066	9293	59
H(56D)	2585	7236	8339	67
H(56E)	3905	6555	8359	67
H(56F)	3570	7063	8915	67

Table S6. Torsion angles [°] for M257.

C(4)-N(1)-C(1)-C(2)	0.0(2)
C(4)-N(1)-C(1)-C(5)	179.61(19)
N(1)-C(1)-C(2)-C(3)	-0.1(2)
C(5)-C(1)-C(2)-C(3)	-179.7(2)
N(1)-C(1)-C(2)-C(6)	-179.65(19)
C(5)-C(1)-C(2)-C(6)	0.7(4)
C(1)-C(2)-C(3)-C(4)	0.2(2)
C(6)-C(2)-C(3)-C(4)	179.8(2)
C(1)-C(2)-C(3)-C(8)	-177.4(2)
C(6)-C(2)-C(3)-C(8)	2.1(3)
C(1)-N(1)-C(4)-C(3)	0.2(2)
C(1)-N(1)-C(4)-C(7)	-177.36(18)
C(2)-C(3)-C(4)-N(1)	-0.3(2)
C(8)-C(3)-C(4)-N(1)	177.56(18)
C(2)-C(3)-C(4)-C(7)	177.0(2)
C(8)-C(3)-C(4)-C(7)	-5.2(3)
N(1)-C(1)-C(5)-O(1)	1.1(3)
C(2)-C(1)-C(5)-O(1)	-179.3(2)
C(9)-N(2)-C(8)-O(2)	2.2(3)
C(9)-N(2)-C(8)-C(3)	-178.45(18)
C(4)-C(3)-C(8)-O(2)	-34.3(3)
C(2)-C(3)-C(8)-O(2)	143.0(2)
C(4)-C(3)-C(8)-N(2)	146.4(2)
C(2)-C(3)-C(8)-N(2)	-36.3(3)
C(8)-N(2)-C(9)-C(10)	-79.5(3)
C(13)-N(3)-C(10)-C(9)	-76.3(3)
C(11)-N(3)-C(10)-C(9)	158.7(2)
N(2)-C(9)-C(10)-N(3)	-174.70(18)
C(10)-N(3)-C(11)-C(12)	-72.6(3)
C(13)-N(3)-C(11)-C(12)	161.3(3)
C(10)-N(3)-C(13)-C(14)	144.8(3)
C(11)-N(3)-C(13)-C(14)	-89.3(3)
C(18)-N(4)-C(15)-C(16)	-0.1(2)
C(18)-N(4)-C(15)-C(19)	-178.70(19)
N(4)-C(15)-C(16)-C(17)	-0.2(2)

C(19)-C(15)-C(16)-C(17)	178.2(2)
N(4)-C(15)-C(16)-C(20)	178.58(19)
C(19)-C(15)-C(16)-C(20)	-2.9(4)
C(15)-C(16)-C(17)-C(18)	0.4(2)
C(20)-C(16)-C(17)-C(18)	-178.3(2)
C(15)-C(16)-C(17)-C(22)	176.7(2)
C(20)-C(16)-C(17)-C(22)	-2.1(4)
C(15)-N(4)-C(18)-C(17)	0.3(2)
C(15)-N(4)-C(18)-C(21)	178.57(18)
C(16)-C(17)-C(18)-N(4)	-0.5(2)
C(22)-C(17)-C(18)-N(4)	-177.01(18)
C(16)-C(17)-C(18)-C(21)	-178.5(2)
C(22)-C(17)-C(18)-C(21)	5.0(3)
N(4)-C(15)-C(19)-O(3)	-0.6(3)
C(16)-C(15)-C(19)-O(3)	-178.9(2)
C(23)-N(5)-C(22)-O(4)	3.9(3)
C(23)-N(5)-C(22)-C(17)	-175.12(18)
C(18)-C(17)-C(22)-O(4)	28.9(3)
C(16)-C(17)-C(22)-O(4)	-146.8(2)
C(18)-C(17)-C(22)-N(5)	-152.1(2)
C(16)-C(17)-C(22)-N(5)	32.2(3)
C(22)-N(5)-C(23)-C(24)	72.8(2)
N(5)-C(23)-C(24)-N(6)	70.1(2)
C(23)-C(24)-N(6)-C(25)	-152.7(2)
C(23)-C(24)-N(6)-C(27)	79.3(2)
C(24)-N(6)-C(25)-C(26)	76.7(3)
C(27)-N(6)-C(25)-C(26)	-155.5(2)
C(25)-N(6)-C(27)-C(28)	-61.5(3)
C(24)-N(6)-C(27)-C(28)	66.9(3)
C(32)-N(7)-C(29)-C(30)	-0.4(2)
C(32)-N(7)-C(29)-C(33)	179.7(2)
N(7)-C(29)-C(30)-C(31)	0.8(2)
C(33)-C(29)-C(30)-C(31)	-179.3(2)
N(7)-C(29)-C(30)-C(34)	-179.51(19)
C(33)-C(29)-C(30)-C(34)	0.4(4)
C(29)-C(30)-C(31)-C(32)	-0.9(2)
C(34)-C(30)-C(31)-C(32)	179.4(2)

C(29)-C(30)-C(31)-C(36)	-178.86(19)
C(34)-C(30)-C(31)-C(36)	1.4(3)
C(29)-N(7)-C(32)-C(31)	-0.1(2)
C(29)-N(7)-C(32)-C(35)	177.72(18)
C(30)-C(31)-C(32)-N(7)	0.6(2)
C(36)-C(31)-C(32)-N(7)	178.54(19)
C(30)-C(31)-C(32)-C(35)	-176.9(2)
C(36)-C(31)-C(32)-C(35)	1.0(4)
N(7)-C(29)-C(33)-O(5)	0.1(4)
C(30)-C(29)-C(33)-O(5)	-179.7(2)
C(37)-N(8)-C(36)-O(6)	-0.3(3)
C(37)-N(8)-C(36)-C(31)	179.88(18)
C(32)-C(31)-C(36)-O(6)	-143.3(2)
C(30)-C(31)-C(36)-O(6)	34.3(3)
C(32)-C(31)-C(36)-N(8)	36.5(3)
C(30)-C(31)-C(36)-N(8)	-145.9(2)
C(36)-N(8)-C(37)-C(38)	-94.6(2)
C(41)-N(9)-C(38)-C(37)	75.1(3)
C(39A)-N(9)-C(38)-C(37)	-115.5(6)
C(39)-N(9)-C(38)-C(37)	-165.7(2)
N(8)-C(37)-C(38)-N(9)	179.24(19)
C(41)-N(9)-C(39)-C(40)	-163.7(3)
C(38)-N(9)-C(39)-C(40)	73.8(3)
C(41)-N(9)-C(39A)-C(40A)	94.2(10)
C(38)-N(9)-C(39A)-C(40A)	-73.5(10)
C(38)-N(9)-C(41)-C(42)	-143.4(2)
C(39A)-N(9)-C(41)-C(42)	48.1(8)
C(39)-N(9)-C(41)-C(42)	95.3(3)
C(46)-N(10)-C(43)-C(44)	-0.8(2)
C(46)-N(10)-C(43)-C(47)	179.9(2)
N(10)-C(43)-C(44)-C(45)	0.7(2)
C(47)-C(43)-C(44)-C(45)	179.9(2)
N(10)-C(43)-C(44)-C(48)	179.6(2)
C(47)-C(43)-C(44)-C(48)	-1.2(4)
C(43)-C(44)-C(45)-C(46)	-0.3(2)
C(48)-C(44)-C(45)-C(46)	-179.2(2)
C(43)-C(44)-C(45)-C(50)	-179.18(19)

C(48)-C(44)-C(45)-C(50)	1.9(3)
C(43)-N(10)-C(46)-C(45)	0.6(2)
C(43)-N(10)-C(46)-C(49)	-179.93(18)
C(44)-C(45)-C(46)-N(10)	-0.2(2)
C(50)-C(45)-C(46)-N(10)	178.65(19)
C(44)-C(45)-C(46)-C(49)	-179.6(2)
C(50)-C(45)-C(46)-C(49)	-0.8(4)
N(10)-C(43)-C(47)-O(7)	-0.2(4)
C(44)-C(43)-C(47)-O(7)	-179.3(2)
C(51)-N(11)-C(50)-O(8)	-10.4(4)
C(51)-N(11)-C(50)-C(45)	170.1(2)
C(46)-C(45)-C(50)-O(8)	-140.4(2)
C(44)-C(45)-C(50)-O(8)	38.2(3)
C(46)-C(45)-C(50)-N(11)	39.1(3)
C(44)-C(45)-C(50)-N(11)	-142.3(2)
C(50)-N(11)-C(51)-C(52)	-112.4(3)
C(50)-N(11)-C(51)-C(52A)	-101.8(6)
N(11)-C(51)-C(52)-N(12)	58.9(6)
C(51)-C(52)-N(12)-C(55)	-135.4(5)
C(51)-C(52)-N(12)-C(53)	84.9(6)
C(55)-N(12)-C(53)-C(54)	-79.6(7)
C(52)-N(12)-C(53)-C(54)	59.0(7)
C(52)-N(12)-C(55)-C(56)	161.7(5)
C(53)-N(12)-C(55)-C(56)	-59.7(6)
N(11)-C(51)-C(52A)-N(12A)	67.3(14)
C(51)-C(52A)-N(12A)-C(55A)	-154.4(12)
C(51)-C(52A)-N(12A)-C(53A)	80.2(15)
C(55A)-N(12A)-C(53A)-C(54A)	-42.1(11)
C(52A)-N(12A)-C(53A)-C(54A)	90.2(10)
C(53A)-N(12A)-C(55A)-C(56A)	-157.4(10)
C(52A)-N(12A)-C(55A)-C(56A)	76.2(14)

Symmetry transformations used to generate equivalent atoms:

Table S7. Hydrogen bonds for M257 [Å and °].

D-H...A	d(D-H)	d(H...A)	d(D...A)	<(DHA)
N(1)-H(1N)...O(3)	0.85(3)	2.02(3)	2.847(2)	165(3)
N(2)-H(2N)...O(4)#1	0.93(3)	1.99(3)	2.850(2)	153(3)
N(4)-H(4N)...O(1)	0.83(3)	2.05(3)	2.832(2)	157(3)
N(5)-H(5N)...O(2)#2	0.90(3)	2.09(3)	2.925(2)	153(3)
N(7)-H(7N)...O(7)#3	0.78(3)	2.12(3)	2.857(2)	160(3)
N(8)-H(8N)...O(8)#4	0.94(3)	1.96(3)	2.812(2)	149(2)
N(10)-H(10N)...O(5)#3	0.83(3)	2.02(3)	2.836(2)	166(3)
N(11)-H(11N)...O(6)	0.94(4)	1.98(3)	2.866(2)	155(3)

Symmetry transformations used to generate equivalent atoms:

#1 -x+1,-y+1,-z+1   #2 -x,-y+1,-z+1   #3 -x+1,-y+2,-z+1

#4 x+1,y,z

8. NMR Spectra

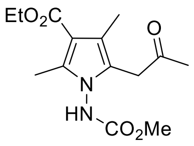

3a

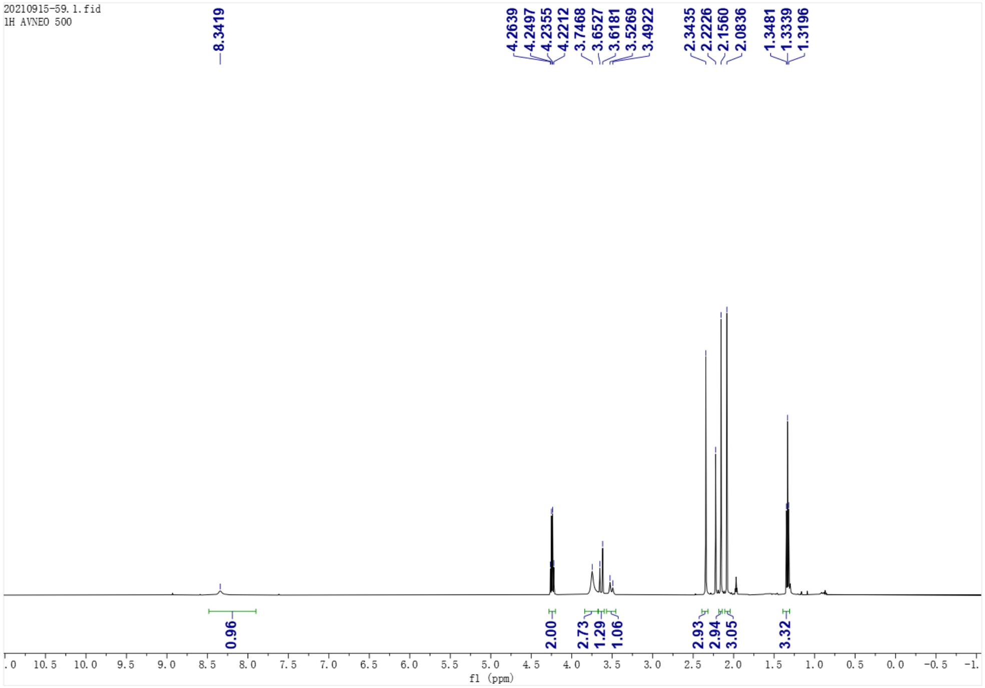

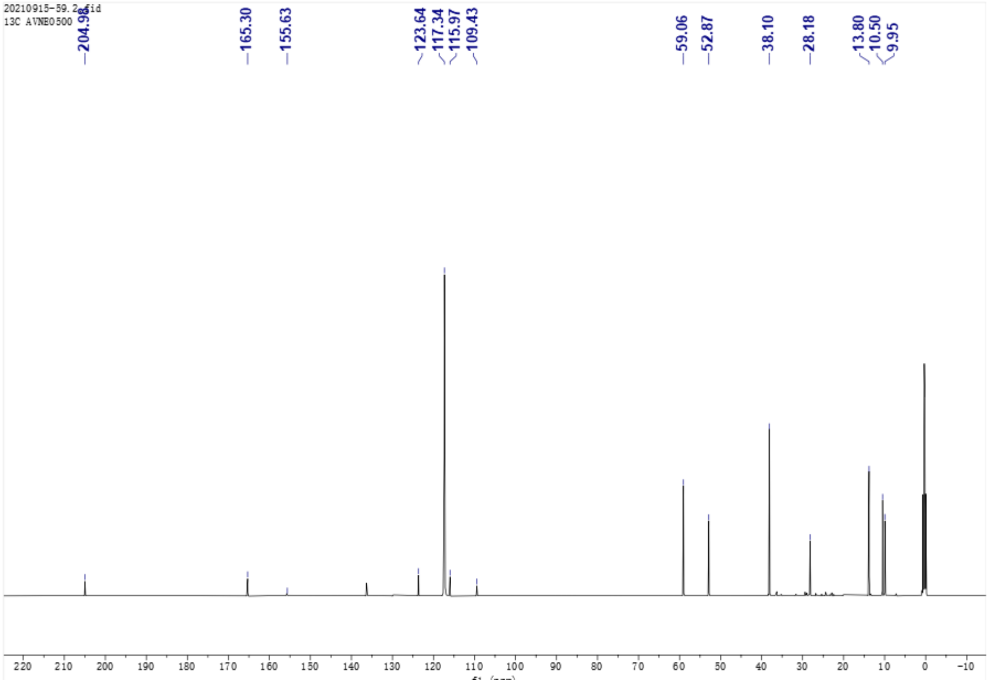

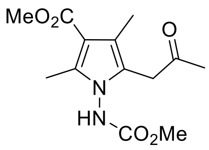
MeO2C
O
N
HN
CO2Me

3b

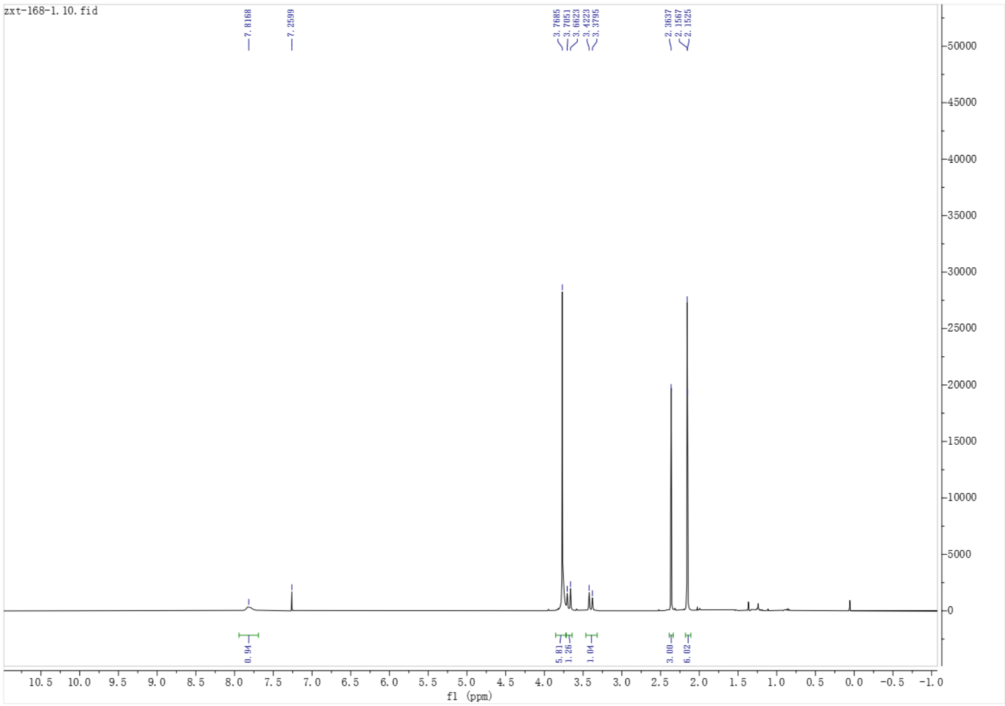
zxt-168-1.10.fid
7.8168
7.2599
3.7685
3.7051
3.6623
3.4223
3.3795
2.3637
2.1567
2.1525
0.94
5.81
1.26
1.04
3.00
6.02
f1 (ppm)

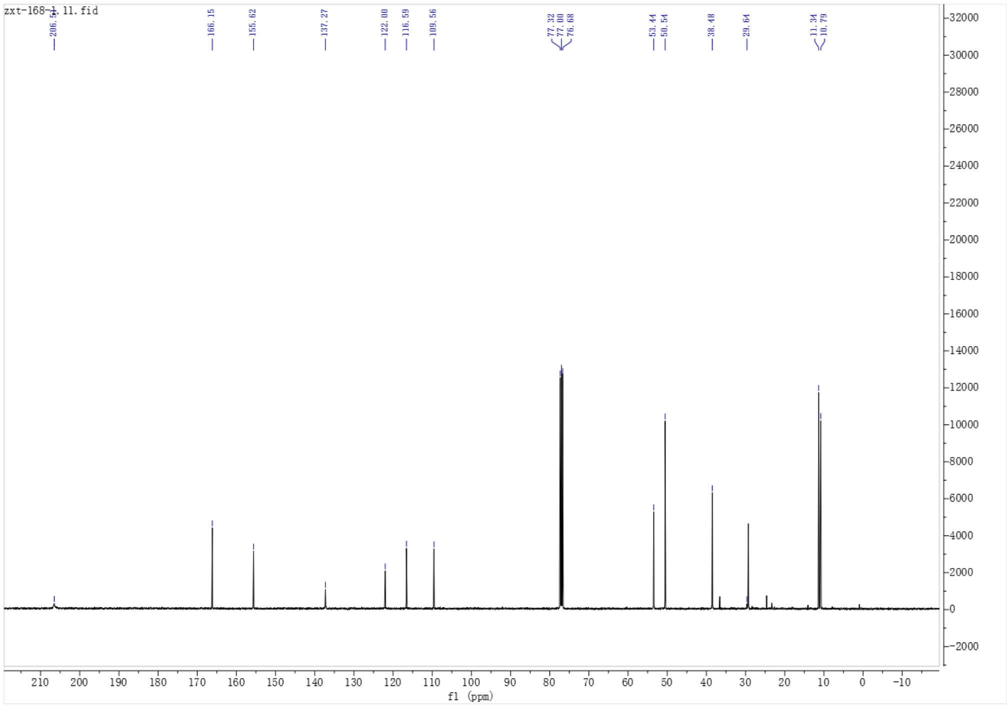
zxt-168-1.11.fid
166.15
155.62
137.27
122.00
116.59
109.56
77.32
77.00
76.68
53.44
50.54
38.48
29.64
11.34
10.79
f1 (ppm)

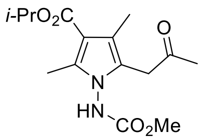

i-PrO2C
O
N
HN
CO2Me

3c

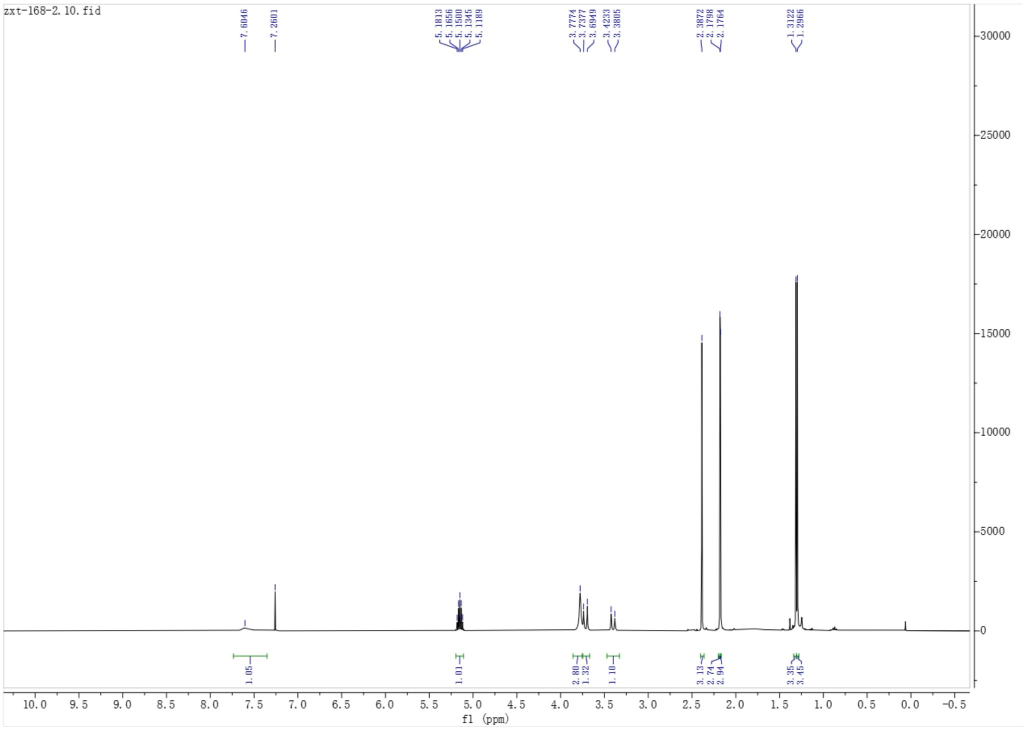

zxt-168-2.10.fid
7.6046
7.2601
5.1813
5.1656
5.1500
5.1345
5.1189
3.7774
3.7377
3.6949
3.4233
3.3805
2.3872
2.1798
2.1764
1.3122
1.2966
1.05
1.01
2.80
1.32
1.10
3.13
2.74
2.94
3.35
3.45
f1 (ppm)

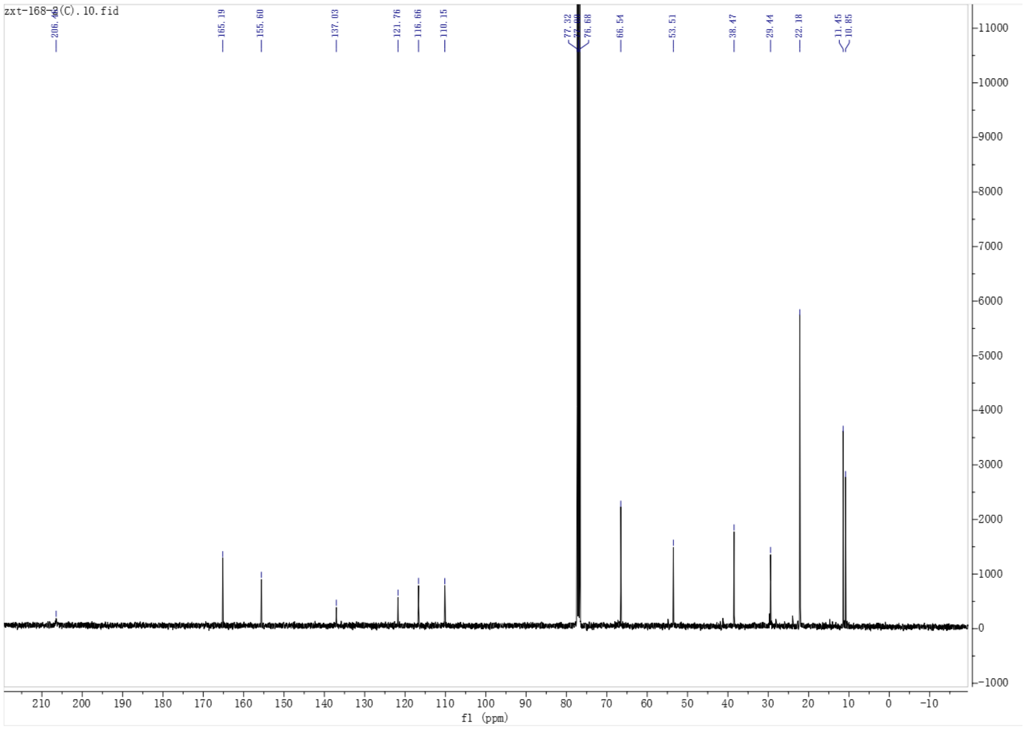

165.19
155.60
137.03
121.76
116.66
110.15
77.32
76.68
66.54
53.51
38.47
29.44
22.18
11.45
10.85
f1 (ppm)

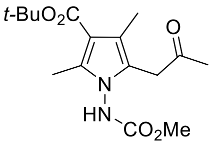

3d

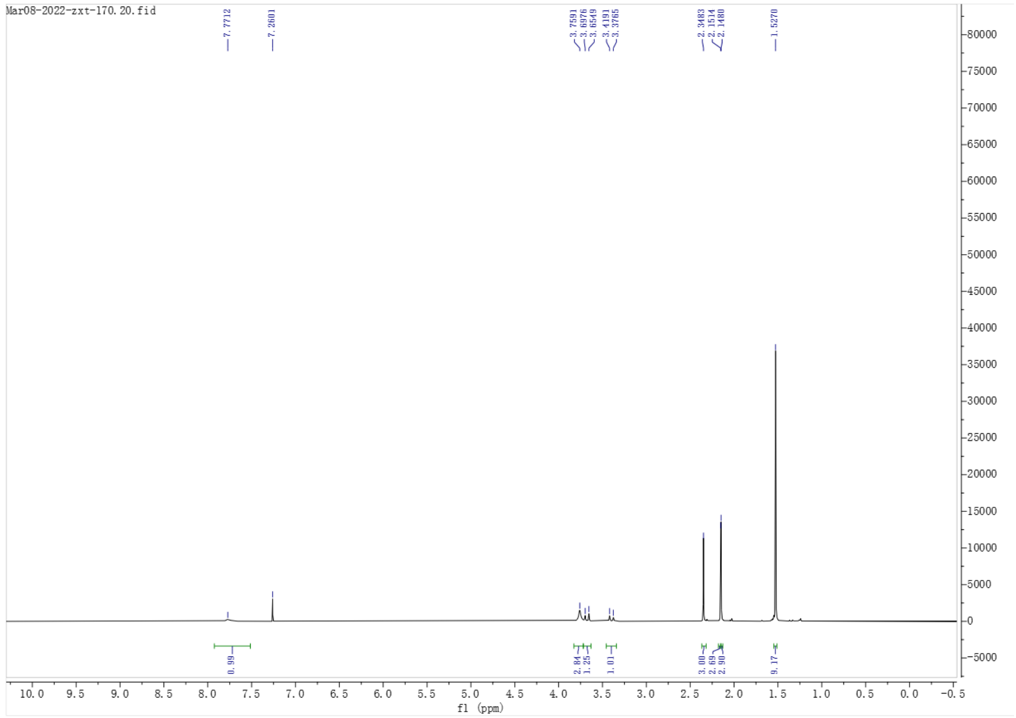
Mar08-2022-zxt-170.20.fid
7.7712
7.2601
3.7591
3.6976
3.6549
3.4191
3.3765
2.3483
2.1514
2.1480
1.5270
0.99
2.84
1.25
1.01
3.00
2.69
2.90
9.17
f1 (ppm)

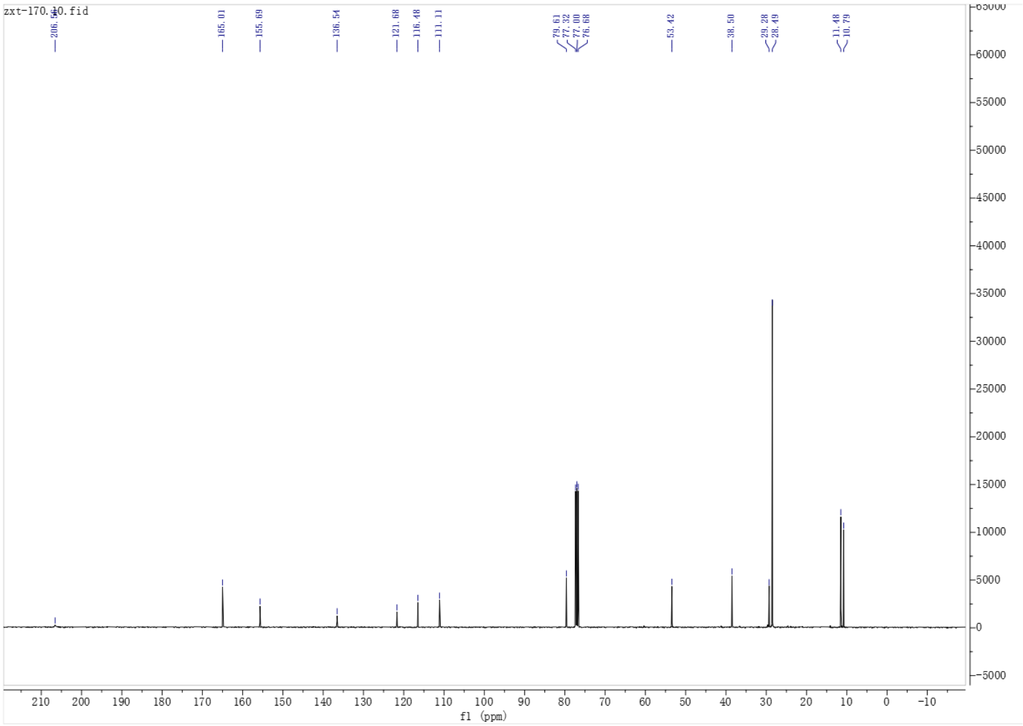
165.01
155.69
136.54
121.68
116.48
111.11
79.61
77.32
77.00
76.68
53.42
38.50
29.28
28.49
11.48
10.79
f1 (ppm)

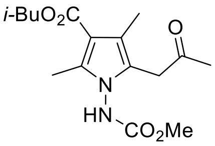
i-BuO2C
O
N
HN
CO2Me

3e

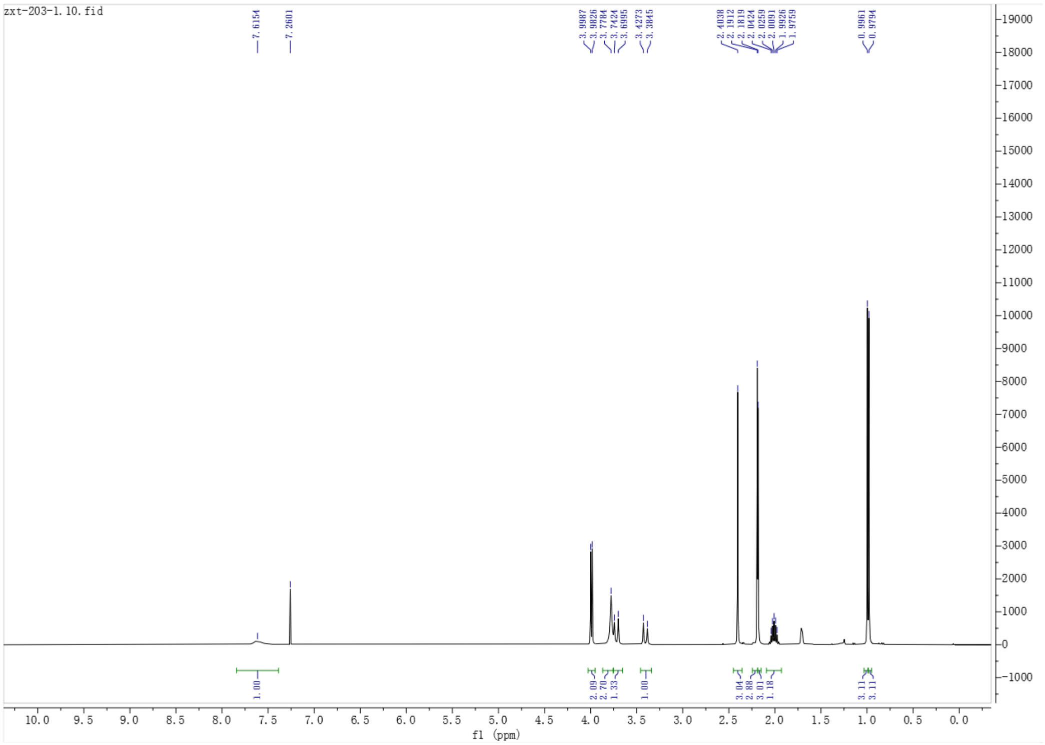
zxt-203-1.10.fid
7.6154
7.2601
3.9987
3.9826
3.7784
3.7424
3.6995
3.4273
3.3845
2.4038
2.1912
2.1819
2.0424
2.0259
2.0091
1.9926
1.9759
0.9961
0.9794
1.00
2.09
2.70
1.33
1.00
3.04
2.88
3.01
1.18
3.11
3.11
f1 (ppm)

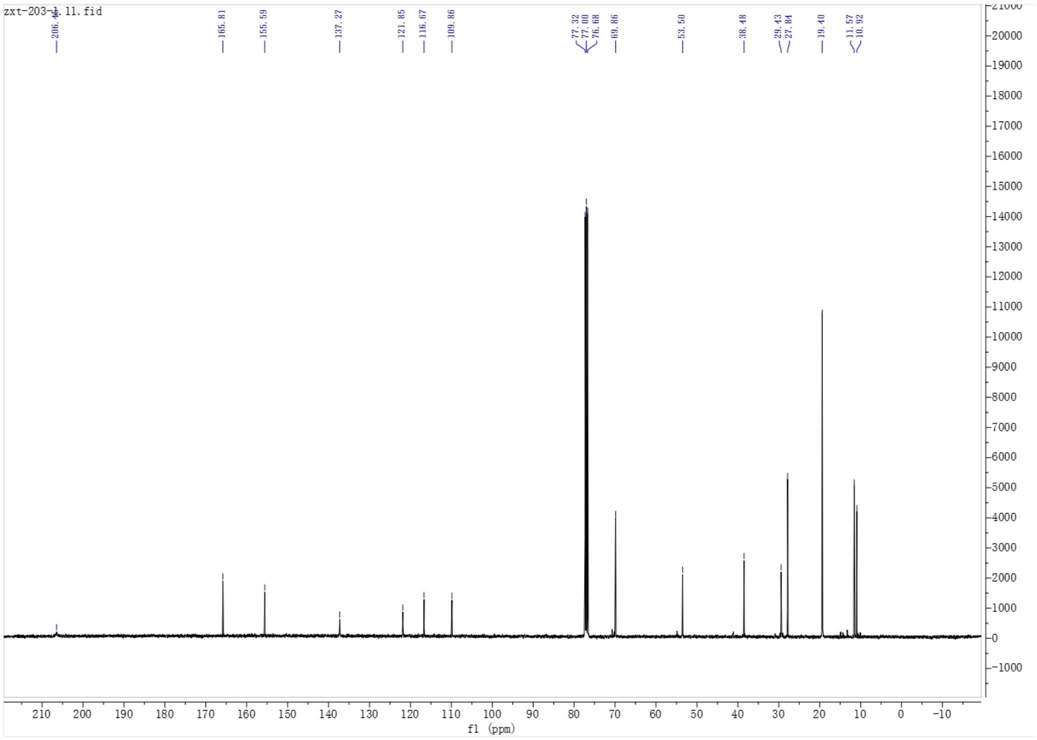
zxt-203-1.11.fid
206
165.81
155.59
137.27
121.85
116.67
109.86
77.32
77.00
76.68
69.86
53.50
38.48
29.43
27.84
19.40
11.57
10.92
f1 (ppm)

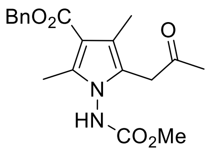

3f

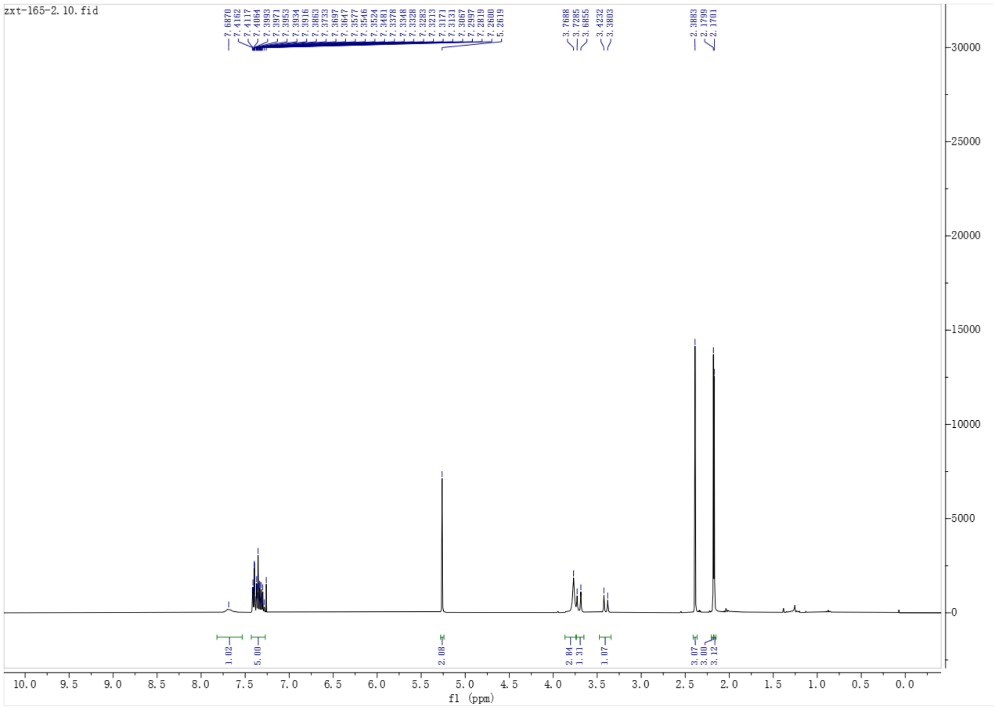

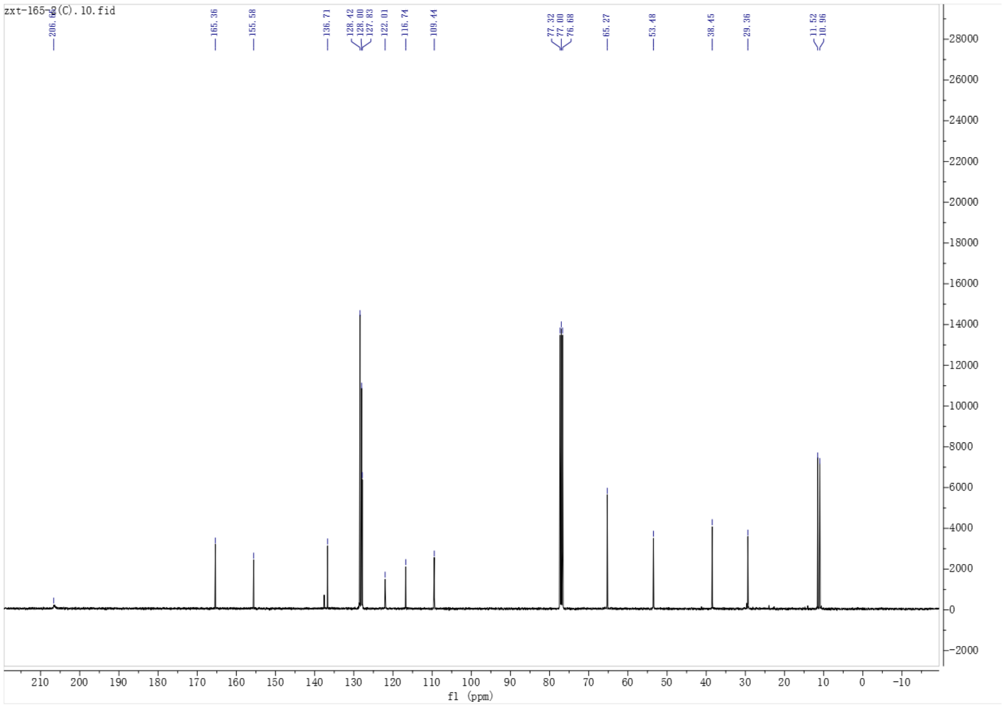

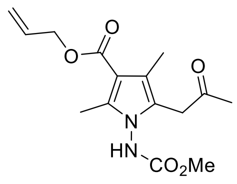

3g

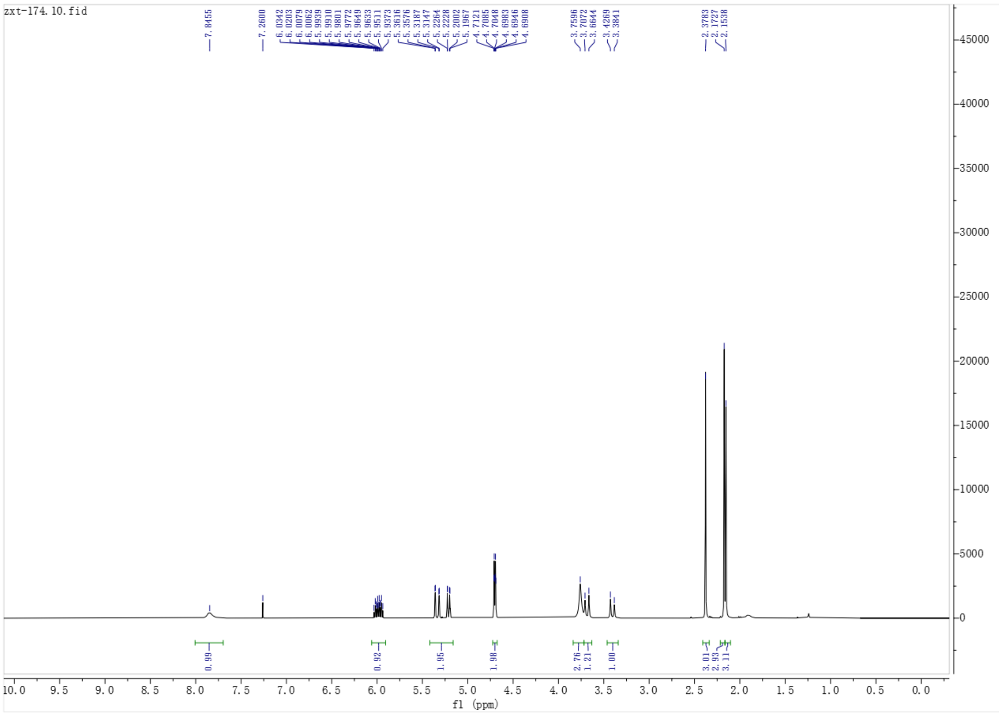

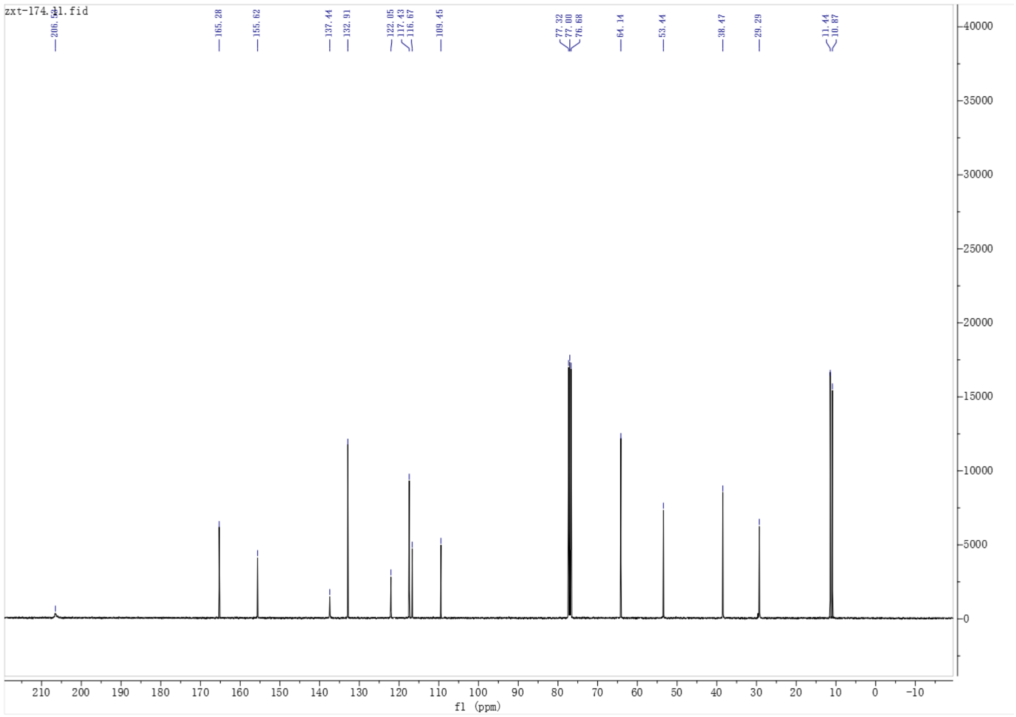

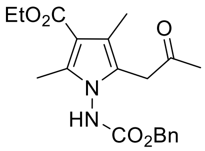
EtO2C
O
N
HN
CO2Bn

3h

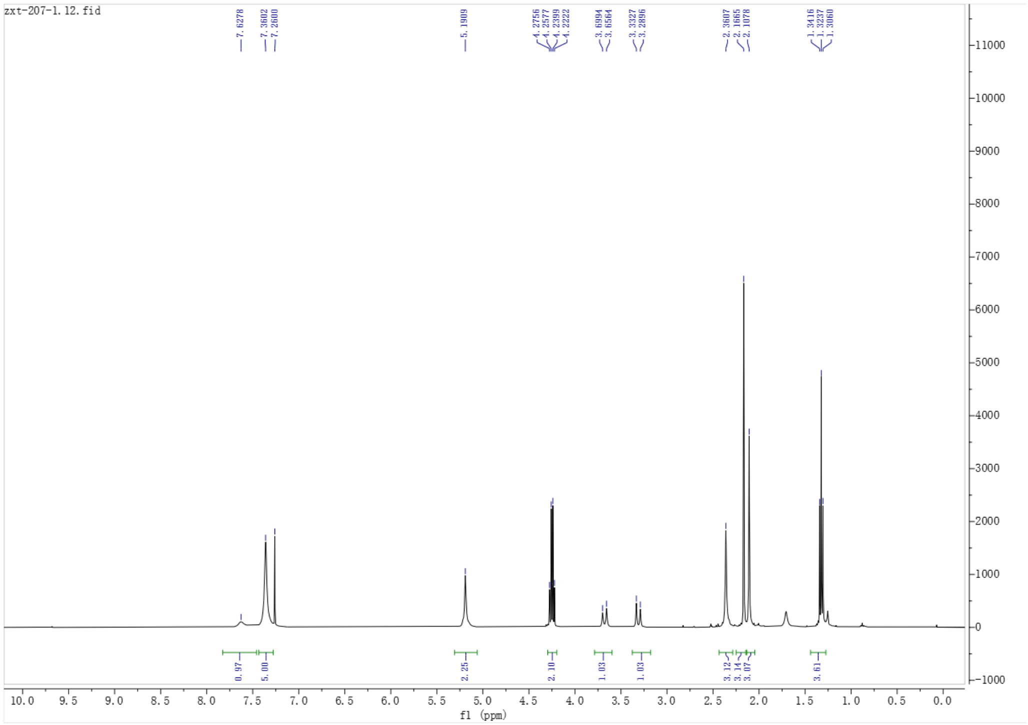
zxt-207-1.12.fid
7.6278
7.3602
7.2600
5.1909
4.2756
4.2577
4.2399
4.2222
3.6994
3.6564
3.3327
3.2896
2.3607
2.1665
2.1078
1.3416
1.3237
1.3060
0.97
5.00
2.25
2.10
1.03
1.03
3.12
3.14
3.07
3.61
f1 (ppm)

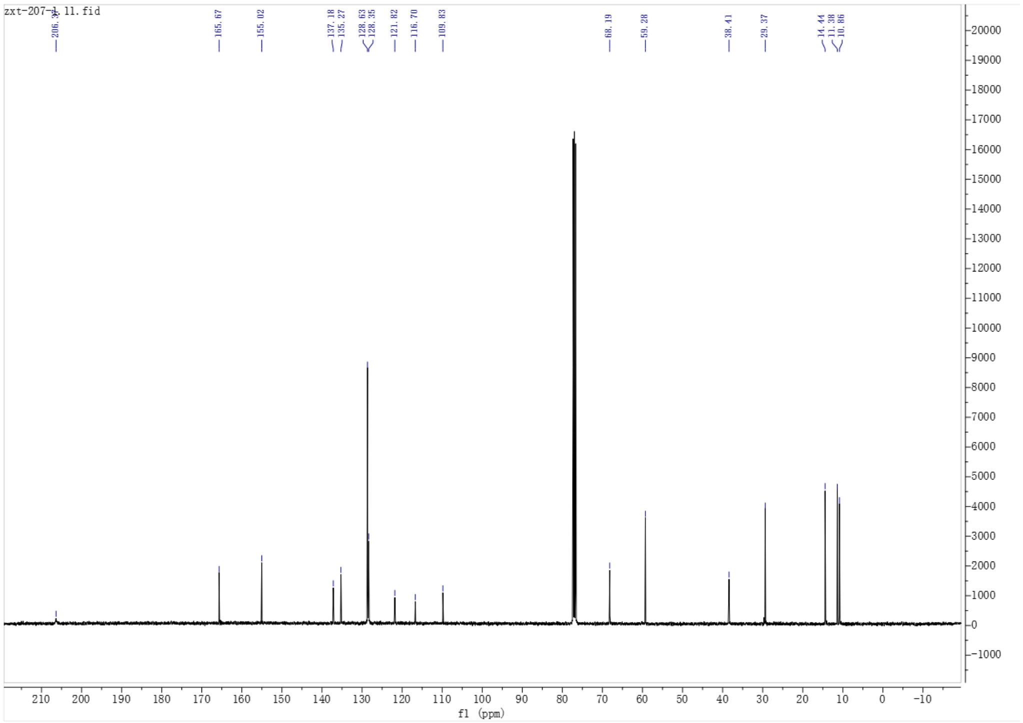
zxt-207-1.11.fid
165.67
155.02
137.18
135.27
128.63
128.35
121.82
116.70
109.83
68.19
59.28
38.41
29.37
14.44
11.38
10.86
f1 (ppm)

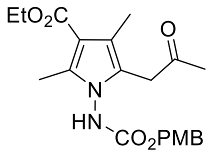

3i

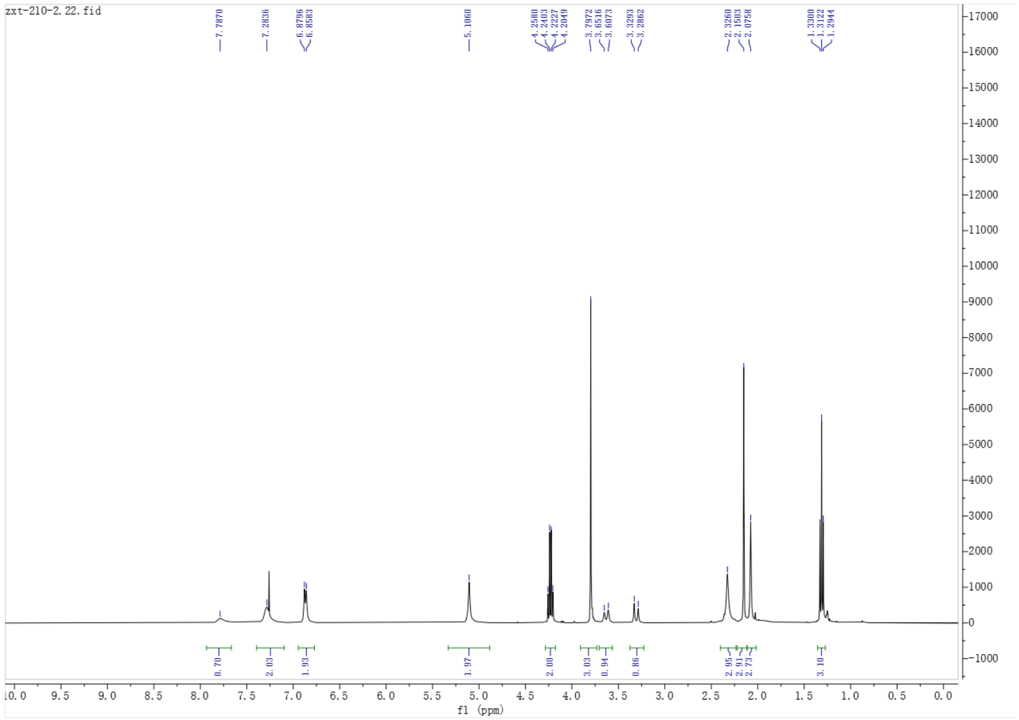

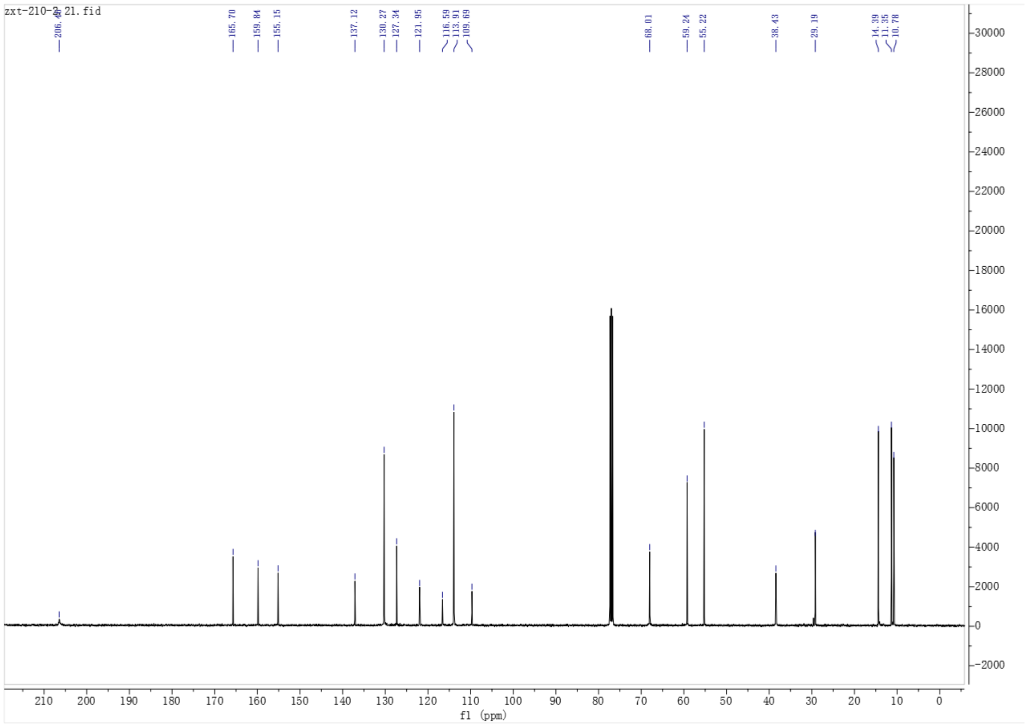

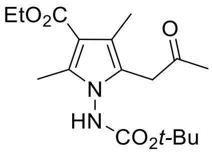
EtO2C
O
N
HN
CO2t-Bu

3j

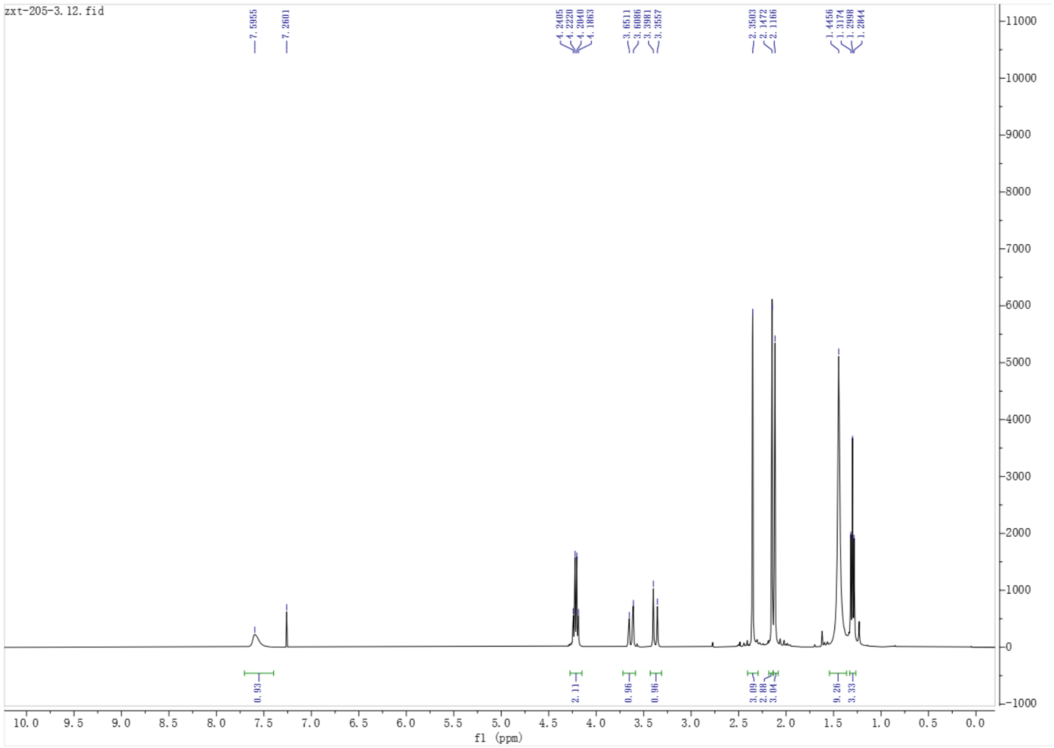
zxt-205-3.12.fid
7.5955
7.2601
4.2405
4.2220
4.2040
4.1863
3.6511
3.6086
3.3981
3.3557
2.3503
2.1472
2.1166
1.4456
1.3174
1.2998
1.2844
0.93
2.11
0.96
0.96
3.09
2.88
3.04
9.26
3.33
f1 (ppm)

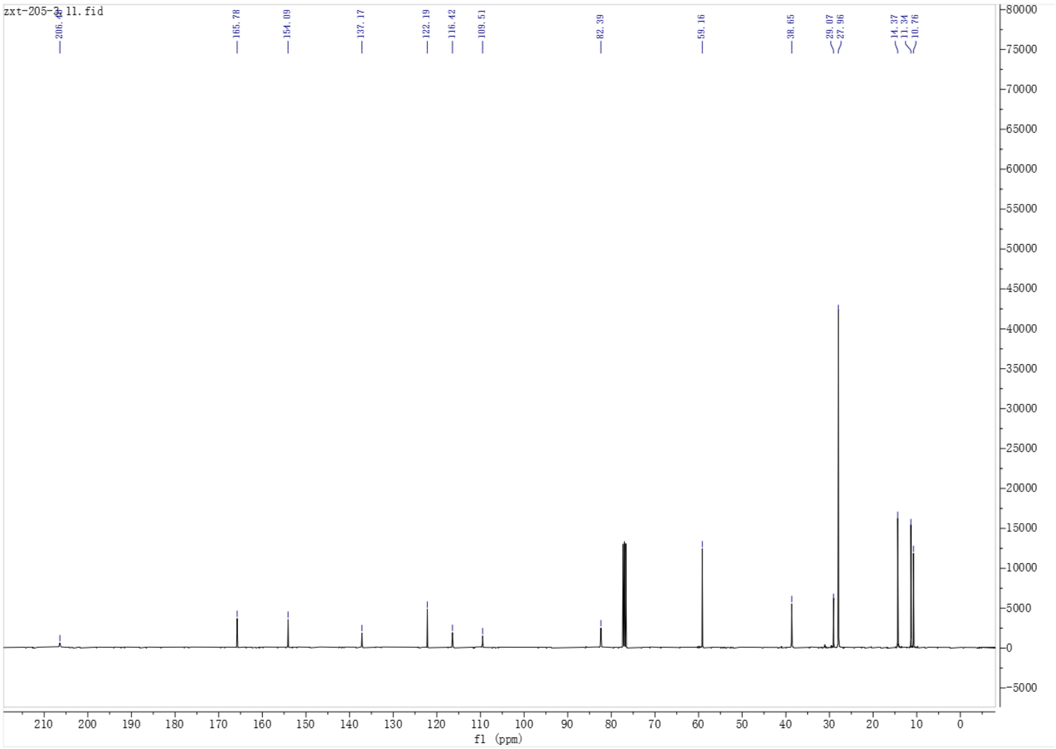
zxt-205-3.11.fid
165.78
154.09
137.17
122.19
116.42
109.51
82.39
59.16
38.65
29.07
27.96
14.37
11.34
10.76
f1 (ppm)

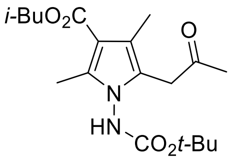

i-BuO2C
O
N
HN
CO2t-Bu

3k

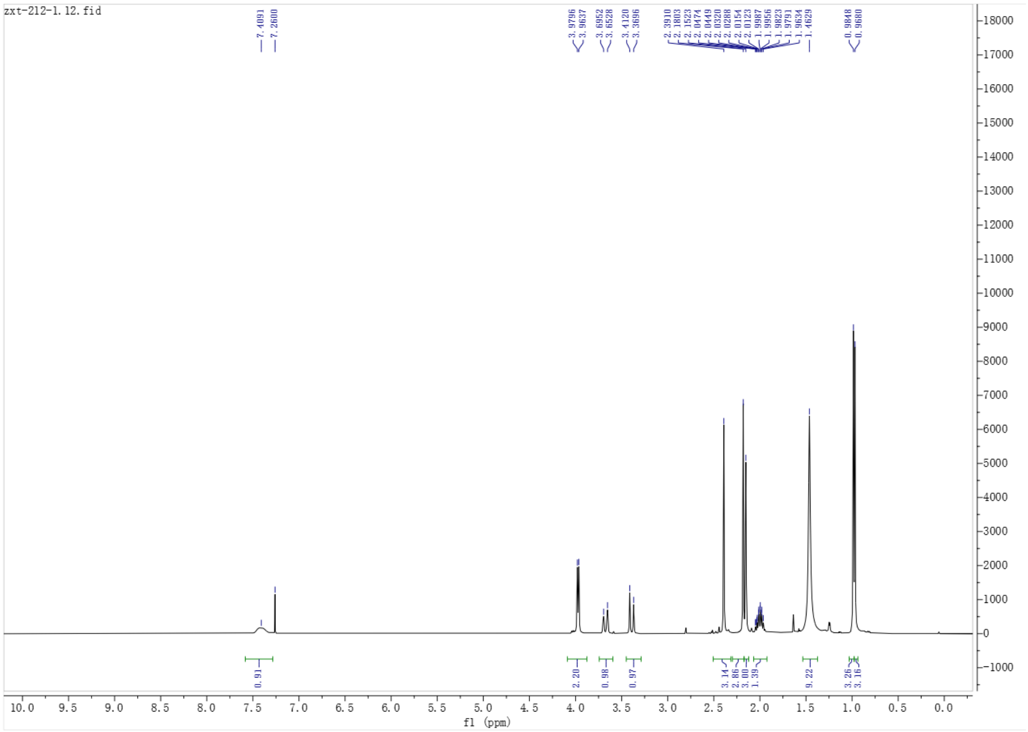

zxt-212-1.12.fid
7.4091
7.2600
3.9796
3.9637
3.6952
3.6528
3.4120
3.3696
2.3910
2.1803
2.1523
2.0474
2.0449
2.0320
2.0288
2.0154
2.0123
1.9987
1.9956
1.9823
1.9791
1.9634
1.4629
0.9848
0.9680
0.91
2.20
0.98
0.97
3.14
2.86
3.00
1.39
9.22
3.26
3.16
f1 (ppm)

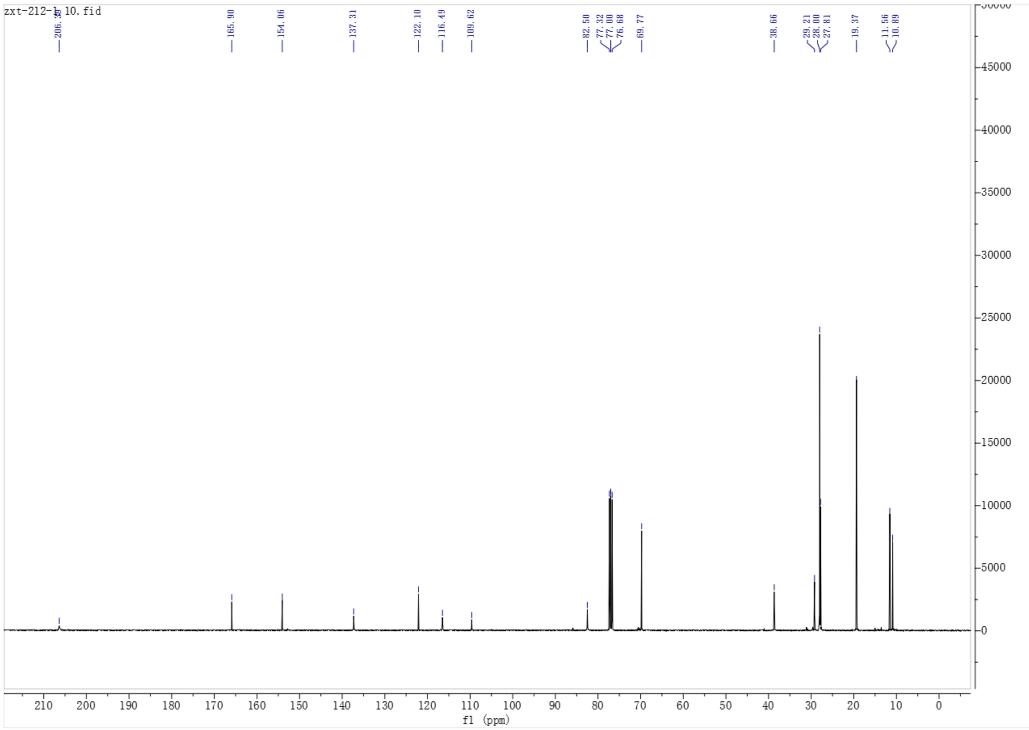

zxt-212-1.10.fid
165.90
154.06
137.31
122.10
116.49
109.62
82.50
77.32
77.00
76.68
69.77
38.66
29.21
28.00
27.81
19.37
11.56
10.89
f1 (ppm)

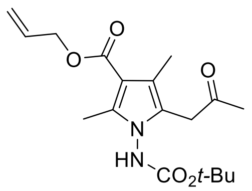

3l

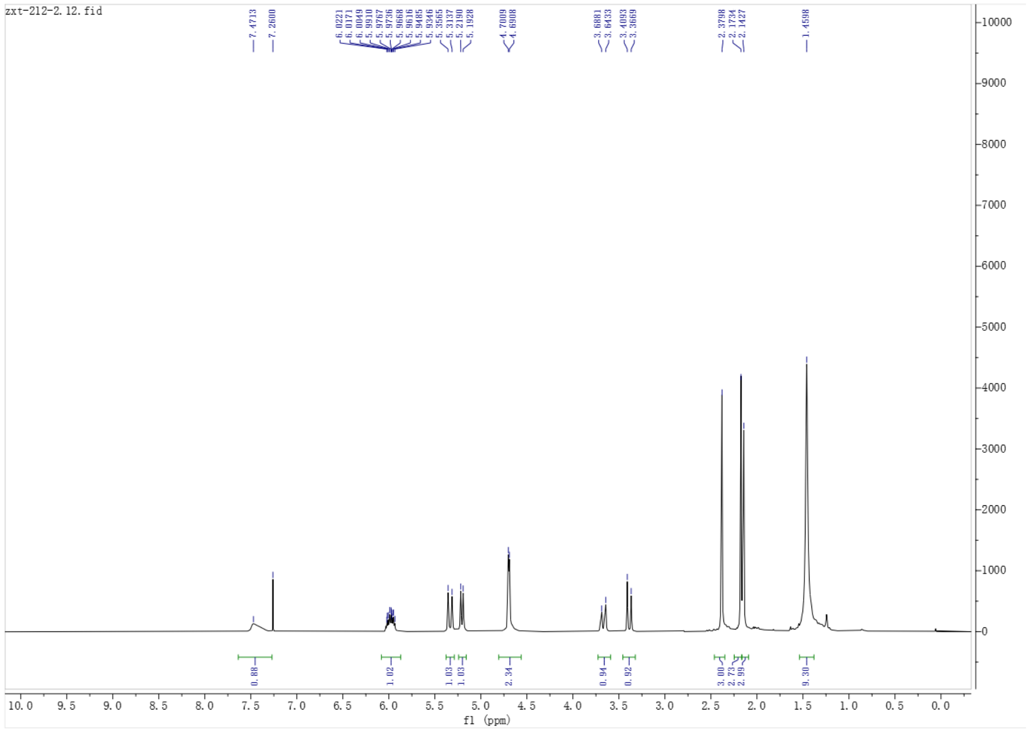

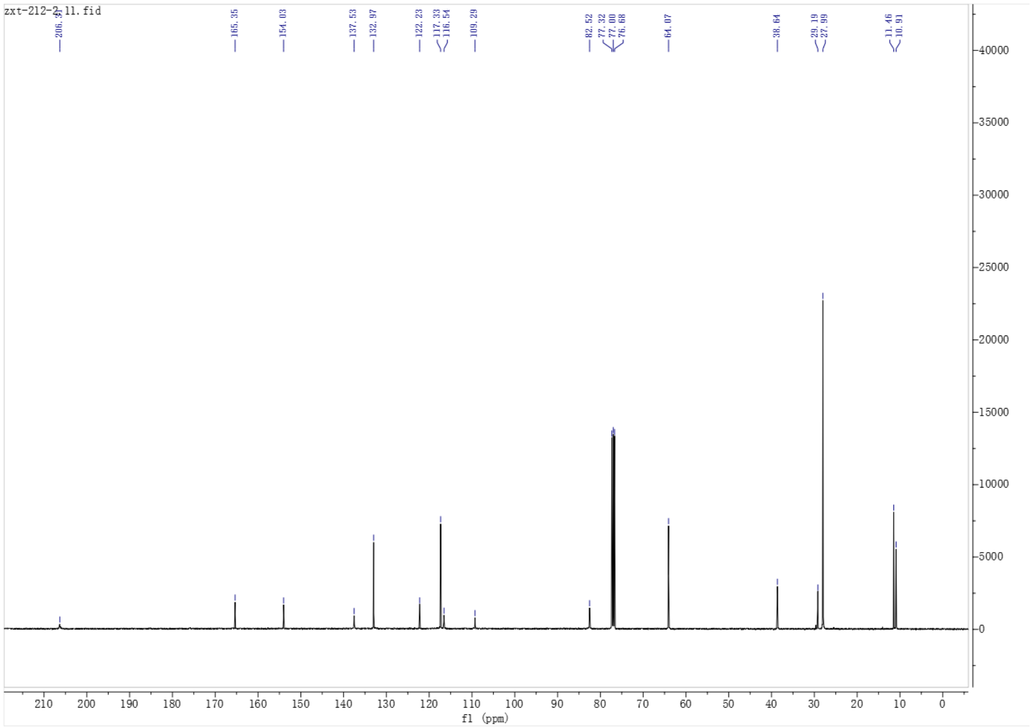

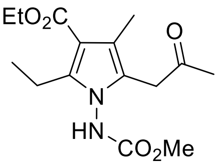
EtO2C
O
N
HN
CO2Me

3m

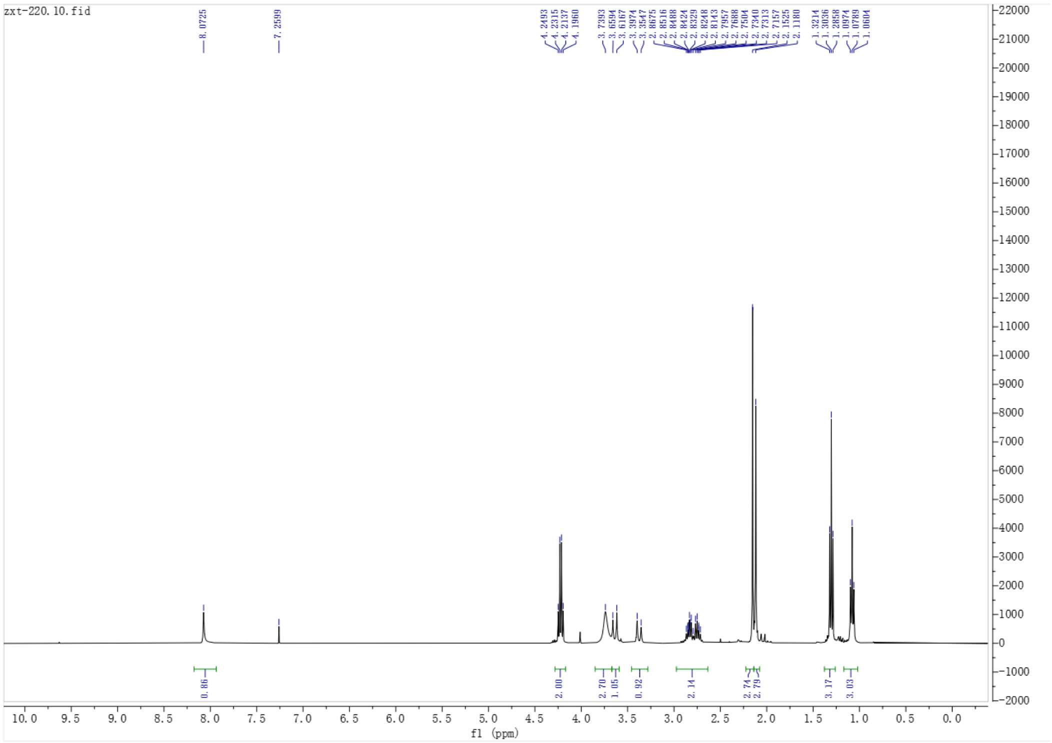
zxt-220.10.fid
8.0725
7.2599
4.2493
4.2315
4.2137
4.1960
0.86
2.00
2.70
1.05
0.92
2.14
2.74
2.79
3.17
3.03
f1 (ppm)

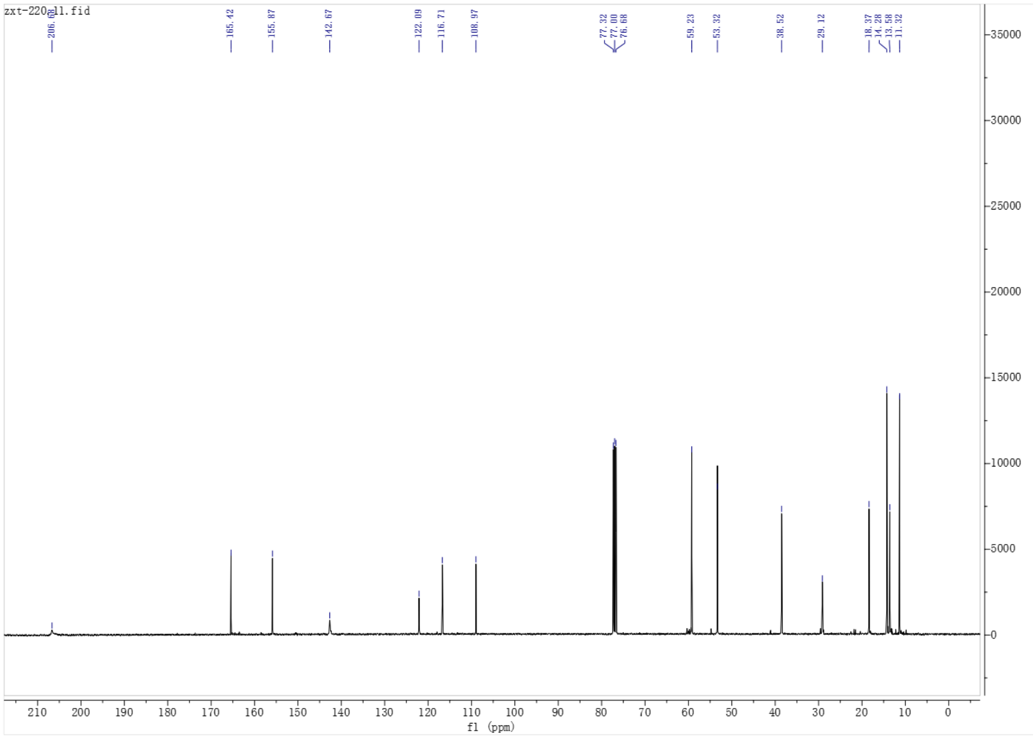
165.42
155.87
142.67
122.09
116.71
108.97
77.32
77.00
76.68
59.23
53.32
38.52
29.12
18.37
14.28
13.58
11.32
f1 (ppm)

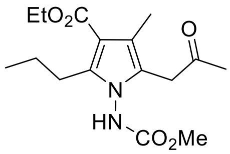
EtO2C
O
N
HN
CO2Me

3n

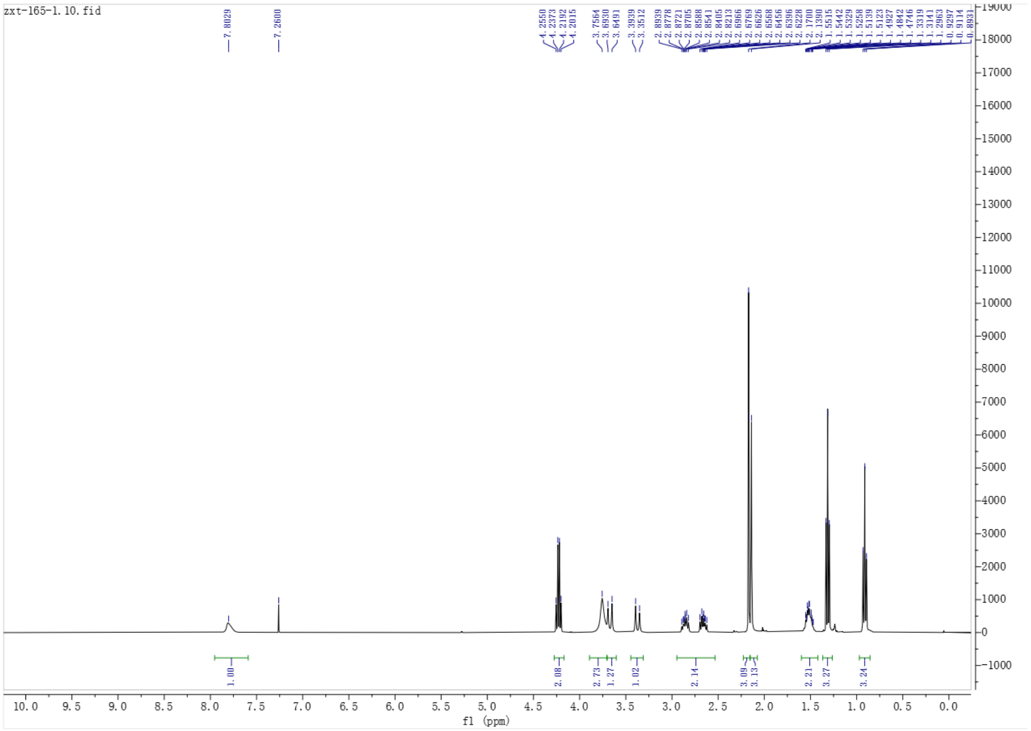
zxt-165-1.10.fid
7.8029
7.2600
4.2550
4.2373
4.2192
4.2015
3.7564
3.6930
3.6491
3.3939
3.3512
1.00
2.08
2.73
1.27
1.02
2.14
3.09
3.13
2.21
3.27
3.24
f1 (ppm)

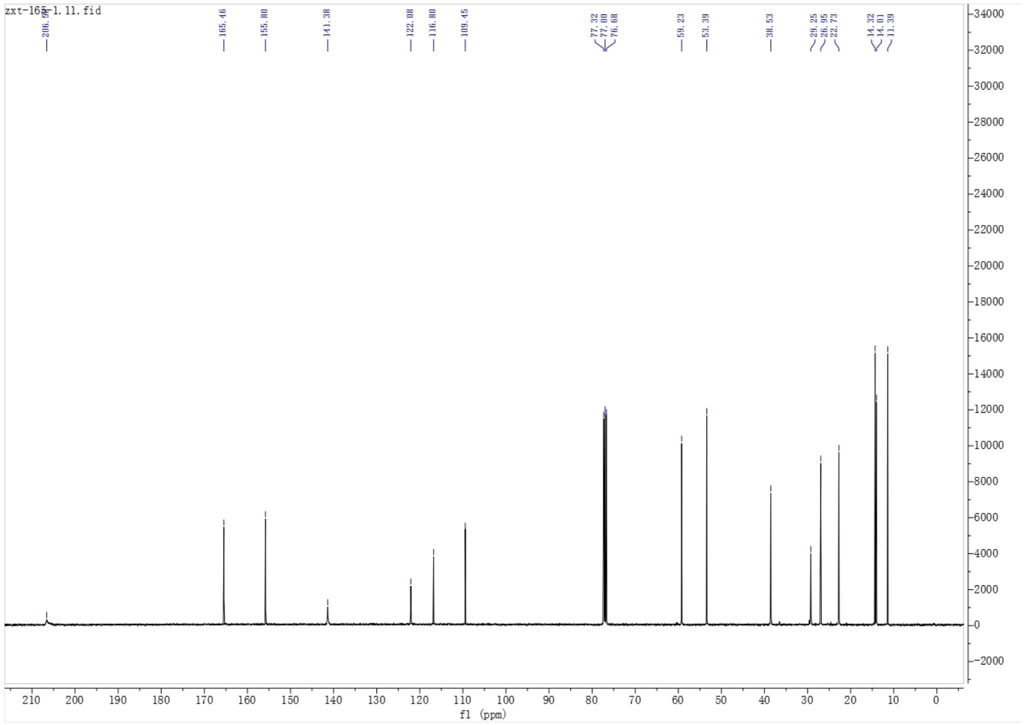
zxt-165-1.11.fid
165.46
155.80
141.38
122.08
116.80
109.45
77.32
77.00
76.68
59.23
53.39
38.53
29.25
26.95
22.73
14.32
14.01
11.39
f1 (ppm)

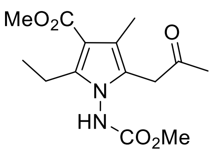
MeO2C
O
N
HN
CO2Me

3o

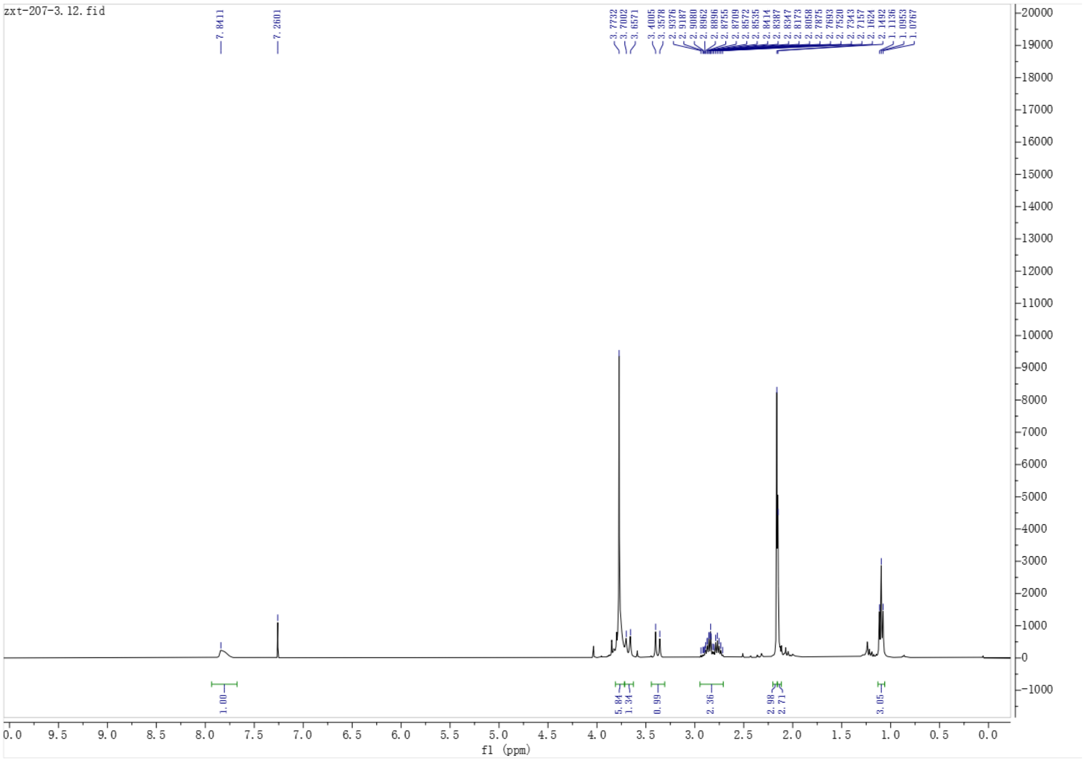
zxt-207-3.12.fid
7.8411
7.2601
f1 (ppm)

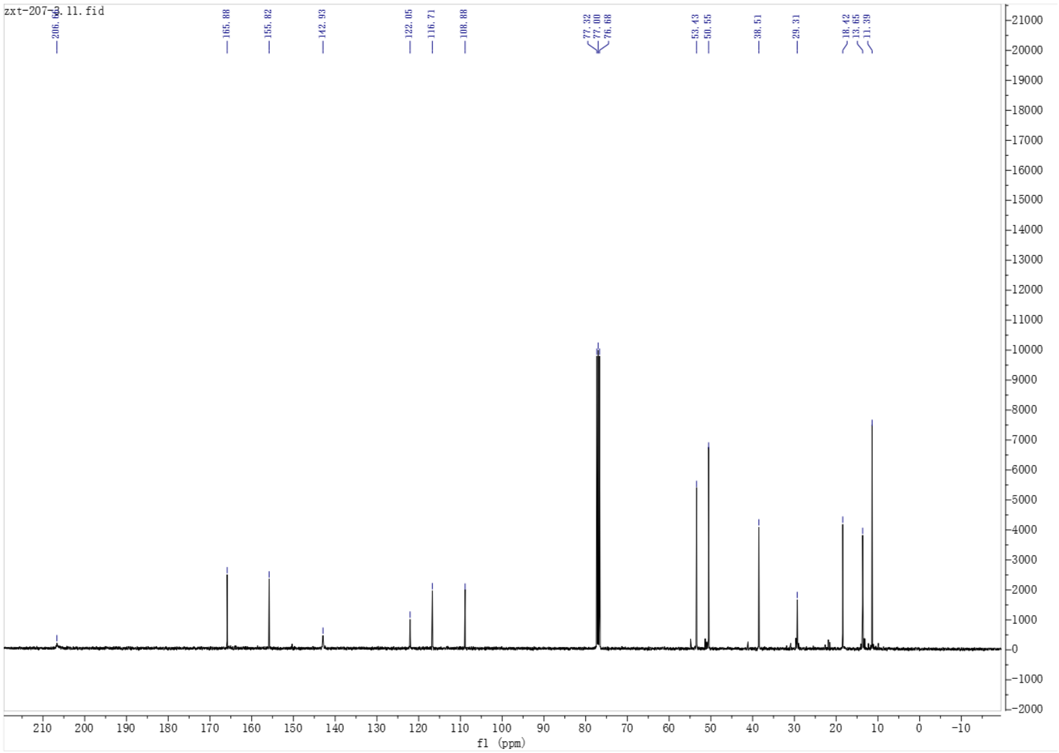
zxt-207-3.11.fid
165.88
155.82
142.93
122.05
116.71
108.88
77.32
77.00
76.68
53.43
50.55
38.51
29.31
18.42
13.65
11.39
f1 (ppm)

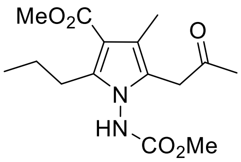

3p

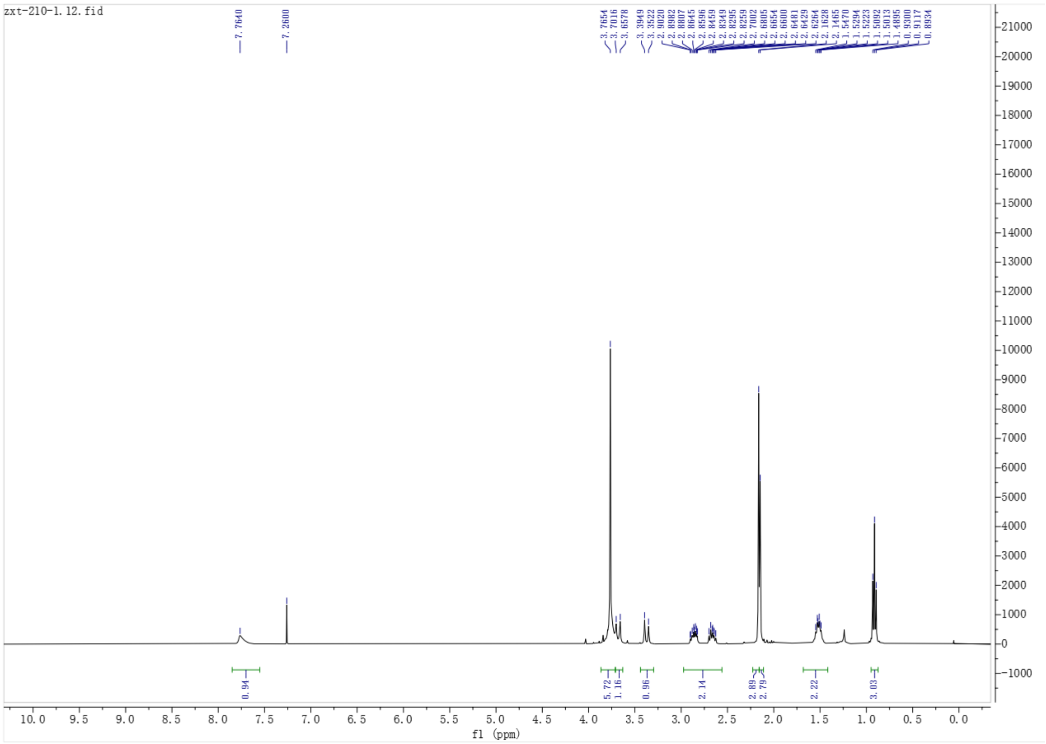

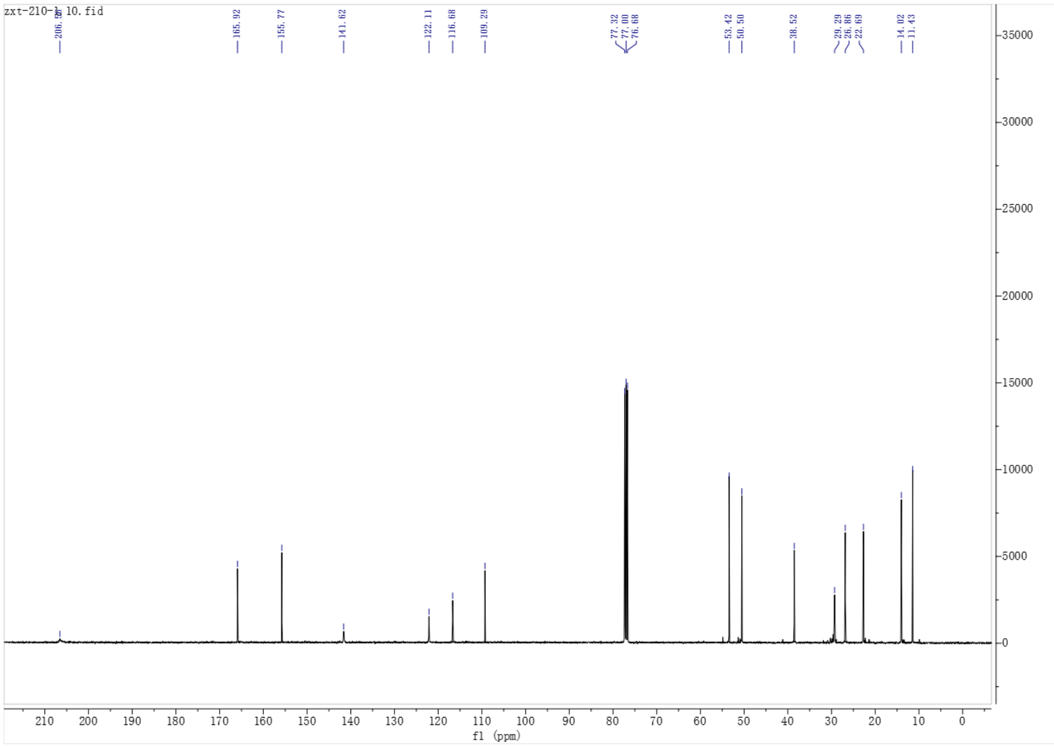

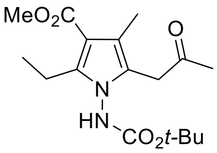
MeO2C
O
N
HN
CO2t-Bu

3q

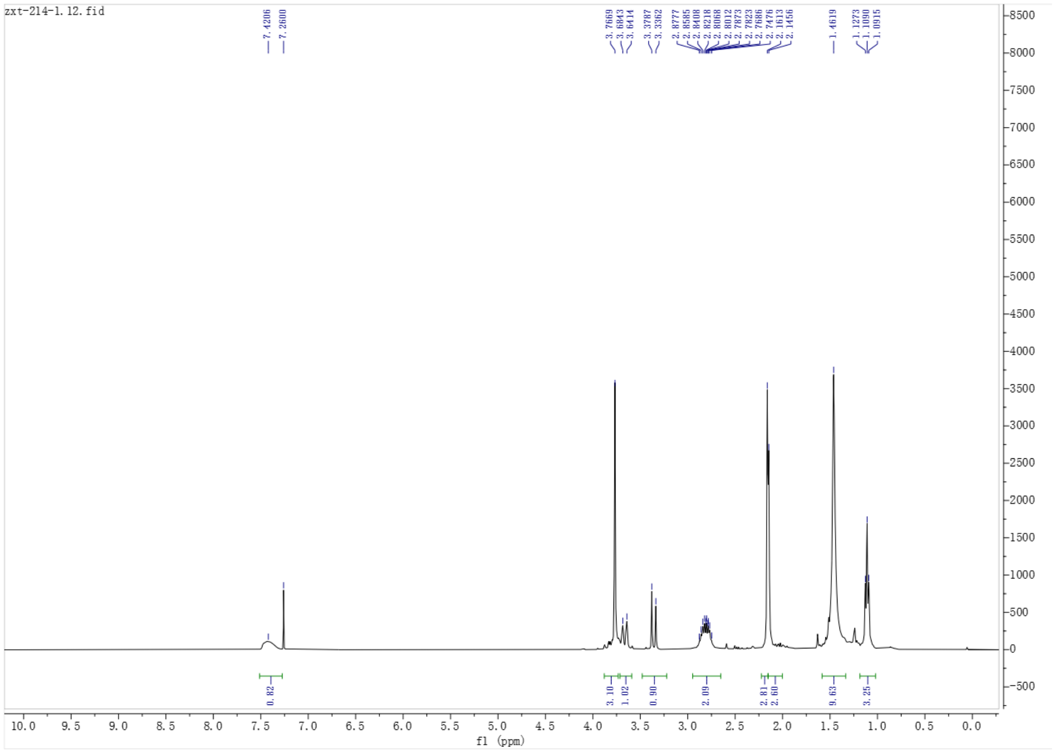
zxt-214-1.12.fid
7.4206
7.2600
3.7669
3.6843
3.6414
3.3787
3.3362
2.8777
2.8585
2.8408
2.8218
2.8068
2.8012
2.7873
2.7823
2.7686
2.7476
2.1613
2.1456
1.4619
1.1273
1.1090
1.0915
0.82
3.10
1.02
0.90
2.09
2.81
2.60
9.63
3.25
f1 (ppm)

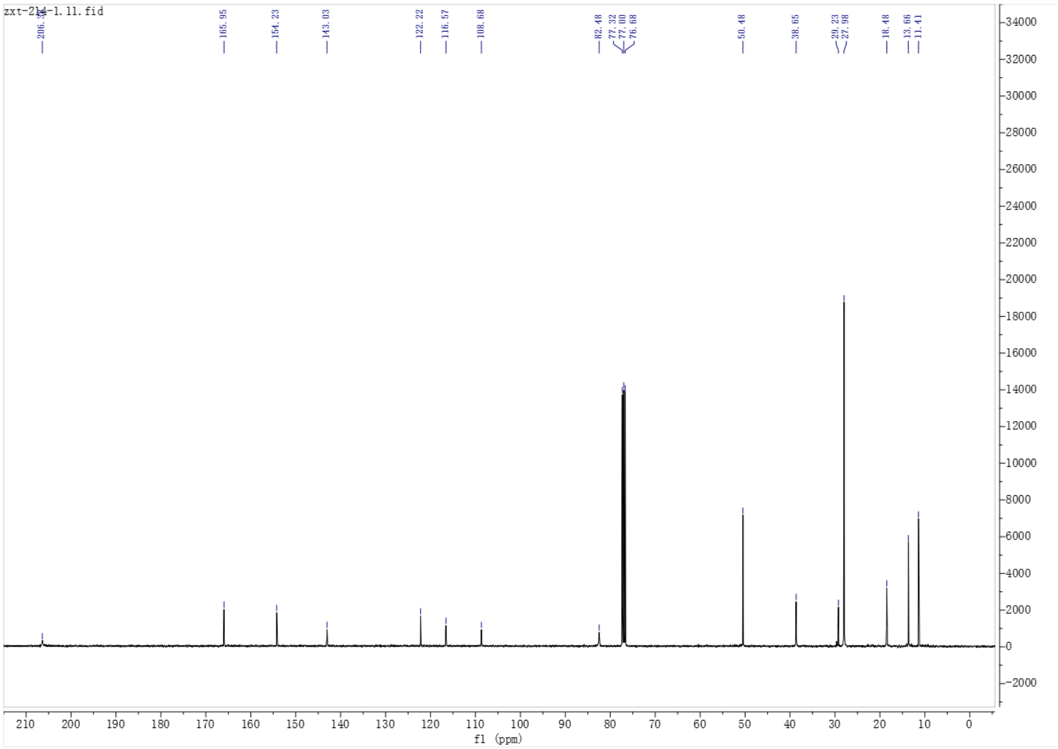
zxt-214-1.11.fid
206.34
165.95
154.23
143.03
122.22
116.57
108.68
82.48
77.32
77.00
76.68
50.48
38.65
29.23
27.98
18.48
13.66
11.41
f1 (ppm)

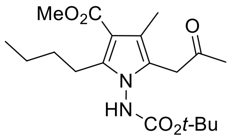
MeO2C
O
N
HN
CO2t-Bu

3r

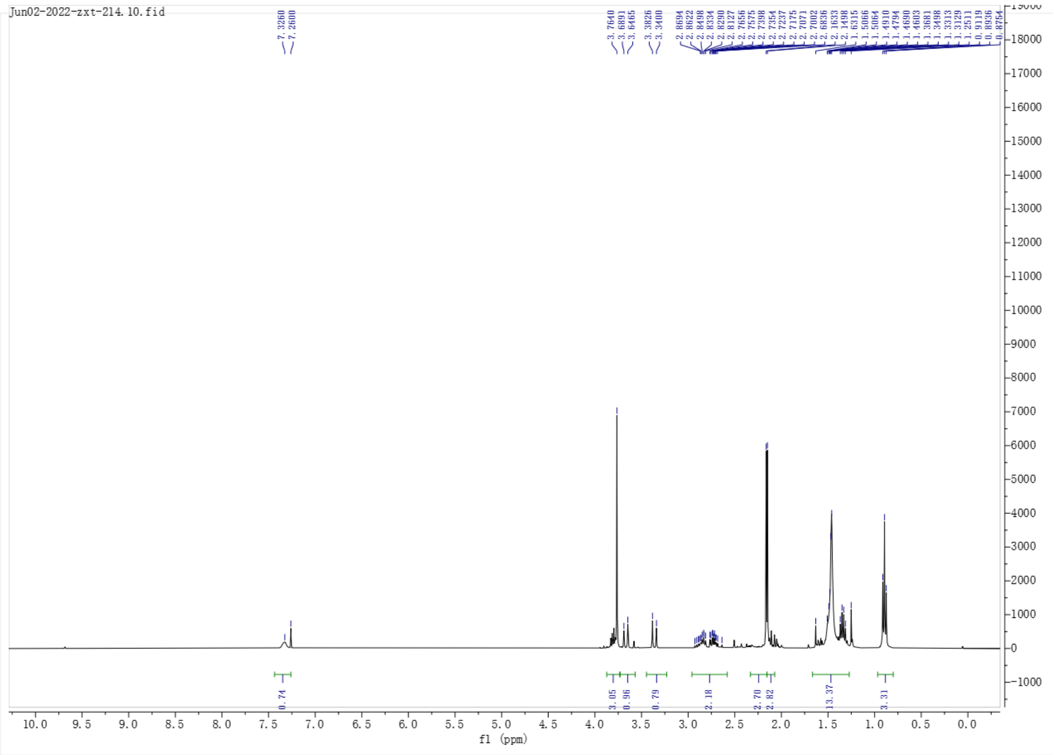
Jun02-2022-zxt-214.10.fid
7.3260
7.2600
3.7640
3.6891
3.6465
3.3826
3.3400
0.74
3.05
0.96
0.79
2.18
2.70
2.82
13.37
3.31
f1 (ppm)

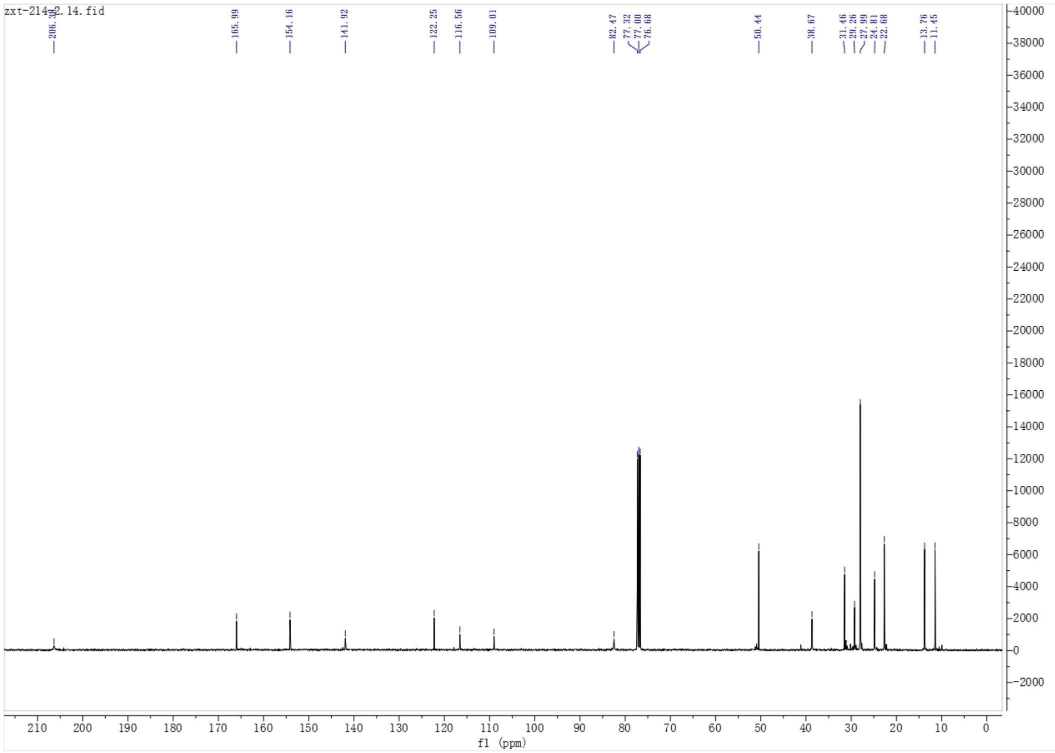
zxt-214-2.14.fid
165.99
154.16
141.92
122.25
116.56
109.01
82.47
77.32
77.00
76.68
50.44
38.67
31.46
29.26
27.99
24.81
22.68
13.76
11.45
f1 (ppm)

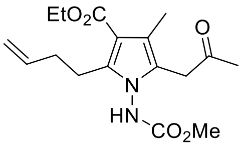
EtO2C
O
N
HN
CO2Me

3s

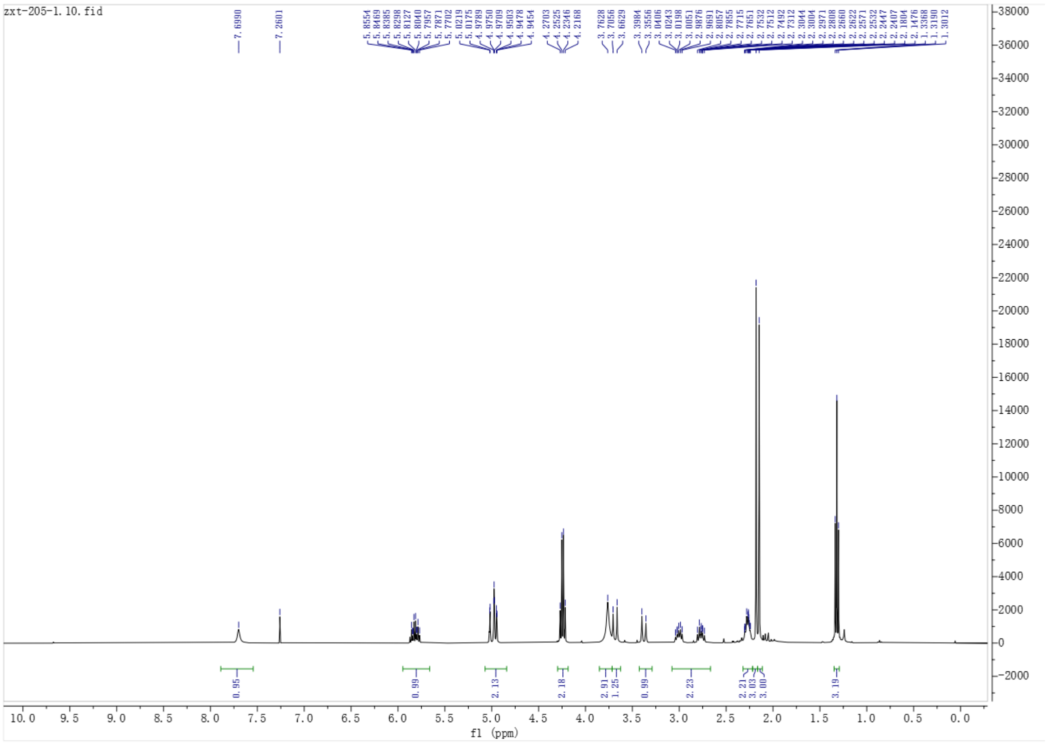
zxt-205-1.10.fid
f1 (ppm)

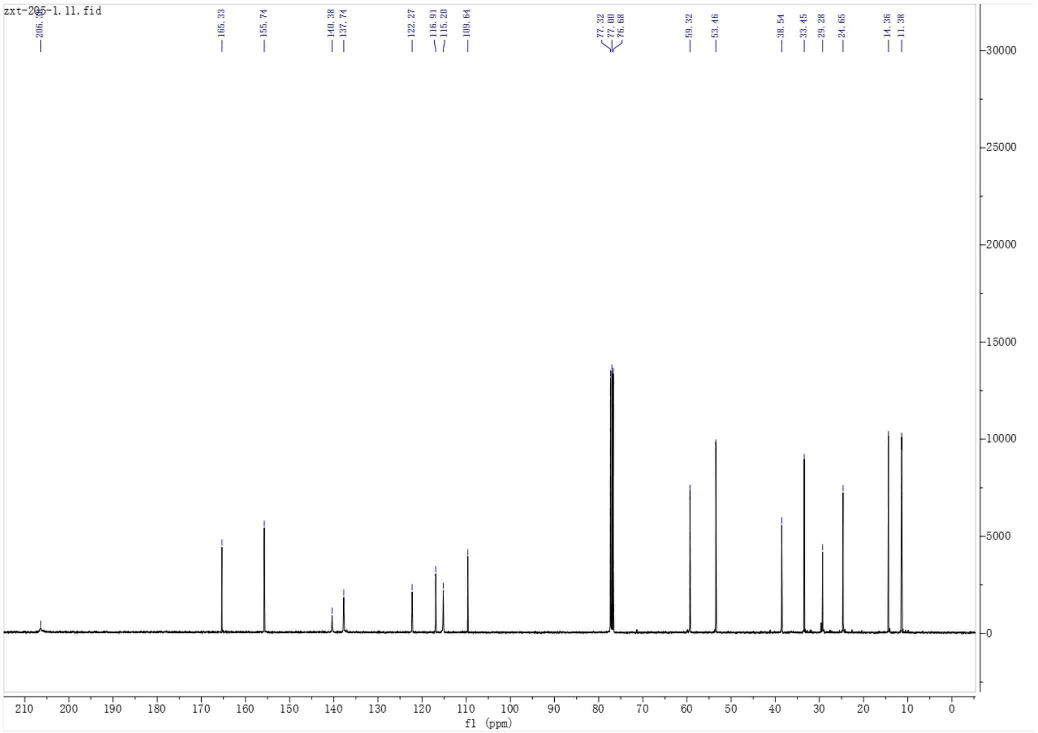
zxt-205-1.11.fid
165.33
155.74
140.38
137.74
122.27
116.91
115.20
109.64
77.32
77.00
76.68
59.32
53.46
38.54
33.45
29.28
24.65
14.36
11.38
f1 (ppm)

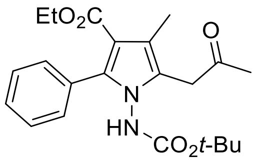
EtO2C
O
N
HN
CO2t-Bu

3t

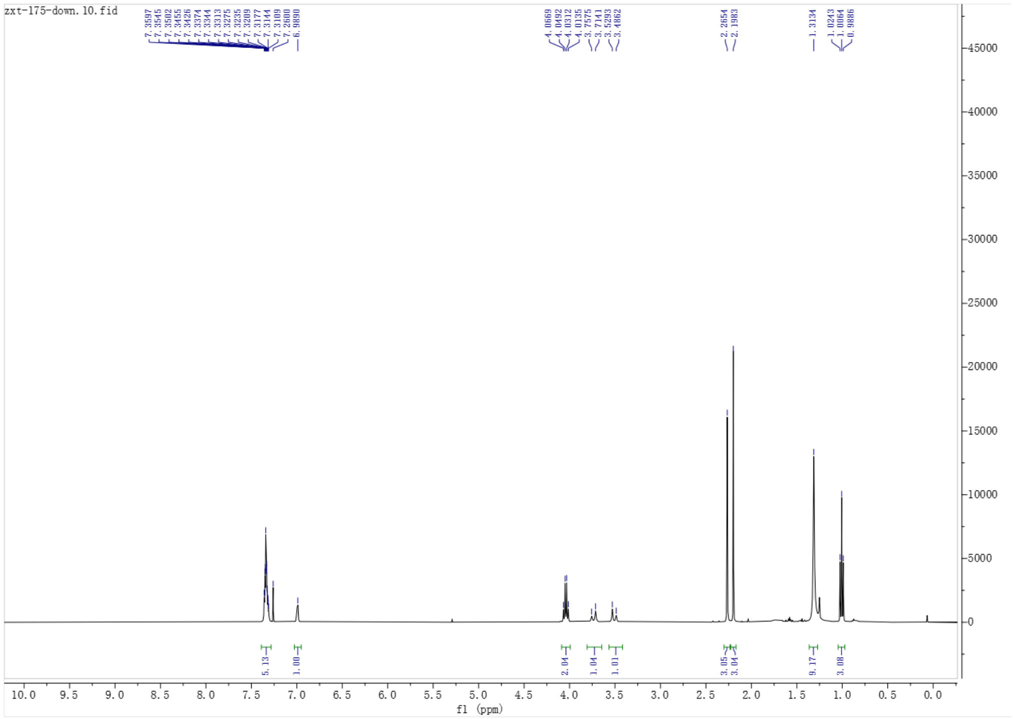
zxt-175-down.10.fid
7.3597
7.3545
7.3502
7.3455
7.3426
7.3374
7.3344
7.3313
7.3275
7.3235
7.3209
7.3177
7.3144
7.3109
7.2600
6.9890
4.0669
4.0492
4.0312
4.0135
3.7575
3.7141
3.5293
3.4862
2.2654
2.1983
1.3134
1.0243
1.0064
0.9886
5.13
1.00
2.04
1.04
1.01
3.05
3.04
9.17
3.08
f1 (ppm)

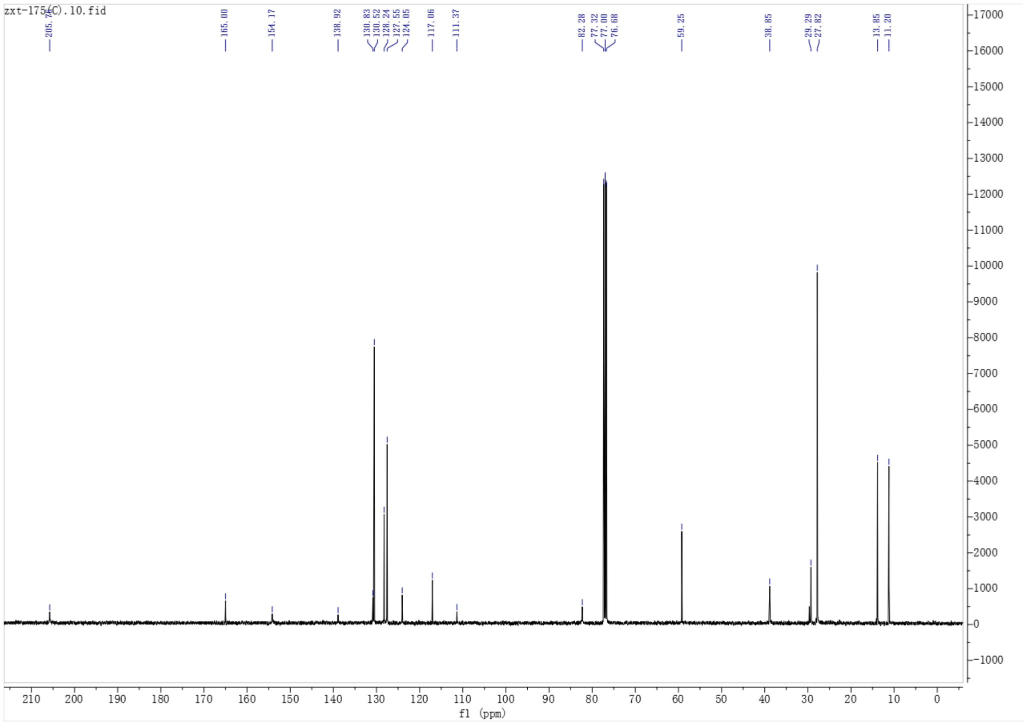
165.00
154.17
138.92
130.83
130.52
128.24
127.55
124.05
117.06
111.37
82.28
77.32
77.00
76.68
59.25
38.85
29.29
27.82
13.85
11.20
f1 (ppm)

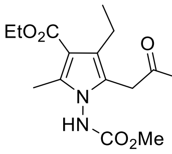
EtO2C
O
N
HN
CO2Me

3ab

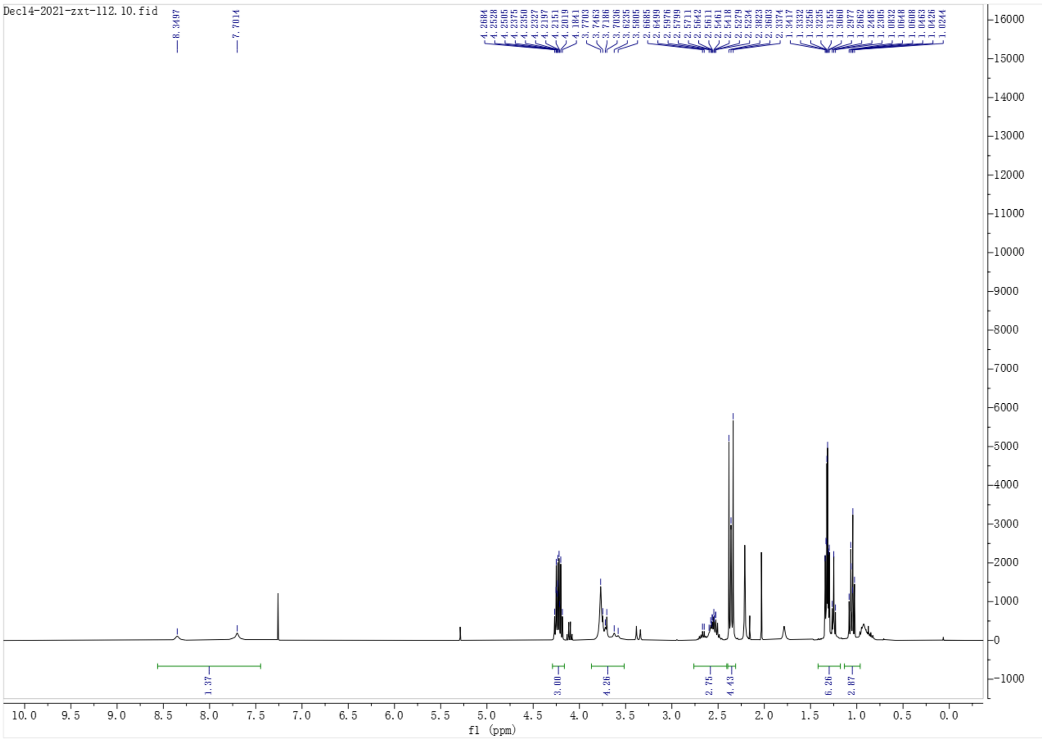
Dec14-2021-zxt-112.10.fid
8.3497
7.7014
1.37
3.00
4.26
2.75
4.43
6.26
2.87
f1 (ppm)

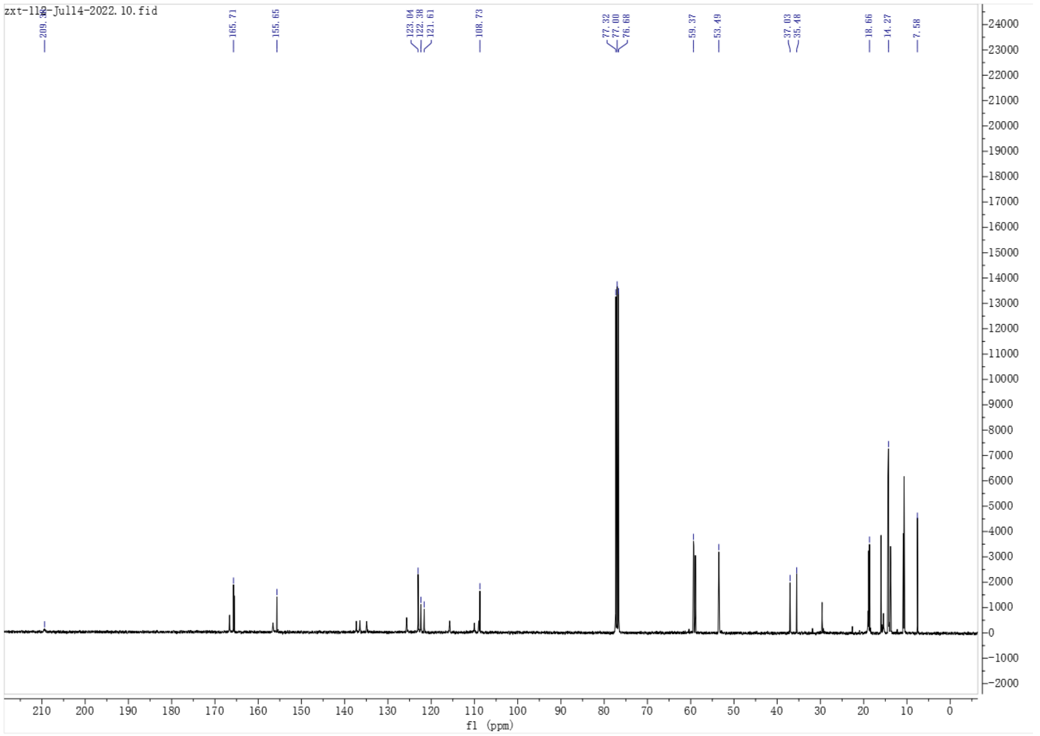
zxt-112-Jul14-2022.10.fid
165.71
155.65
123.04
122.38
121.61
108.73
77.32
77.00
76.68
59.37
53.49
37.03
35.48
18.66
14.27
7.58
f1 (ppm)

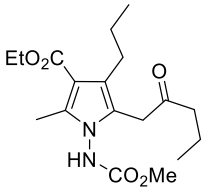

3ac

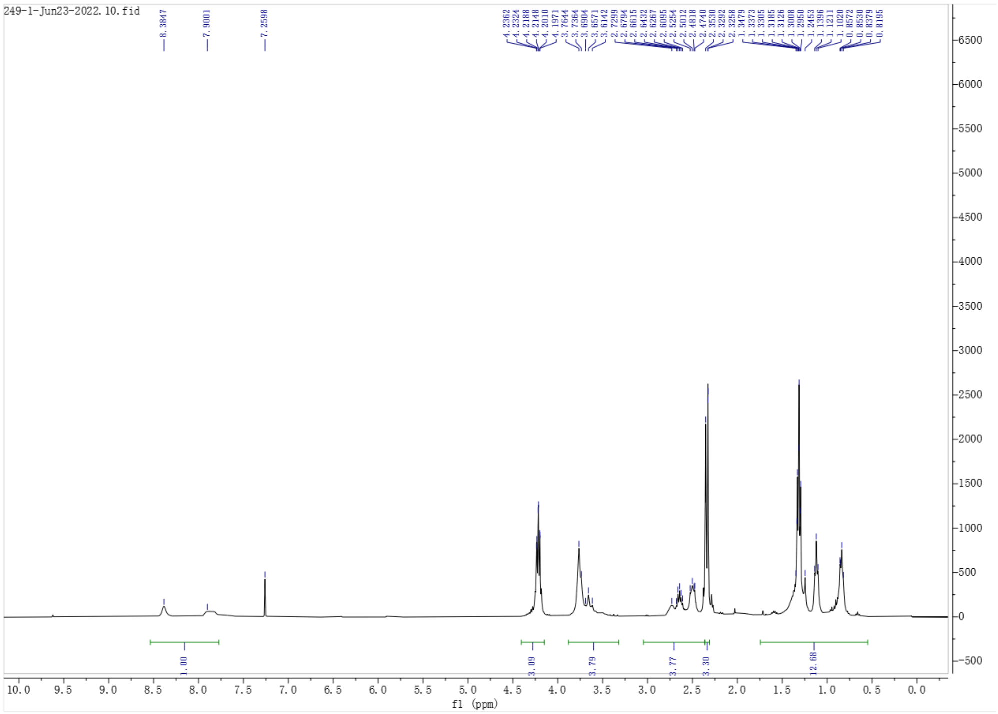

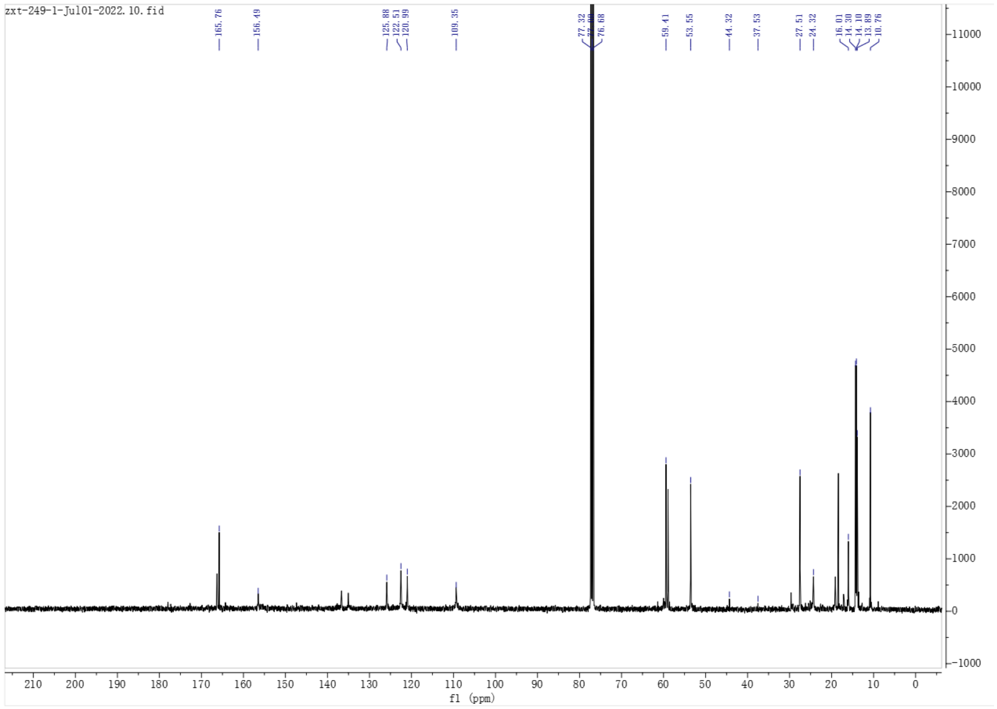

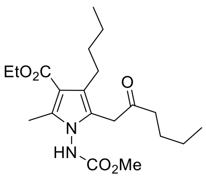

3ad

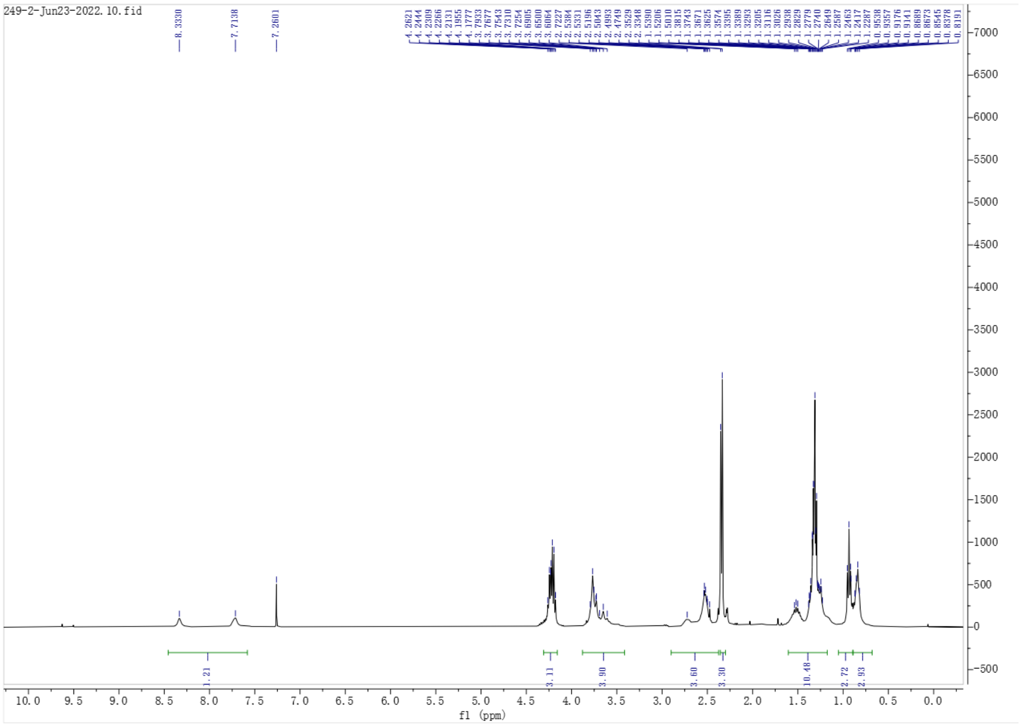

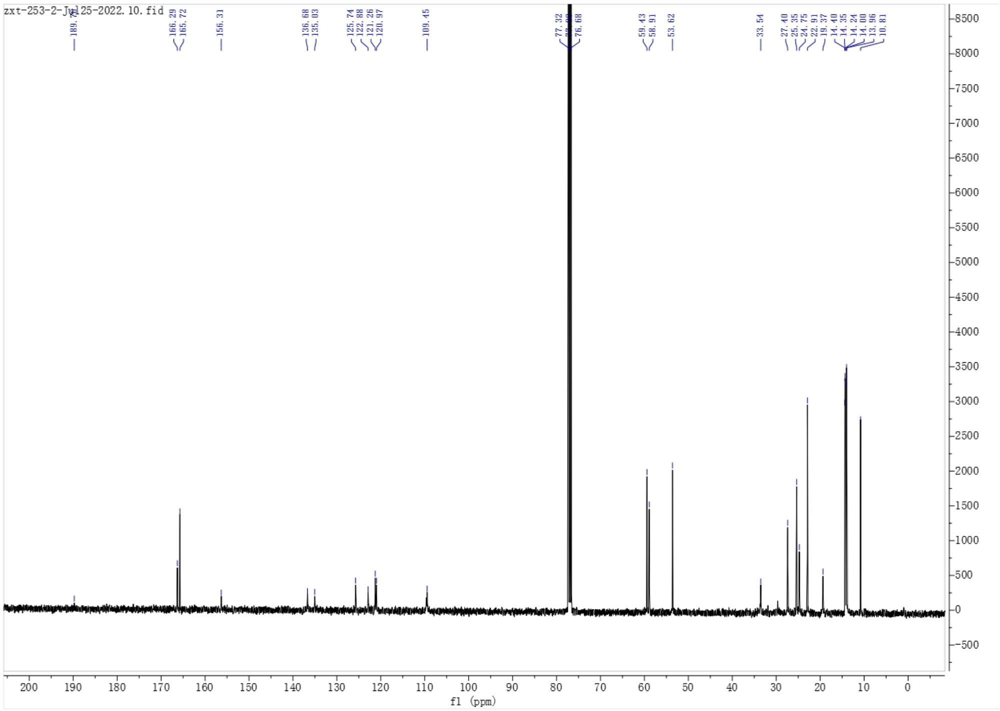

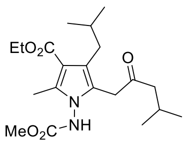

3ae

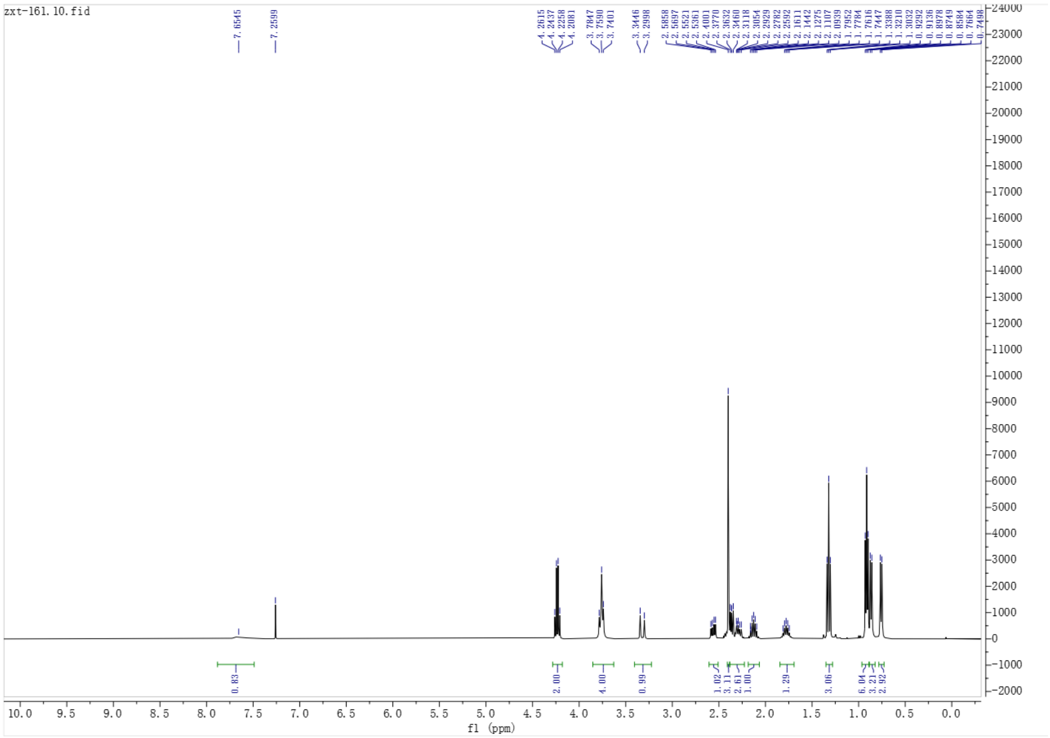

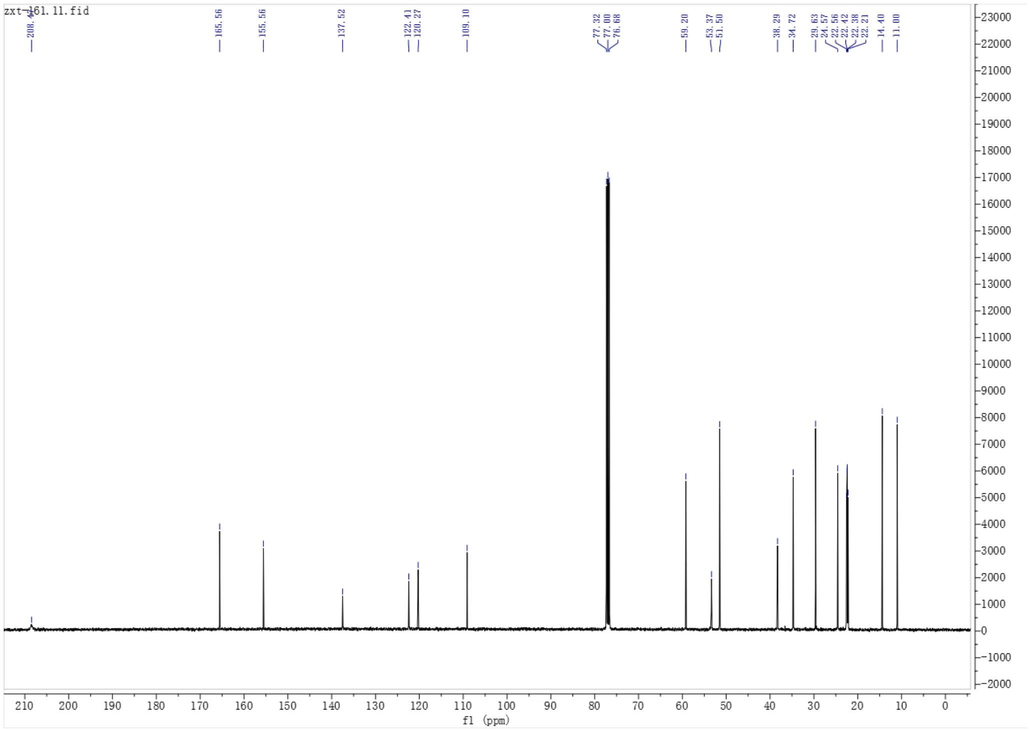

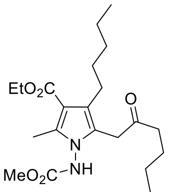

3af

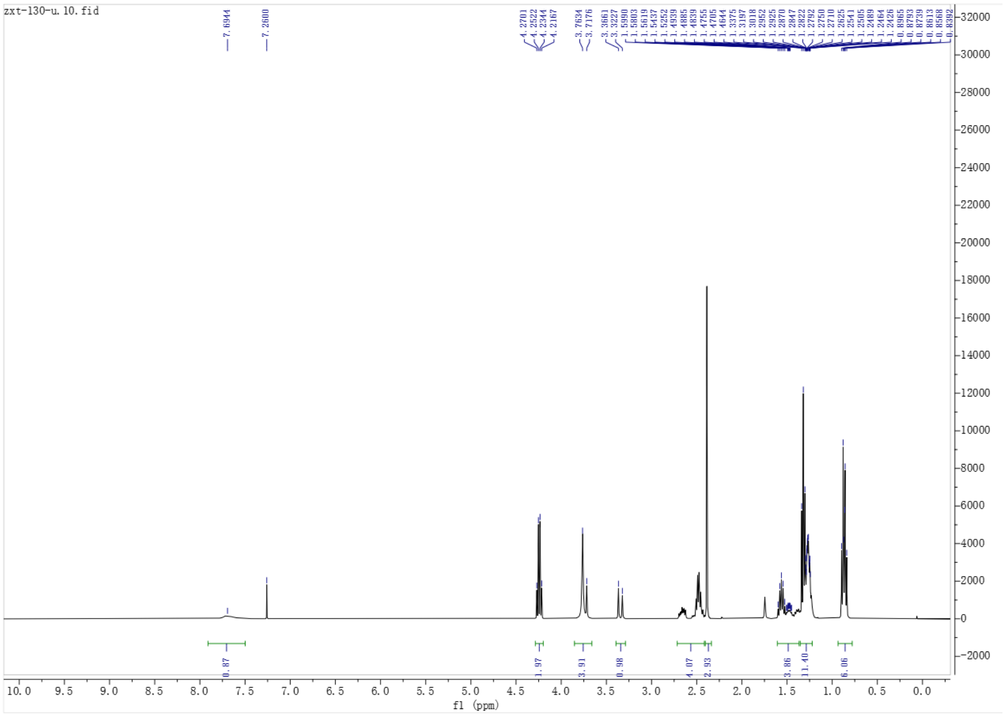

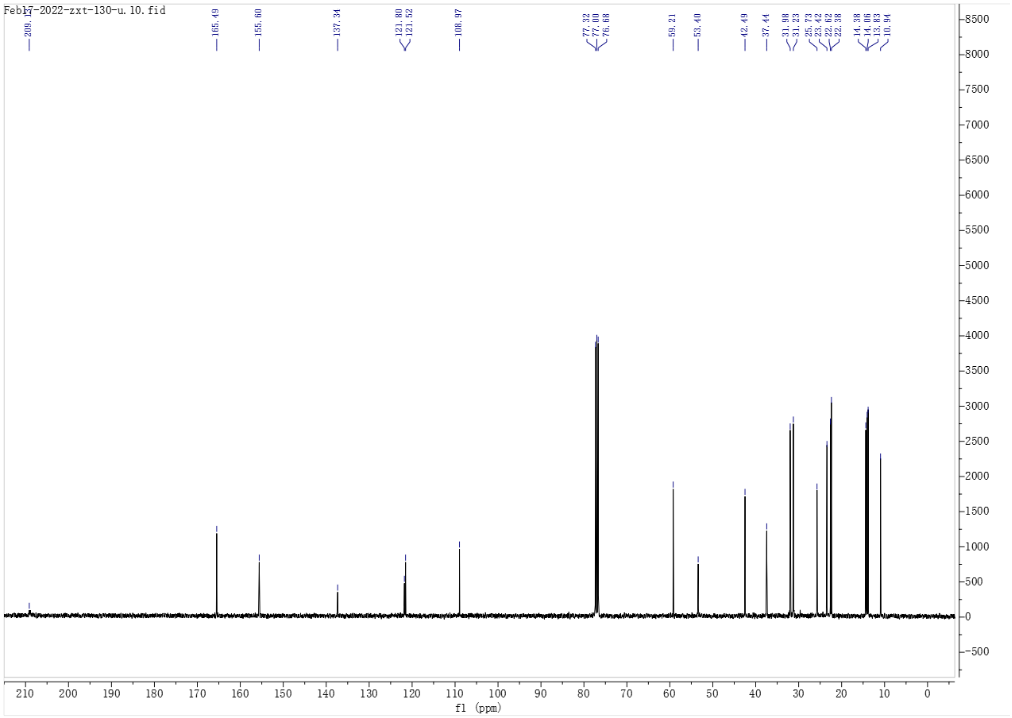

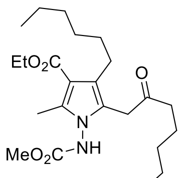
EtO2C
O
N
NH
MeO2C

3ag

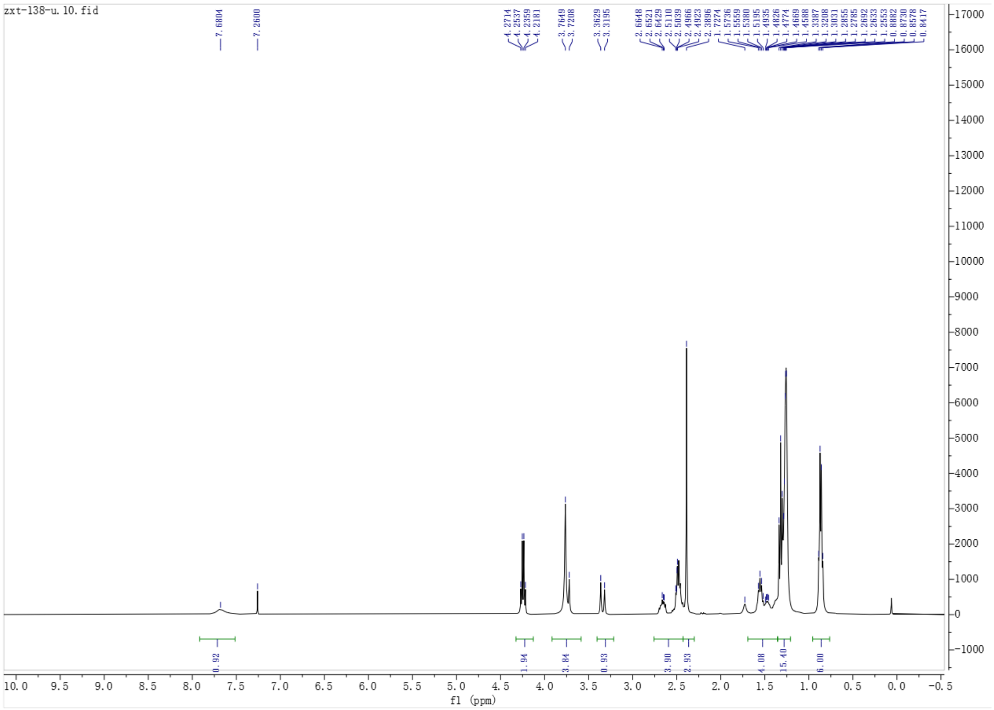
zxt-138-u.10.fid
7.6804
7.2600
4.2714
4.2537
4.2359
4.2181
3.7649
3.7208
3.3629
3.3195
2.6648
2.6521
2.6429
2.5110
2.5039
2.4966
2.4923
2.3896
1.7274
1.5736
1.5559
1.5380
1.5195
1.4935
1.4826
1.4774
1.4669
1.4588
1.3387
1.3208
1.3031
1.2855
1.2785
1.2692
1.2633
1.2553
0.8882
0.8730
0.8578
0.8417
0.92
1.94
3.84
0.93
3.90
2.93
4.08
15.40
6.00
f1 (ppm)

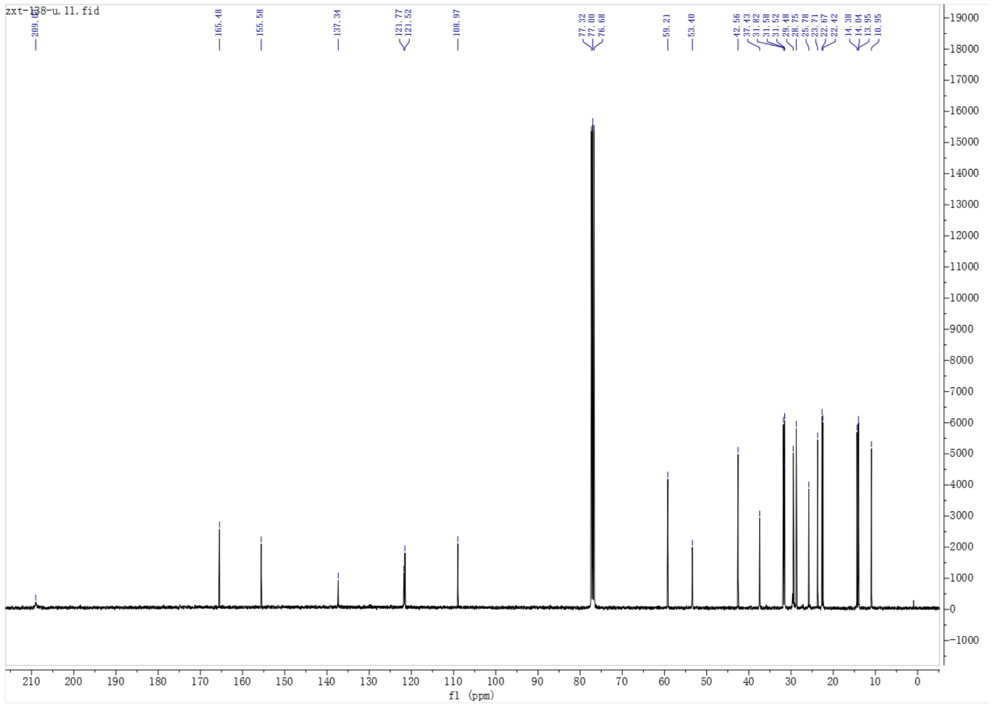
zxt-138-u.11.fid
165.48
155.58
137.34
121.77
121.52
108.97
77.32
77.00
76.68
59.21
53.40
42.56
37.43
31.82
31.52
29.48
28.75
25.78
23.71
22.67
22.42
14.38
14.04
13.95
10.95
f1 (ppm)

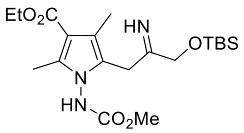

EtO2C
HN
OTBS
N
HN
CO2Me

3ah

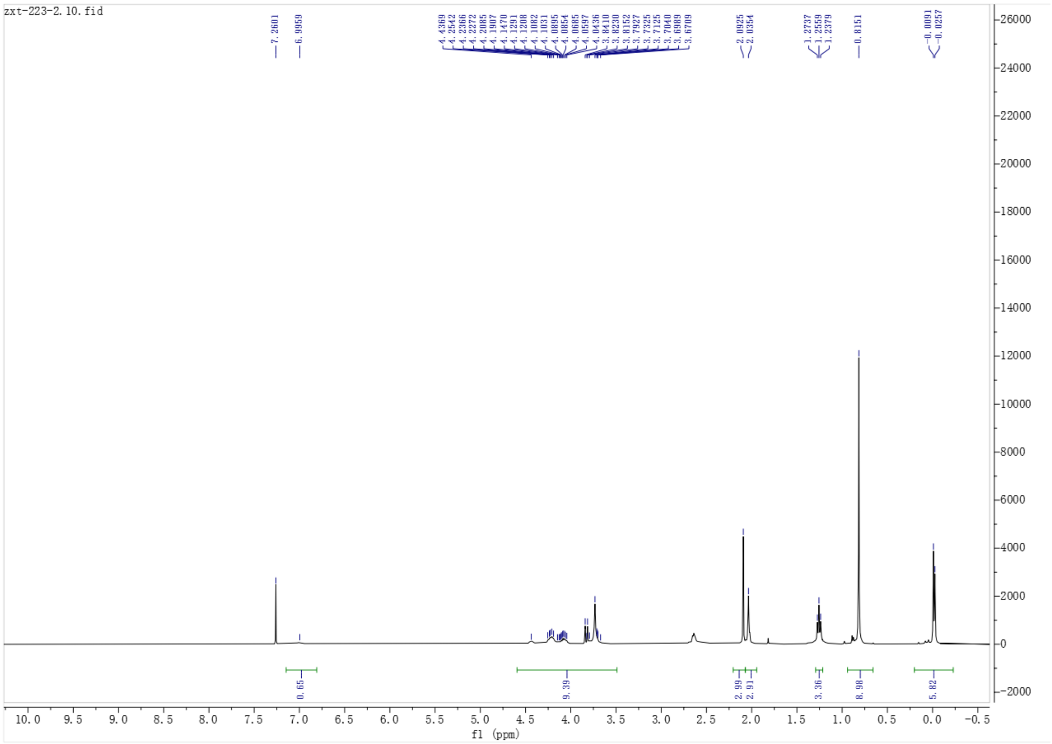

zxt-223-2.10.fid
7.2601
6.9959
4.4369
4.2542
4.2366
4.2272
4.2085
4.1907
4.1470
4.1291
4.1208
4.1082
4.1031
4.0895
4.0854
4.0685
4.0597
4.0436
3.8410
3.8230
3.8152
3.7927
3.7325
3.7125
3.7040
3.6989
3.6709
2.0925
2.0354
1.2737
1.2559
1.2379
0.8151
-0.0091
-0.0257
0.65
9.39
2.99
2.91
3.36
8.98
5.82
f1 (ppm)

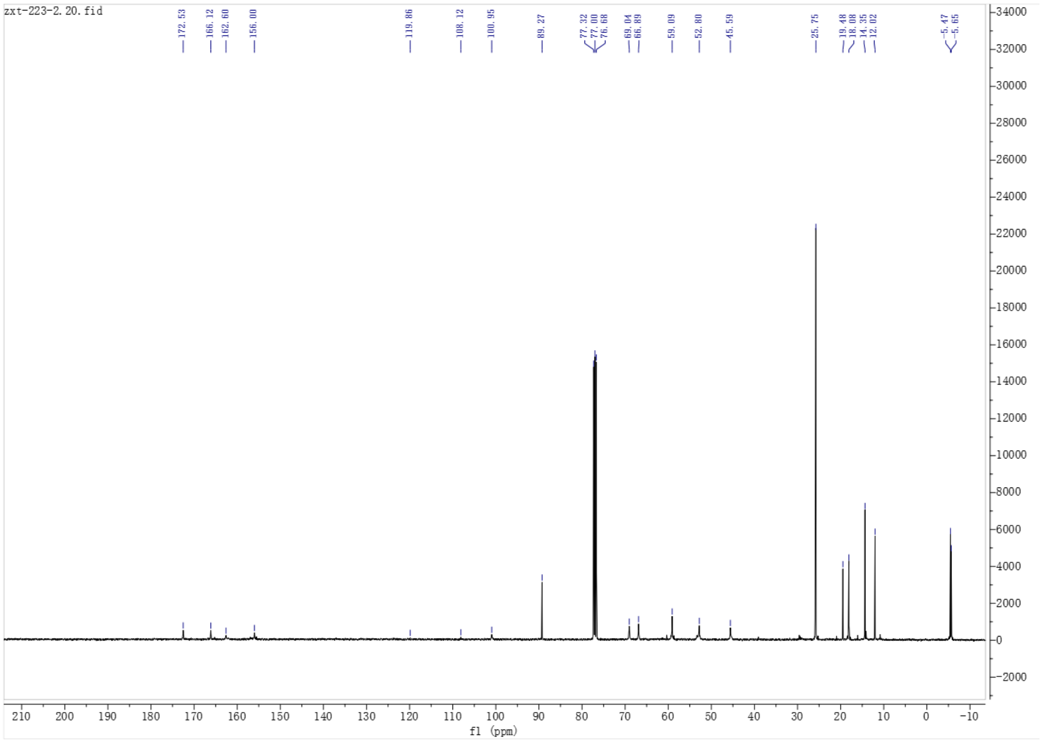

zxt-223-2.20.fid
172.53
166.12
162.60
156.00
119.86
108.12
100.95
89.27
77.32
77.00
76.68
69.04
66.89
59.09
52.80
45.59
25.75
19.48
18.08
14.35
12.02
-5.47
-5.65
f1 (ppm)

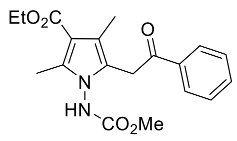

EtO2C
O
N
HN
CO2Me

3ai

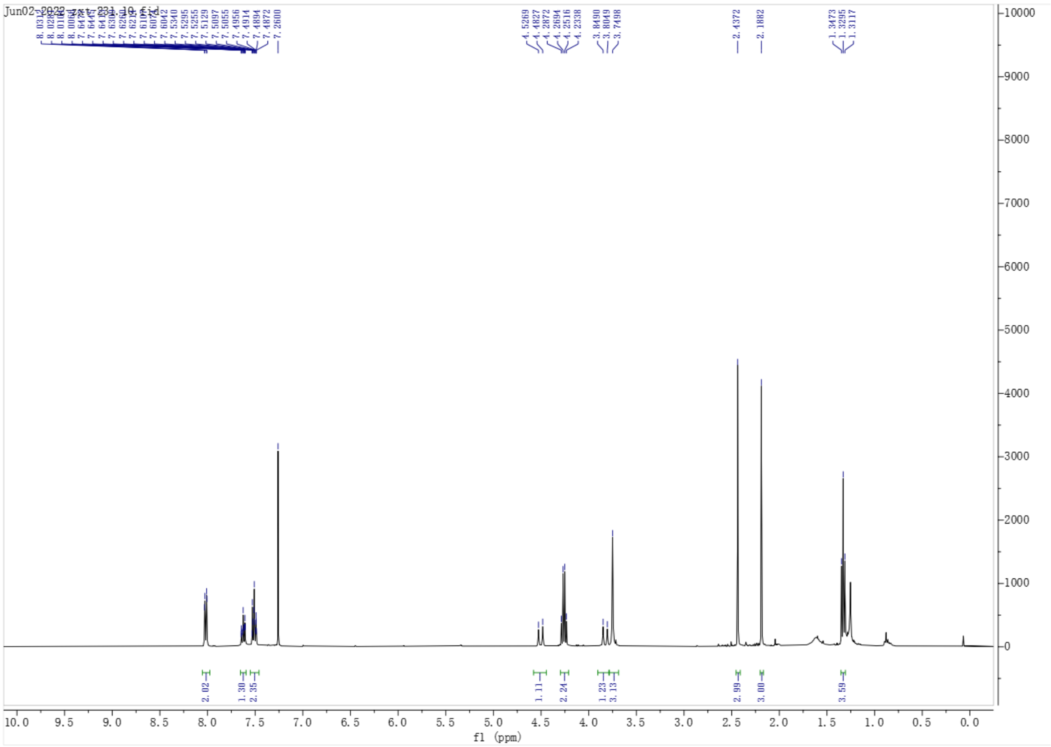

7.2600
4.5269
4.4827
4.2872
4.2694
4.2516
4.2338
3.8490
3.8049
3.7498
2.4372
2.1882
1.3473
1.3295
1.3117
2.02
1.30
2.35
1.11
2.24
1.23
3.13
2.99
3.00
3.59
f1 (ppm)

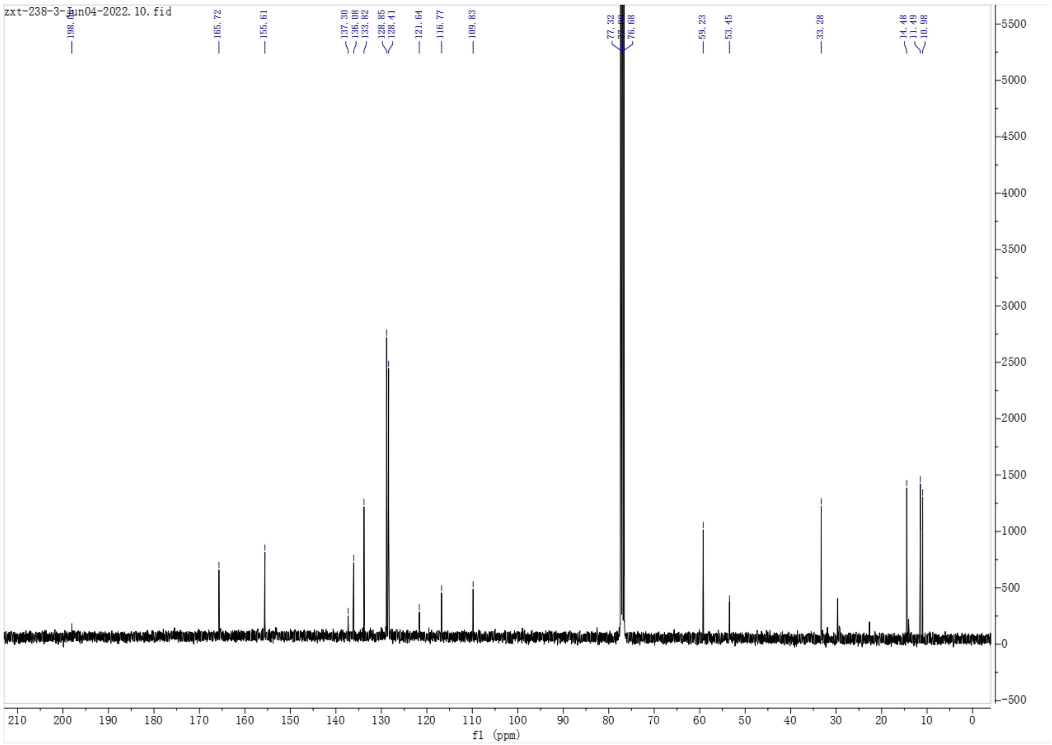

165.72
155.61
137.30
136.08
133.82
128.85
128.41
121.64
116.77
109.83
77.32
76.68
59.23
53.45
33.28
14.48
11.49
10.98
f1 (ppm)

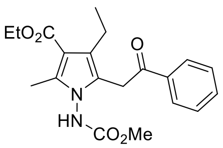
EtO2C
O
N
HN
CO2Me

3aj

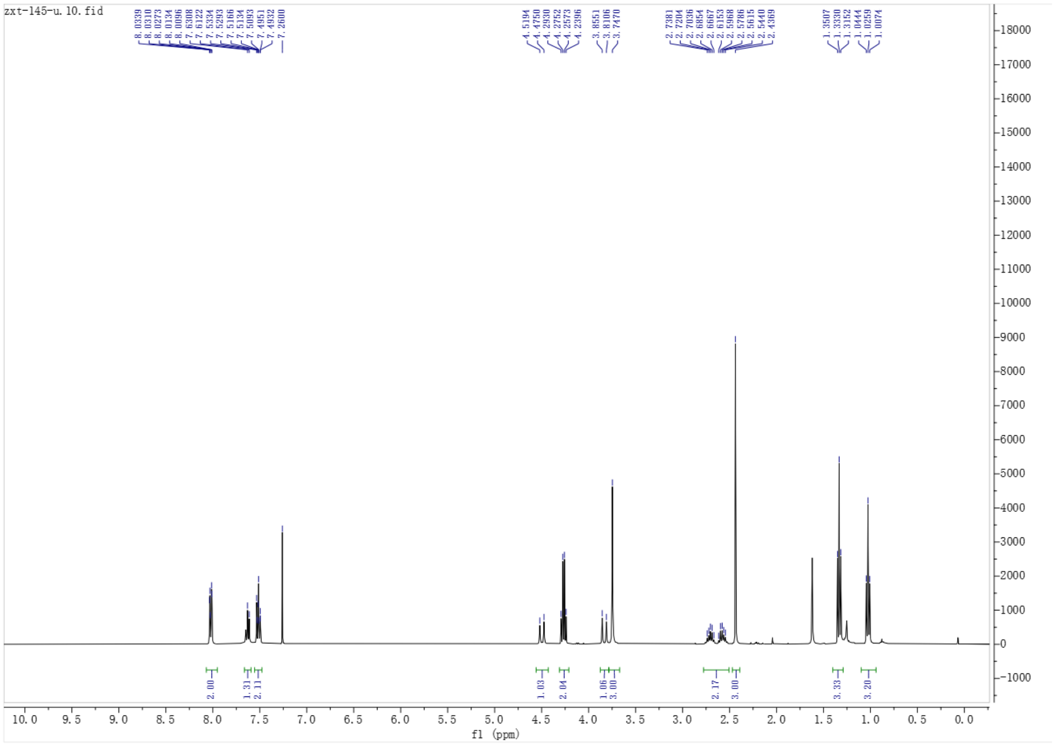
zxt-145-u.10.fid
8.0339
8.0310
8.0273
8.0134
8.0096
7.6308
7.6122
7.5334
7.5293
7.5166
7.5134
7.5093
7.4951
7.4932
7.2600
4.5194
4.4750
4.2930
4.2752
4.2573
4.2396
3.8551
3.8106
3.7470
2.7381
2.7204
2.7036
2.6854
2.6667
2.6153
2.5968
2.5786
2.5615
2.5440
2.4369
1.3507
1.3330
1.3152
1.0444
1.0259
1.0074
2.00
1.31
2.11
1.03
2.04
1.06
3.00
2.17
3.00
3.33
3.20
f1 (ppm)

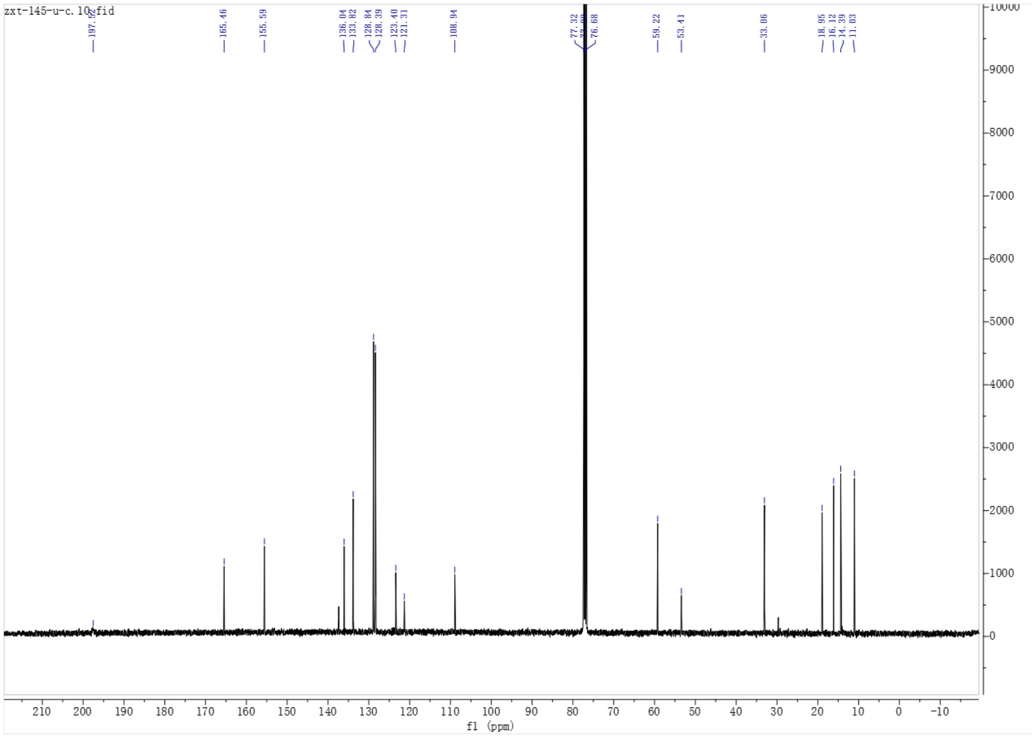
zxt-145-u-c.10.fid
165.46
155.59
136.04
133.82
128.84
128.39
123.40
121.31
108.94
77.32
76.68
59.22
53.41
33.06
18.95
16.12
14.39
11.03
f1 (ppm)

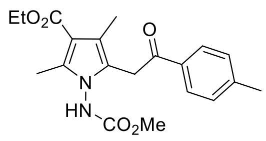
EtO2C
O
N
HN
CO2Me

3ak

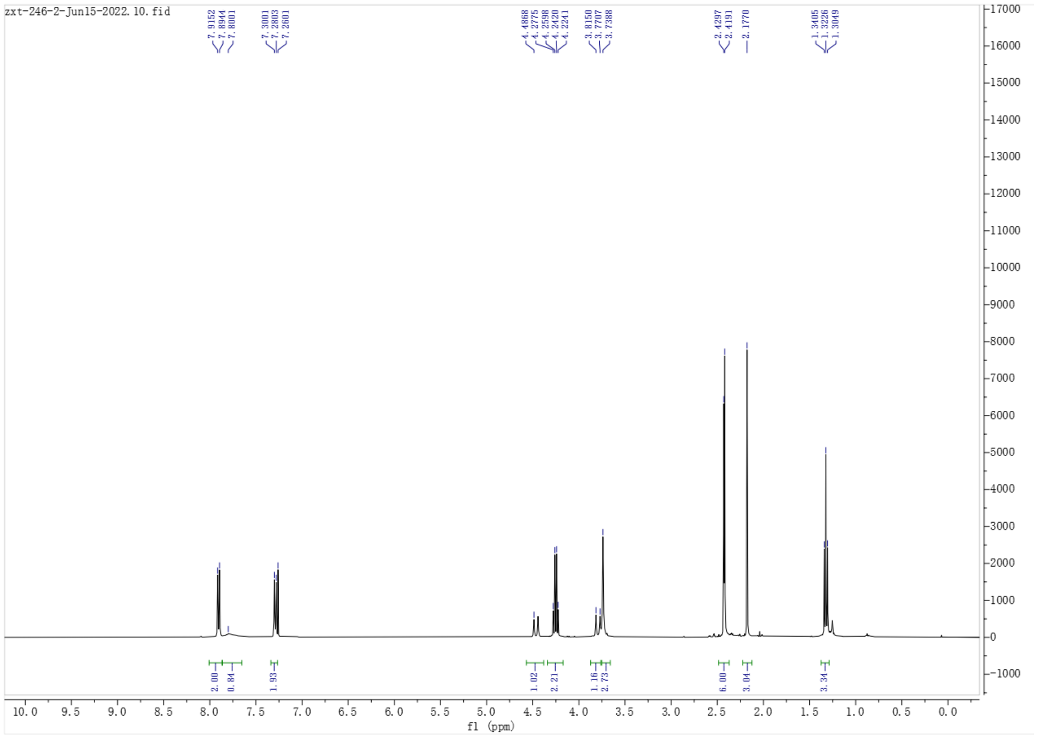
zxt-246-2-Jun15-2022.10.fid
7.9152
7.8944
7.8001
7.3001
7.2803
7.2601
4.4868
4.2775
4.2598
4.2420
4.2241
3.8150
3.7707
3.7388
2.4297
2.4191
2.1770
1.3405
1.3226
1.3049
2.00
0.84
1.93
1.02
2.21
1.16
2.73
6.00
3.04
3.34
f1 (ppm)

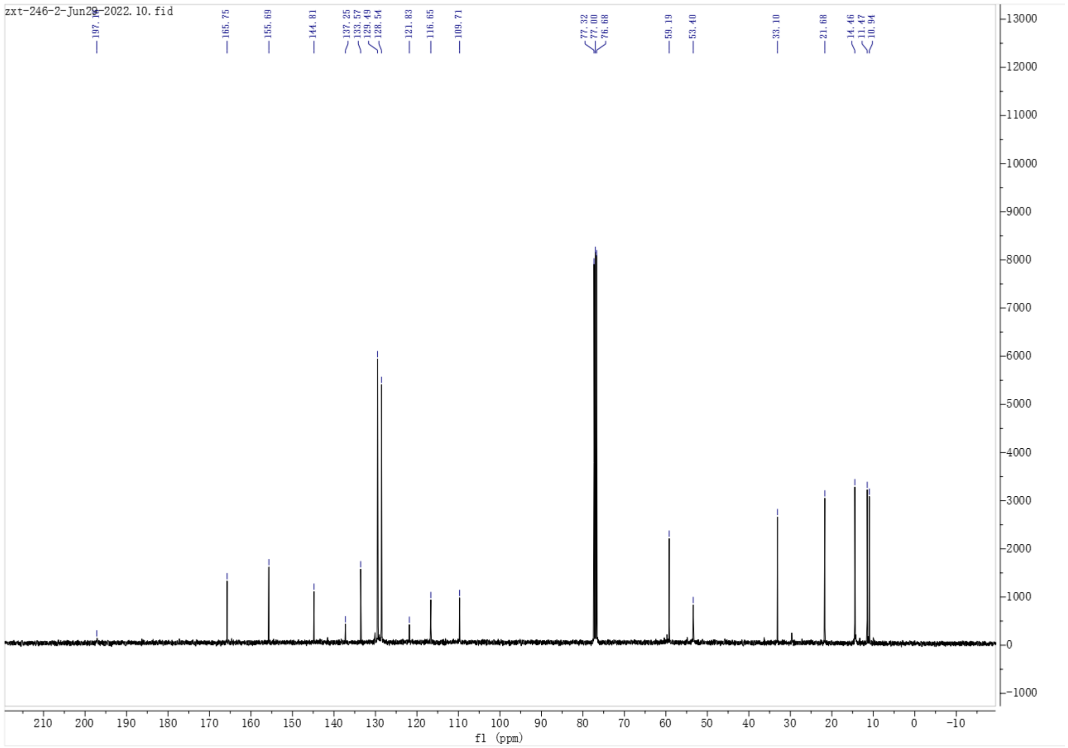
165.75
155.69
144.81
137.25
133.57
129.49
128.54
121.83
116.65
109.71
77.32
77.00
76.68
59.19
53.40
33.10
21.68
14.46
11.47
10.94
f1 (ppm)

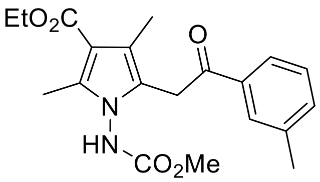
EtO2C
O
N
HN
CO2Me

3al

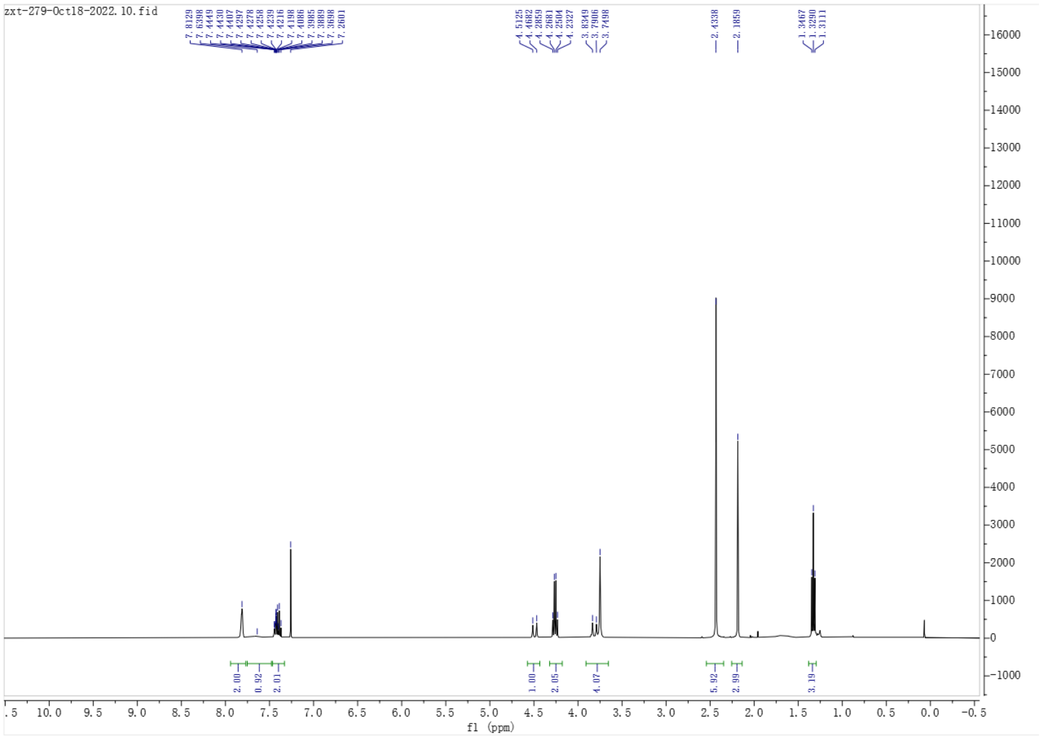
zxt-279-Oct18-2022.10.fid
f1 (ppm)

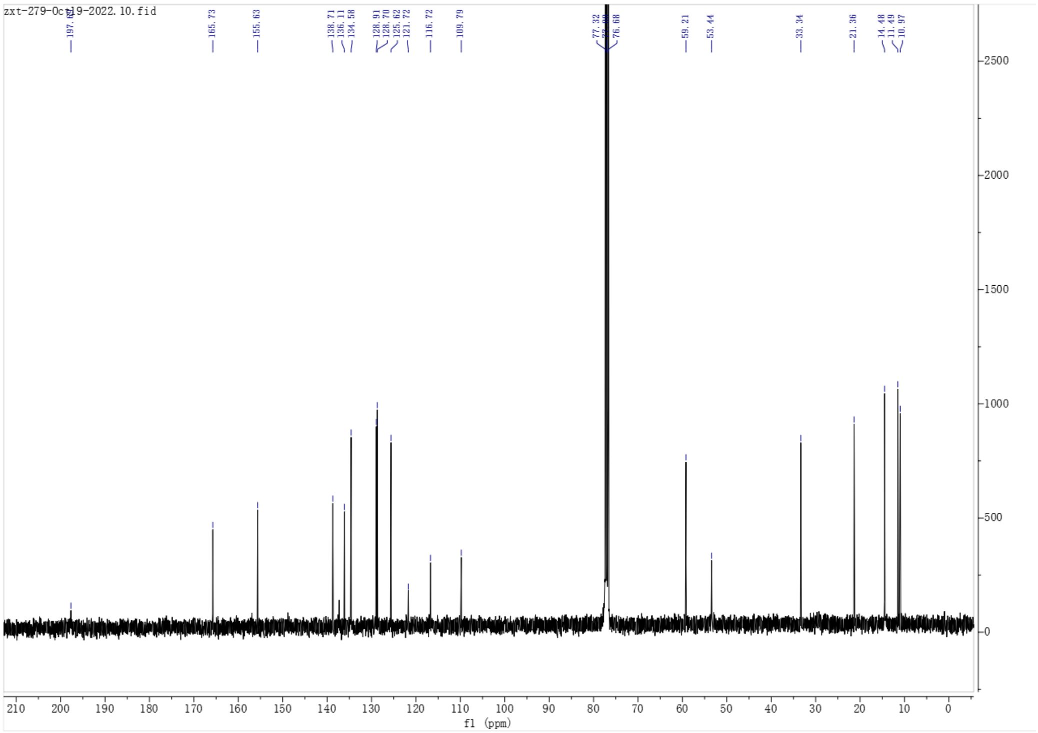
zxt-279-Oct19-2022.10.fid
f1 (ppm)

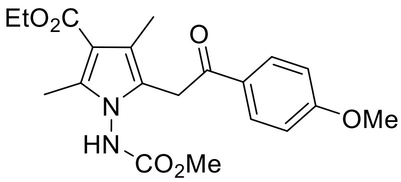
EtO2C
O
N
HN
CO2Me
OMe

3am

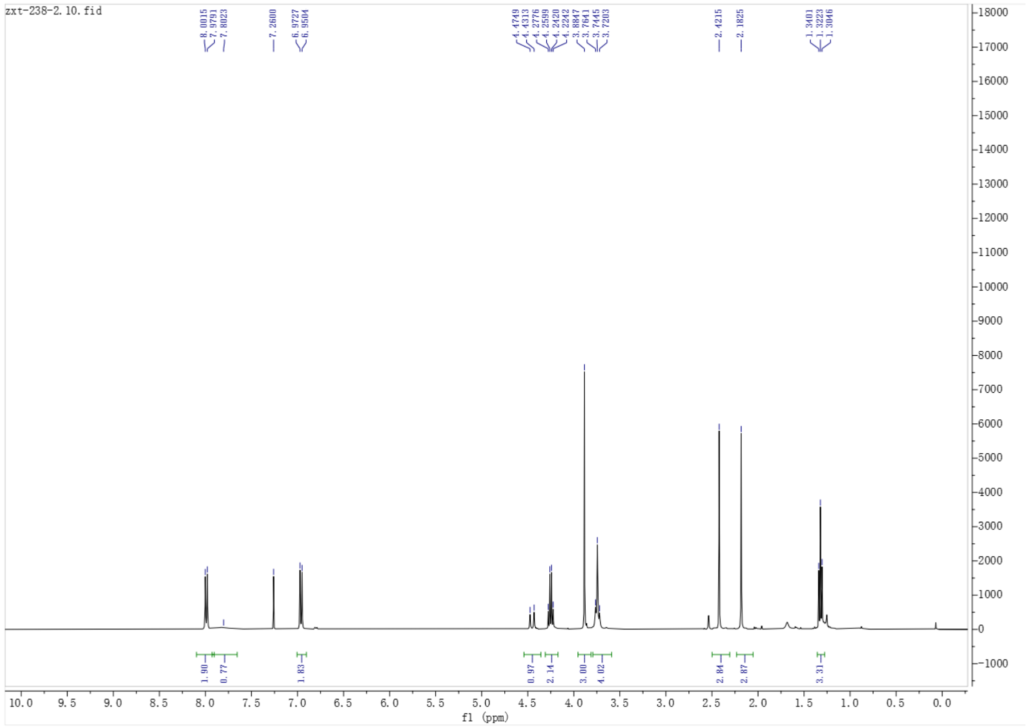
zxt-238-2.10.fid
f1 (ppm)

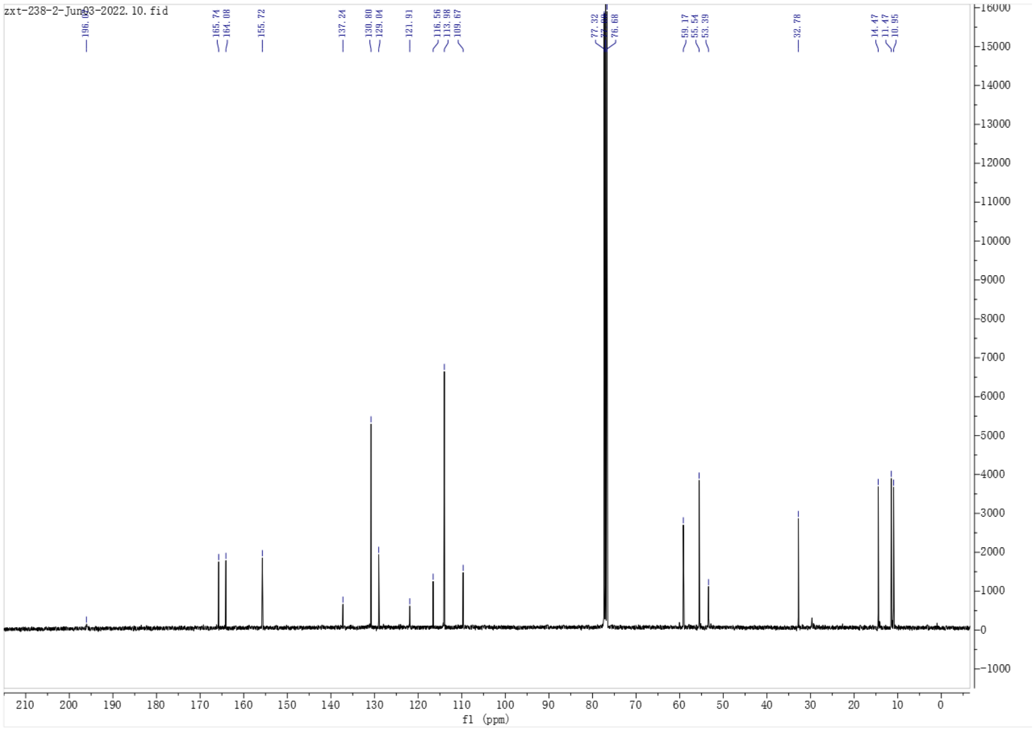
zxt-238-2-Jun03-2022.10.fid
f1 (ppm)

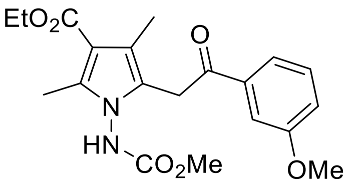
EtO2C
O
N
HN
CO2Me
OMe

3an

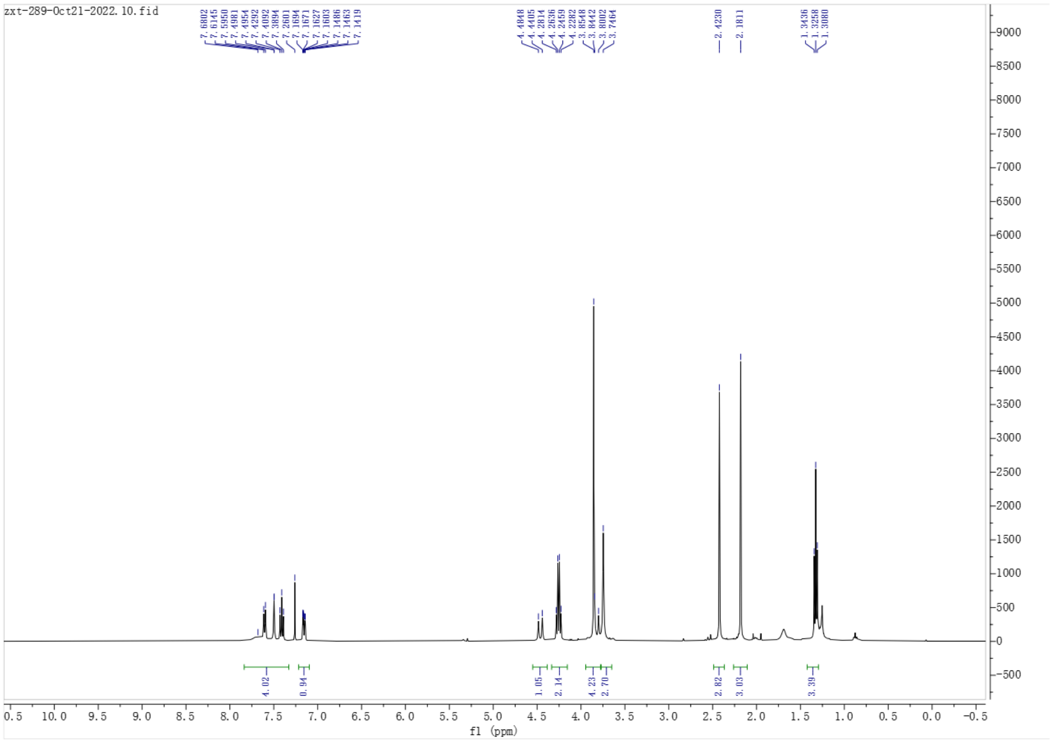
zxt-289-Oct21-2022.10.fid
f1 (ppm)

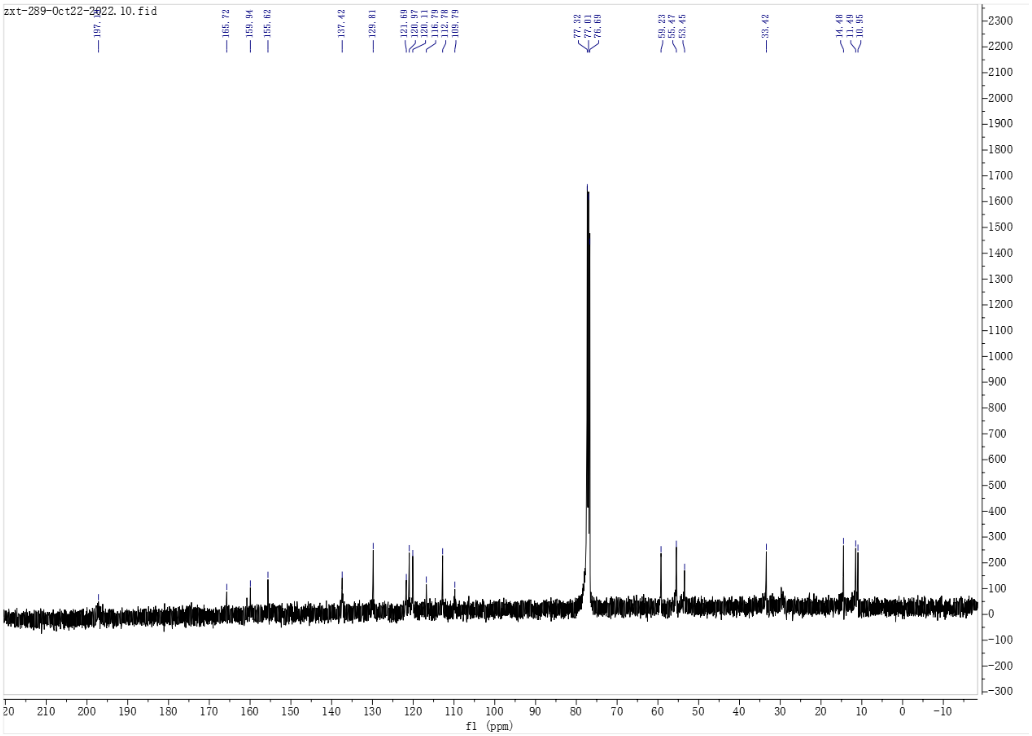
zxt-289-Oct22-2022.10.fid
f1 (ppm)

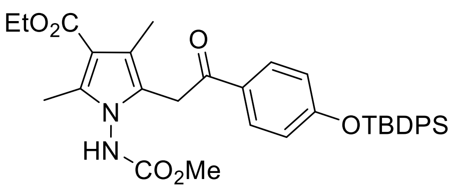

3ao

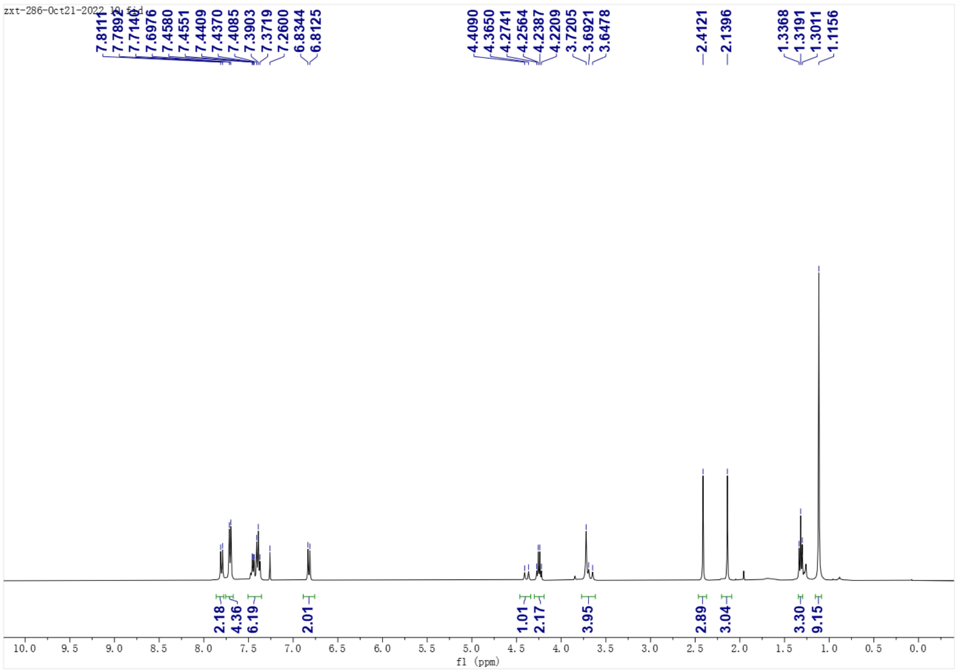

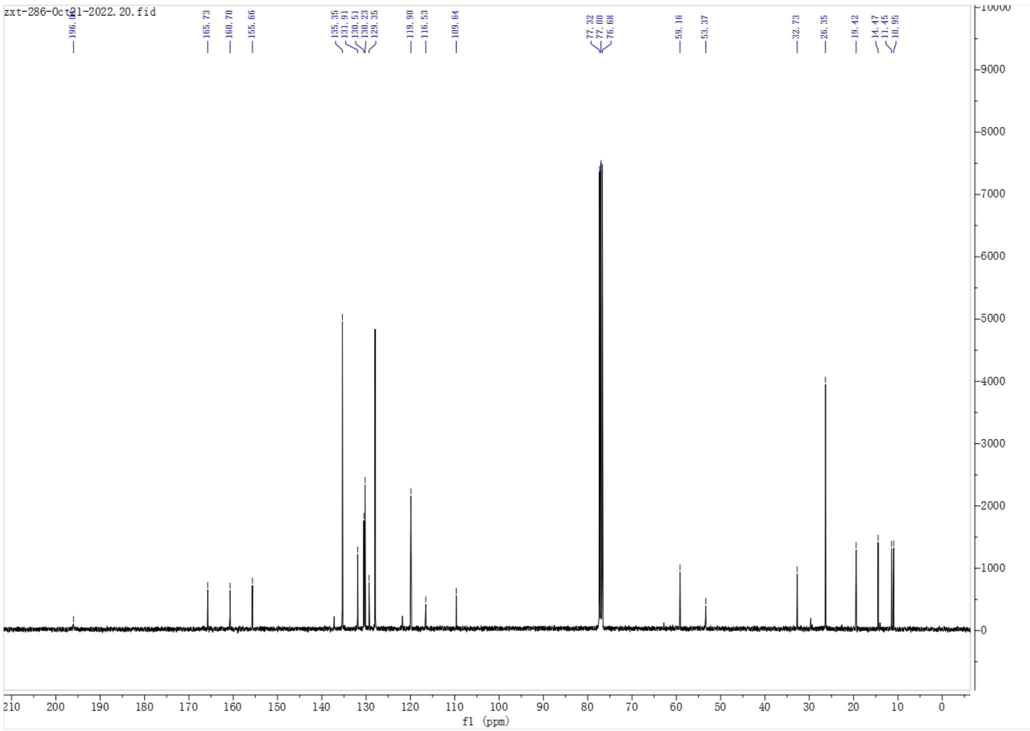

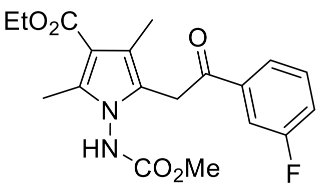

3ap

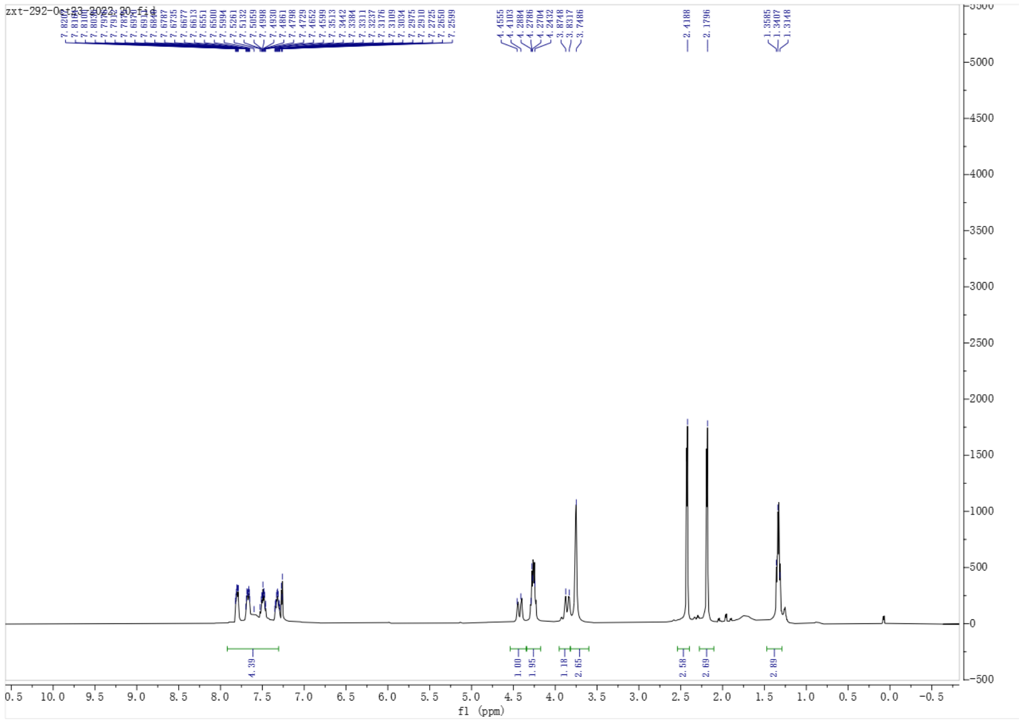

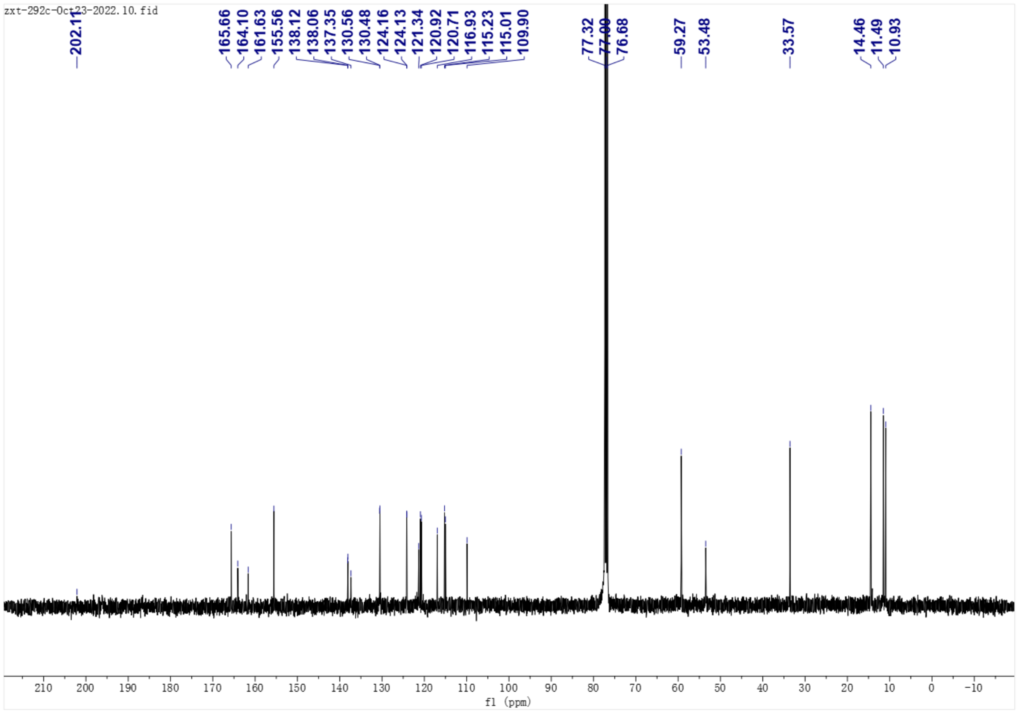

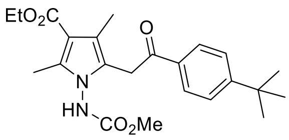
EtO2C
O
N
HN
CO2Me

3aq

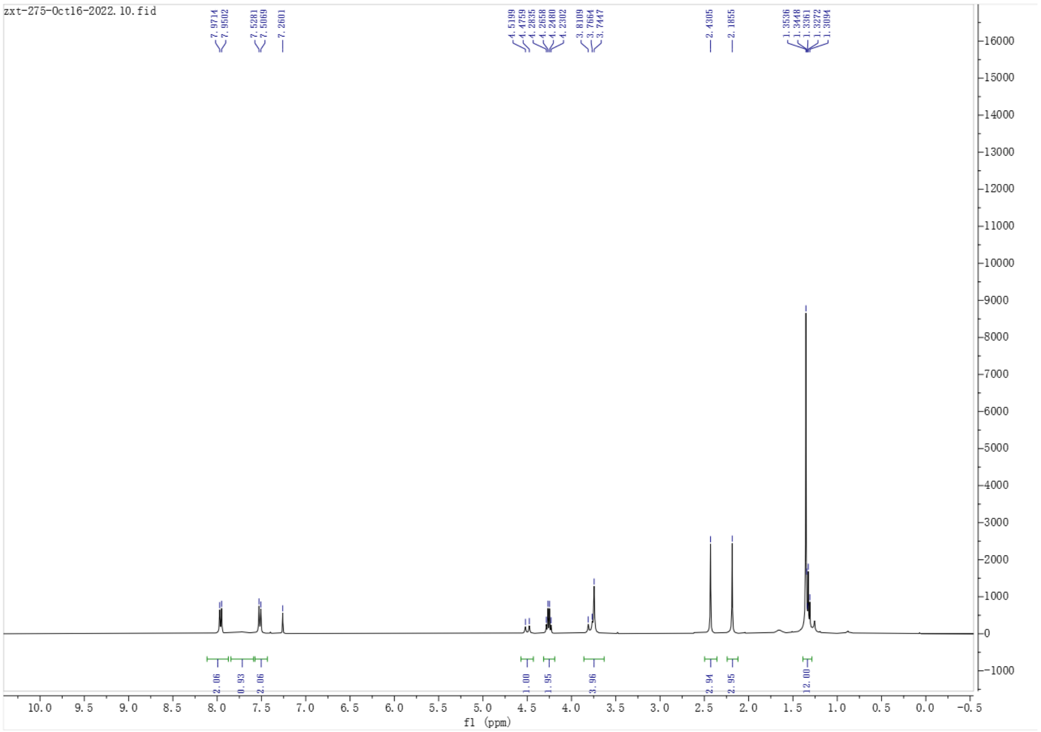
zxt-275-Oct16-2022.10.fid
7.9714
7.9502
7.5281
7.5069
7.2601
4.5199
4.4759
4.2835
4.2658
4.2480
4.2302
3.8109
3.7664
3.7447
2.4305
2.1855
1.3536
1.3448
1.3361
1.3272
1.3094
2.06
0.93
2.06
1.00
1.95
3.96
2.94
2.95
12.00
f1 (ppm)

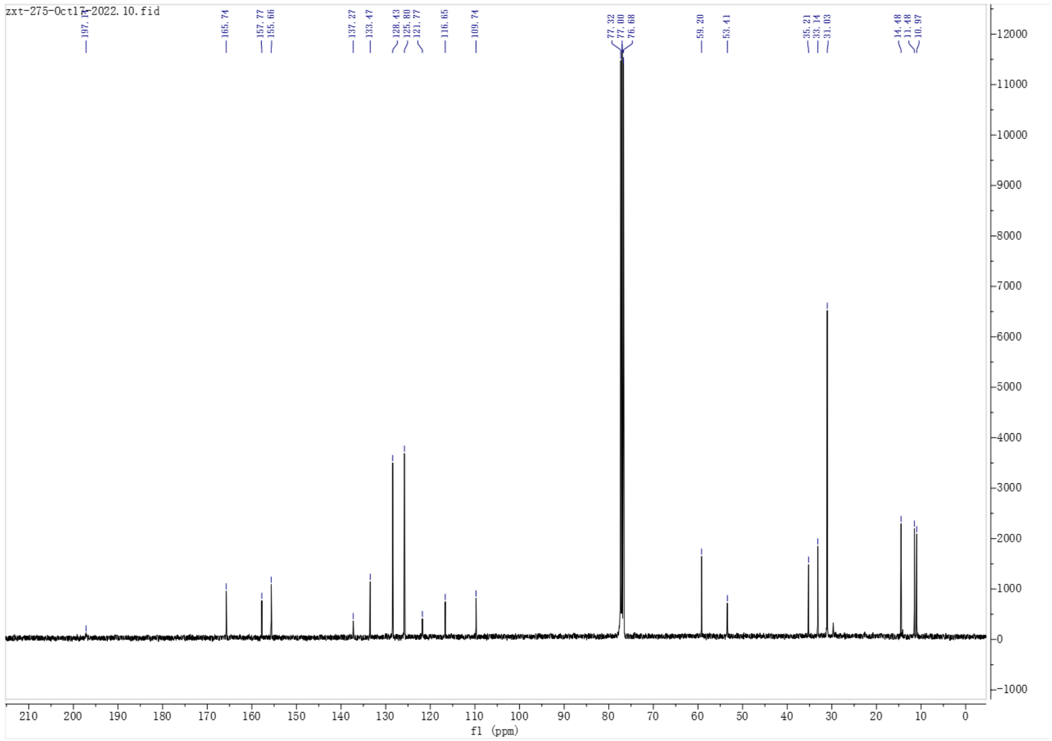
zxt-275-Oct17-2022.10.fid
165.74
157.77
155.66
137.27
133.47
128.43
125.80
121.77
116.65
109.74
77.32
77.00
76.68
59.20
53.41
35.21
33.14
31.03
14.48
11.48
10.97
f1 (ppm)

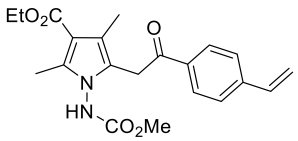
EtO2C
O
N
HN
CO2Me

3ar

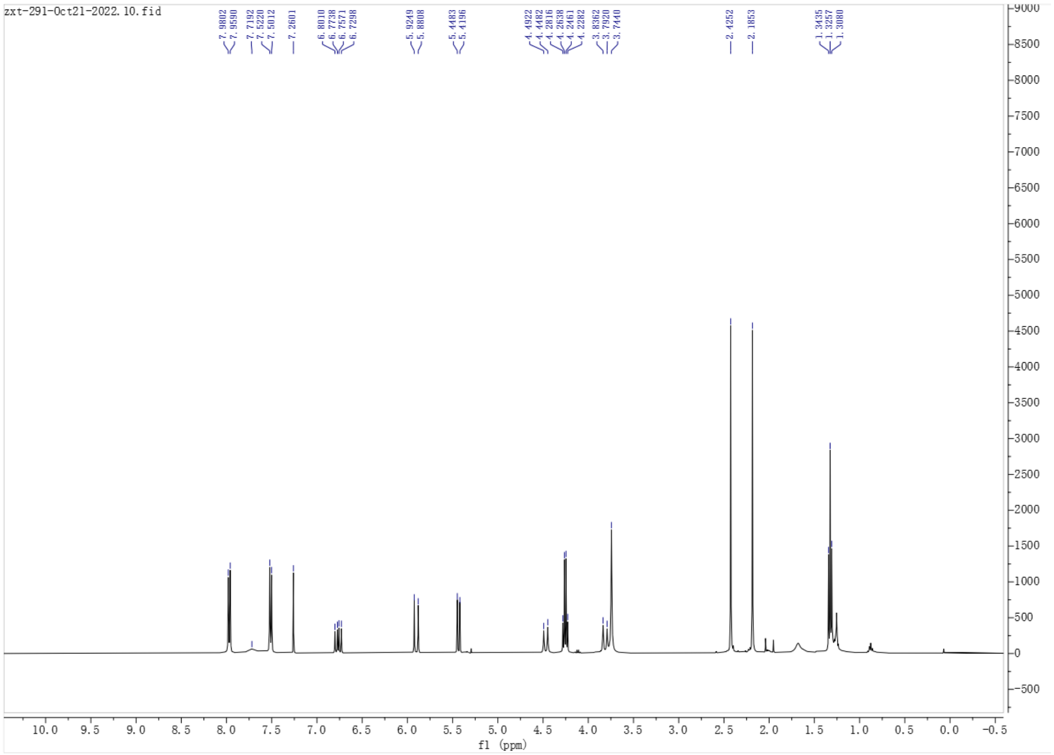
zxt-291-Oct21-2022.10.fid
7.9882
7.9590
7.7192
7.5220
7.5012
7.2601
6.8010
6.7738
6.7571
6.7298
5.9249
5.8808
5.4483
5.4196
4.4922
4.4482
4.2816
4.2638
4.2461
4.2282
3.8362
3.7920
3.7440
2.4252
2.1853
1.3435
1.3257
1.3080
f1 (ppm)

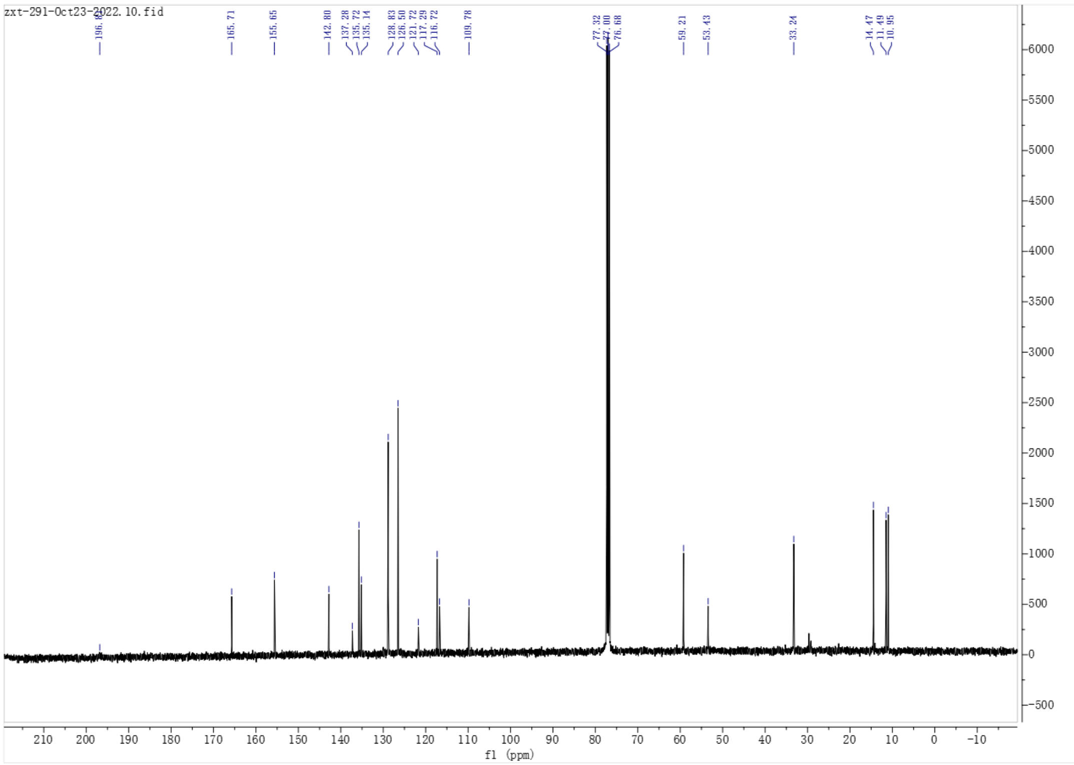
zxt-291-Oct23-2022.10.fid
[illegible]
165.71
155.65
142.80
137.28
135.72
135.14
128.83
126.50
121.72
117.29
116.72
109.78
77.32
77.00
76.68
59.21
53.43
33.24
14.47
11.49
10.95
f1 (ppm)

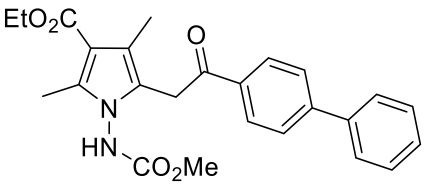

3as

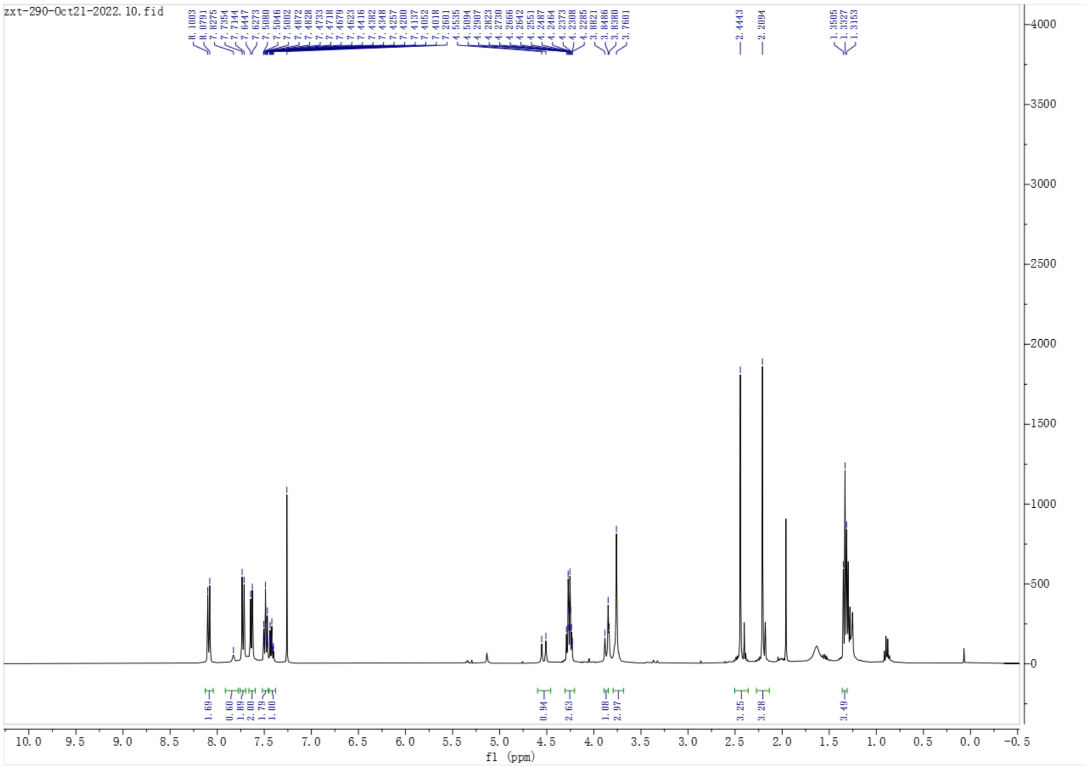

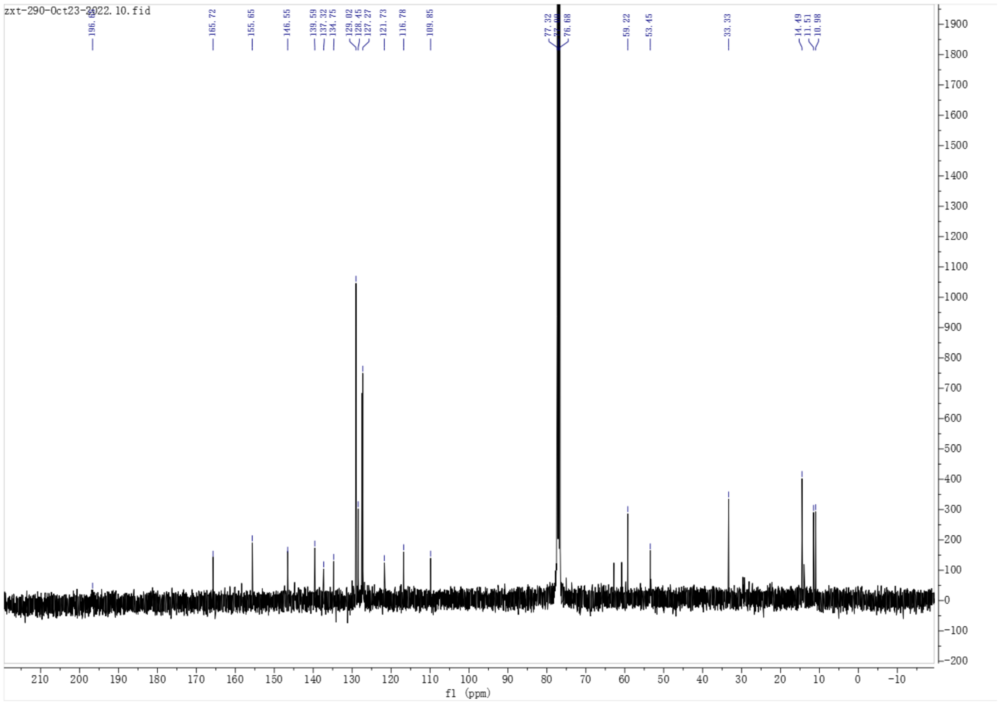

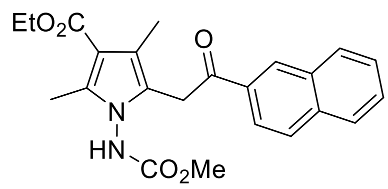
EtO2C
O
N
HN
CO2Me

3at

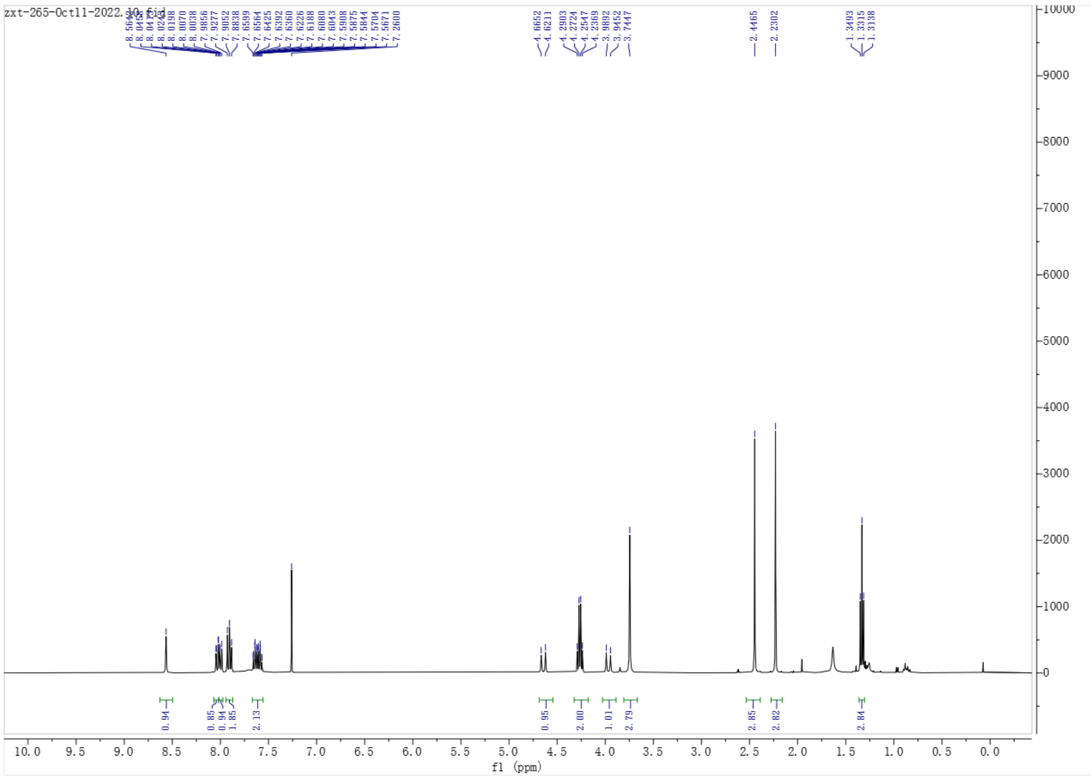
zxt-265-Oct11-2022.10.fid
4.6652
4.6211
4.2903
4.2724
4.2547
4.2369
3.9892
3.9452
3.7447
2.4465
2.2302
1.3493
1.3315
1.3138
0.94
0.85
0.94
1.85
2.13
0.95
2.00
1.01
2.79
2.85
2.82
2.84
f1 (ppm)

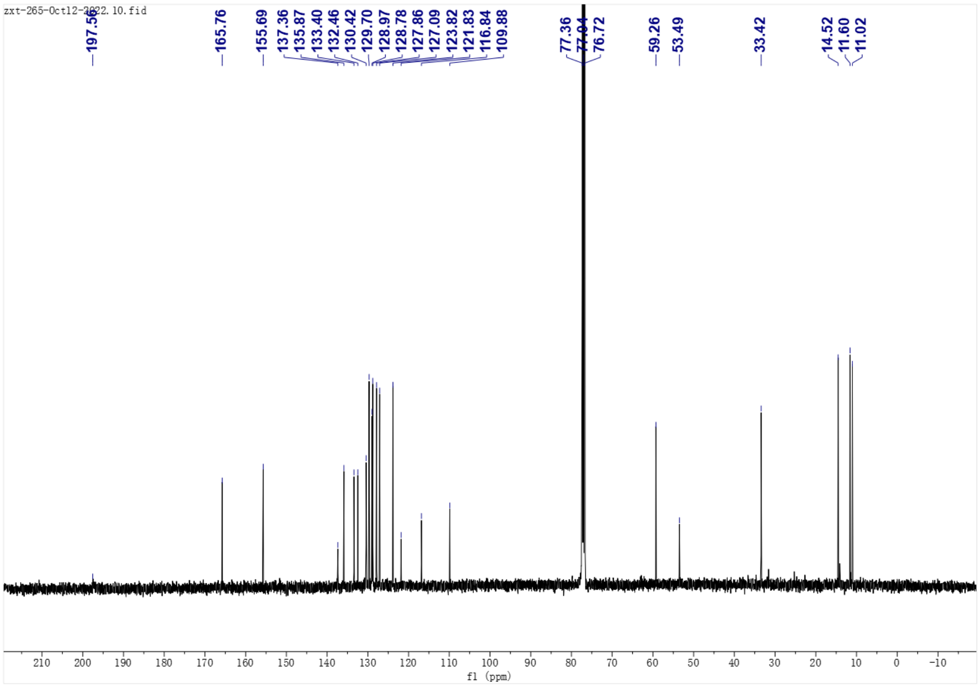
zxt-265-Oct12-2022.10.fid
197.56
165.76
155.69
137.36
135.87
133.40
132.46
130.42
129.70
128.97
128.78
127.86
127.09
123.82
121.83
116.84
109.88
77.36
77.04
76.72
59.26
53.49
33.42
14.52
11.60
11.02
f1 (ppm)

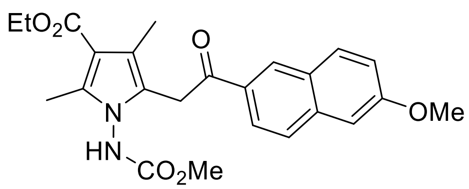

3au

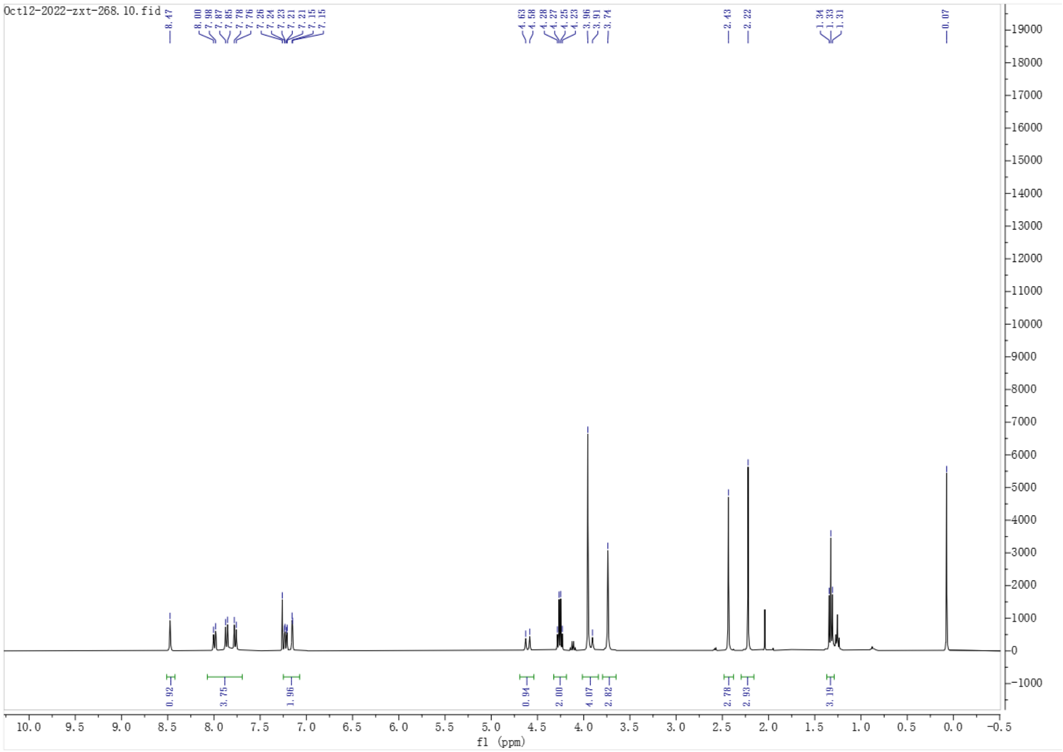

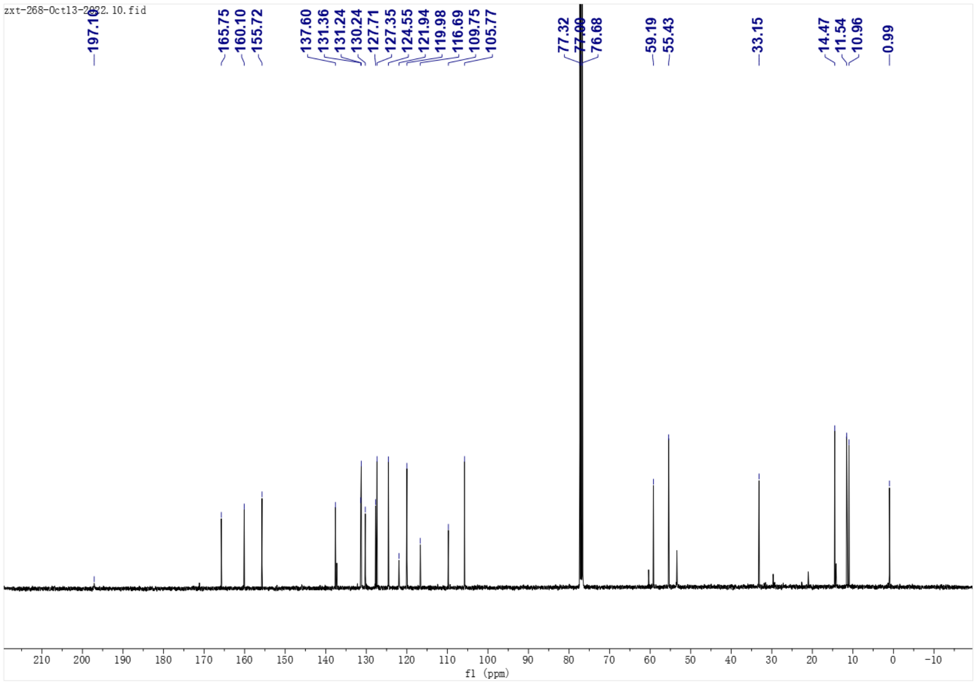

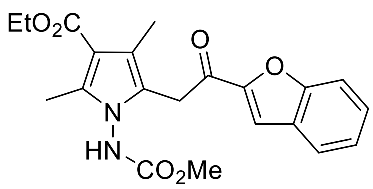
EtO2C
O
O
N
HN
CO2Me

3av

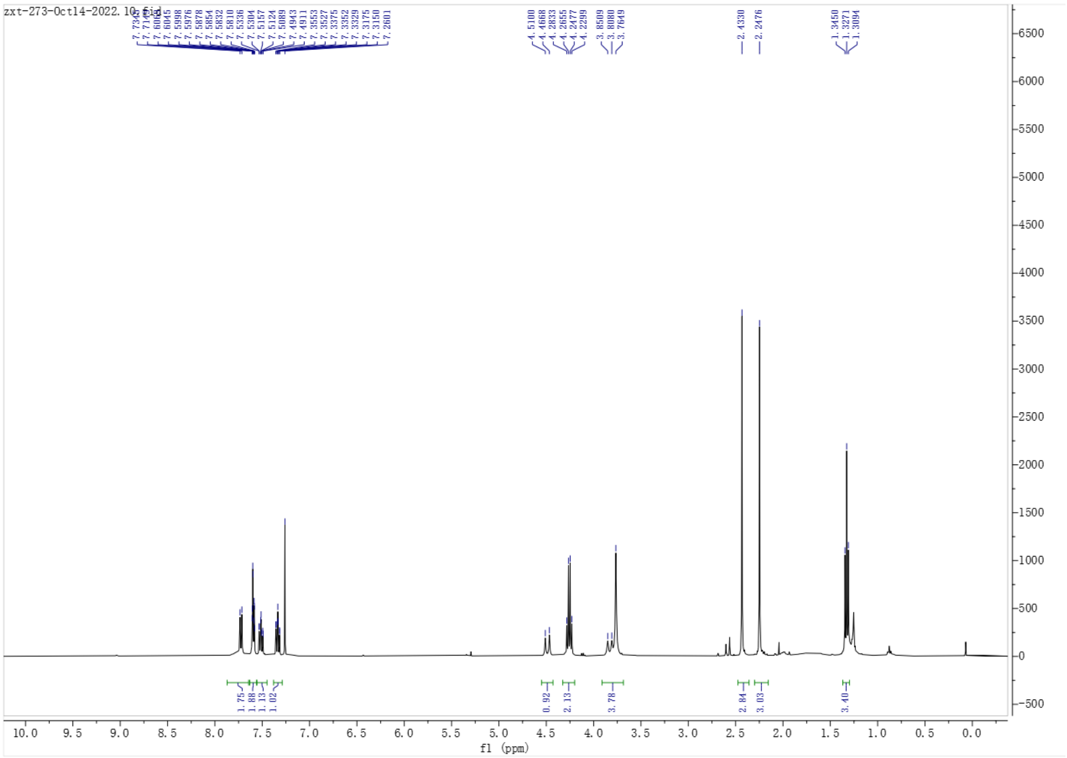
zxt-273-Oct14-2022.10.fid
f1 (ppm)

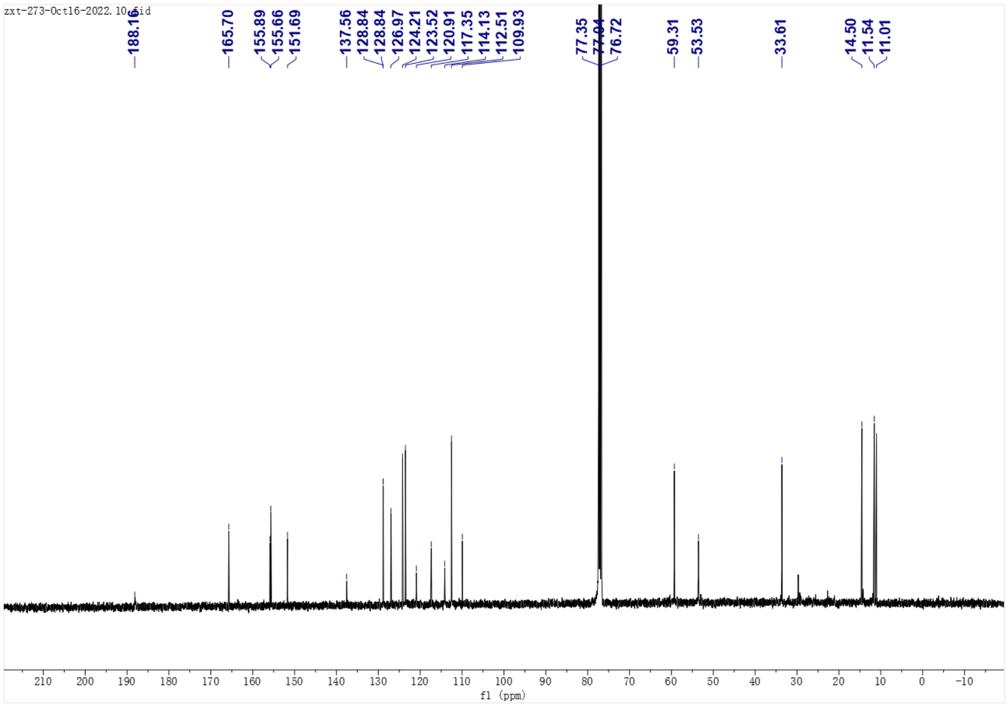
zxt-273-Oct16-2022.10.fid
188.16
165.70
155.89
155.66
151.69
137.56
128.84
128.84
126.97
124.21
123.52
120.91
117.35
114.13
112.51
109.93
77.35
77.04
76.72
59.31
53.53
33.61
14.50
11.54
11.01
f1 (ppm)

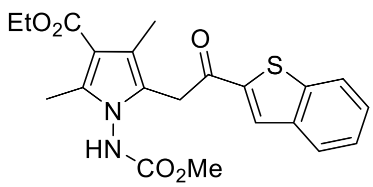

3aw

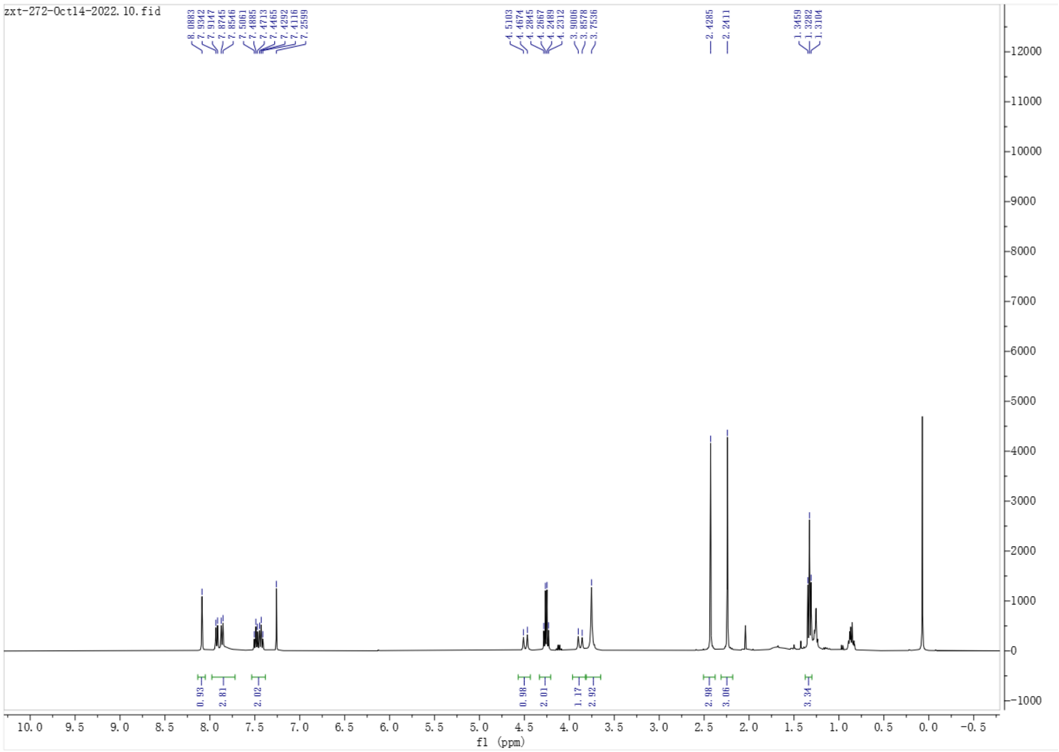

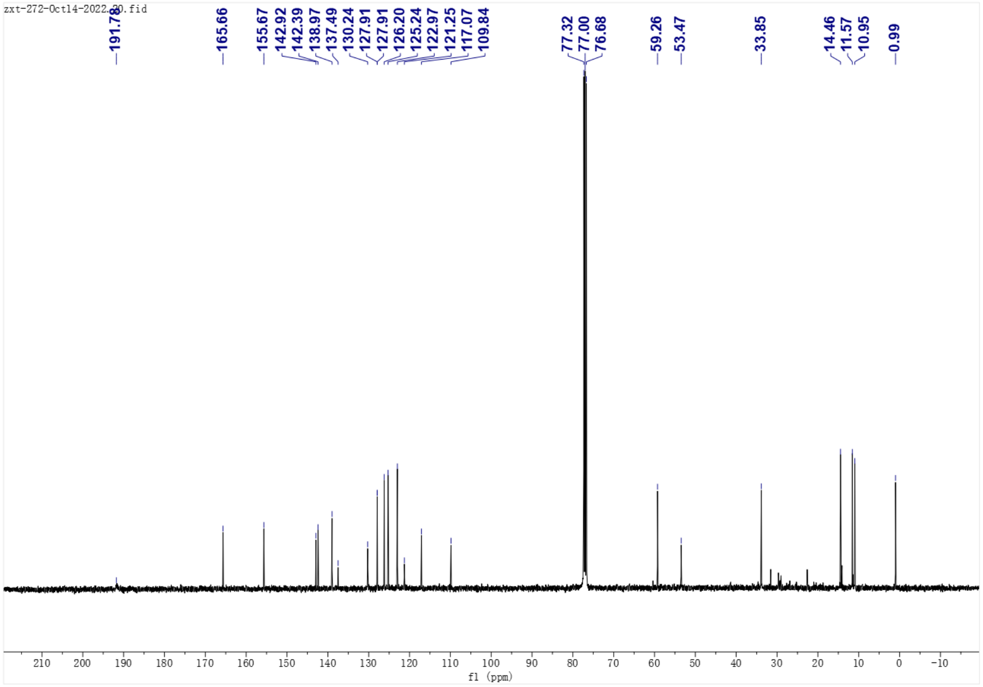

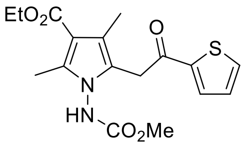

3ax

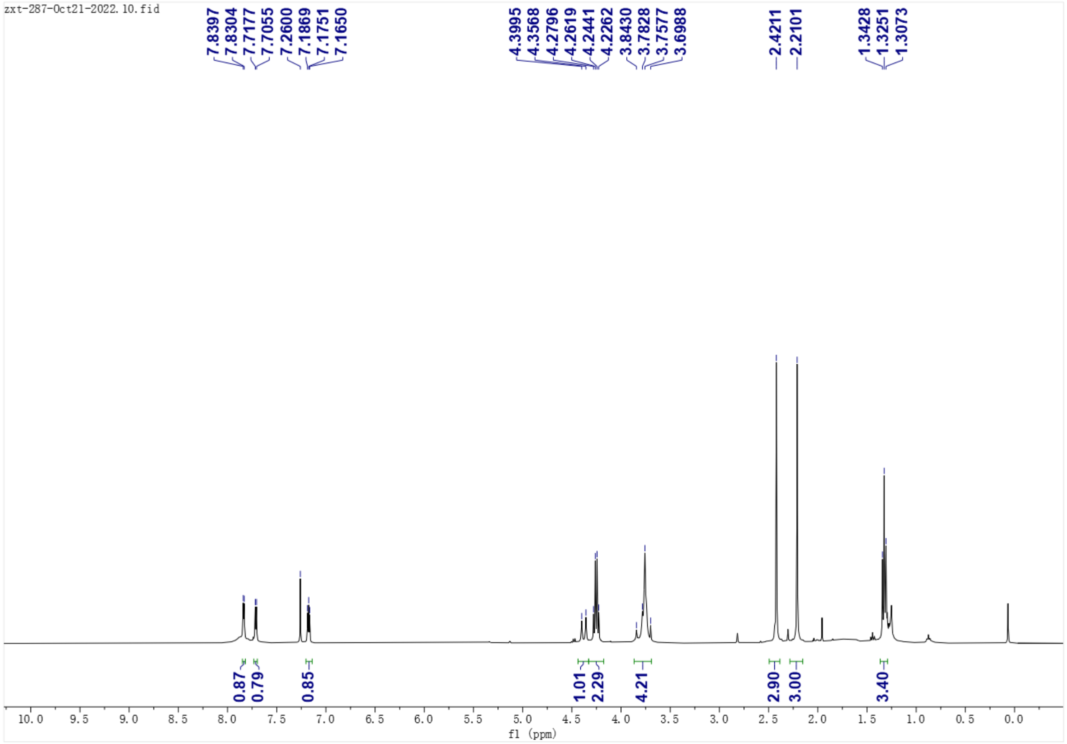
zxt-287-Oct21-2022.10.fid
7.8397
7.8304
7.7177
7.7055
7.2600
7.1869
7.1751
7.1650
4.3995
4.3568
4.2796
4.2619
4.2441
4.2262
3.8430
3.7828
3.7577
3.6988
2.4211
2.2101
1.3428
1.3251
1.3073
0.87
0.79
0.85
1.01
2.29
4.21
2.90
3.00
3.40
f1 (ppm)

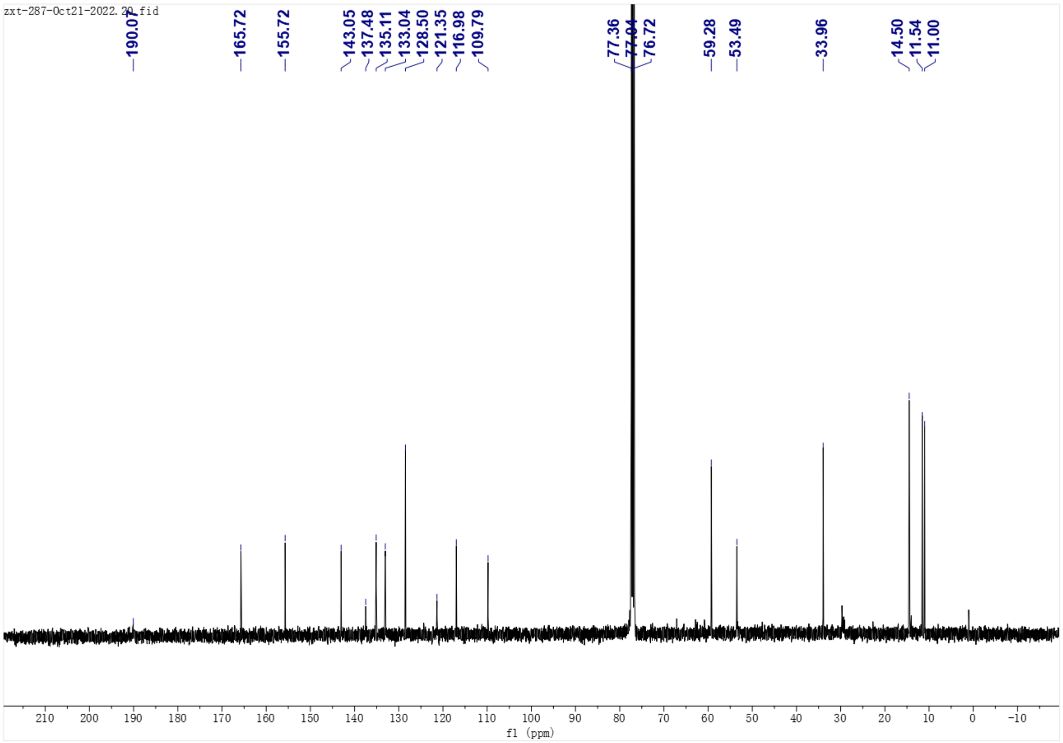
190.07
165.72
155.72
143.05
137.48
135.11
133.04
128.50
121.35
116.98
109.79
77.36
77.04
76.72
59.28
53.49
33.96
14.50
11.54
11.00
f1 (ppm)

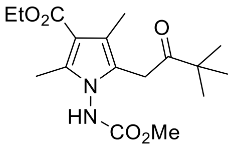
EtO2C
O
N
HN
CO2Me

3az

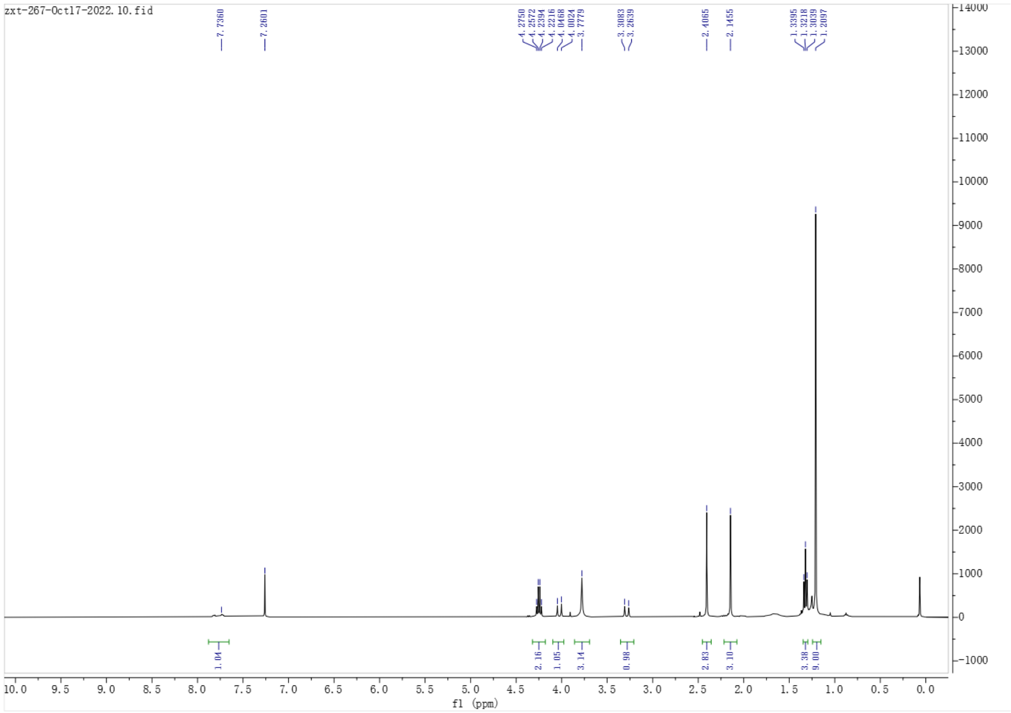
zxt-267-Oct17-2022.10.fid
7.7360
7.2601
4.2750
4.2572
4.2394
4.2216
4.0468
4.0024
3.7779
3.3083
3.2639
2.4065
2.1455
1.3395
1.3218
1.3039
1.2097
1.04
2.16
1.05
3.14
0.98
2.83
3.10
3.38
9.00
f1 (ppm)

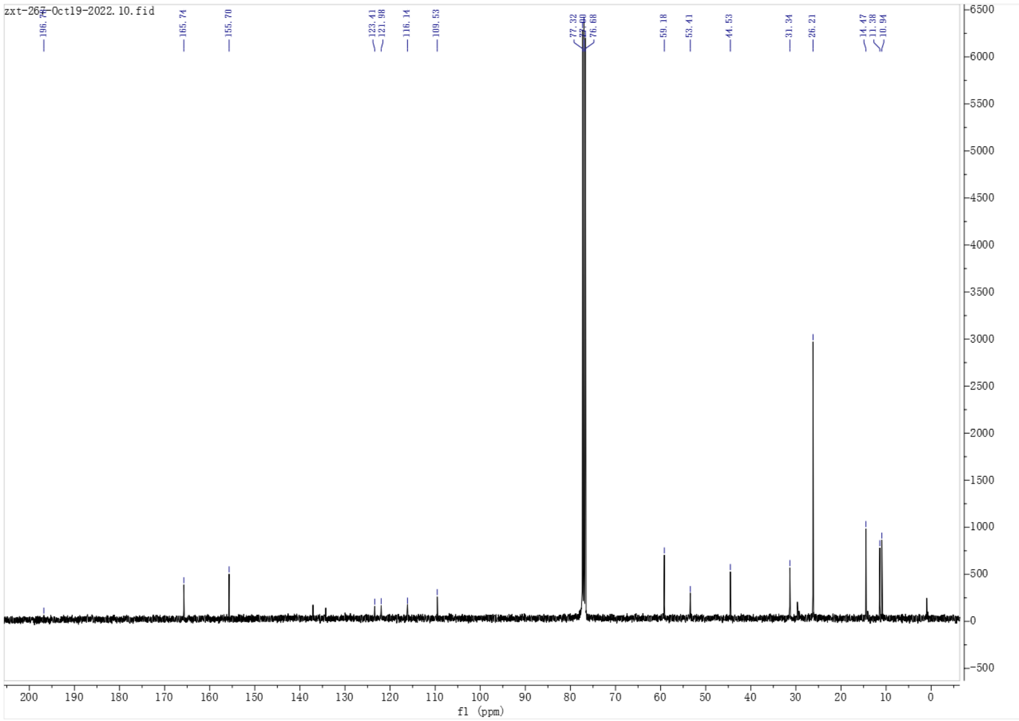
165.74
155.70
123.41
121.98
116.14
109.53
77.32
76.68
59.18
53.41
44.53
31.34
26.21
14.47
11.38
10.94
f1 (ppm)

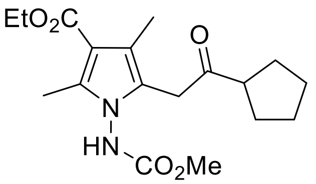
EtO2C
O
N
HN
CO2Me

3az'

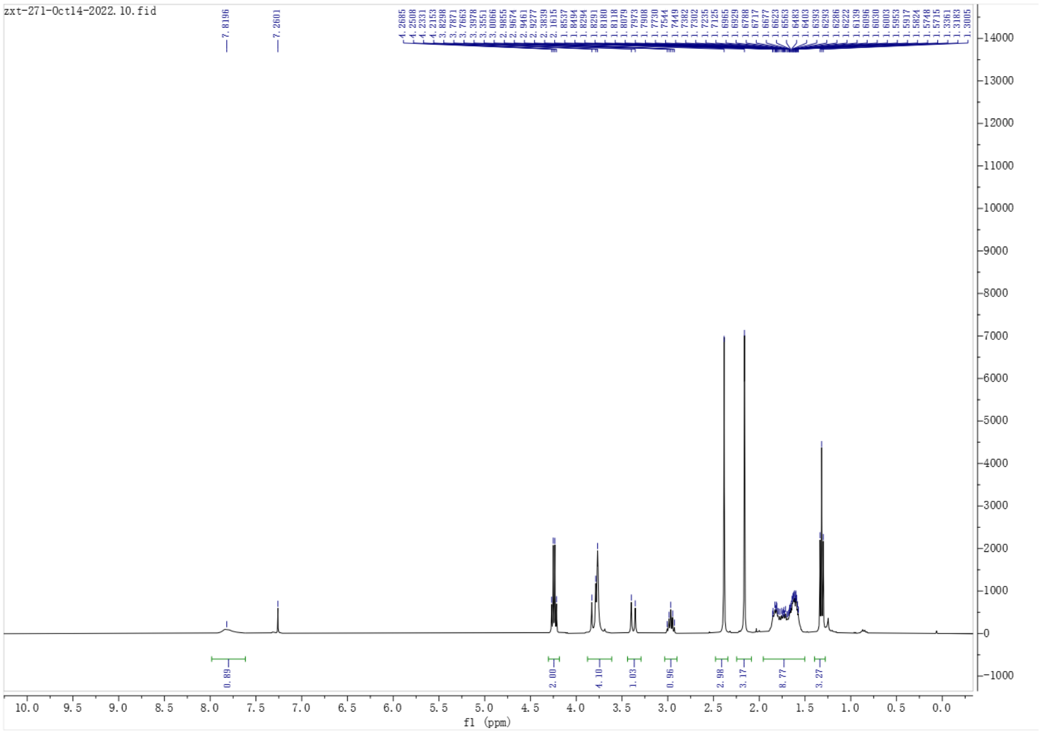
zxt-271-Oct14-2022.10.fid
f1 (ppm)

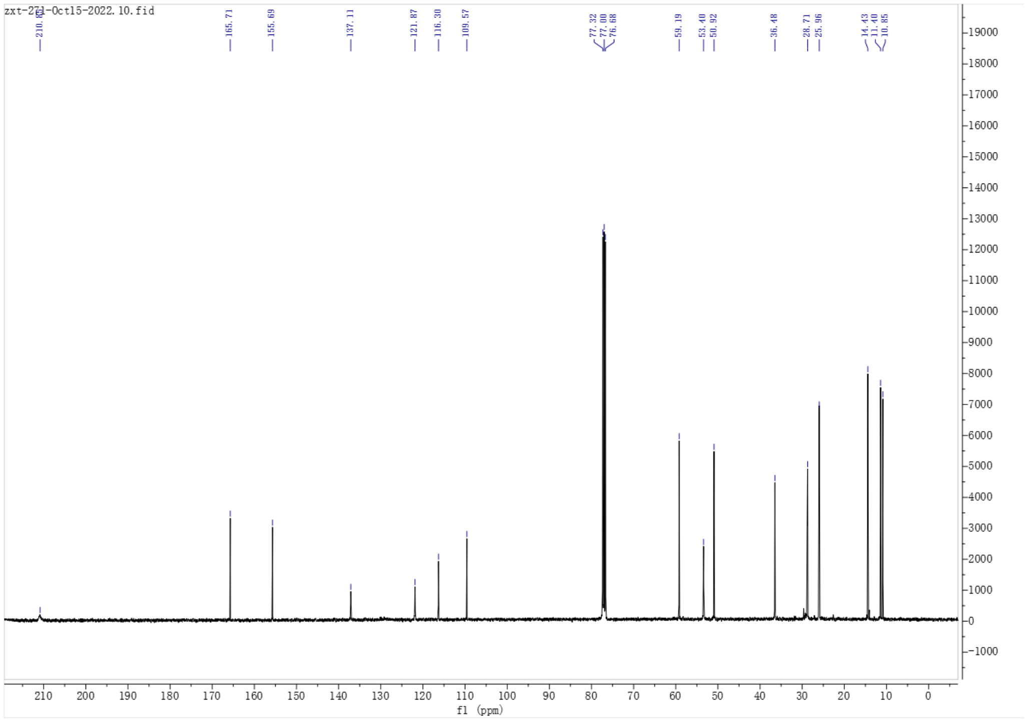
zxt-271-Oct15-2022.10.fid
165.71
155.69
137.11
121.87
116.30
109.57
77.32
77.00
76.68
59.19
53.40
50.92
36.48
28.71
25.96
14.43
11.40
10.85
f1 (ppm)

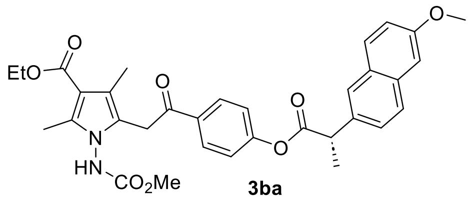

EtO
O
O
O
O
O
N
HN
CO2Me

3ba

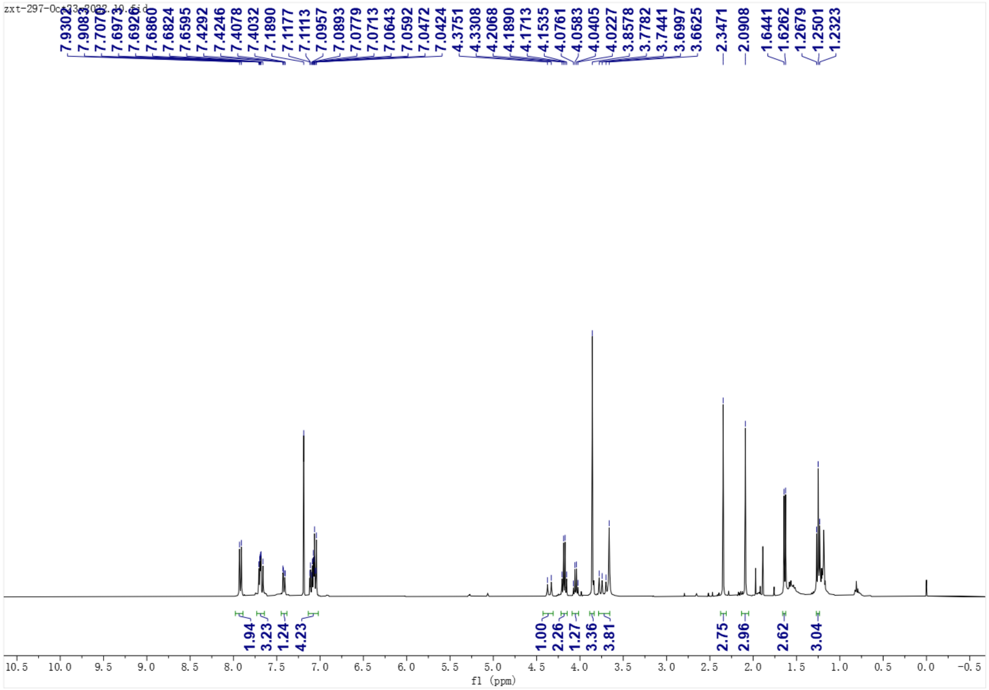

7.9302
7.9083
7.7073
7.6973
7.6926
7.6860
7.6824
7.6595
7.4292
7.4246
7.4078
7.4032
7.1890
7.1177
7.1113
7.0957
7.0893
7.0779
7.0713
7.0643
7.0592
7.0472
7.0424
4.3751
4.3308
4.2068
4.1890
4.1713
4.1535
4.0761
4.0583
4.0405
4.0227
3.8578
3.7782
3.7441
3.6997
3.6625
2.3471
2.0908
1.6441
1.6262
1.2679
1.2501
1.2323
1.94
3.23
1.24
4.23
1.00
2.26
1.27
3.36
3.81
2.75
2.96
2.62
3.04
f1 (ppm)

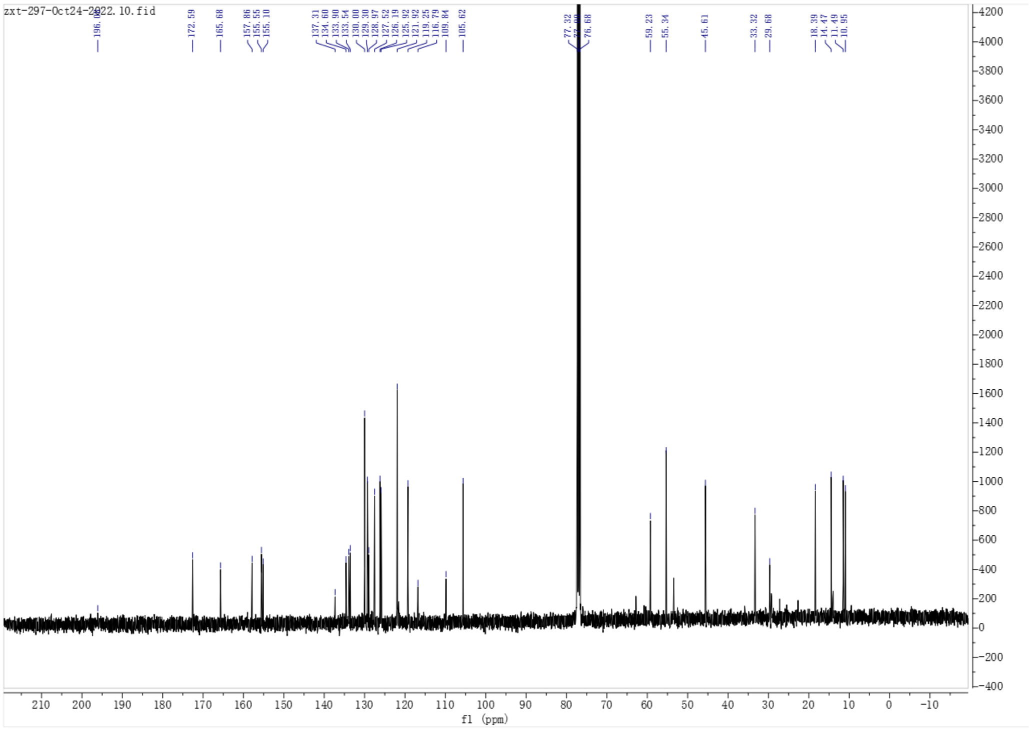

zxt-297-Oct24-2022.10.fid
172.59
165.68
157.86
155.55
155.10
137.31
134.60
133.90
133.54
130.00
129.30
128.97
127.52
126.19
125.92
121.92
119.25
116.79
109.84
105.62
77.32
76.68
59.23
55.34
45.61
33.32
29.68
18.39
14.47
11.49
10.95
f1 (ppm)

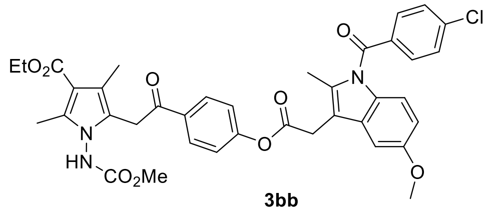
EtO2C
O
O
O
O
N
N
HN
CO2Me
Cl
O

3bb

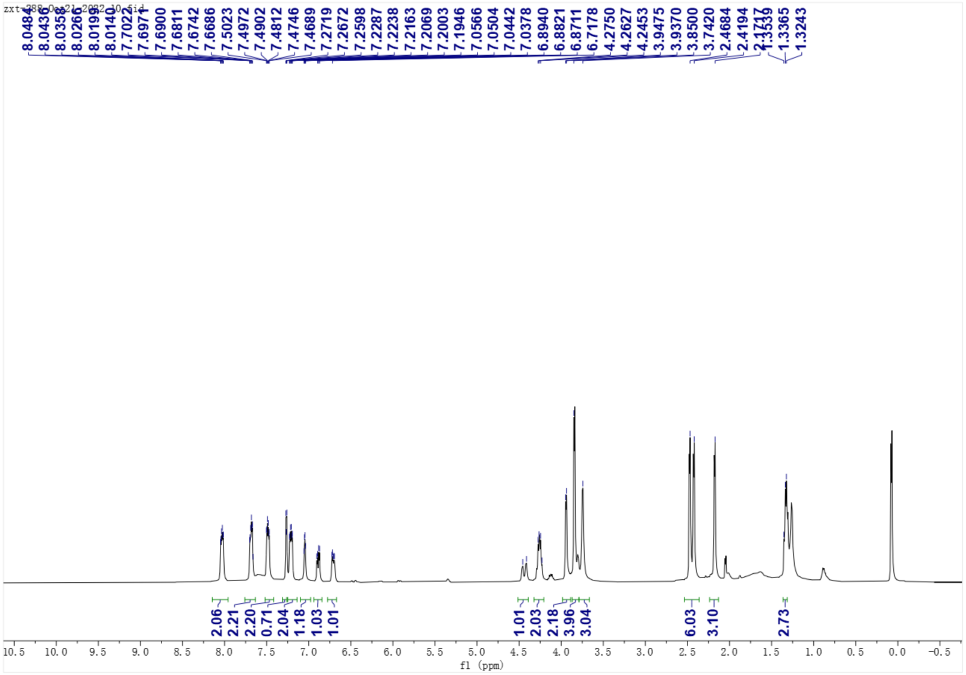
zxt-288-Oct21-2022.10.fid
8.0484
8.0430
8.0358
8.0265
8.0193
8.0140
7.7022
7.6971
7.6900
7.6811
7.6742
7.6686
7.5023
7.4972
7.4902
7.4812
7.4746
7.4689
7.2719
7.2672
7.2598
7.2287
7.2238
7.2163
7.2069
7.2003
7.1946
7.0566
7.0504
7.0442
7.0378
6.8940
6.8821
6.8711
6.7178
4.2750
4.2627
4.2453
3.9475
3.9370
3.8500
3.7420
2.4684
2.4194
2.1717
1.3539
1.3365
1.3243
2.06
2.21
2.20
0.71
2.04
1.18
1.03
1.01
1.01
2.03
2.18
3.96
3.04
6.03
3.10
2.73
10.5 10.0 9.5 9.0 8.5 8.0 7.5 7.0 6.5 6.0 5.5 5.0 4.5 4.0 3.5 3.0 2.5 2.0 1.5 1.0 0.5 0.0 -0.5
f1 (ppm)

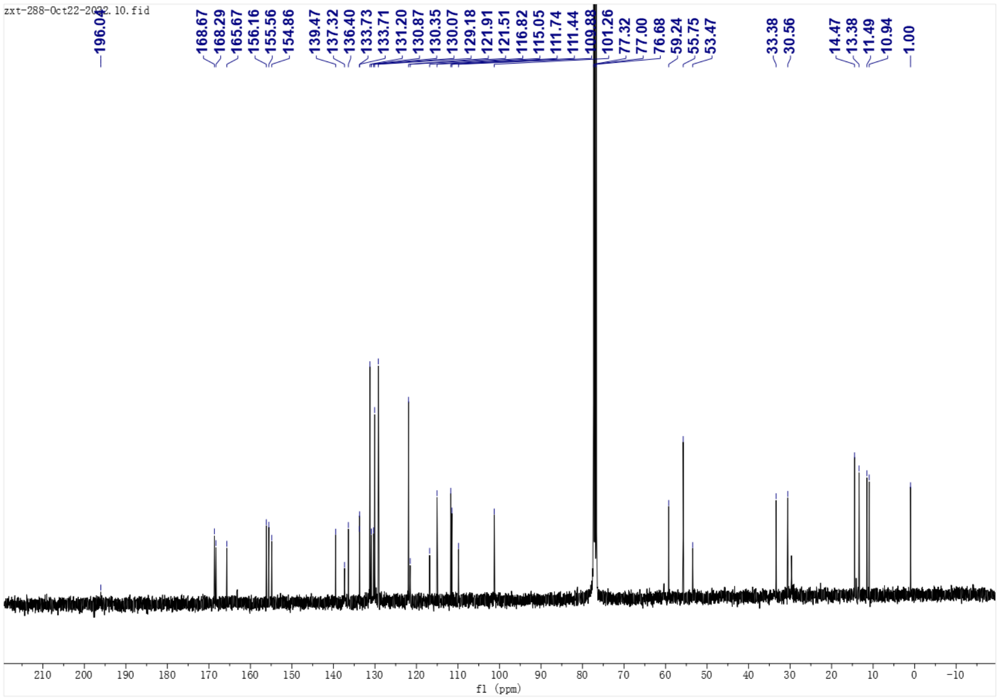
zxt-288-Oct22-2022.10.fid
196.04
168.67
168.29
165.67
156.16
155.56
154.86
139.47
137.32
136.40
133.73
133.71
131.20
130.87
130.35
130.07
129.18
121.91
121.51
116.82
115.05
111.74
111.44
109.88
101.26
77.32
77.00
76.68
59.24
55.75
53.47
33.38
30.56
14.47
13.38
11.49
10.94
1.00
210 200 190 180 170 160 150 140 130 120 110 100 90 80 70 60 50 40 30 20 10 0 -10
f1 (ppm)

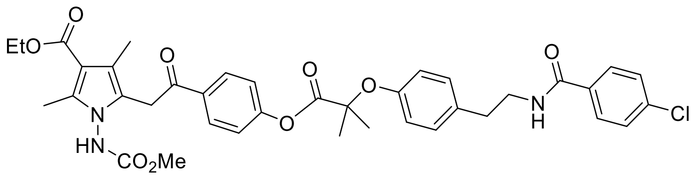

3bc

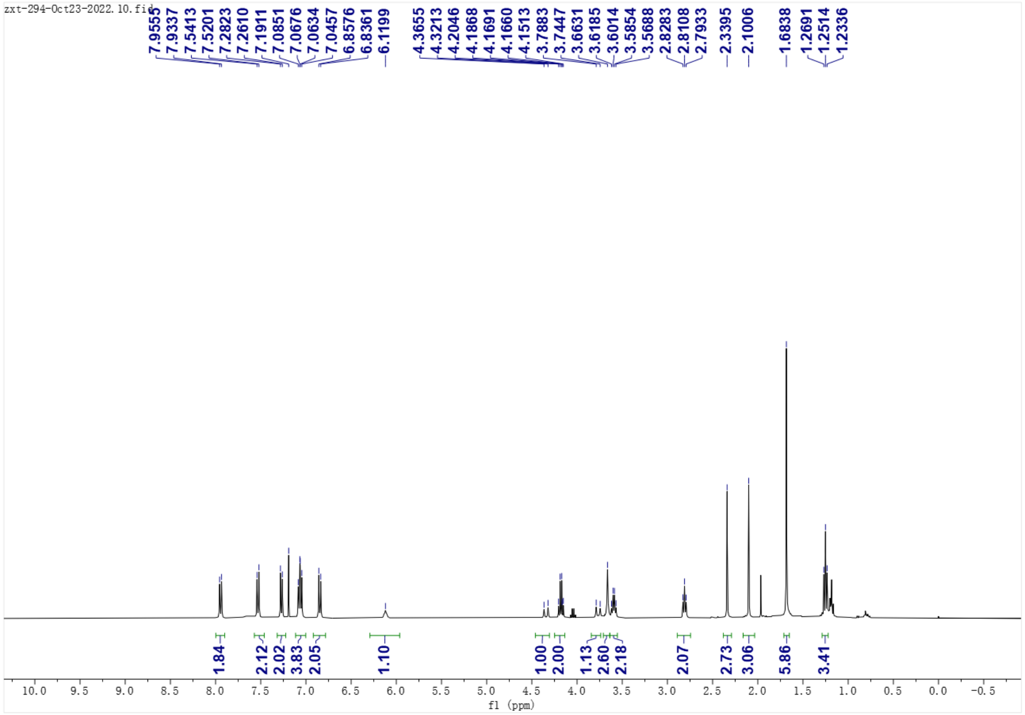

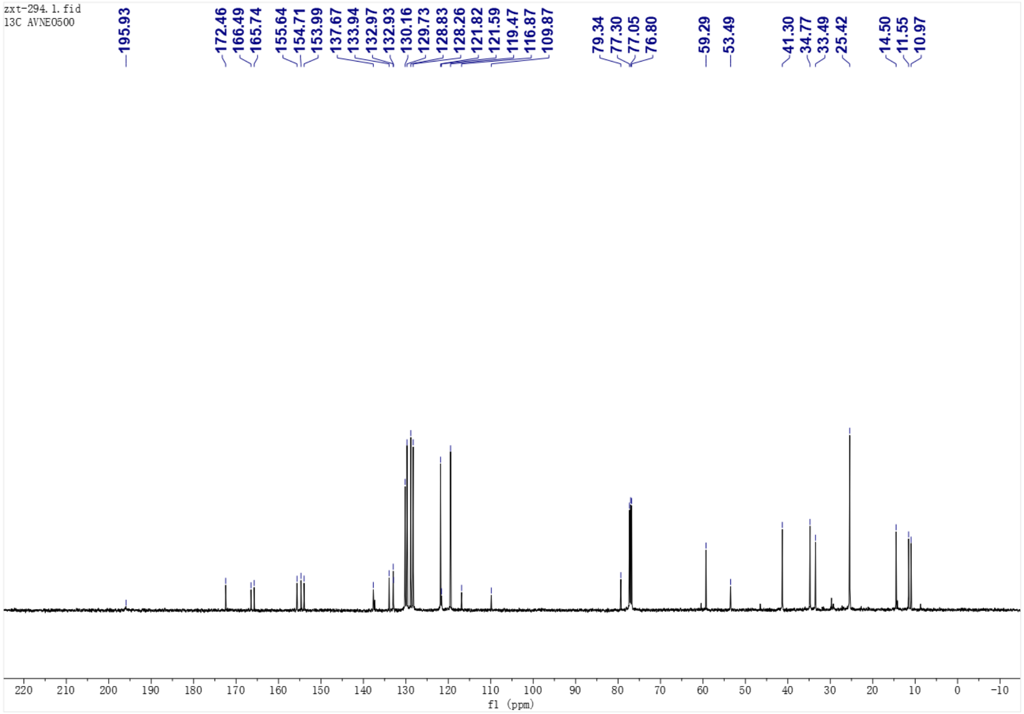

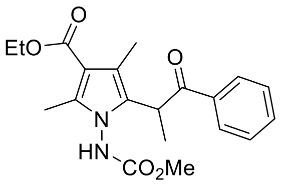

EtO
O
O
N
HN
CO2Me

3bd

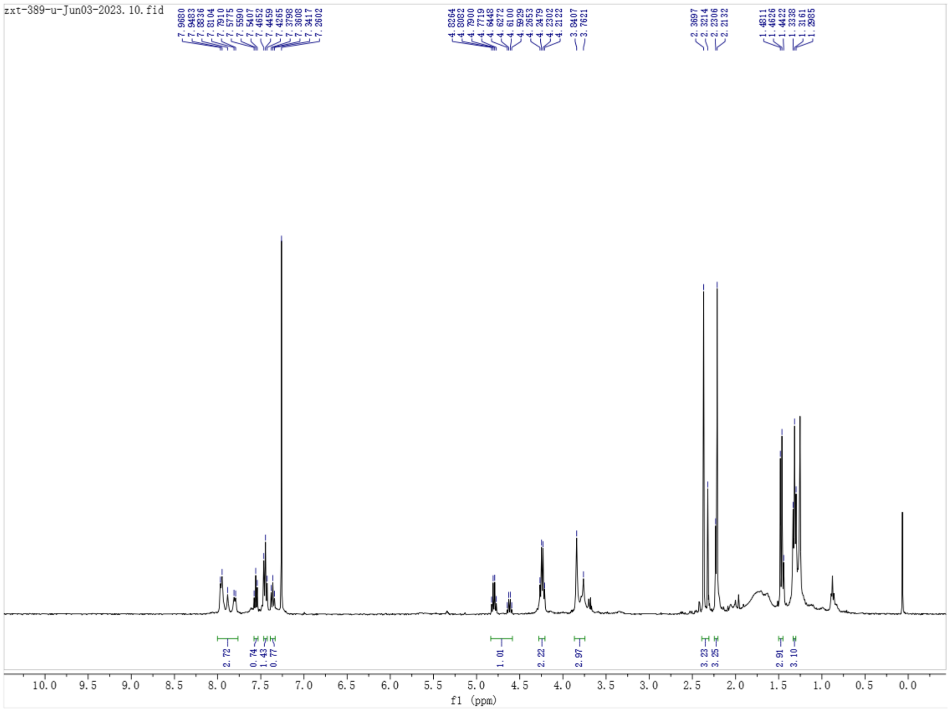

zxt-389-u-Jun03-2023.10.fid
f1 (ppm)

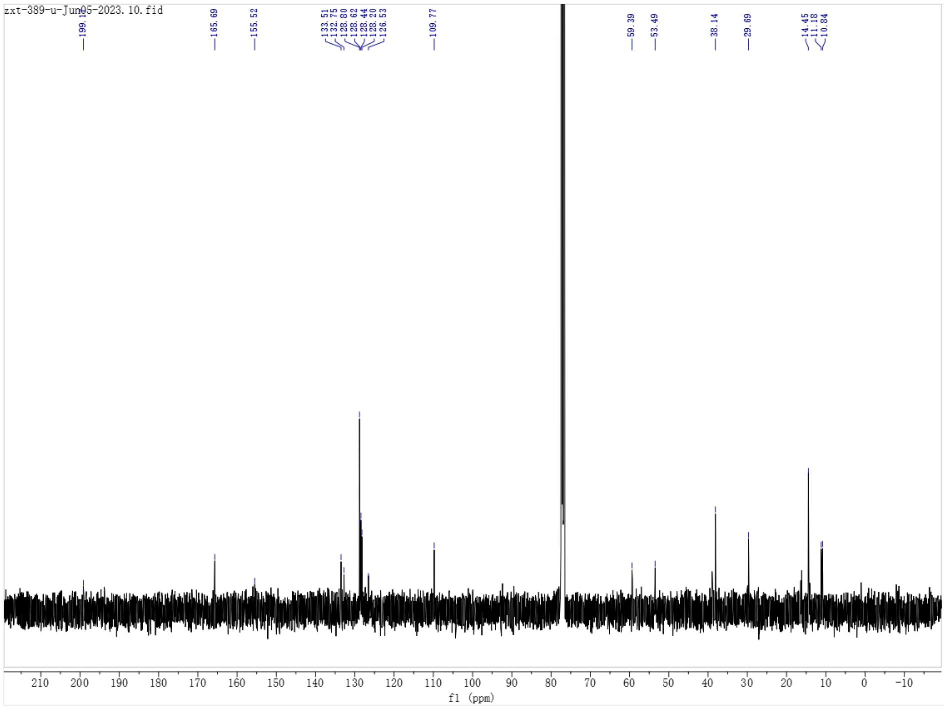

zxt-389-u-Jun05-2023.10.fid
165.69
155.52
133.51
132.75
128.80
128.62
128.44
128.20
126.53
109.77
59.39
53.49
38.14
29.69
14.45
11.18
10.84
f1 (ppm)

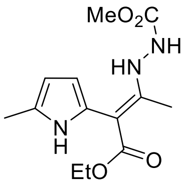

3be'

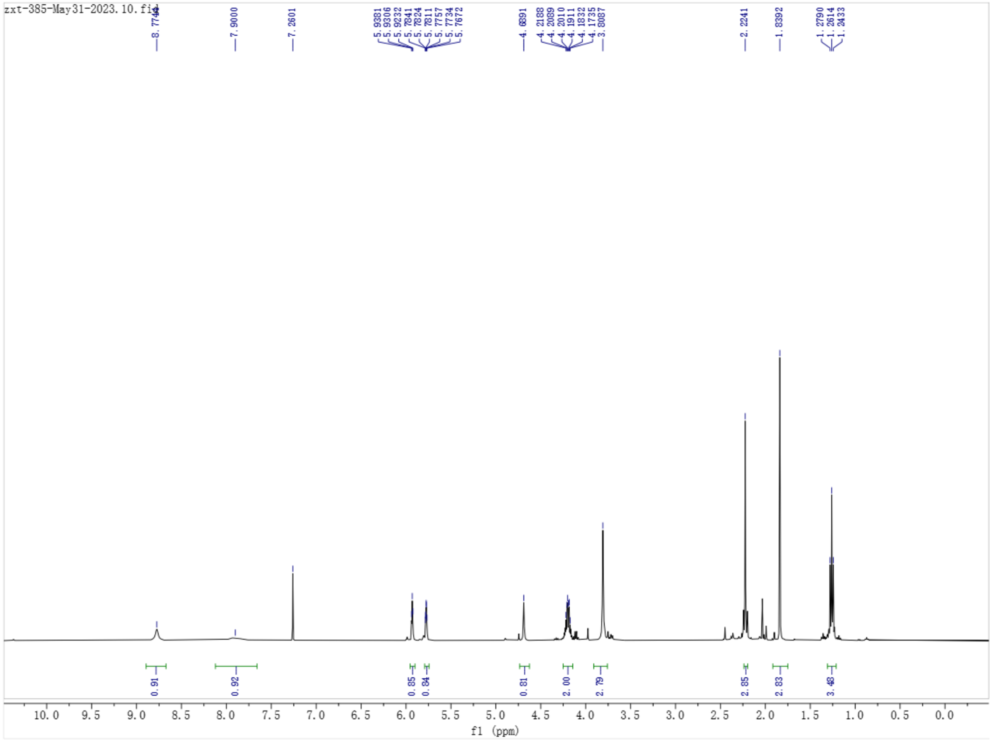

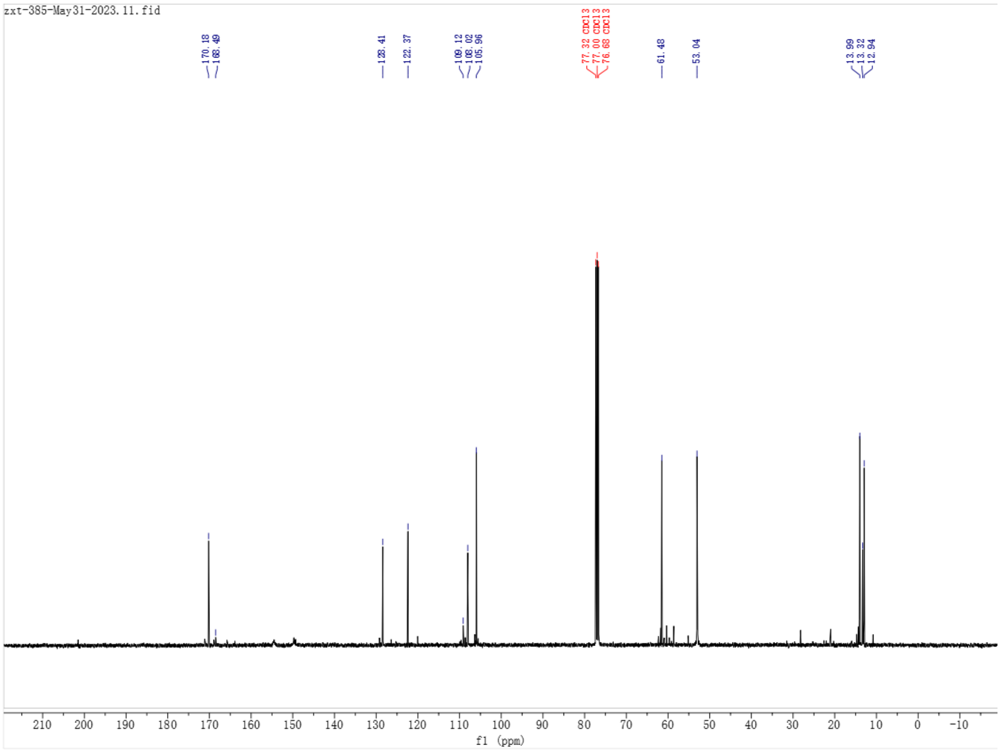

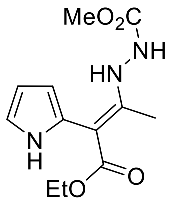

MeO2C
NH
HN
N
H
O
EtO

3bf'

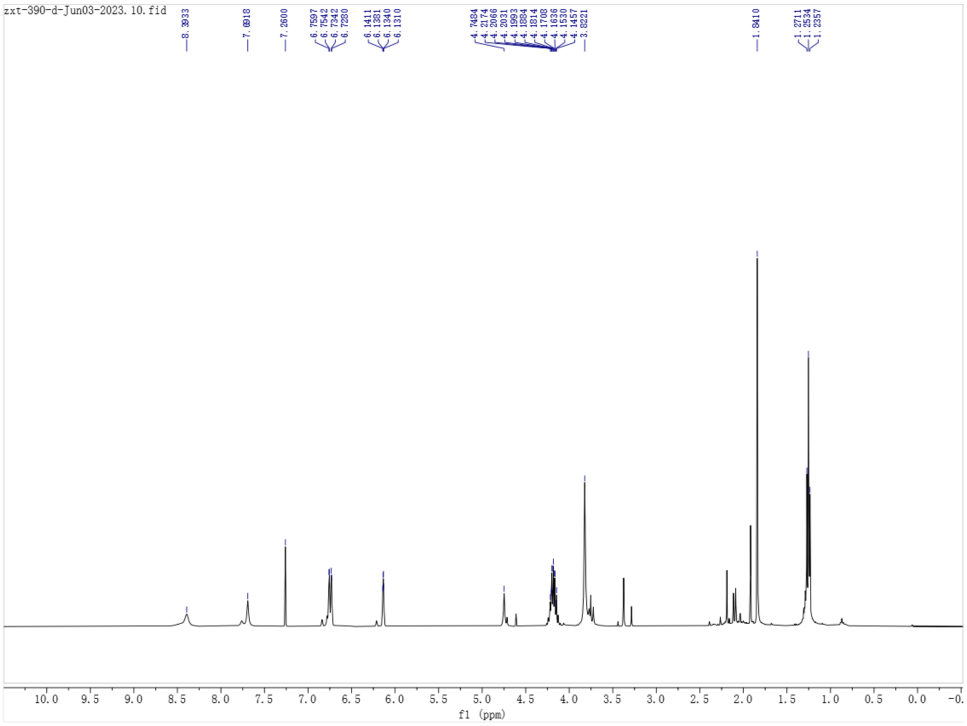

zxt-390-d-Jun03-2023.10.fid
8.3933
7.6918
7.2600
6.7597
6.7542
6.7342
6.7280
6.1411
6.1381
6.1340
6.1310
4.7484
4.2174
4.2066
4.2031
4.1993
4.1884
4.1814
4.1768
4.1636
4.1530
4.1457
3.8221
1.8410
1.2711
1.2534
1.2357
f1 (ppm)

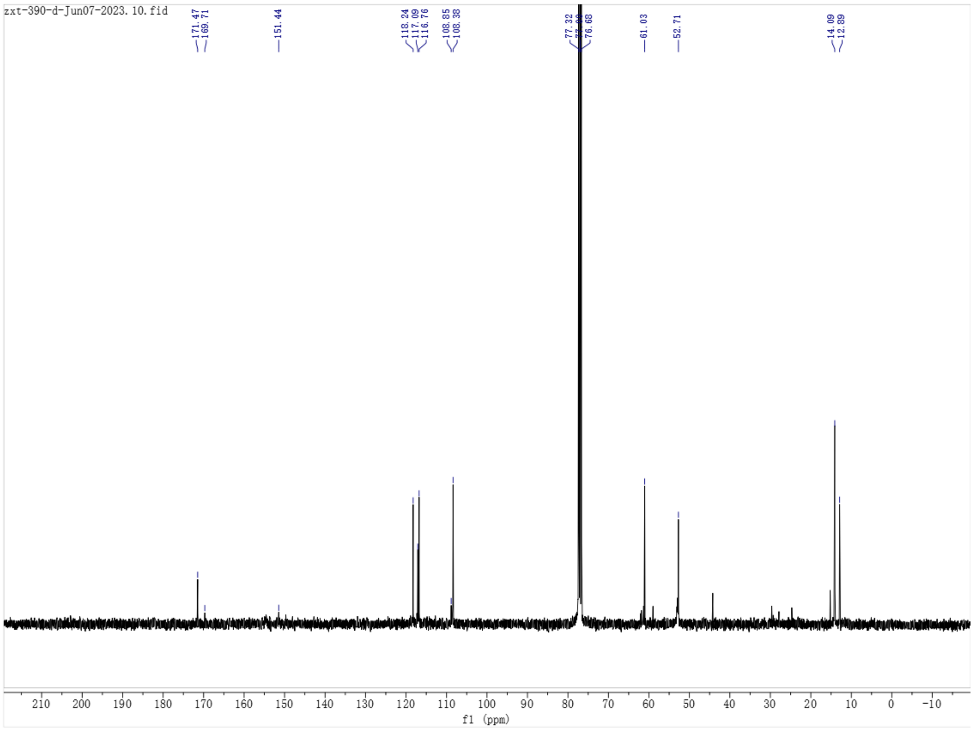

zxt-390-d-Jun07-2023.10.fid
171.47
169.71
151.44
118.24
117.09
116.76
108.85
108.38
77.32
76.68
61.03
52.71
14.09
12.89
f1 (ppm)

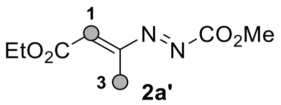
EtO2C
N
N
CO2Me
1
3
2a'

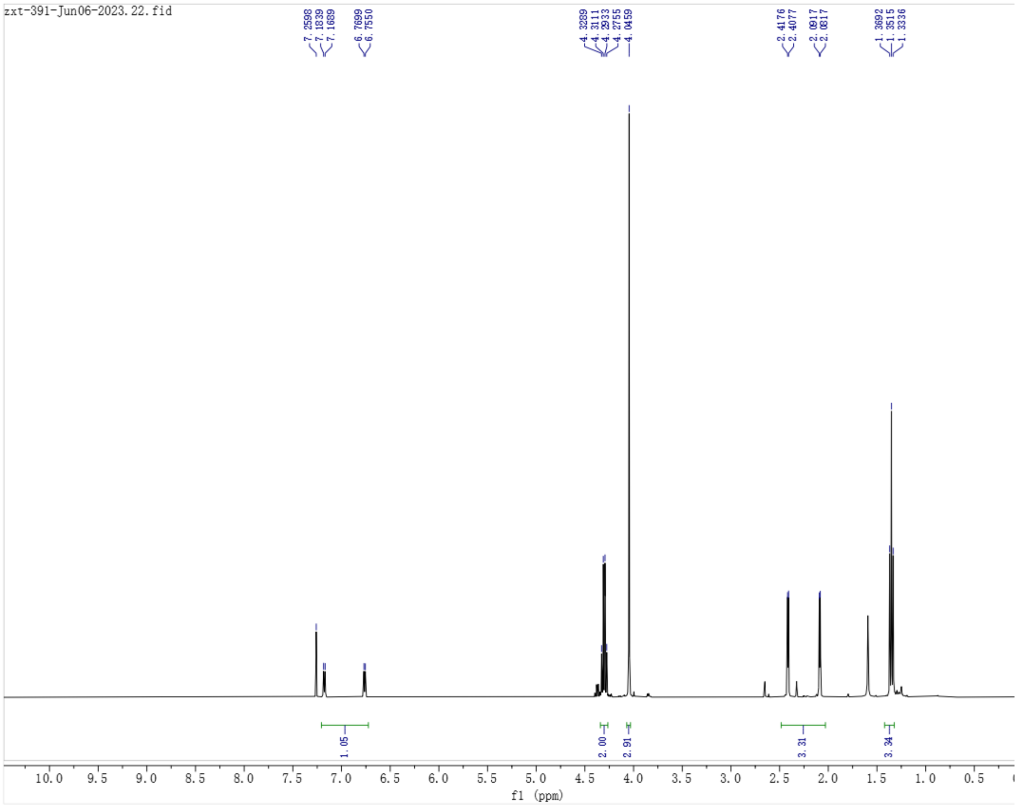
zxt-391-Jun06-2023.22.fid
7.2598
7.1839
7.1689
6.7699
6.7550
4.3289
4.3111
4.2933
4.2755
4.0459
2.4176
2.4077
2.0917
2.0817
1.3692
1.3515
1.3336
1.05
2.00
2.91
3.31
3.34
10.0 9.5 9.0 8.5 8.0 7.5 7.0 6.5 6.0 5.5 5.0 4.5 4.0 3.5 3.0 2.5 2.0 1.5 1.0 0.5
f1 (ppm)

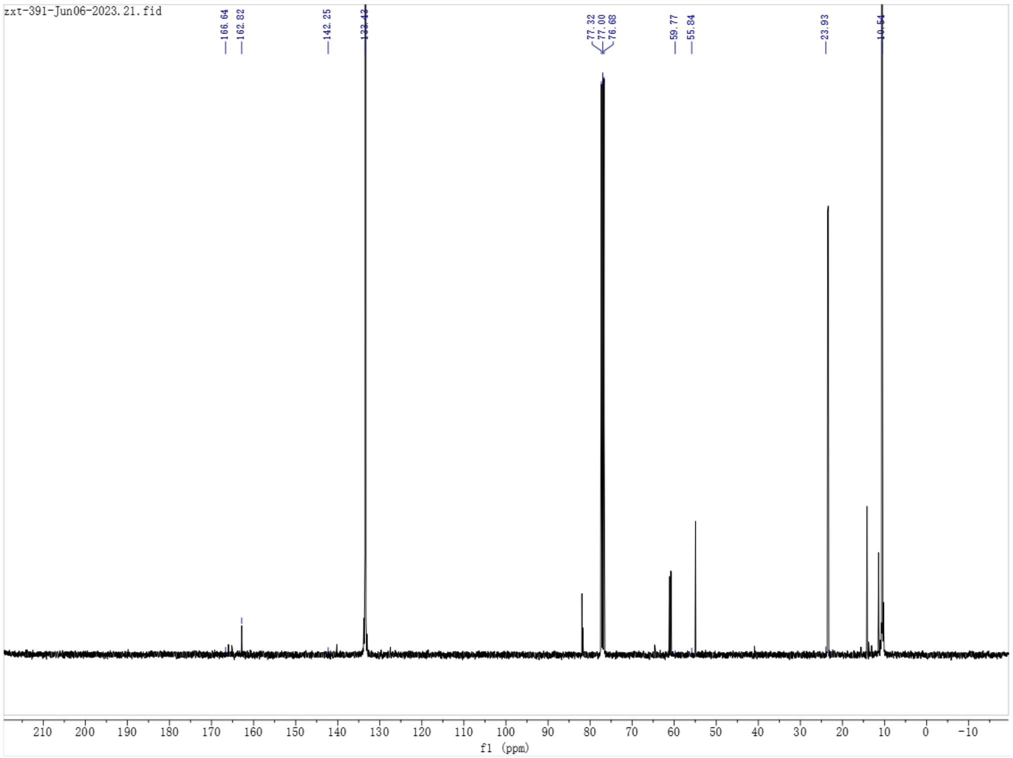
zxt-391-Jun06-2023.21.fid
166.64
162.82
142.25
133.42
77.32
77.00
76.68
59.77
55.84
23.93
10.54
210 200 190 180 170 160 150 140 130 120 110 100 90 80 70 60 50 40 30 20 10 0 -10
f1 (ppm)

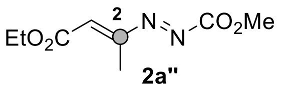
2
EtO2C
N
N
CO2Me

2a''

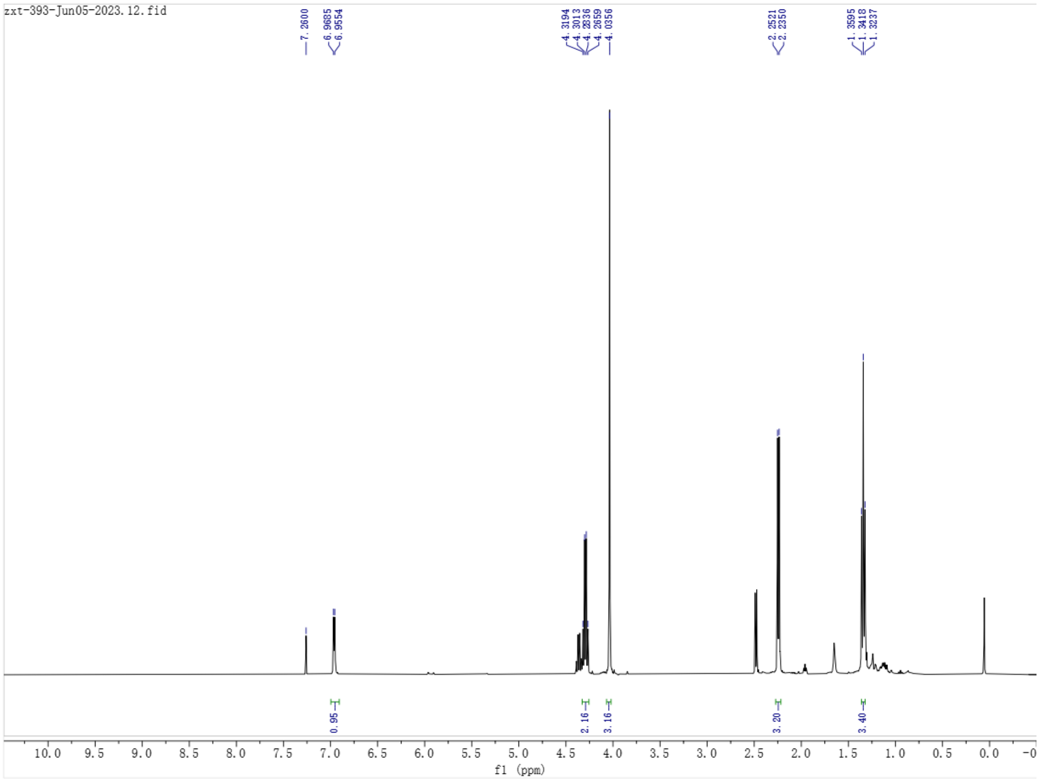
zxt-393-Jun05-2023.12.fid
7.2600
6.9685
6.9554
4.3194
4.3013
4.2836
4.2659
4.0356
2.2521
2.2350
1.3595
1.3418
1.3237
0.95
2.16
3.16
3.20
3.40
f1 (ppm)

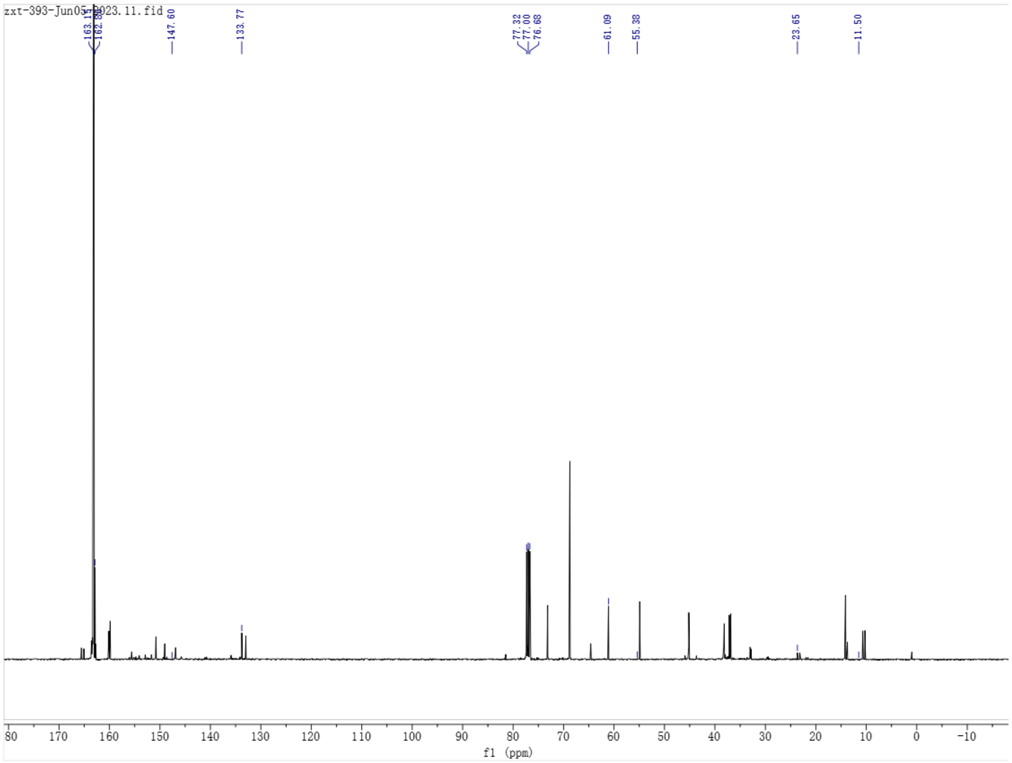
zxt-393-Jun05-2023.11.fid
163.15
162.88
147.60
133.77
77.32
77.00
76.68
61.09
55.38
23.65
11.50
f1 (ppm)

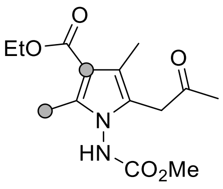

3aa'

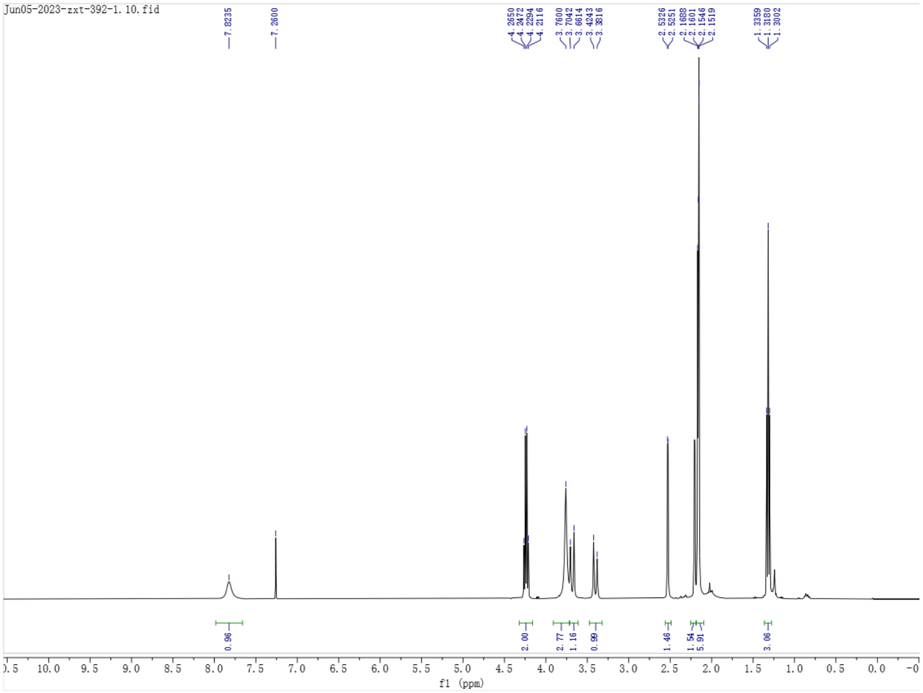

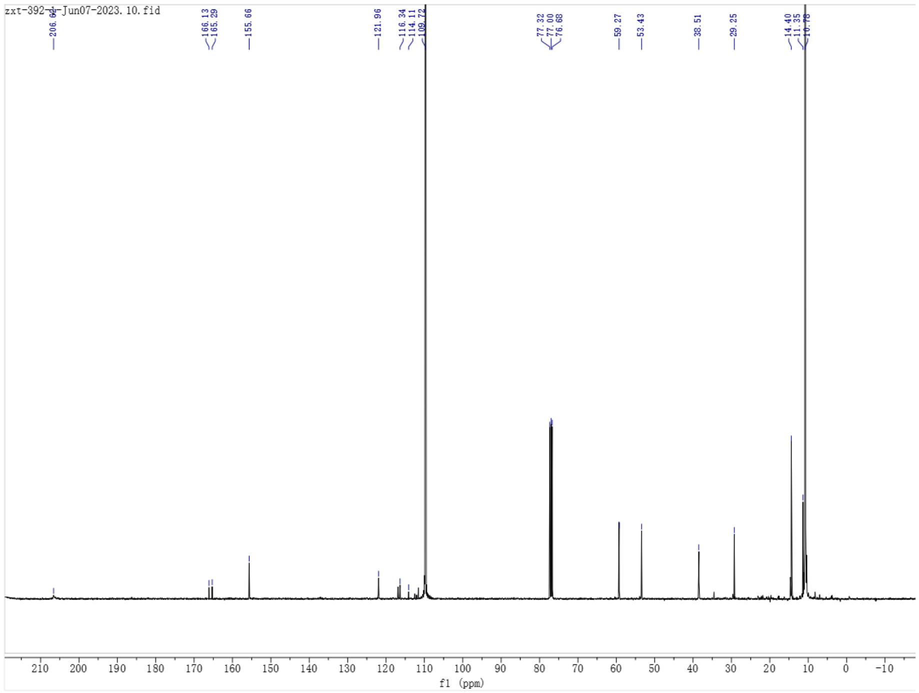

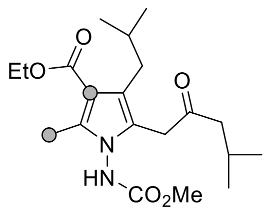

3ae'

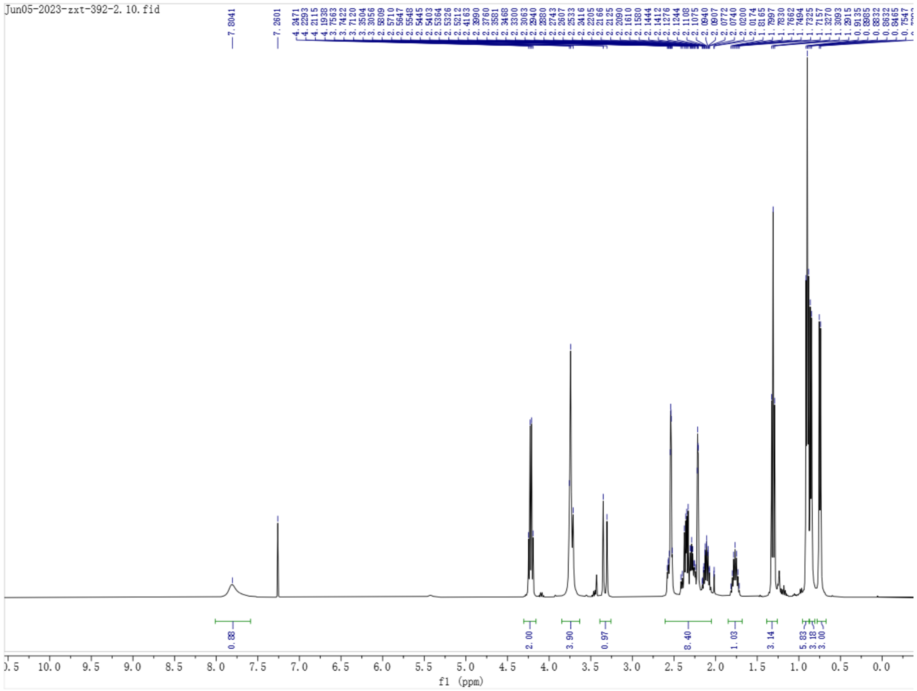

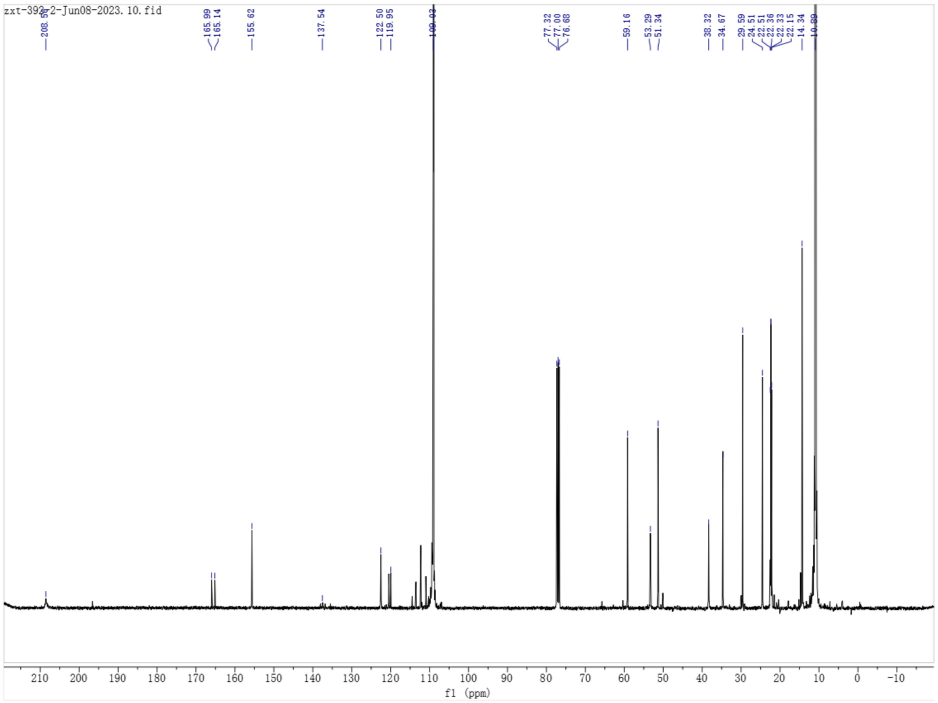

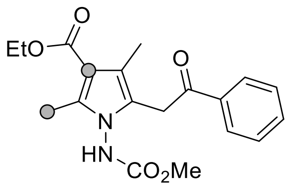
EtO
O
O
N
HN
CO2Me

3ai'

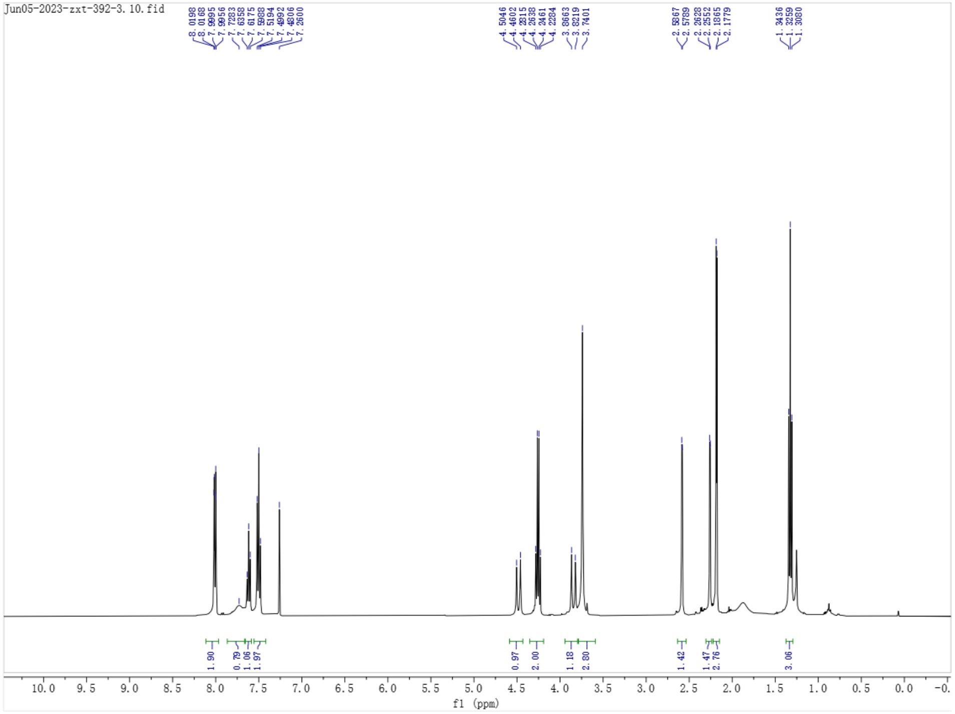
Jun05-2023-zxt-392-3.10.fid
8.0198
8.0168
7.9995
7.9956
7.7283
7.6358
7.6175
7.5988
7.5194
7.4992
7.4806
7.2600
4.5046
4.4602
4.2815
4.2638
4.2461
4.2284
3.8663
3.8219
3.7401
2.5867
2.5789
2.2628
2.2552
2.1865
2.1779
1.3436
1.3259
1.3080
1.90
0.79
1.06
1.97
0.97
2.00
1.18
2.80
1.42
1.47
2.76
3.06
10.0 9.5 9.0 8.5 8.0 7.5 7.0 6.5 6.0 5.5 5.0 4.5 4.0 3.5 3.0 2.5 2.0 1.5 1.0 0.5 0.0
f1 (ppm)

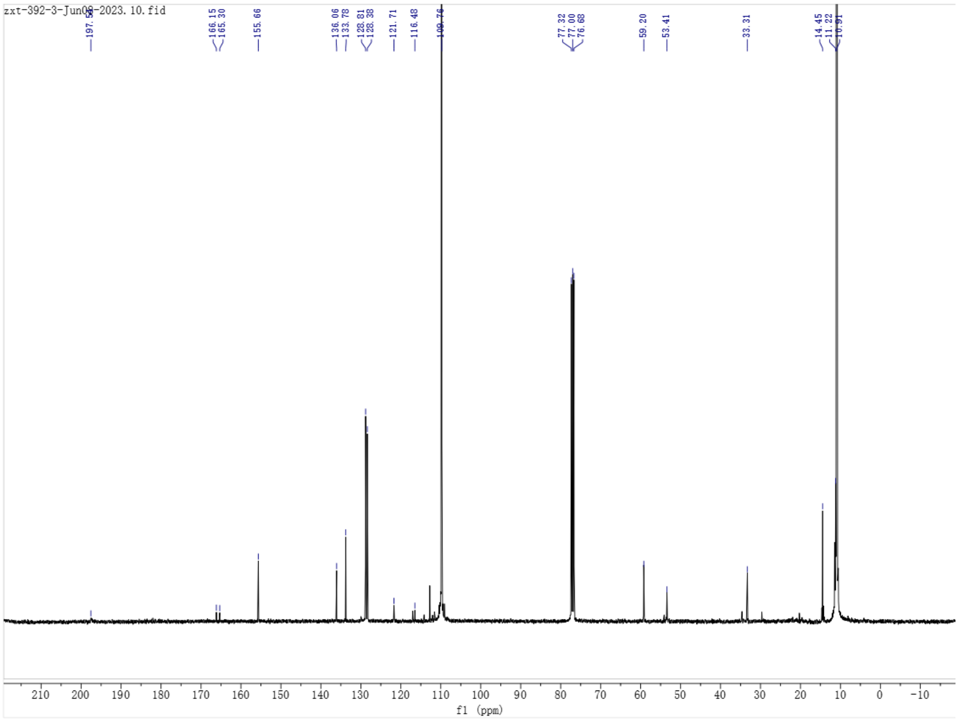
166.15
165.30
155.66
136.06
133.78
128.81
128.38
121.71
116.48
77.32
77.00
76.68
59.20
53.41
33.31
14.45
11.22
210 200 190 180 170 160 150 140 130 120 110 100 90 80 70 60 50 40 30 20 10 0 -10
f1 (ppm)

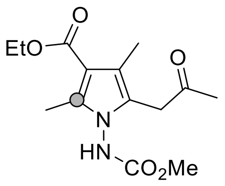
EtO
O
O
N
HN
CO2Me

3aa''

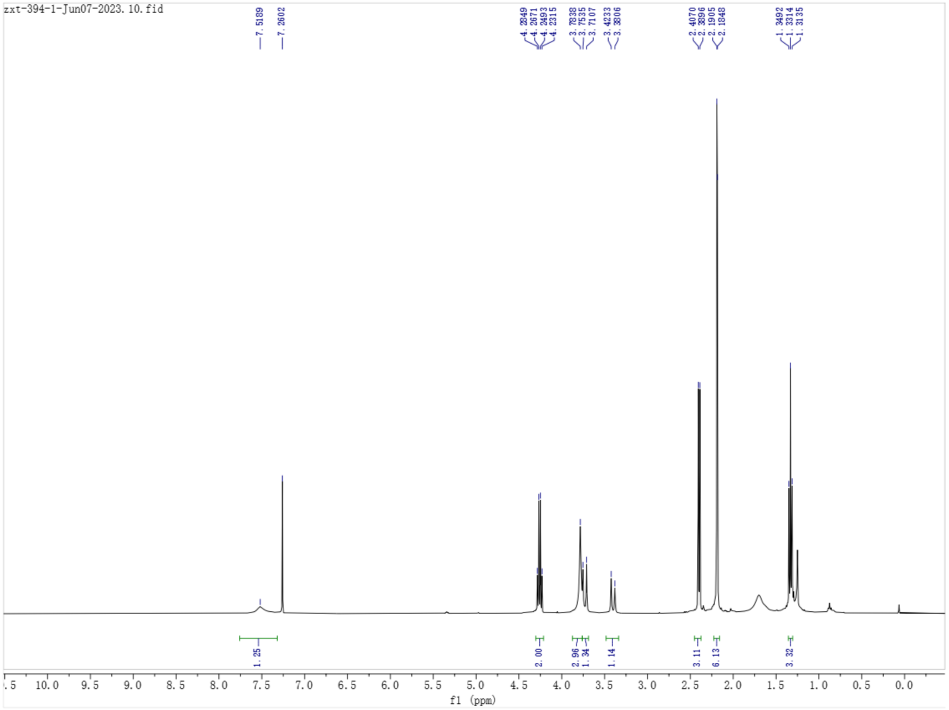
zxt-394-1-Jun07-2023.10.fid
7.5189
7.2602
4.2849
4.2671
4.2493
4.2315
3.7838
3.7535
3.7107
3.4233
3.3806
2.4070
2.3896
2.1905
2.1848
1.3492
1.3314
1.3135
1.25
2.00
2.96
1.34
1.14
3.11
6.13
3.32
f1 (ppm)

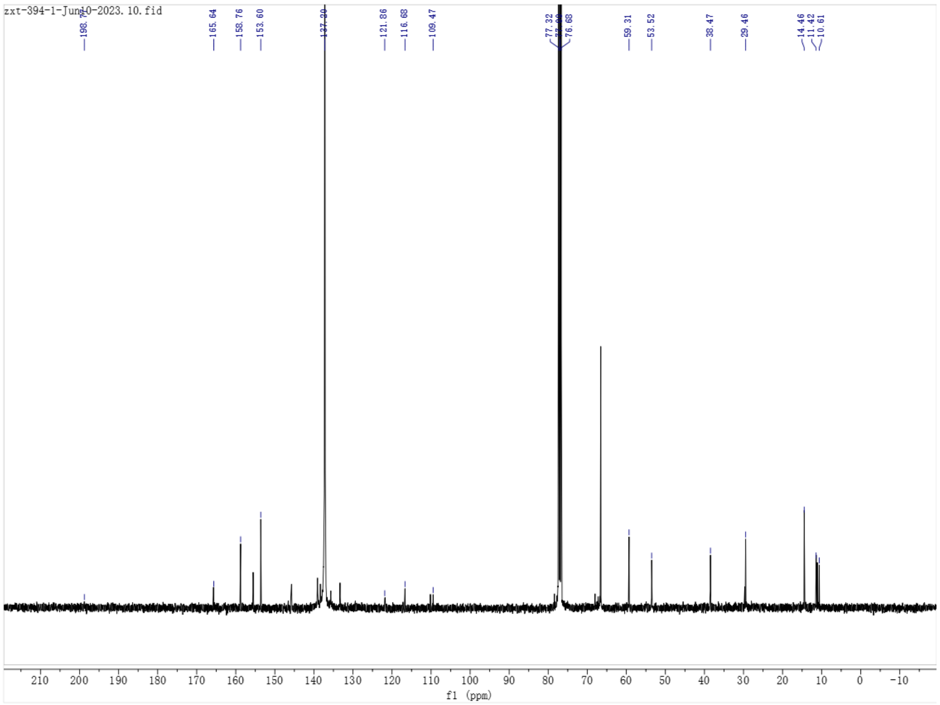
165.64
158.76
153.60
121.86
116.68
109.47
77.32
76.68
59.31
53.52
38.47
29.46
14.46
11.42
10.61
f1 (ppm)

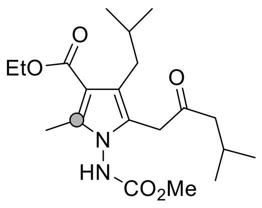

3ae''

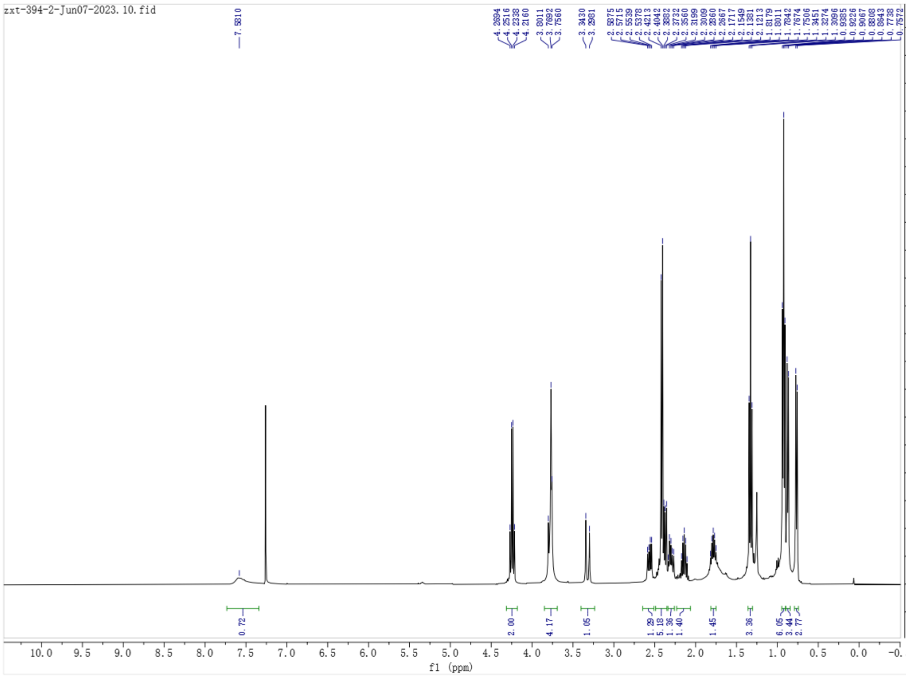

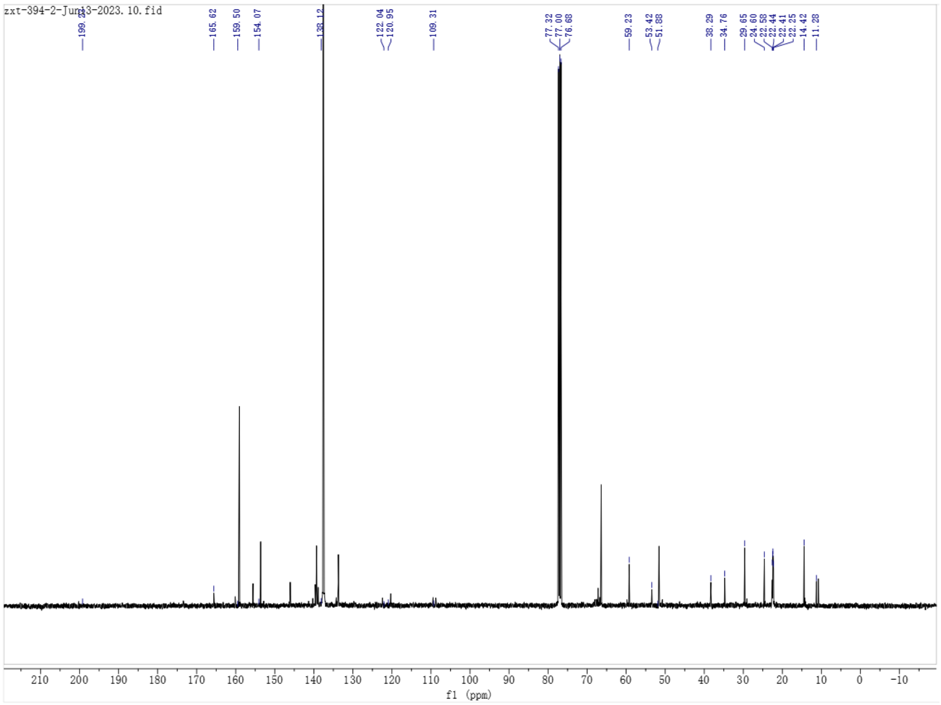

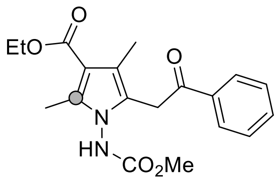
EtO
O
O
N
HN
CO2Me

3ai''

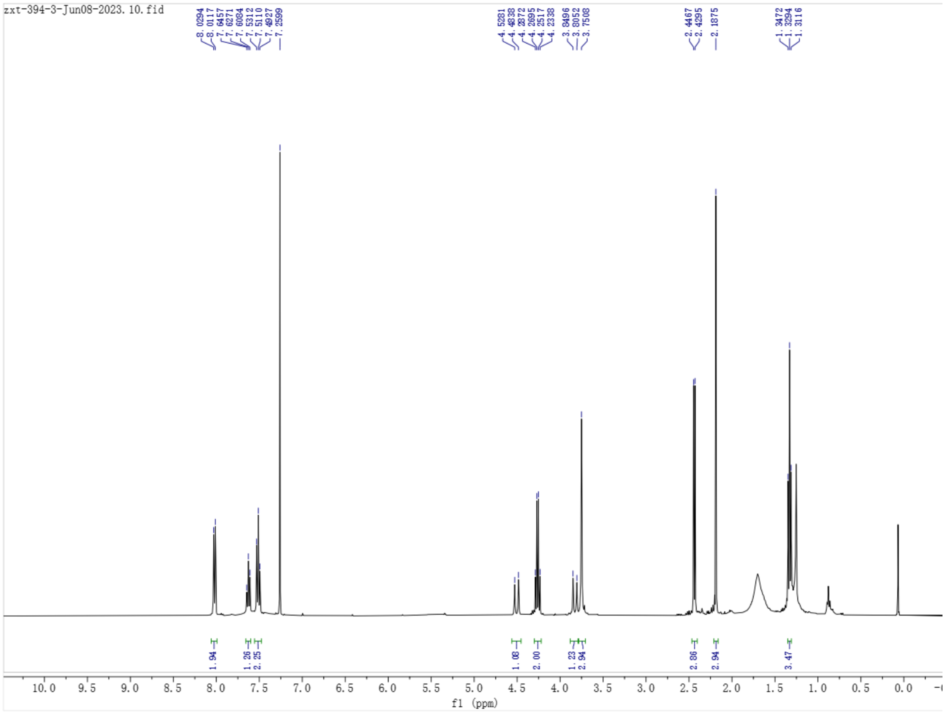
zxt-394-3-Jun08-2023.10.fid
8.0294
8.0117
7.6457
7.6271
7.6084
7.5312
7.5110
7.4927
7.2599
4.5281
4.4838
4.2872
4.2695
4.2517
4.2338
3.8496
3.8052
3.7508
2.4467
2.4295
2.1875
1.3472
1.3294
1.3116
1.94
1.26
2.25
1.00
2.00
1.23
2.94
2.86
2.94
3.47
f1 (ppm)

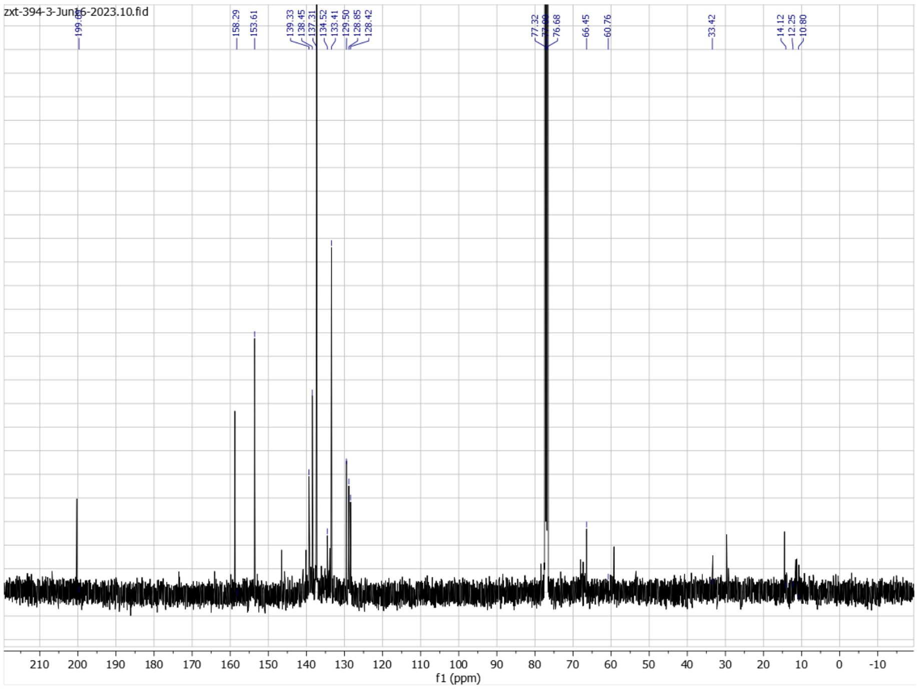
158.29
153.61
139.33
138.45
137.31
134.52
133.41
129.50
128.85
128.42
77.32
76.68
66.45
60.76
33.42
14.12
12.25
10.80
f1 (ppm)

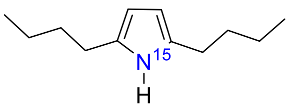

1d'

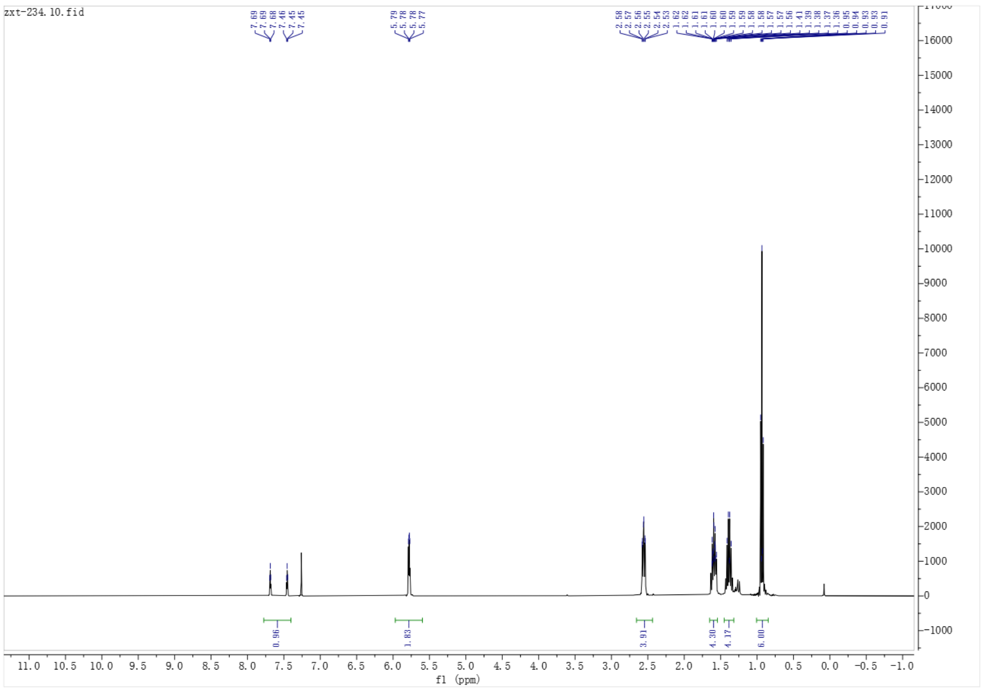

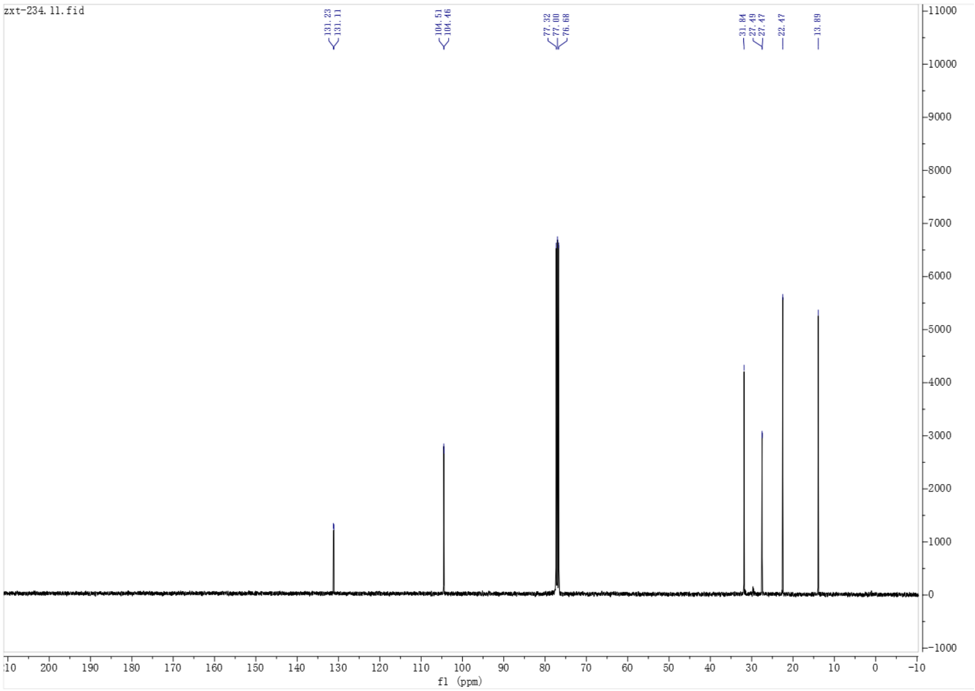

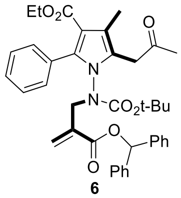

6

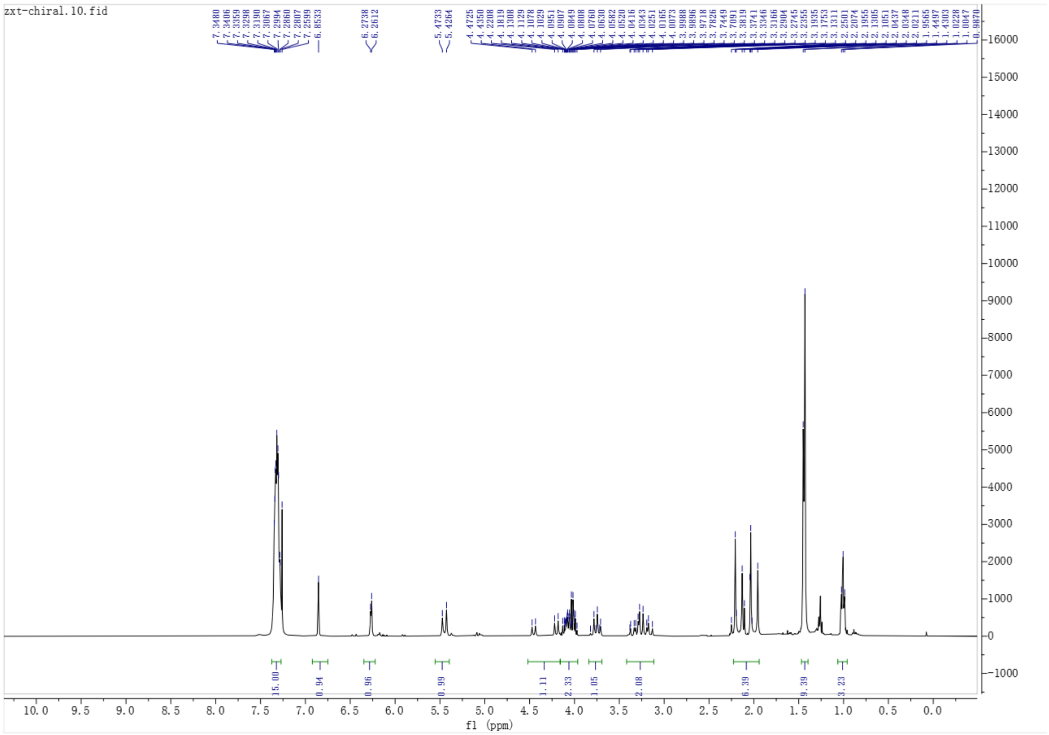

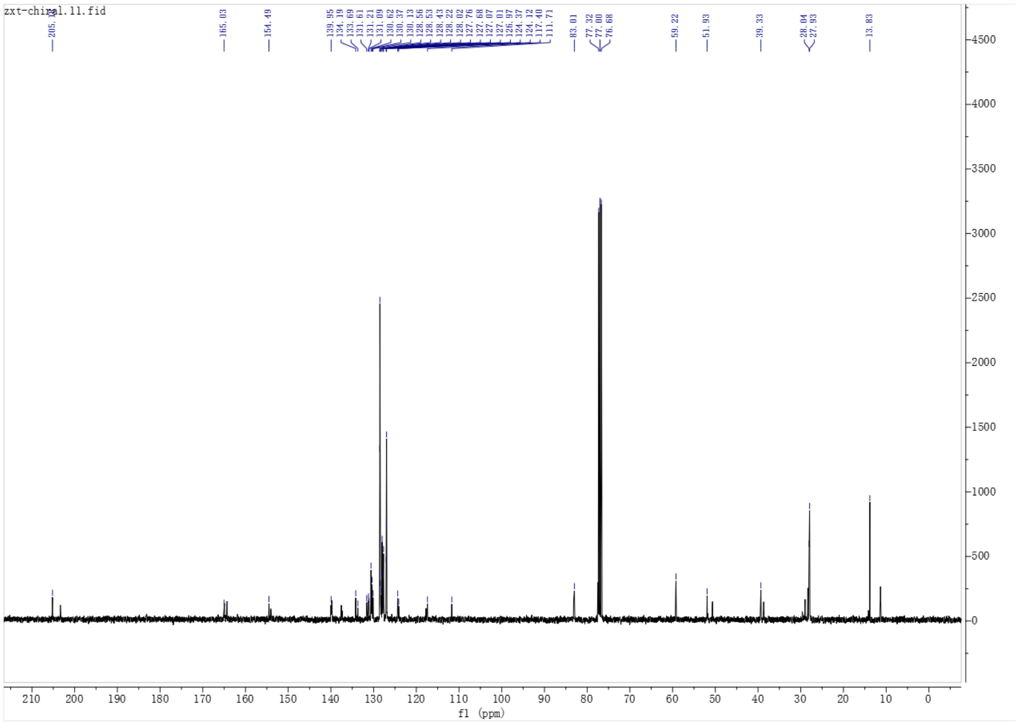

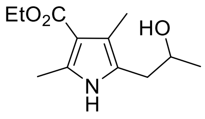

7a

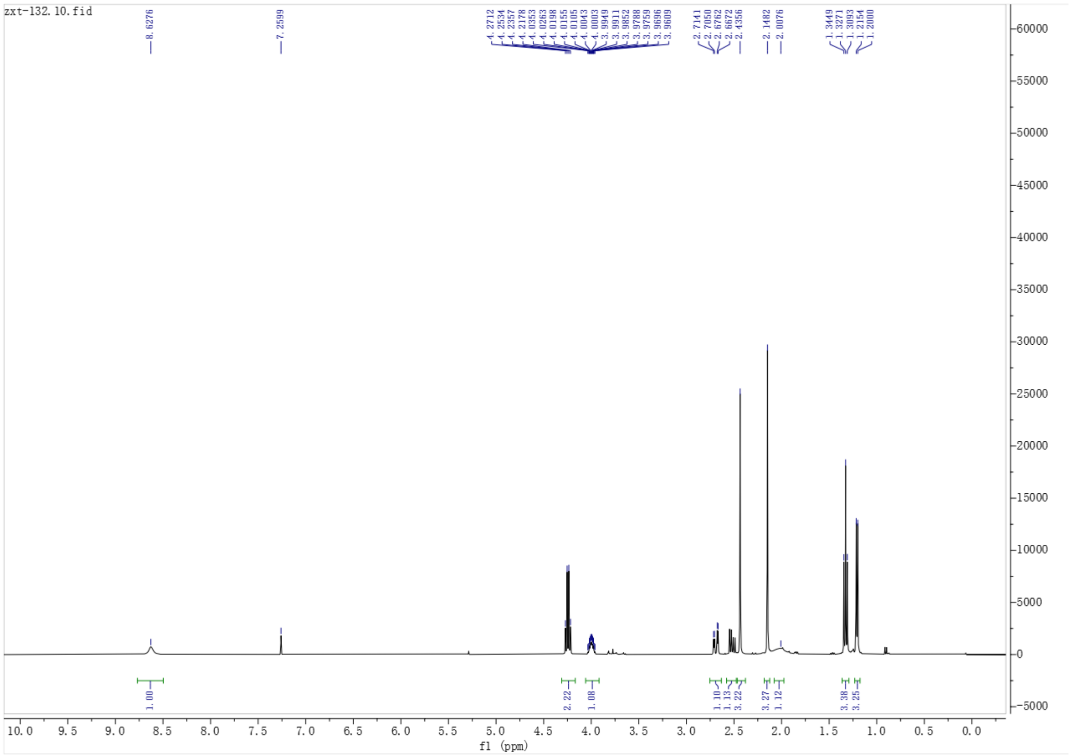

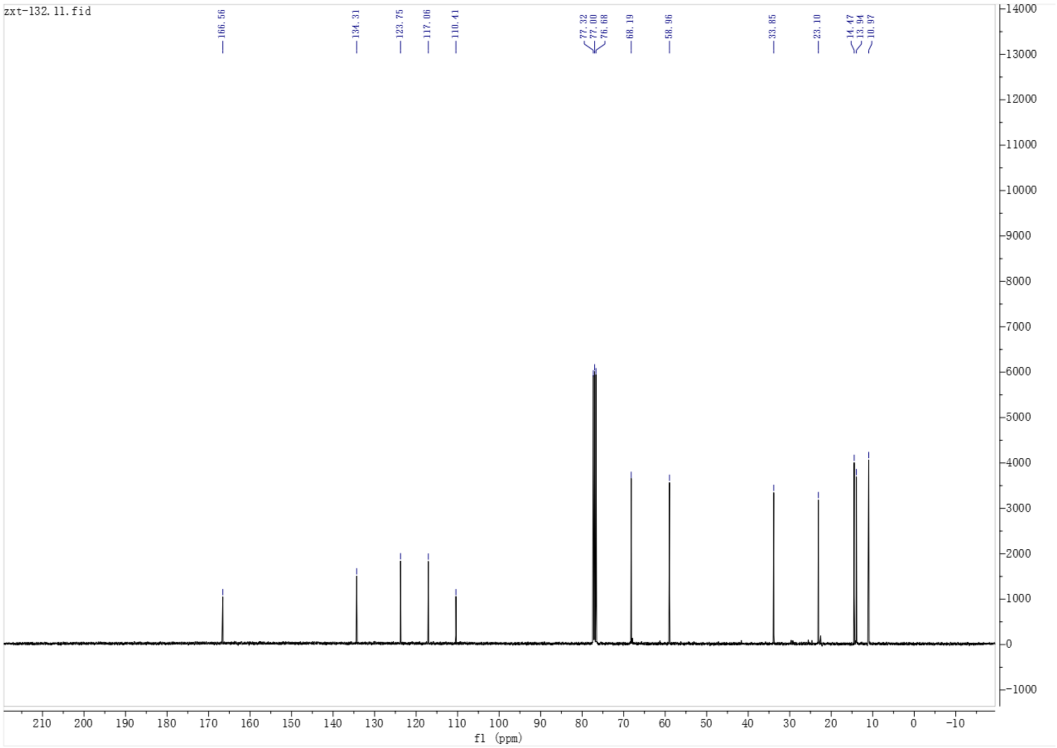

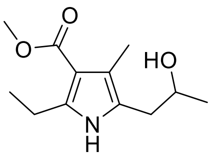

7b

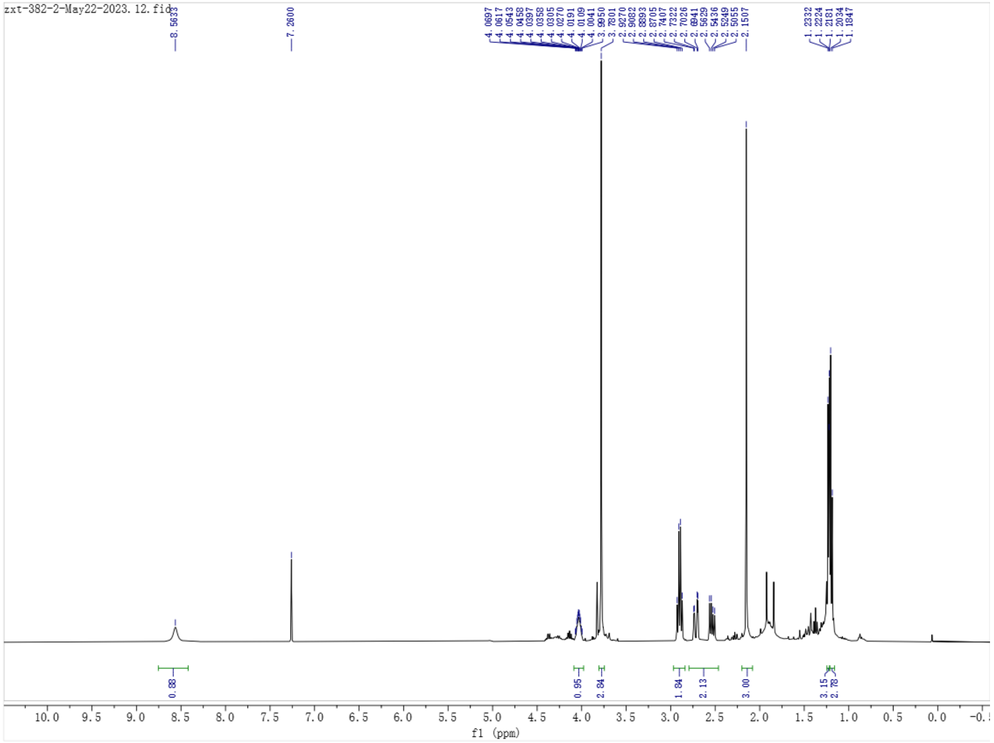

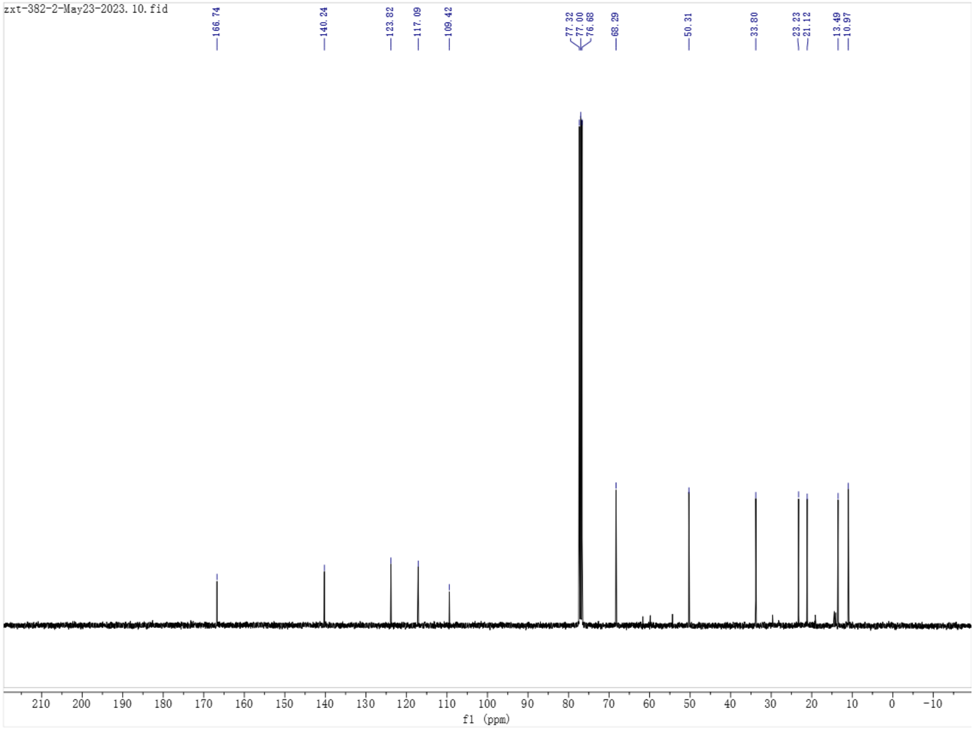

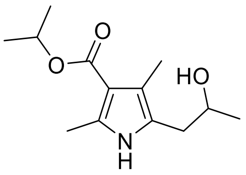
O
O
HO
N
H

7c

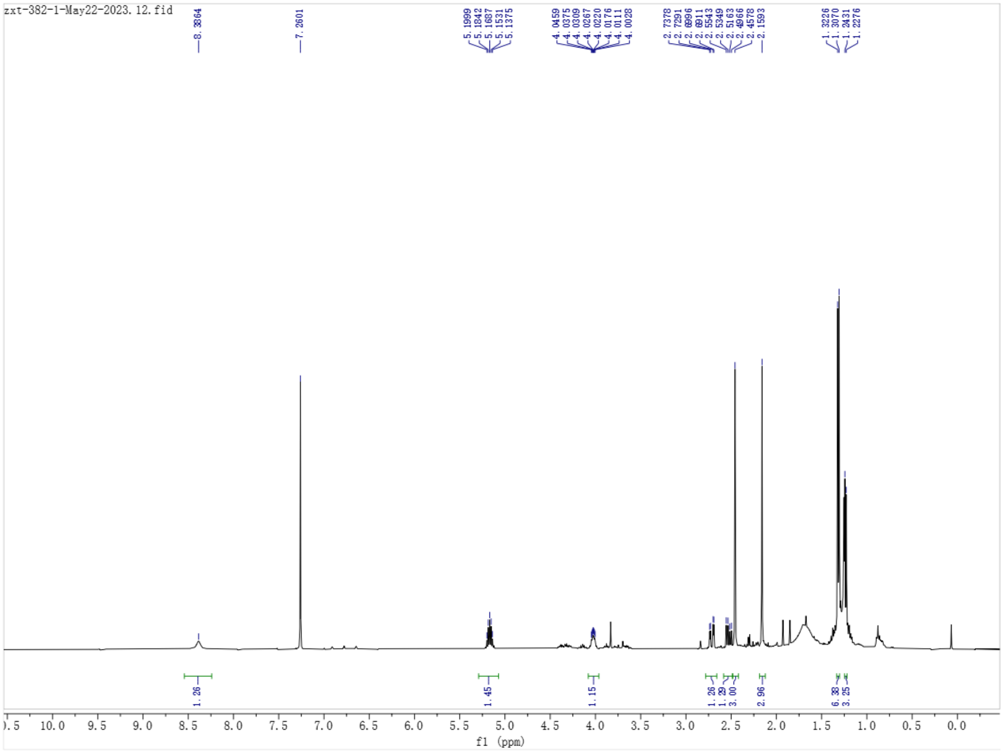
zxt-382-1-May22-2023.12.fid
8.3864
7.2601
5.1999
5.1842
5.1687
5.1531
5.1375
4.0459
4.0375
4.0267
4.0220
4.0176
4.0028
2.7378
2.7291
2.5543
2.5349
2.5163
2.4578
2.1593
1.3226
1.3070
1.2431
1.2276
f1 (ppm)

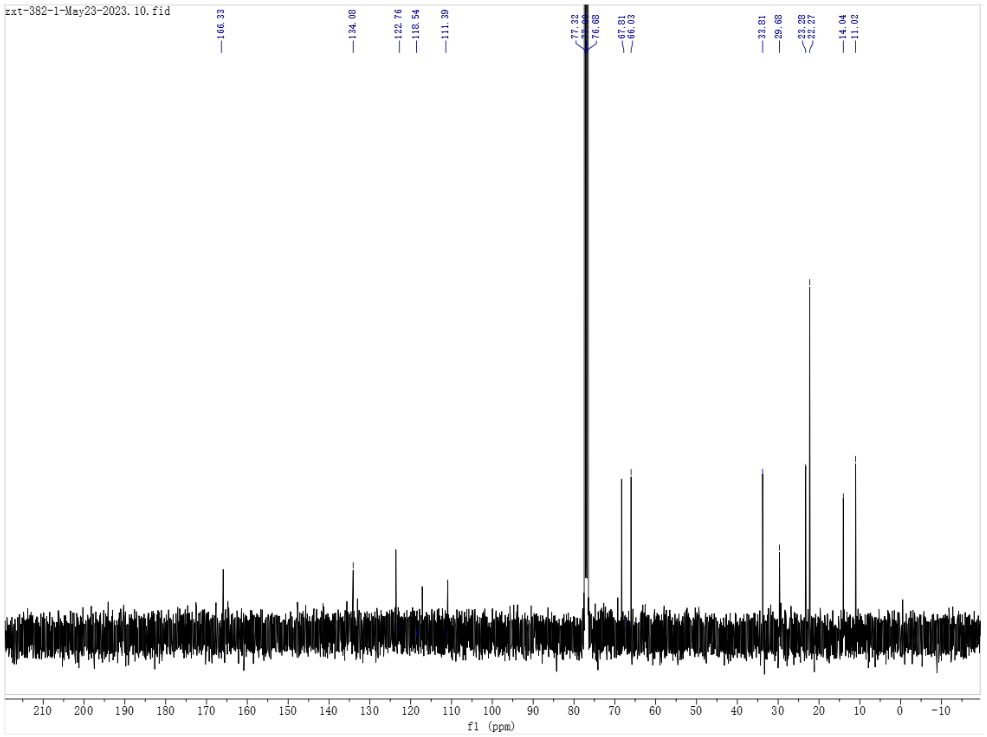
zxt-382-1-May23-2023.10.fid
166.33
134.08
122.76
118.54
111.39
77.32
76.68
67.81
66.03
33.81
29.68
23.28
22.27
14.04
11.02
f1 (ppm)

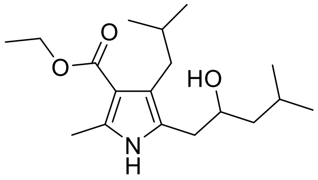

7d

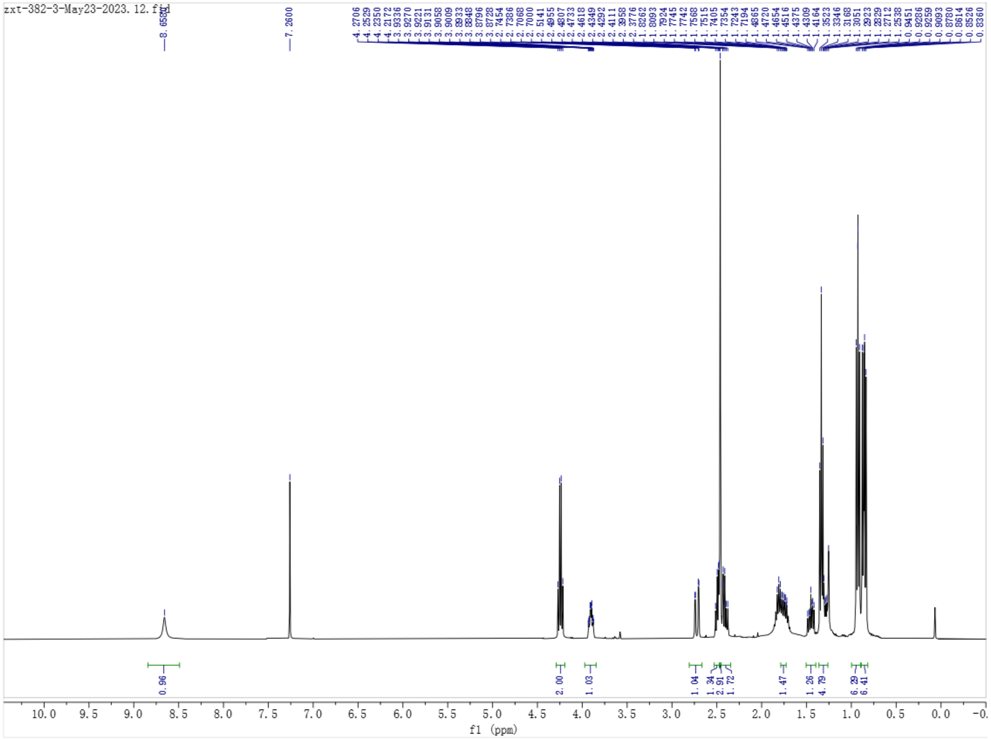

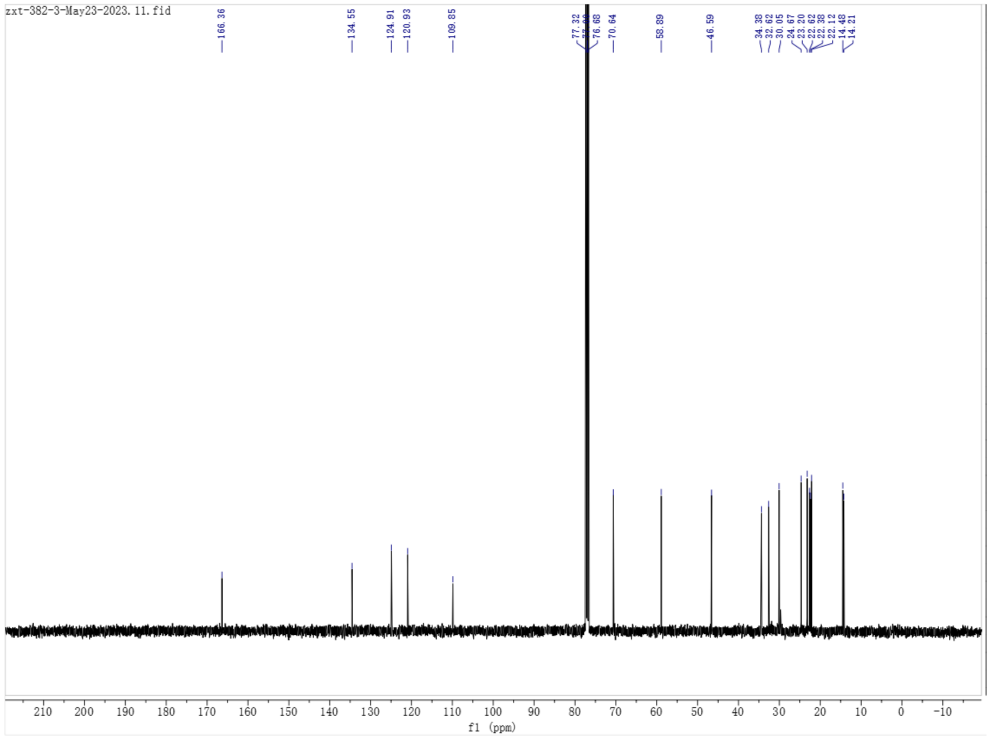

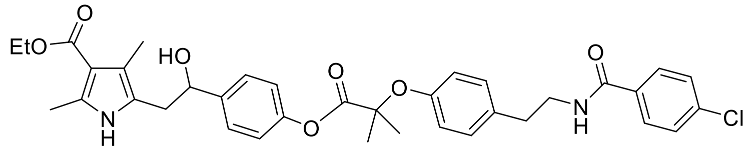
EtO
O
HO
O
O
O
N
H
Cl
N
H

7e

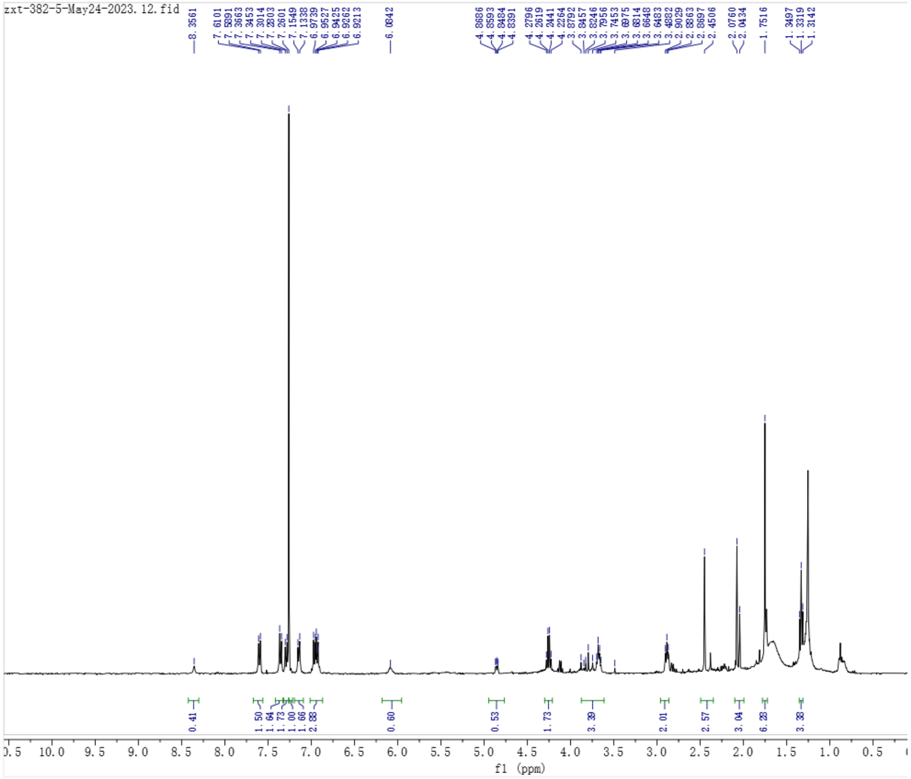
zxt-382-5-May24-2023.12.fid
8.3561
7.6101
7.5891
7.3663
7.3453
7.3014
7.2803
7.2601
7.1549
7.1338
6.9739
6.9527
6.9425
6.9262
6.9213
6.0842
4.8686
4.8593
4.8484
4.8391
4.2796
4.2619
4.2441
4.2264
3.8792
3.8457
3.8246
3.7956
3.7453
3.6975
3.6814
3.6648
3.6483
3.4882
2.9029
2.8863
2.8697
2.4506
2.0760
2.0434
1.7516
1.3497
1.3319
1.3142
0.41
1.50
1.64
1.73
1.00
1.66
2.88
0.60
0.53
1.73
3.39
2.01
2.57
3.04
6.28
3.38
f1 (ppm)

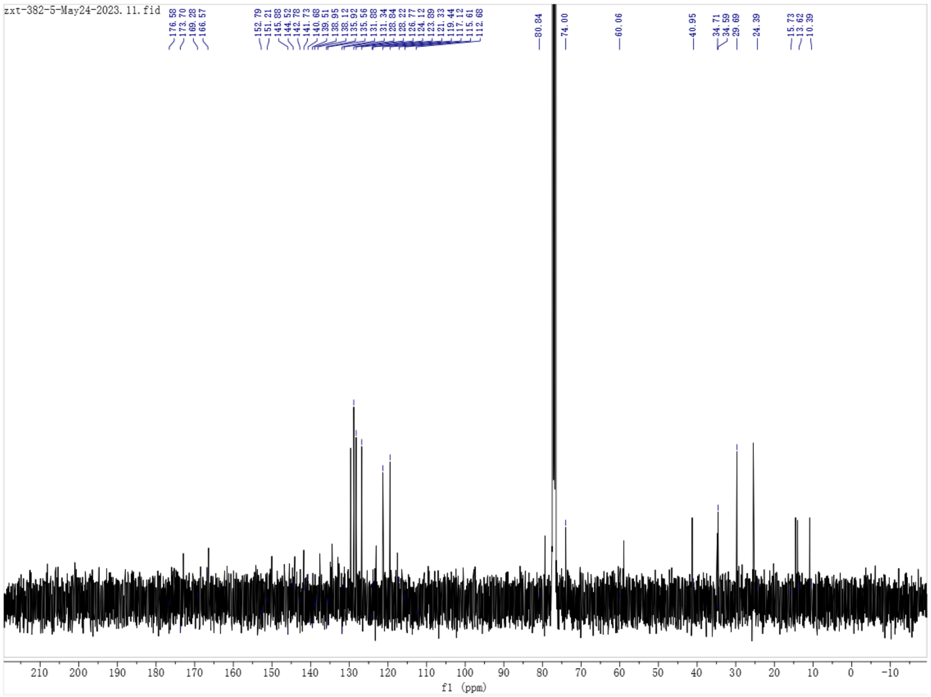
zxt-382-5-May24-2023.11.fid
176.58
173.70
169.28
166.57
152.79
151.21
145.88
144.52
142.78
141.73
140.68
139.51
138.95
138.12
135.92
135.56
131.88
131.34
128.84
128.22
126.77
124.12
123.89
121.33
119.44
117.12
115.61
112.68
80.84
74.00
60.06
40.95
34.71
34.59
29.69
24.39
15.73
13.62
10.39
f1 (ppm)

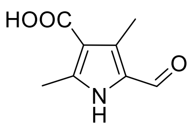

8

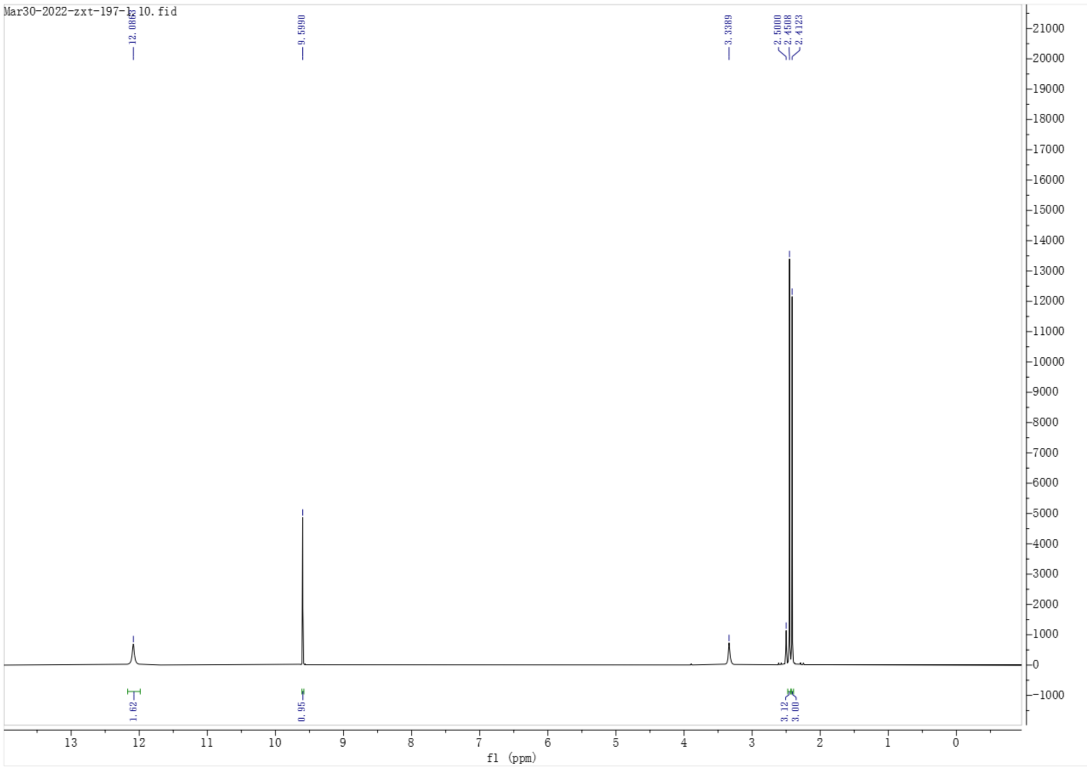
Mar30-2022-zxt-197-1.10.fid
12.086
9.5990
3.3389
2.5000
2.4508
2.4123
1.62
0.95
3.12
3.00
f1 (ppm)

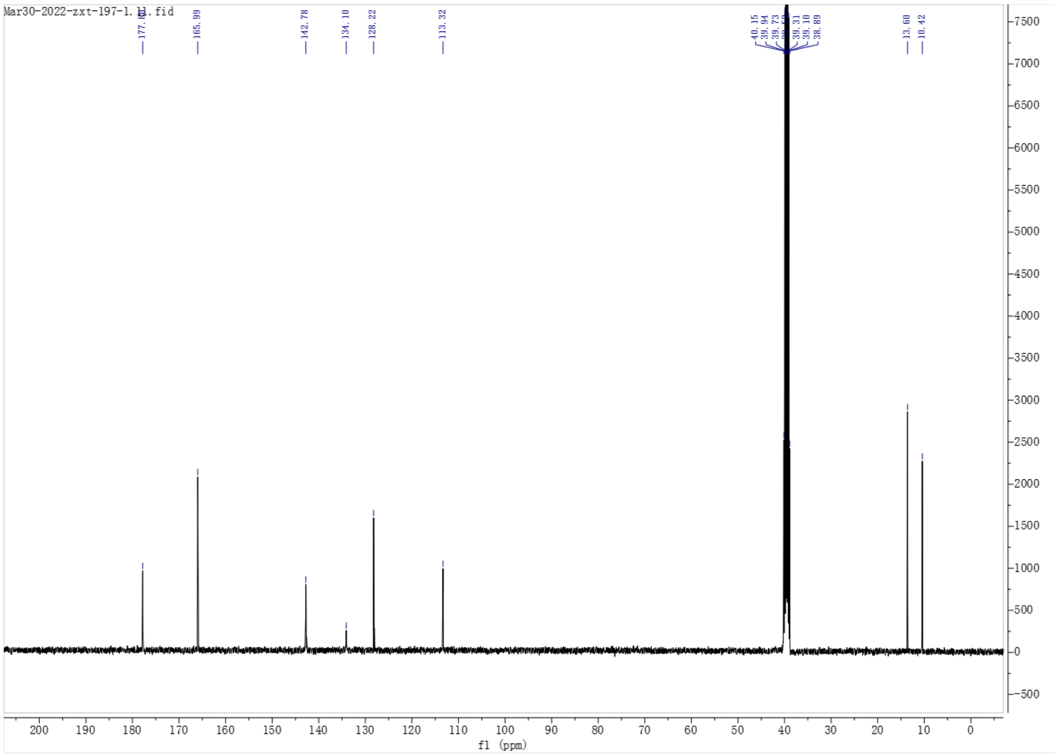
Mar30-2022-zxt-197-1.11.fid
165.99
142.78
134.10
128.22
113.32
40.15
39.94
39.73
39.31
39.10
38.89
13.60
10.42
f1 (ppm)

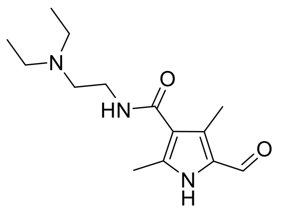

9

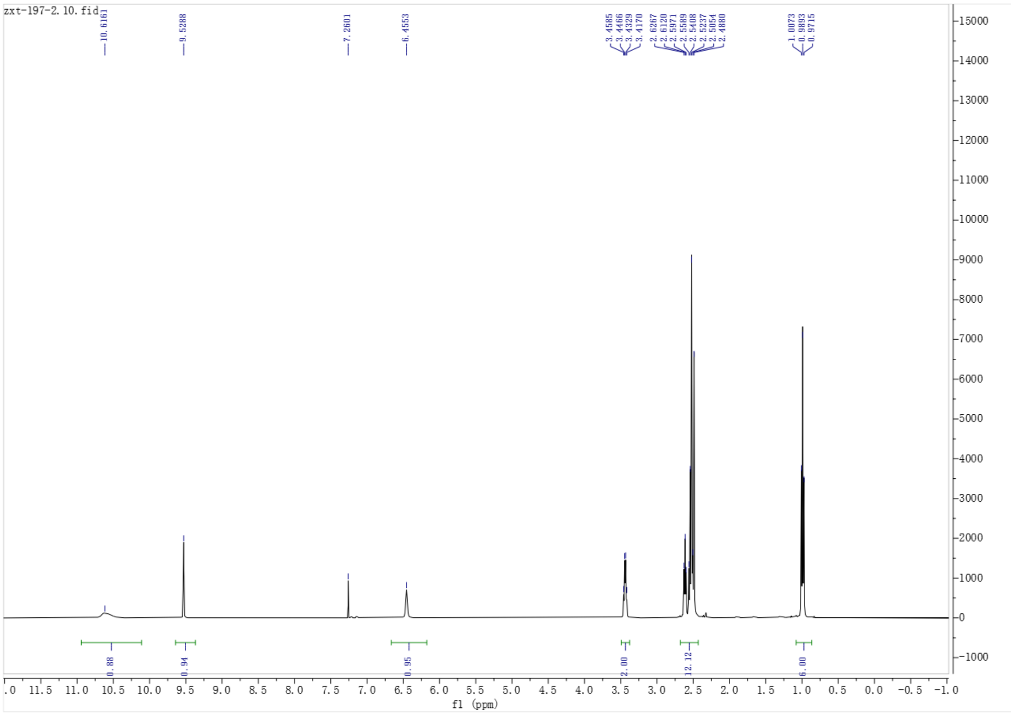

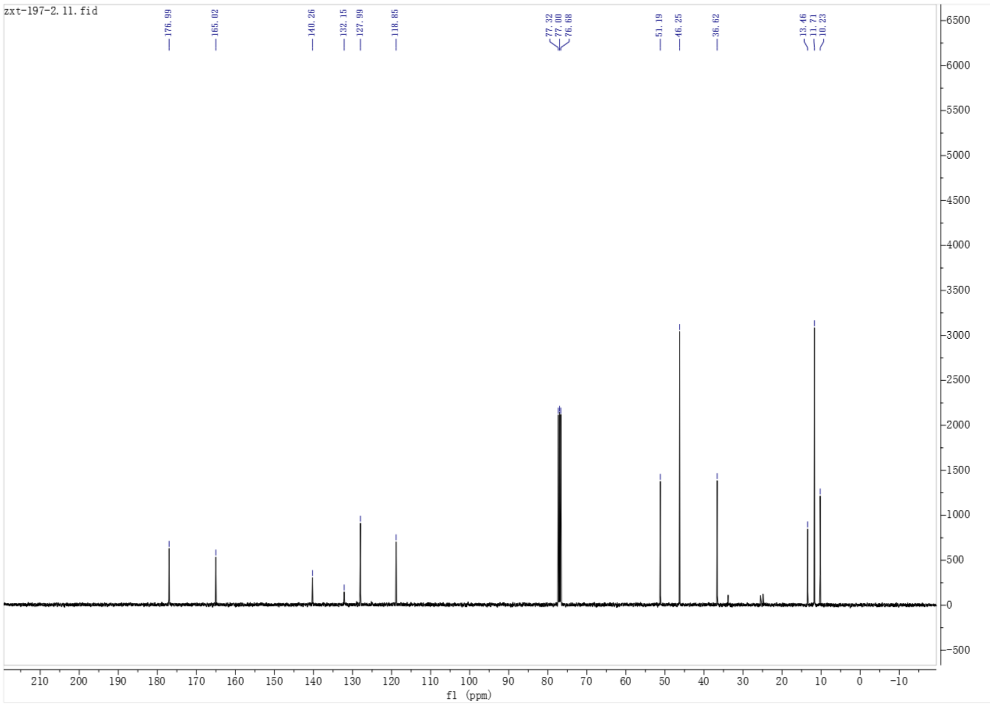

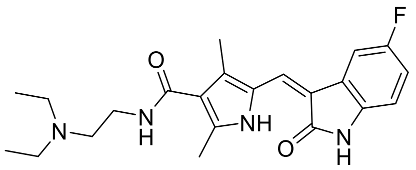

10

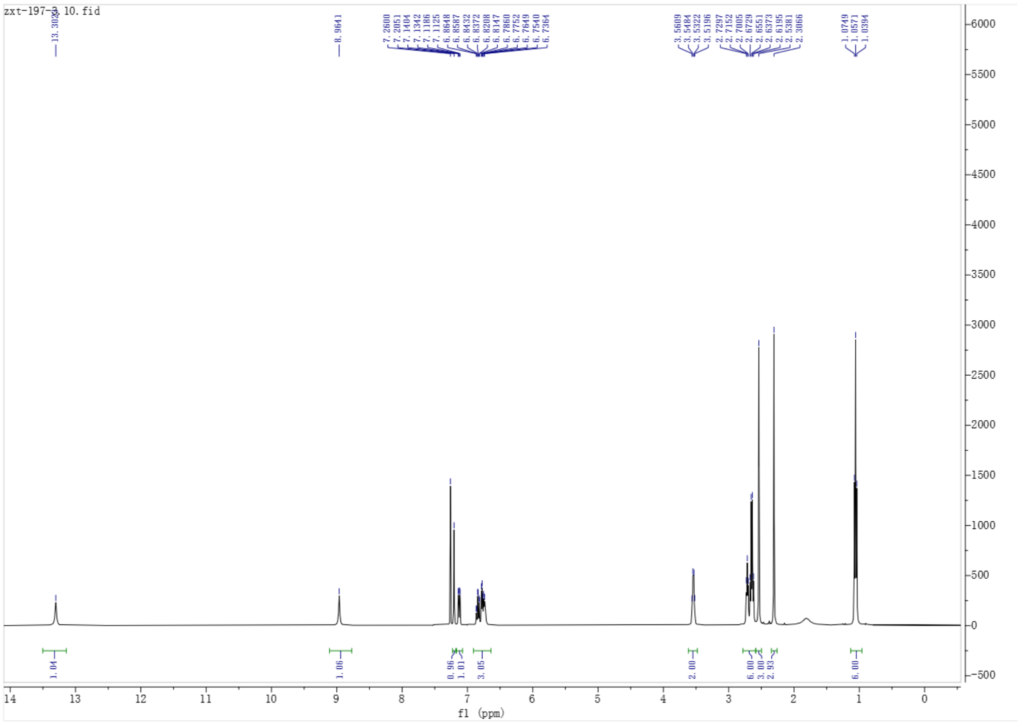

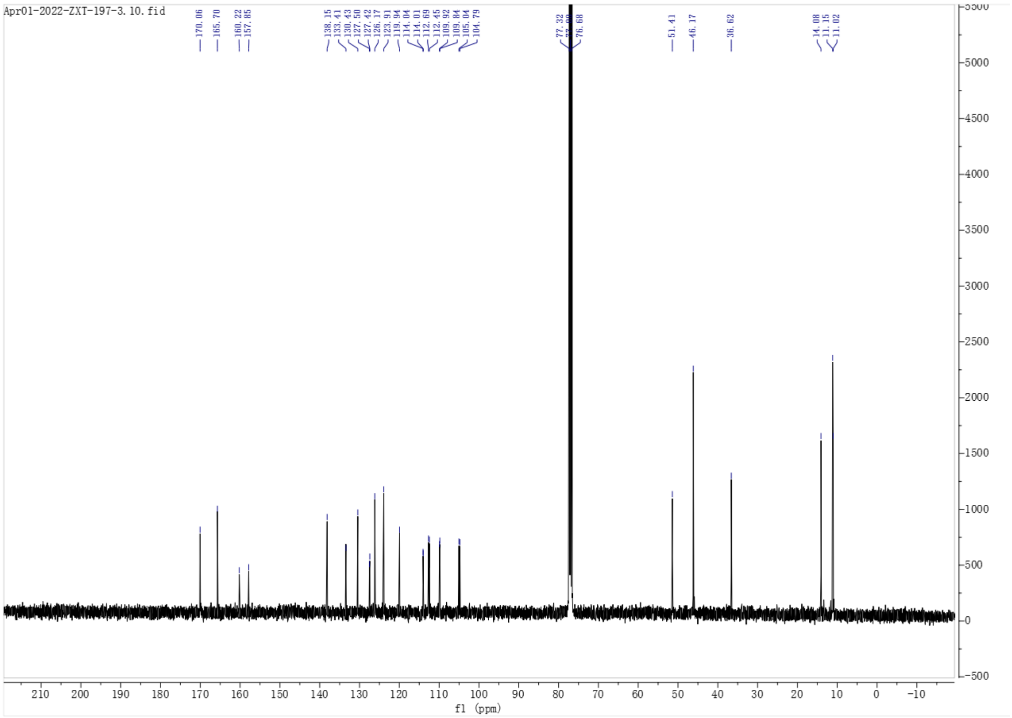

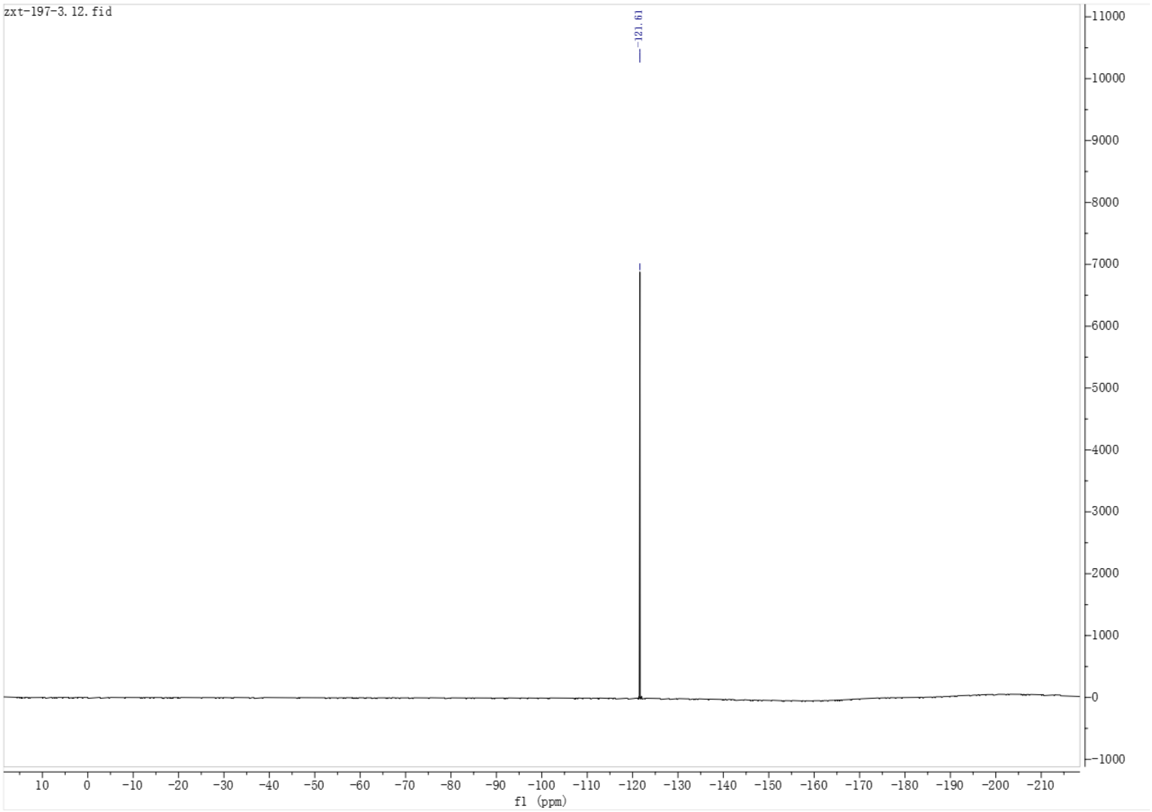

^{19}F NMR spectrum

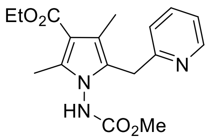

EtO_2C
N
HN
CO_2Me
N

14

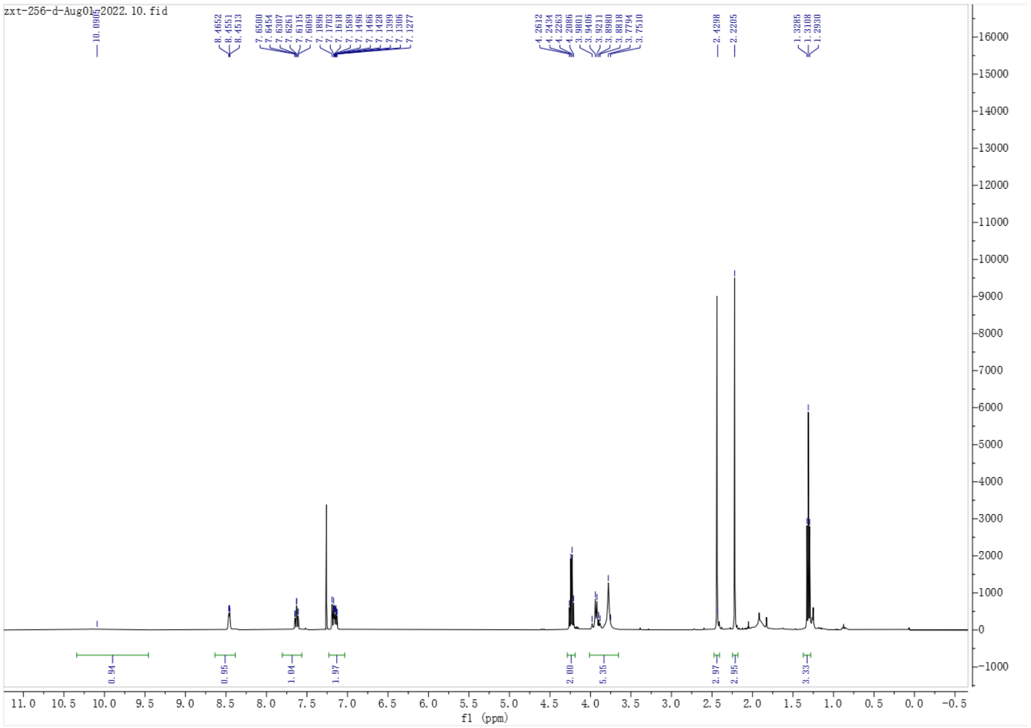

zxt-256-d-Aug01-2022.10.fid
f1 (ppm)

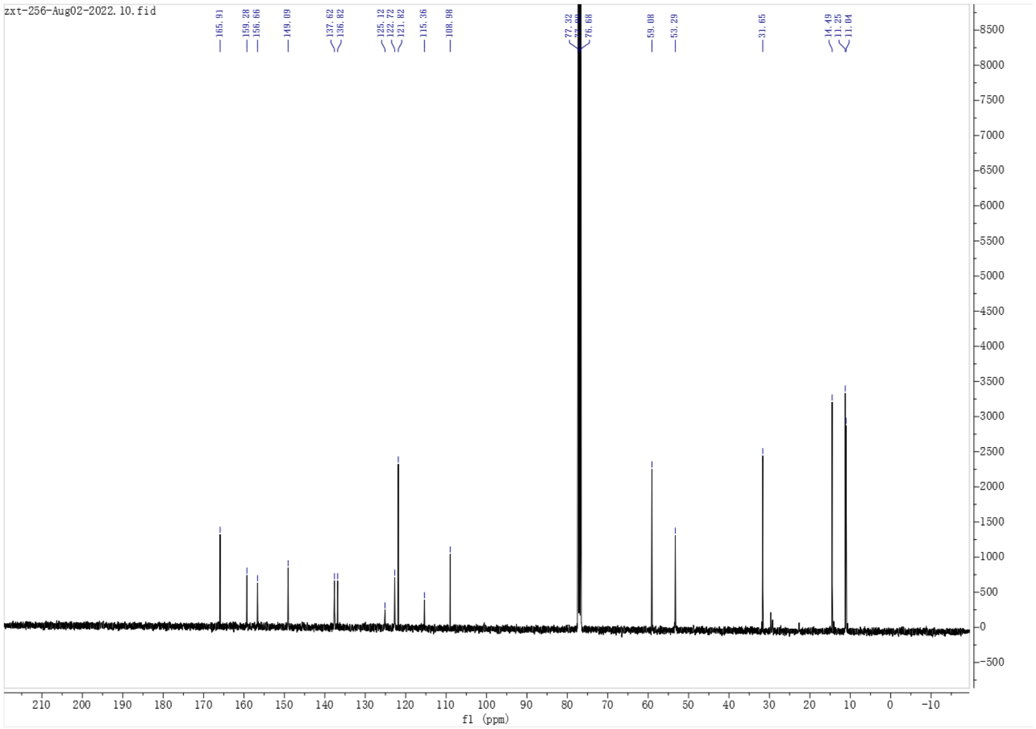

zxt-256-Aug02-2022.10.fid
f1 (ppm)

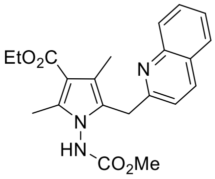
EtO2C
N
N
HN
CO2Me

15

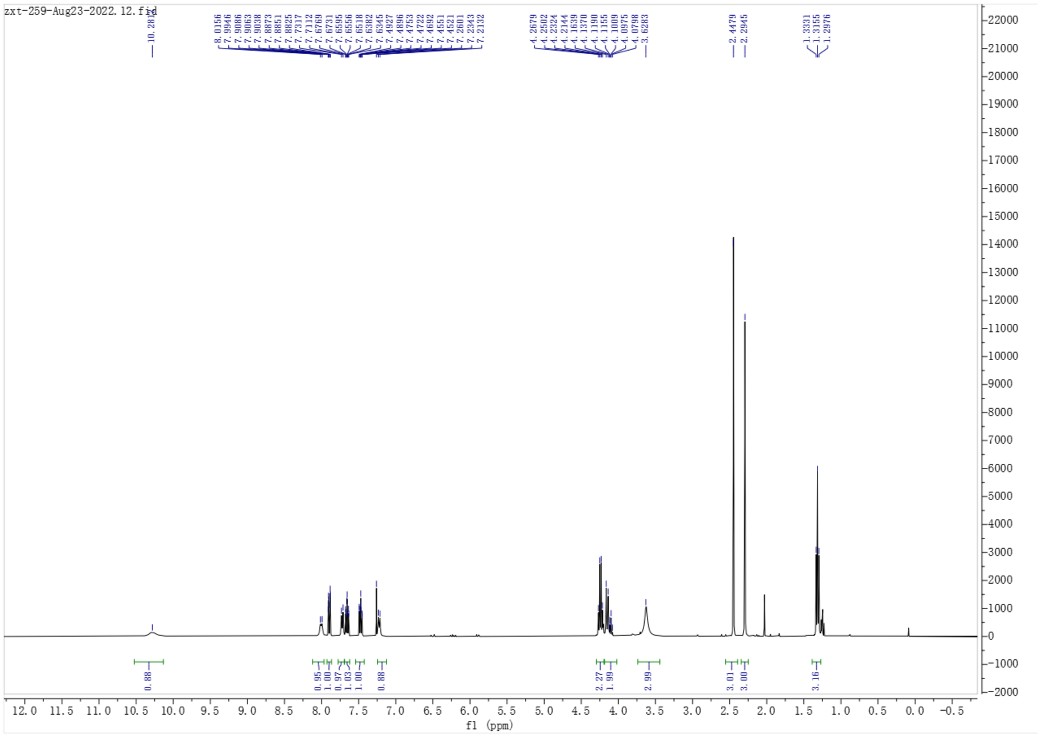
zxt-259-Aug23-2022.12.fid
f1 (ppm)

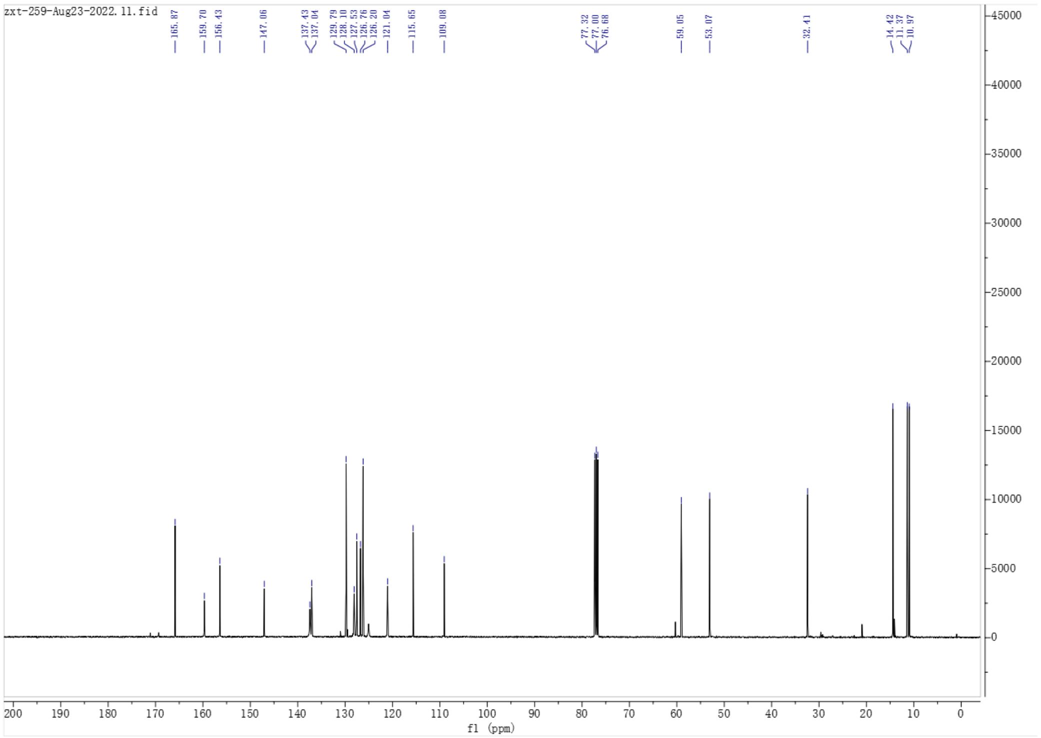
zxt-259-Aug23-2022.11.fid
165.87
159.70
156.43
147.06
137.43
137.04
129.79
128.10
127.53
126.76
126.20
121.04
115.65
109.08
77.32
77.00
76.68
59.05
53.07
32.41
14.42
11.37
10.97
f1 (ppm)

9. References

1 Schultz, A. G. & Hagmann, W. K. Synthesis of indole-2-carboxylic esters. *J. Org. Chem.* **43**, 3391-3393 (1978).

2 Chen, K.-W. *et al.* Application of 3-Alkyl-2-vinylindoles in Catalytic Asymmetric Dearomative (2+3) Cycloadditions. *J. Org. Chem.* **86**, 10427-10439 (2021).

3 Preti, L. *et al.* One-Pot Synthesis of Imidazole-4-Carboxylates by Microwave-Assisted 1,5-Electrocyclization of Azavinyl Azomethine Ylides. *Eur. J. Org. Chem.* **2010**, 4312-4320 (2010).

4 Mei, G.-J. *et al.* Chemo-, regio- and enantioselective dearomative (3+2) reaction of non-functionalized 1-naphthols. *Chem Catal.* **2**, 386-399 (2022).

5 Ballini, R., Barboni, L. & Giarlo, G. Nitroalkanes in Aqueous Medium as an Efficient and Eco-Friendly Source for the One-Pot Synthesis of 1,4-Diketones, 1,4-Diols, δ -Nitroalkanols, and Hydroxytetrahydrofurans. *J. Org. Chem.* **68**, 9173-9176 (2003).

6 Biava, M. *et al.* Identification of a novel pyrrole derivative endowed with antimycobacterial activity and protection index comparable to that of the current antitubercular drugs streptomycin and rifampin. *Biorg. Med. Chem.* **18**, 8076-8084 (2010).

7 Patel, B. H., Heath, S. F. A., Mason, A. M. & Barrett, A. G. M. Efficient two directional syntheses of a homophthalate ester and novel resorcylate oligomers. *Tetrahedron Lett.* **52**, 2258-2261 (2011).

8 Piscitelli, F. *et al.* 1-Aryl-5-(1H-pyrrol-1-yl)-1H-pyrazole-3-carboxamide: An effective scaffold for the design of either CB1 or CB2 receptor ligands. *Eur. J. Med. Chem.* **46**, 5641-5653 (2011).

9 Cho, H., Madden, R., Nisanci, B. & Török, B. The Paal–Knorr reaction revisited. A catalyst and solvent-free synthesis of underivatized and N-substituted pyrroles. *Green Chem.* **17**, 1088-1099 (2015).

10 Mari, G., Favi, G., Santeusanio, S., Mantellini, F. & De Crescentini, L. A practical and effective method for the N–N bond cleavage of N-amino-heterocycles. *Org. Chem. Front.* **6**, 3408-3414 (2019).

11 Sun, L. *et al.* Discovery of 5-[5-Fluoro-2-oxo-1,2- dihydroindol-(3Z)-ylidenemethyl]-2,4-dimethyl-1H-pyrrole-3-carboxylic Acid (2-Diethylaminoethyl)amide, a Novel Tyrosine Kinase Inhibitor Targeting Vascular Endothelial and Platelet-Derived Growth Factor Receptor Tyrosine Kinase. *J. Med. Chem.* **46**, 1116-1119 (2003).
